# Combining single-cell analysis and molecular docking techniques to construct a prognostic model for colon adenocarcinoma and uncovering inhibin subunit βb as a novel therapeutic target

**DOI:** 10.3389/fimmu.2024.1524560

**Published:** 2025-01-09

**Authors:** Qinqing Wu, Lu Ye, Yuwei Wu, Mengyu Zhao, Jiaxin Lu, Yanping Yu, Yixiao Niu, Luxiao Zhang, Peijun Zuo

**Affiliations:** ^1^ Department of Preventive Medicine, Shantou University Medical College, Shantou, China; ^2^ School of Public Health, Shantou University, Shantou, China; ^3^ Department of Breast Surgical Oncology, National Cancer Center/National Clinical Research Center for Cancer/Cancer Hospital, Chinese Academy of Medical Sciences and Peking Union Medical College, Beijing, China; ^4^ Department of General Surgery, Union Hospital affiliated to Fujian Medical University, Fuzhou, China; ^5^ Department of Neurosurgery, First Affiliated Hospital of Anhui Medical University, Hefei, China

**Keywords:** anoikis, colon adenocarcinoma, single-cell analysis, molecular docking, INHBB 1

## Abstract

**Background:**

Colon adenocarcinoma (COAD) is a malignancy with a high mortality rate and complex biological characteristics and heterogeneity, which poses challenges for clinical treatment. Anoikis is a type of programmed cell death that occurs when cells lose their attachment to the extracellular matrix (ECM), and it plays a crucial role in tumor metastasis. However, the specific biological link between anoikis and COAD, as well as its mechanisms in tumor progression, remains unclear, making it a potential new direction for therapeutic strategy research.

**Methods:**

We employed transcriptomic data and clinical information from The Cancer Genome Atlas (TCGA) and Gene Expression Omnibus (GEO) to pinpoint differentially expressed anoikis-related genes (ARGs) in COAD. Using Cox proportional hazards models and Lasso regression analysis, we developed a prognostic signature derived from these ARGs. We also investigated the roles and interactions of these genes in the tumor microenvironment by analyzing single-cell RNA sequencing data. Additionally, we employed molecular docking techniques to evaluate the potential of inhibin subunit beta B (INHBB) as therapeutic targets and to assess the binding affinity of candidate drugs. Finally, we used gene knockout techniques to silence the key gene INHBB and explored its biological functions *in vitro*.

**Results:**

In our study, by analyzing the expression differences of ARGs, we successfully classified patients with COAD. Kaplan-Meier survival analysis demonstrated that patients with elevated risk scores experienced poorer prognosis, a finding that was confirmed in both the training and validation cohorts. Additionally, immune infiltration analysis revealed a notable increase in immune cell presence within the tumor microenvironment of high-risk patients. Molecular docking identified potential drug candidates with high binding affinity to INHBB, including risperidone. Furthermore, *in vitro* experiments with INHBB showed that downregulation of its expression in COAD cell lines significantly reduced cellular viability and migration capacity.

**Conclusion:**

In summary, our research, based on the expression characteristics of ARGs, provides new insights into the precise classification, prognosis assessment, and identification of potential therapeutic targets in COAD. It also validates the key role of INHBB in the progression of COAD, establishing the foundation for future personalized treatment strategies.

## 1 Introduction

Colorectal cancer (CC) ranks among the most prevalent cancers globally. According to the 2022 cancer statistics from the World Health Organization, CC ranks third in incidence among all cancer types, with 1,926,425 new cases reported; it also ranks second in mortality, with 904,019 new deaths attributable to CC (1–3). This poses a significant challenge to healthcare systems globally. Among all histopathological types, colon adenocarcinoma (COAD) comprises the majority (4). Currently, surgical resection remains the predominant curative approach; Nonetheless, progress in chemotherapy, targeted therapies, and immunotherapy has introduced new treatment alternatives for patients with COAD (5). Despite these advancements, issues such as tumor specificity, treatment resistance, and the potential for local recurrence or metastasis still negatively impact patient outcomes. Consequently, it is crucial to explore the molecular mechanisms driving COAD progression, investigate early diagnostic techniques, and assess the importance of prognostic biomarkers.

Anoikis is a specific form of apoptosis initiated when cells lose their usual attachment to the extracellular matrix (ECM) (6, 7). This self-destructive process is activated when cells detach from their supportive matrix or surrounding tissues, preventing them from proliferating in inappropriate locations and thereby avoiding the formation of abnormal tissues or cancer. Cancer cells often evade anoikis through various mechanisms, allowing them to survive after detaching from their primary site and metastasize to other tissues or organs, contributing to tumor spread. Recent studies have indicated that anoikis serves as a mechanism by which tumor cells evade apoptosis, closely correlating with tumor aggressiveness, metastatic potential, and prognosis (8, 9). Breast cancer cells exhibit a heightened ability to evade anoikis, particularly pronounced in triple-negative breast cancer (TNBC). These cells promote their survival upon detachment from the ECM by increasing the expression of anti-apoptotic factors (such as Bcl-2) and activating the PI3K/Akt signaling pathway, which enhances their invasive and metastatic potential (10–12). In non-small cell lung cancer (NSCLC) cells, anoikis is also linked to metastatic potential. Some studies suggest that by modulating the expression of integrins and E-cadherin, tumor cells can adjust their dependency on the ECM, allowing them to escape immune detection and apoptosis, thus facilitating their colonization and growth in distant tissues (13). However, comprehensive analyses of the impact of anoikis in COAD remain scarce. Therefore, identifying anoikis-related genes (ARGs) with prognostic significance in COAD is crucial.

Single-cell sequencing is a technique that enables the analysis of genetic information, including genomics, transcriptomics, and epigenomics, at the level of individual cells (14). Unlike traditional bulk sequencing, single-cell sequencing reveals critical details of heterogeneity within cellular populations, such as gene expression differences among various cell types, functional states of specific cell subpopulations, and dynamic changes in cells during developmental processes. Molecular docking is a computational simulation method used to predict the binding modes of small molecules, such as drug compounds, with target proteins. This technique estimates the interactions and energy between molecules to predict the optimal binding conformations and sites of small molecules with receptor proteins, thereby inferring potential drug targets or aiding in the design of novel therapeutics (15).

In this research, we seek to reveal the molecular features and clinical significance of ARGs in COAD by integrating extensive transcriptomic data from The Cancer Genome Atlas (TCGA) and Gene Expression Omnibus (GEO) alongside clinical information, with the goal of developing a prognostic gene model for COAD (16). Utilizing single-cell analysis techniques, we further investigate the roles of these genes within the tumor microenvironment and their interactions with the tumor immune microenvironment (17). Additionally, through molecular docking studies, we will explore the potential of these genes as therapeutic targets and assess the binding affinities of candidate drugs to these targets. *In vitro* experiments will further confirm the expression levels and functions of critical genes identified in the model (18). This research offers fresh perspectives and a foundational framework for the early diagnosis, targeted therapy, and prognostic evaluation of COAD.

## 2 Materials and methods

### 2.1 Data acquisition and preprocessing

RNA-seq data and clinical details for COAD patients were sourced from TCGA database (https://portal.gdc.cancer.gov/). A total of 585 transcriptomic datasets, clinical data (GSE40967), and single-cell datasets from 13 COAD samples (GSE110009) were acquired from the GEO database (https://www.ncbi.nlm.nih.gov/geo/). A total of 576 ARGs were sourced from the GeneCards database (https://www.genecards.org/) and the Harmonizome database (https://maayanlab.cloud/Harmonizome/) (19). To maintain the accuracy and reliability of the analyses, only ARGs with a correlation coefficient exceeding 0.4 were chosen for further examination.

### 2.2 Exploration of Anoikis-related prognostic genes

In this research, we obtained transcriptomic and clinical data from COAD samples within TCGA database. Differential expression analysis was performed using the R package “DESeq2” to identify differentially expressed genes (DEGs) with a fold change of at least 2 and a p-value of less than 0.01. By intersecting these DEGs with ARGs, we identified 134 significantly different genes in COAD. Standardized merging with GEO data led to further prognostic analysis, revealing 37 genes associated with overall survival (OS). Gene copy number variations were assessed using data obtained from the UCSC Xena website (https://xena.ucsc.edu/), while a protein-protein interaction (PPI) network was generated using the online tool STRING to investigate co-expression relationships and potential molecular interactions among the genes (20, 21).

### 2.3 Consistency cluster analysis

We performed an in-depth clustering analysis of samples using the K-means clustering algorithm from the “ConsensusClusterPlus” package, setting the maximum number of clusters to 9 (maxK = 9). Through detailed analysis of the consistency matrix of clustering results, we successfully determined the optimal number of clusters to be 2, which was validated using principal component analysis (PCA) (22). Heatmap analysis demonstrated relationships between alterations in gene expression and clinical characteristics, while survival curve analysis showed that Cluster A had a better prognosis than Cluster B, revealing notable differences in immune cell expression between the two clusters. Gene Set Variation Analysis (GSVA) and Gene Set Enrichment Analysis (GSEA) were utilized to further investigate biological functions and pathway activity differences across the various subtypes.

### 2.4 Construction of prognostic model

We utilized the “createDataPartition” function in R to randomly partition the dataset into training and testing subsets. Univariate Cox regression analysis was performed to identify significant genes, followed by Lasso regression and cross-validation to optimize a multivariate Cox model (23). The “glmnet” package facilitated the calculation of risk scores for COAD patients based on the refined model. Patients were categorized into high-risk and low-risk groups based on the median risk score. The risk score was calculated using the formula: Risk score = ∑(expi * βi), where expi represents the expression level of each gene and βi denotes the corresponding regression coefficient. Kaplan-Meier survival curves were plotted using the “survival” package, and time-dependent receiver operating characteristic (tROC) curves were generated with the “timeROC” package to evaluate the model’s predictive accuracy regarding patient survival.

### 2.5 Construction of nomograms

Nomograms were developed using the “rms” and “regplot” packages in R to forecast survival in COAD patients, taking into account factors such as age, sex, and stage. Calibration curves validated the precision of the nomogram’s predictions for survival rates at 1 year, 3 years, and 5 years (24). Decision curve analysis revealed that the nomogram’s predictive ability was superior to that of individual clinical factors. Performance assessment of the risk score showed that its accuracy in prediction surpassed that of conventional clinical indicators.

### 2.6 Risk score correlation with clinical variables

Both univariate and multivariate Cox regression analyses indicated a significant association between age, stage, and risk scores with the survival duration of patients with COAD. The risk score model constructed using these variables demonstrated significant differences in survival times across patients of varying gender, age, and stage.

### 2.7 Enrichment analysis

Using R packages “clusterProfiler” and “org.Hs.eg.db,” we conducted Gene Ontology (GO) and Kyoto Encyclopedia of Genes and Genomes (KEGG) enrichment analysis on differentially expressed genes across different risk groups. GO enrichment analysis revealed distinct patterns in the distribution of differentially expressed genes between high-risk and low-risk groups, spanning biological processes (BP), molecular functions (MF), and cellular components (CC) (25). Additionally, KEGG pathway analysis highlighted the key pathways enriched by these differentially expressed genes.

### 2.8 Immune cell infiltration

We employed the CIBERSORT algorithm for precise calculation of immune cell infiltration proportions (26). A heatmap was utilized to visually represent the strength and directionality of correlations among different immune cell types. Specific immune cell subpopulations exhibited significant expression differences across risk groups. Furthermore, we employed the “estimate” package to calculate tumor microenvironment scores (TME scores) to evaluate the infiltration levels of immune and stromal cells within the tumor microenvironment (27).

### 2.9 Single-cell analysis

We performed thorough quality control and filtering of scRNA-seq data utilizing the “Seurat” and “SingleR” R packages. Each gene was required to be expressed in a minimum of three cells and to have an expression level of at least 50 genes. The “subset” function was applied to filter cells based on the criteria of having more than 50 genes and less than 5% mitochondrial gene expression. Data normalization was carried out using the “NormalizeData” function with a scaling factor of 10,000. The “FindVariableFeatures” function was then employed to identify genes exhibiting high variability, selecting the 1,500 genes with the most significant expression fluctuations for further analysis. Through PCA dimensionality reduction and t-SNE clustering analysis, we successfully identified multiple distinct cell clusters and recognized several marker genes (28). We used the SingleR algorithm in R to annotate cell types in our single-cell dataset by comparing it to a reference dataset. The results were visualized using t-SNE, which provided a clear representation of the cellular landscape. Additionally, we applied the Monocle algorithm to infer the developmental trajectories of the cells and construct dynamic models of cellular differentiation.

### 2.10 Candidate drug prediction and molecular docking

We conducted an extensive drug screening utilizing the gene prediction drug online resource (https://maayanlab.cloud/Enrichr/), selecting drugs based on an adjusted p-value of less than 0.05. The 2D structures of these drugs were retrieved from the PubChem database (https://pubchem.ncbi.nlm.nih.gov/) and converted into 3D models using “Chem3D.” Protein structure data for the relevant genes were sourced from the PDB database (http://www.rcsb.org/). The receptor was prepared using “PyMol,” removing water molecules and small ligand compounds. Molecular docking was conducted to identify active pockets using “Autodock Vina v.1.5.7” (29).

### 2.11 Cell transfection

The cell lines present in this study were obtained from The Cell Resource Center of Shanghai Life Sciences Institute. Firstly, lipo3000 transfection reagent was mixed with siRNA to form transfection complex. These complexes are then added to cells in a petri dish to bind to the cell membrane and enter the cell. After transfection, the cells were cultured for 24 hours under suitable culture conditions so that the foreign nucleic acid could be expressed or function. Finally, PCR was used to detect the transfection effect and the knockdown of exogenous genes. Primer information and siRNA sequence are **Supplementary Table 1**.

### 2.12 Western blotting

Total protein from the cells was extracted using RIPA Lysis Buffer (Beyotime, P0013B), and its concentration was determined using the Enhanced BCA Protein Assay Kit (Beyotime, P0010). The results were measured by ImageJ software.

### 2.13 Colony

After diluting the cells to an appropriate concentration, they are evenly seeded into sterile 6-well plates and then cultured in an incubator for 10-14 days until visible cell colonies form. Next, the cells are fixed with formaldehyde and stained with crystal violet.

### 2.14 Wound healing

Cultivate the cells to near confluence and then gently create a straight line scratch in the cell layer at the bottom of the culture dish using a sterile pipette. Subsequently, remove any cell debris from the scratch area and rinse once with fresh medium to eliminate floating cells. After that, place the cells back in the incubator and observe and capture images of the scratch healing at 0 and 48 hours to assess the rate and capacity of cell migration.

### 2.15 Statistical analysis

In this study, we performed logarithmic transformation and batch correction to standardize the data. All data analyses and graphical visualizations were performed using R software (version 4.3.3). To explore the co-expression relationships among genes, we employed Pearson correlation analysis. Furthermore, Spearman correlation analysis was performed to assess the association between risk scores and levels of immune cell infiltration. A comprehensive predictive model was established by combining univariate, Lasso, and multivariate Cox regression analyses. For comparative evaluations, t-tests were utilized to determine differences between two groups, while one-way ANOVA was applied to examine statistical variations across multiple groups. A p-value of less than 0.05 was deemed significant, with * denoting P < 0.05, ** indicating P < 0.01, and *** representing P < 0.001.

## 3 Results

### 3.1 Prognostic genes and subtype classification based on anoikis-related genes in COAD

**Figure 1:**
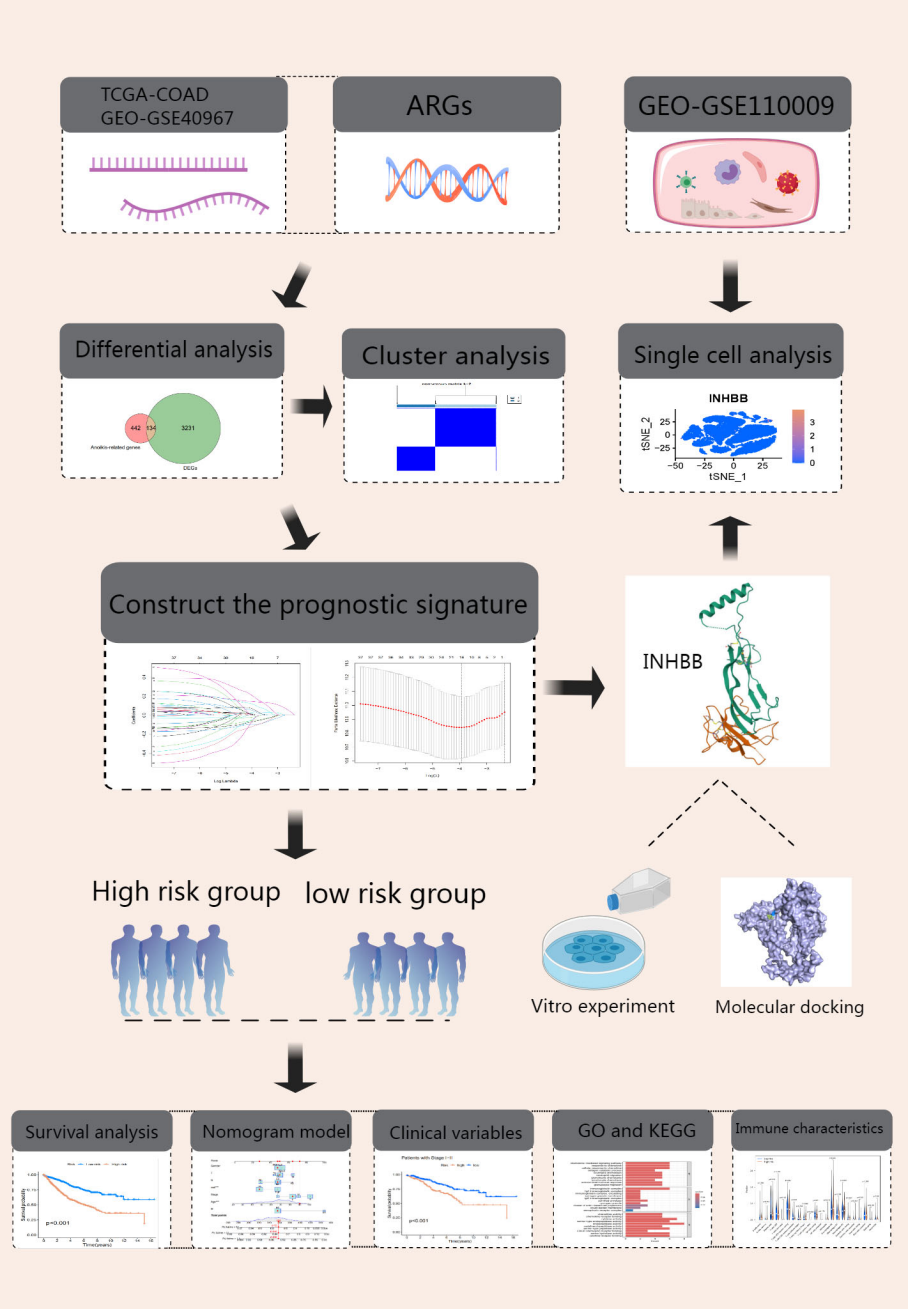
Experimental Design Flowchart.

**Figure 2:**
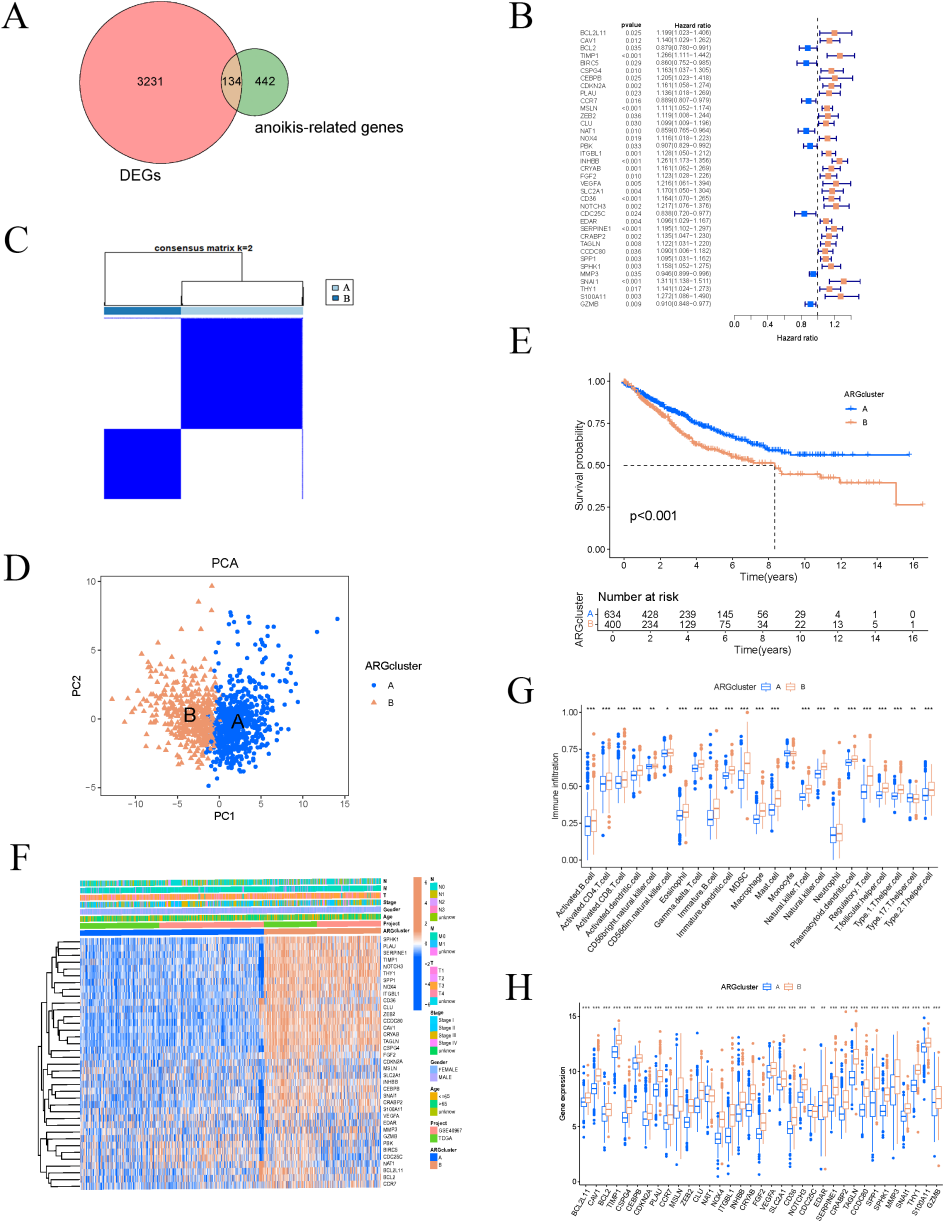
Consensus clustering analysis of ARGs. **(A)** Differential expression between COAD and ARGs. **(B)** Univariate Cox regression forest plot. **(C)** Divide COAD patients into two subgroups through consensus clustering analysis. **(D)** PCA for classification. **(E)** Survival analysis based on classification. **(F)** Thermogram combining typing with clinical characteristics of samples. **(G–H)** Differences in ARGs and immune cells across different clusters. These symbols (* for P < 0.05, ** for P < 0.01, *** for P < 0.001) are used to denote the significance levels of statistical results.

Our research workflow is clearly illustrated (**Figure 1**). We collected transcriptomic data and clinical details from 483 COAD samples and 41 adjacent normal samples via TCGA database. After data organization, we merged the samples and converted probe IDs into gene IDs. We then compared gene expression levels between tumor and normal tissues, performing differential analysis using the “DESeq2” package (fold change = 2, p-value = 0.01). The intersection with ARGs yielded 134 significantly different genes in COAD (**Figure 2A**). We then standardized and merged the transcriptomic dataset of 585 samples and corresponding clinical data from the GEO database with TCGA dataset, extracting the expression levels of DEGs. Employing survival status and OS as outcome measures, univariate Cox regression analysis identified 37 prognostic genes, which are depicted in a forest plot (**Figure 2B**). The prognostic network diagram depicted in **Supplementary Figure 1A** illustrates the co-expression relationships among 29 high-risk and 8 low-risk genes. We subsequently displayed the frequency of copy number increases or decreases for specific genes within the samples, indicating the prevalence of gene variations across the samples, as shown in **Supplementary Figure 1B**. A copy number circle plot depicted in Supplementary **Figure 1C** shows the frequency of gene copy numbers at corresponding chromosomal positions. We constructed a PPI network for the anoikis-related DEGs, as shown in **Supplementary Figure 1D**, identifying genes with protein interaction relationships through connecting lines. The correlation network diagram in **Supplementary Figure 1E** illustrated the strength of correlations among different genes.

**Figure 3:**
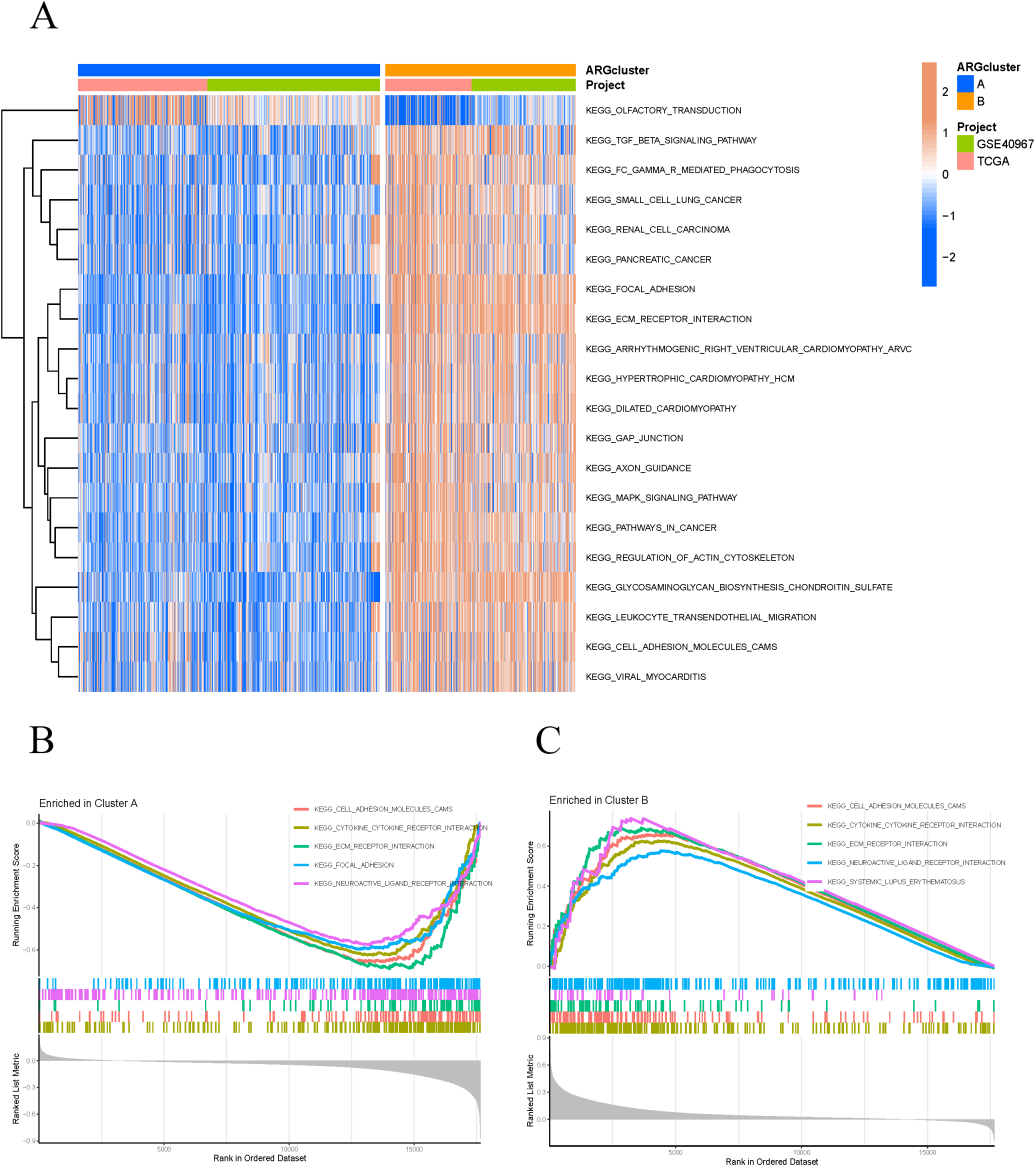
GSVA and GSEA analysis. **(A)** Differential expression of multiple biological pathways in different clusters and projects. **(B, C)** The GSEA pathways significantly enriched in clusters A and B.

Based on the expression levels of ARGs, the samples were classified into two subtypes, A and B (**Figure 2C**). PCA demonstrated that the subtyping effectively separated the samples into distinct groups (**Figure 2D**). To further investigate the differences between subtypes A and B, survival curves confirmed a significant disparity in patient survival (p < 0.001), with subtype A exhibiting superior prognosis compared to subtype B (**Figure 2E**). The subtype heatmap integrated subtype classifications with clinical characteristics, providing a detailed analysis of the distribution of upregulated and downregulated genes (**Figure 2F**). We also examined the differential expression of ARGs and immune cells between subtypes A and B (**Figures 2G, H**). Subsequently, GSVA and GSEA analyses were performed to investigate the biological functions and pathways associated with the different subtypes, emphasizing the discrepancies in biological pathway activities (**Figure 3A**). The findings indicated that cell adhesion molecules (CAMs), cytokine-cytokine receptor interactions, ECM receptor interactions, focal adhesion, and neuroactive ligand-receptor interactions were significantly reduced in subtype A (**Figure 3B**). Conversely, subtype B exhibited increased expression of CAMs, cytokine-cytokine receptor interactions, ECM receptor interactions, neuroactive ligand-receptor interactions, and systemic lupus erythematosus (**Figure 3C**).

### 3.2 Construction of a nine-gene prognostic model

**Figure 4:**
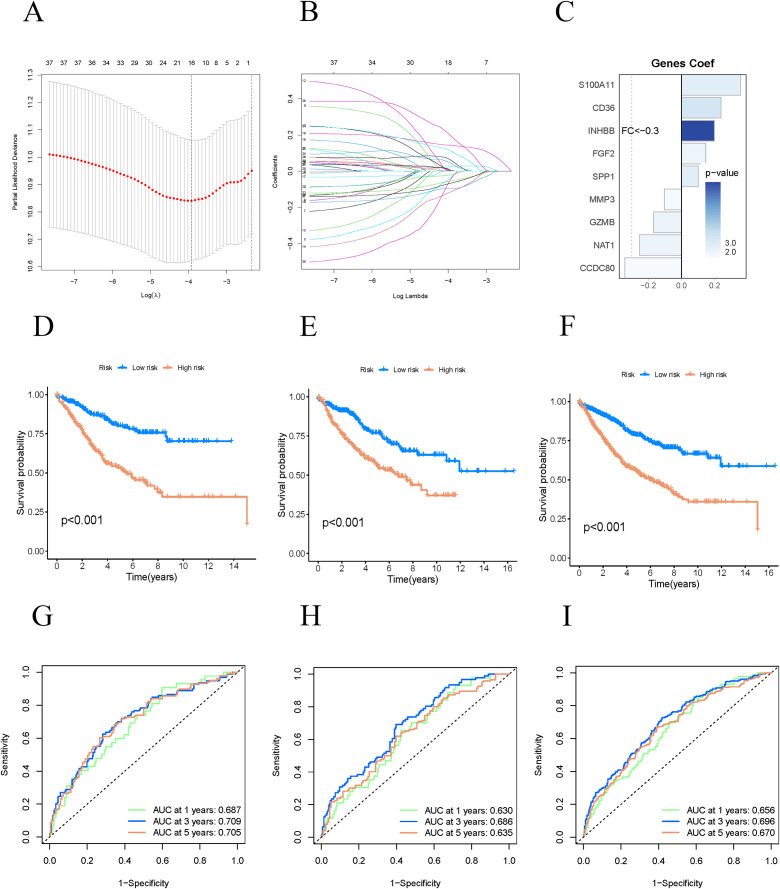
Constructing a prognostic model. **(A–C)** Constructing prognostic related model genes using Lasso regression. **(D–F)** The K-M curves of the training group, validation group, and all groups show the prognosis of COAD patients in high-risk and low-risk groups. **(G–I)** ROC curves of three groups of queues for one year, three years, and five years.

We integrated a transcriptome dataset of 1,034 samples with survival data from TCGA and GEO databases, randomly partitioning it into a training cohort (n=517) and a test cohort (n=517). We employed the Least Absolute Shrinkage and Selection Operator (LASSO) to construct a regression model, identifying the point of minimal error through cross-validation (**Figures 4A, B**). Subsequently, we performed multivariate Cox regression analysis to enhance the model, selecting nine genes as risk features: NAT1, INHBB, FGF2, CD36, CCDC80, SPP1, MMP3, S100A11, and GZMB (**Figure 4C**). The risk score for COAD patients was computed using the following formula: Risk Score (RS)=(−0.25374×NAT1 expression)+(0.19775×INHBB expression)+(0.14497×FGF2 expression)+(0.23801×CD36 expression)+(−0.34139×CCDC80 expression)+(0.09980×SPP1 expression)+(−0.10291×MMP3 expression)+(0.35502×S100A11 expression)+(−0.16830×GZMB expression) Using the median risk score, we stratified the samples into high-risk and low-risk groups within both the training and test cohorts. We evaluated our model’s predictive efficacy for OS in COAD patients across all cohorts. Kaplan-Meier survival analysis revealed that high-risk COAD patients experienced significantly poorer OS in all three cohorts (**Figures 4D–F**). The ROC curves for the three cohorts showed AUC values exceeding 0.6, specifically 0.687, 0.709, and 0.705 for 1-year, 3-year, and 5-year survival, respectively (**Figures 4G–I**). These results indicate that our model is highly accurate in predicting survival outcomes for COAD patients at 1, 3, and 5 years.

### 3.3 Construction and validation of a prognostic nomogram for COAD survival (clinical and molecular features)

**Figure 5:**
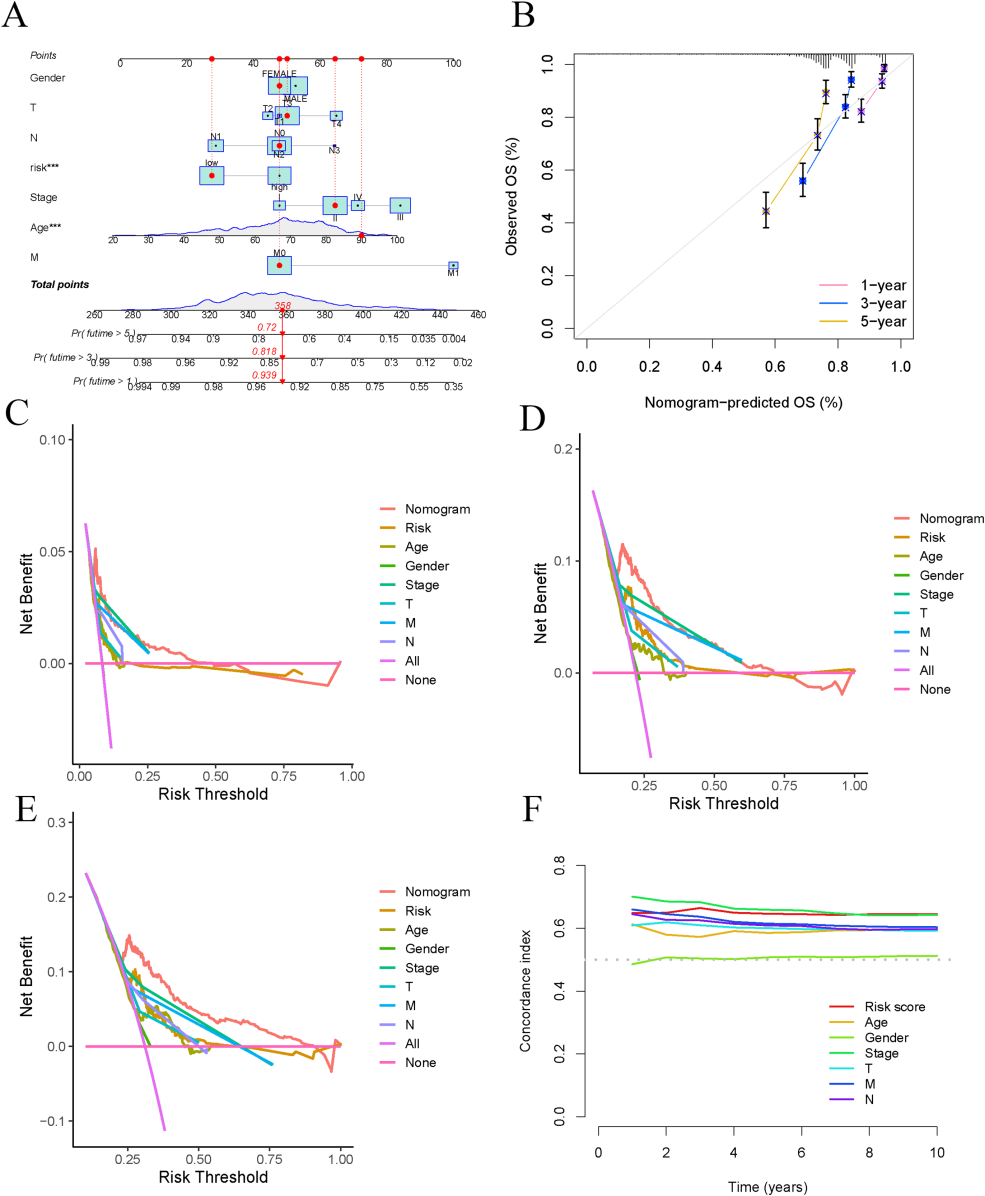
Establish and validate prognostic Nomogram. **(A)** Nomogram validation of OS in COAD patients. **(B)** Verify the predictive ability of Nomogram through calibration curves. **(C–E)** DCA curves for risk scores and clinical characteristics (1 year, 3 years, and 5 years). **(F)** C-index used to evaluate the performance of predictive models.

We constructed a nomogram (**Figure 5A**) that aggregates scores from each clinical characteristic (age, sex, stage) to predict patient survival. To assess the predictive performance of the nomogram, we generated calibration curves and decision curves. The calibration curves showed high accuracy in forecasting 1-year, 3-year, and 5-year survival using the nomogram (**Figure 5B**). The decision curves for 1 year, 3 years, and 5 years indicated that the nomogram’s predictive power significantly exceeded that of other clinical factors (**Figures 5C–E**). In analyzing the survival rates of COAD patients, we compared the effectiveness of ARG features. Our findings revealed that the risk score exhibited the highest C-index, affirming its predictive accuracy and surpassing traditional clinical indicators, including pathological stage, age, and sex (**Figure 5F**).

**Figure 6:**
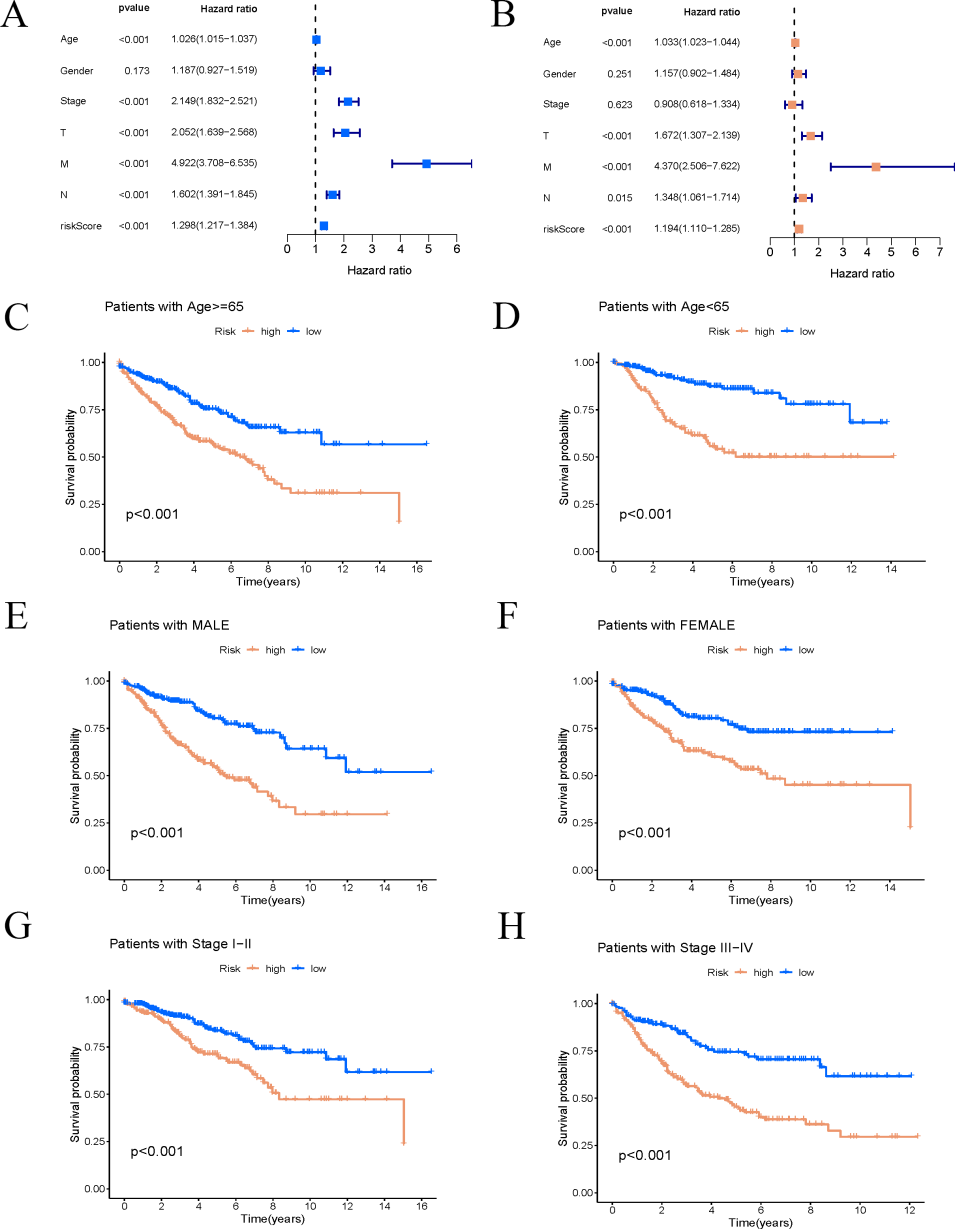
Risk score is highly correlated with clinical variables. **(A, B)** Univariate and multivariate Cox regression forest plots of risk score and clinical features. **(C–H)** K–M survival curves of patients stratified by different clinical pathological factors.

Univariate Cox regression analysis was conducted to generate a forest plot (**Figure 6A**), showing significant associations between survival time and age, stage, and risk score. Multivariate Cox regression analysis further demonstrated statistical relationships between survival time and age, T, M, N stages, and risk score, as illustrated in a forest plot (**Figure 6B**). Consequently, the risk score calculated based on the nine ARGs can predict the survival rate of COAD patients. Stratification by age, sex, and stage demonstrated significant differences in survival times between high-risk and low-risk groups across all categories (p < 0.01). High-risk patients showed reduced survival durations compared to those classified as low-risk (**Figures 6C–H**).

### 3.4 Enrichment analysis and immune microenvironment analysis reveal biological mechanisms and clinical relevance

**Figure 7:**
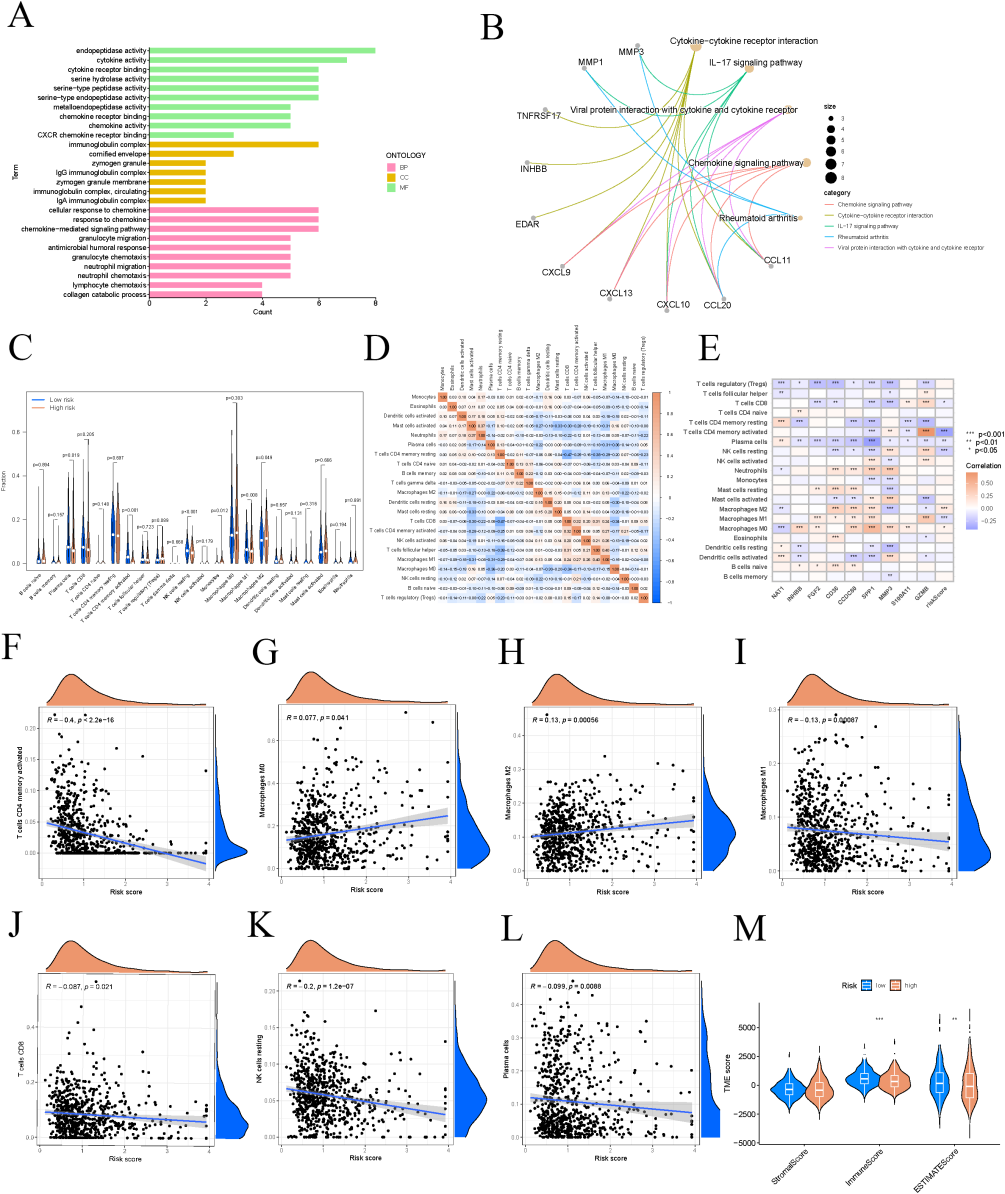
Different risk groups have different immune characteristics. **(A)** The GO signaling pathway involves biological processes in BP, MF, and CC. **(B)** Pathways significantly enriched in KEGG. **(C)** Differential analysis of immune cells in different risk groups. **(D)** Correlation heatmap of immune cells. **(E)** The correlation strength between model genes and immune cell types. **(F–L)** Immune cells significantly correlated with risk scores. **(M)** Differential assessment of TME scores. These symbols (* for P < 0.05, ** for P < 0.01, *** for P < 0.001) are used to denote the significance levels of statistical results.

To examine the distribution patterns of gene sets across BP, MF, and CC, we conducted GO enrichment analysis using differentially expressed genes from the high and low-risk groups. GO analysis indicated significant enrichment in cellular response to chemokine, endopeptidase activity, and immunoglobulin complex (**Figures 7A**). KEGG pathway analysis revealed that the differentially expressed genes were mainly enriched in pathways including cytokine-cytokine receptor interaction, IL-17 signaling pathway, viral protein interactions with cytokines and receptors, chemokine signaling pathway, and rheumatoid arthritis (**Figures 7B**). Notable differences in expression levels were found for plasma cells, activated CD4 memory T cells, resting NK cells, monocytes, M1 macrophages, and M2 macrophages across the different risk categories (**Figure 7C**). We conducted a differential analysis of immune cells between high-risk and low-risk groups, illustrated by a heatmap that shows the correlation strength and direction among various immune cell types, such as monocytes, eosinophils, and activated dendritic cells (**Figure 7D**). A heatmap illustrating the correlations between immune cells, model genes, and risk scores was also presented (**Figure 7E**). Notably, risk scores exhibited significant expression differences with M0 macrophages, M1 macrophages, M2 macrophages, resting NK cells, plasma cells, activated CD4 memory T cells, and CD8 T cells, with the strongest correlation identified with activated CD4 memory T cells (R = -0.4, p < 2.2e−16) (**Figures 7F–L**). Patients in the low-risk group generally exhibit higher TME scores, which may be associated with stronger immune responses and lower tumor purity, thereby potentially having a positive impact on patient treatment responses and survival outcomes. The violin plot demonstrated statistically significant differences in tumor TME scores, including immunescore and estimatescore, between high and low-risk groups (**Figure 7M**).

### 3.5 Single-cell analysis identifies key gene expression and cell types

**Figure 8:**
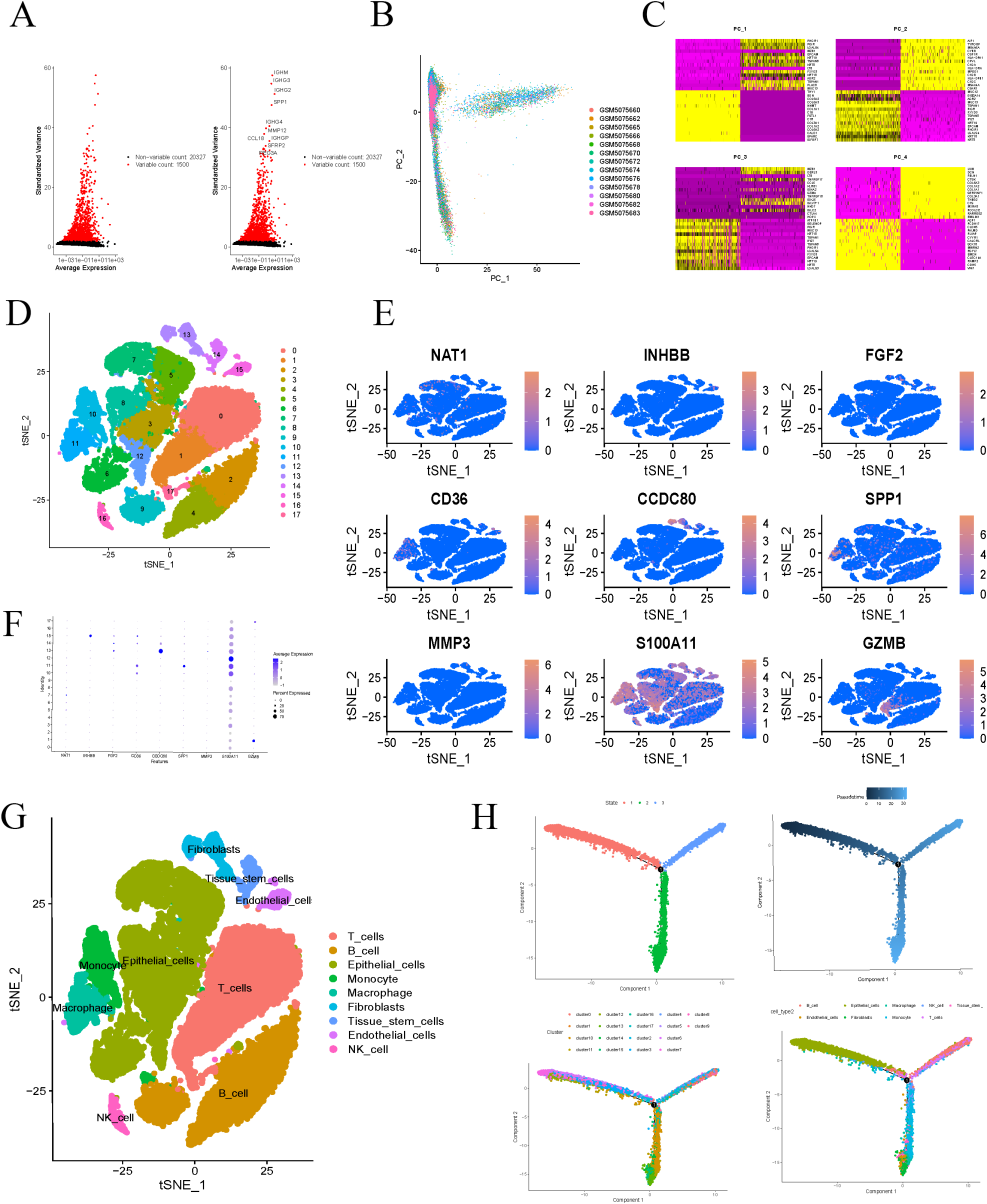
Single-cell analysis. **(A)** Select the top 1500 genes with the largest fluctuations and mark the top 10 genes for ranking. **(B)** Using PCA to reduce the dimensionality of the top 1500 genes in the ranking. **(C)** Heatmap displays the characteristic genes of PC1-PC4. **(D)** Divide all cells into 17 clusters using t-SNE algorithm. **(E, F)** Expression of model genes in 17 clusters. **(G)** After dimensionality reduction using t-SNE algorithm, the classification of cells was demonstrated. **(H)** Cell differentiation trajectory diagram.

We retrieved single-cell datasets for COAD from the GEO database, utilizing gene expression profiles from 13 COAD samples for further analysis. Initial quality control and filtering were performed, isolating the 1,500 most variably expressed genes, with the top ten most variable genes highlighted (**Figure 8A**). PCA was employed to reduce dimensionality on these 1,500 genes (**Figure 8B**), and expression levels of PC1-PC4 feature genes were visualized in a heatmap (**Figure 8C**). TSNE clustering analysis of 15 principal components identified 17 distinct clusters (**Figure 8D**). The differential analysis for each cluster has identified 5,933 marker genes, which are detailed in **Supplementary Table 2**. We conducted a detailed examination of the expression patterns for the nine key genes within the constructed model. Notably, the INHBB gene exhibited significantly elevated expression in cluster 15, while CCDC80 showed higher expression in cluster 13, and S100A11 was notably expressed in cluster 12 (**Figures 8E, F**). Analysis of cell types within each cluster revealed diverse populations, including T cells, B cells, epithelial cells, monocytes, macrophages, fibroblasts, tissue stem cells, and natural killer cells (**Figure 8G**). Further annotation revealed specific gene-cell type associations: INHBB was most highly expressed in endothelial cells, CCDC80 in fibroblasts, and S100A11 in epithelial cells. These findings provide crucial molecular evidence for understanding the roles of different cell types in biological processes. The cells were primarily derived from hematopoietic stem cells and differentiated along two distinct pathways: one pathway leading to the formation of epithelial-like cells, and the other leading to the generation of monocytes (**Figure 8H**).

### 3.6 Candidate drug prediction and molecular docking

**Figure 9:**
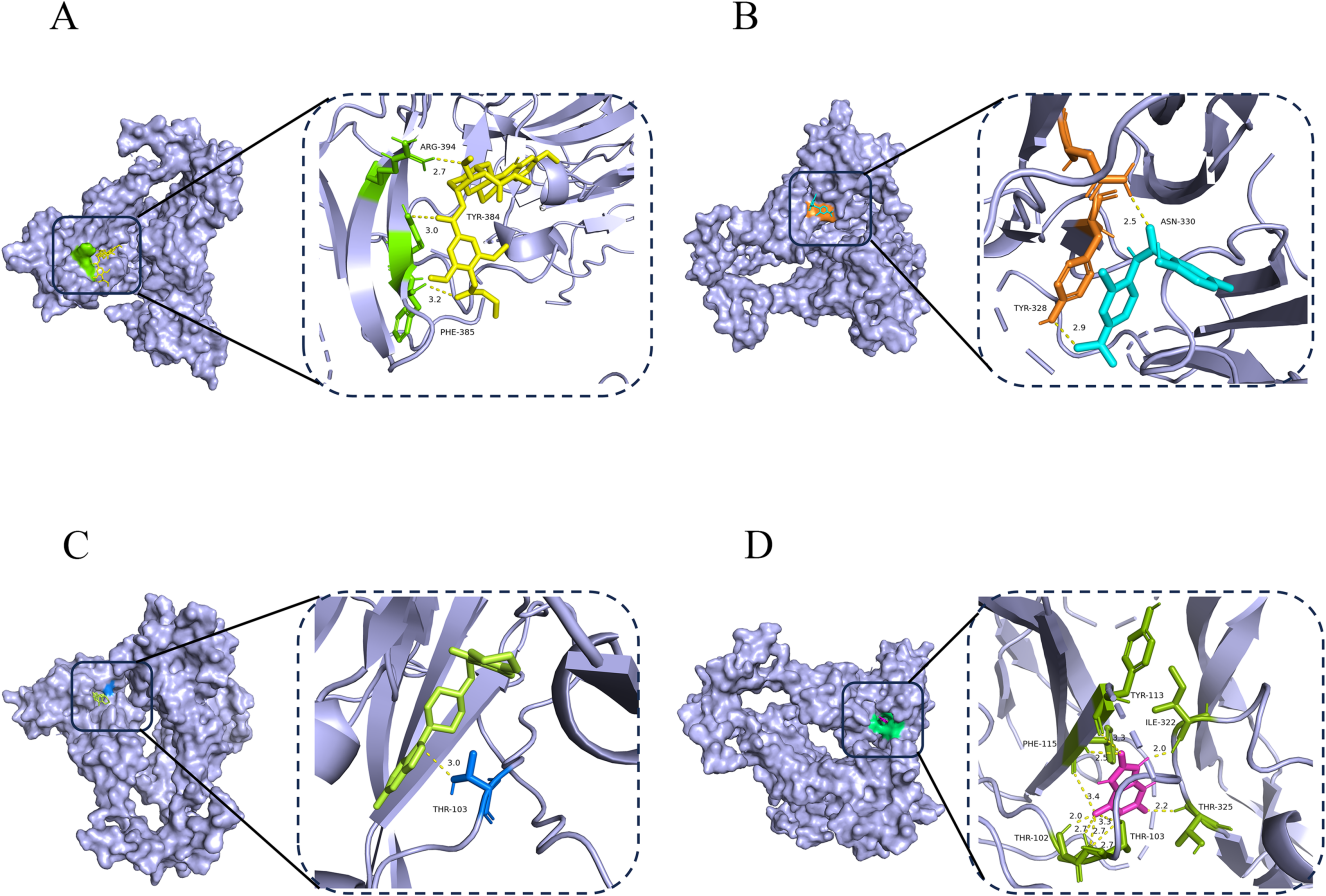
Potential therapeutic compounds and molecular docking analysis of INHBB. **(A–D)** Molecular docking technology was applied to the top four drugs (syrosingopine, niclosamide, risperidone and uric acid) ranked.

In the prognostic model, the INHBB gene was identified as the most significant biomarker affecting OS in COAD patients, as shown in **Supplementary Figure 2**. To explore potential therapeutic strategies targeting this key gene, we conducted a comprehensive drug screening using a gene-drug prediction database. By considering adjusted p-values, we successfully identified 22 compounds with potential therapeutic relevance in **Supplementary Table 3**. To further assess the binding affinity of these candidate drugs with their targets, we performed molecular docking for the top four compounds. Using AutoDock Vina v.1.5.7, we analyzed the binding sites and interactions of these candidates with the INHBB protein, calculating binding energies for each interaction. The docking results indicated that INHBB had the lowest binding energy with risperidone at -9.5 kcal/mol, suggesting a stable interaction. Visualize the docking results of four drugs with INHBB using Pymol software (**Figures 9A–D**). This analysis not only allows us to predict potentially effective drugs but also provides critical insights into drug-target interactions, informing drug development and optimization efforts.

### 3.7 Impact of INHBB knockdown on proliferation and survival

**Figure 10:**
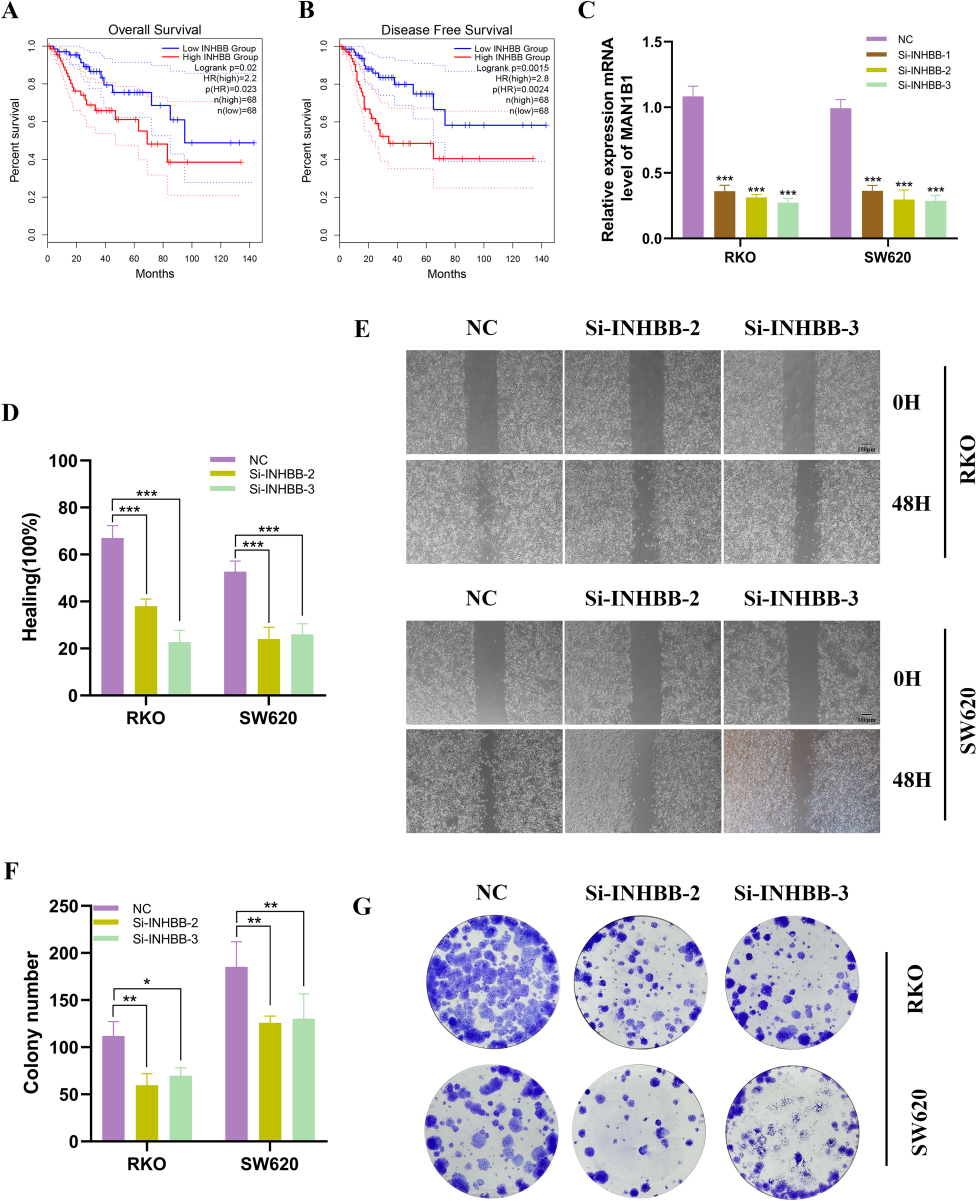
Analysis and experimental validation of INHBB expression. **(A, B)** Investigating INHBB’s impact on COAD OS and DFS in TCGA dataset. **(C)** INHBB was knocked down in RKO and SW620. **(D, E)** Scratch assays demonstrated that the migratory activity of cells with INHBB knockdown was significantly reduced. **(F, G)** Colony formation assays showed that the proliferative activity of cells with INHBB knockdown was significantly decreased. These symbols (* for P < 0.05, ** for P < 0.01, *** for P < 0.001) are used to denote the significance levels of statistical results.

Further analysis revealed that patients with high INHBB expression in COAD had worse OS and disease-free survival (DFS) (**Figures 10A, B**). In RKO and SW620 cell lines, INHBB knockdown resulted in significant changes in INHBB expression levels (**Figure 10C**). The verification of the low efficiency protein level is shown in the **Supplementary Figure 3**. In the wound healing assay, we observed a significant decrease in the proliferation activity of RKO and SW620 cells post-INHBB knockdown compared to control cells (**Figures 10D, E**). To further validate the impact of INHBB on the proliferative capacity of colorectal cancer cells, we conducted colony formation assays. The results showed that after INHBB gene knockdown, both cell lines exhibited reduced colony number and size (**Figure 10F, G**).

## 4 Discussion

Anoikis was first described in 1994 and refers to the programmed cell death of normally adherent cells when they remain in a suspended state for an extended period, leading to their demise due to “homelessness” (6). This form of apoptosis, characterized as a type of cellular “suicide,” is induced by the loss of contact with the ECM. Anoikis serves to eliminate cells that fail to attach properly to the matrix, thereby preventing their excessive proliferation (30). This mechanism is crucial for immune surveillance, helping to avoid the survival and dissemination of potential tumor or infected cells within the body. Tumor cells often evade immune attacks by suppressing anoikis; they may upregulate anti-apoptotic proteins (such as Bcl-2 family proteins) to inhibit apoptosis, thereby surviving even in the absence of matrix attachment, which provides a selective advantage for tumor progression. In the tumor microenvironment, anoikis may also impact the infiltration and functionality of immune cells (31). Damaged or unstable matrices can enable tumor cells to escape immune surveillance, adversely affecting the efficacy of immunotherapy. COAD cells frequently escape normal death signals through anoikis, thereby acquiring the ability to grow at distant sites (32). Understanding the mechanisms underlying anoikis is essential for elucidating how tumor cells survive during the metastatic process.

Due to the high heterogeneity of tumors, traditional molecular subtyping still exhibits significant variability in treatment outcomes for COAD patients. Precision therapy is crucial in the management of various solid tumors and represents a key direction for future cancer treatment. In this study, we leveraged COAD samples from TCGA database and GEO database to explore and identify a series of ARGs. Leveraging these genes, we established a prognostic model comprising nine key genes (NAT1, INHBB, FGF2, CD36, CCDC80, SPP1, MMP3, S100A11, and GZMB) (33, 34). This model stratified COAD patients into high-risk and low-risk groups based on risk scores, revealing significant disparities in survival outcomes, mutation patterns, immune cell infiltration, and chemotherapy responses between the two groups.

GO and KEGG enrichment analyses conducted on the differentially expressed genes across the risk categories indicated their participation in cellular responses to chemokines. This suggests these genes could have significant roles in inflammation and immune response mechanisms. KEGG pathway enrichment further suggests active inflammatory responses and intercellular signaling among the differentially expressed genes, which could significantly impact tumor progression and metastasis. Significant differences were observed in the expression levels of various immune cell populations, such as plasma cells, activated CD4 memory T cells, resting NK cells, monocytes, and M1 and M2 macrophages across the risk groups. The notable decrease in plasma cell expression within the high-risk group may suggest a compromised ability to produce antibodies, which could diminish the immune system’s capacity to combat tumor cells. Additionally, lower NK cell infiltration in the high-risk group may facilitate immune evasion by tumor cells, as NK cells are critical for directly killing tumor cells. M1 macrophages are typically associated with anti-tumor immune responses, while M2 macrophages may promote tumor development and metastasis, suggesting a potential strong immune evasion mechanism in the high-risk group. Notable differences in immune scores and tumor microenvironment scores reinforce the link between immune cell infiltration and tumor progression. Single-cell analysis uncovered unique expression patterns of different cell types and their associated genes within the COAD microenvironment, offering valuable insights into the biological mechanisms driving tumor behavior.

Elevated levels of crucial genes such as INHBB, CCDC80, and S100A11 could be significantly linked to tumor development, progression, and the modulation of the tumor microenvironment. INHBB is a member of the transforming growth factor-beta (TGF-β) superfamily, primarily encoded by the INHBB gene, and is part of the inhibin family, functioning alongside inhibin subunit beta A (INHBA) and inhibin subunit beta C (INHBC) to play important physiological roles (35). INHBB is involved in various biological processes, including cell proliferation and apoptosis, making it an important candidate in cancer research. Previous studies have shown that INHBB exhibits a complex dual role in different types of cancer. It not only promotes the proliferation and invasion of various cancers, such as clear cell renal carcinoma (36), hepatocellular carcinoma (37), endometrial carcinoma (38), and prostate cancer (39), but also, in some cases, suppresses the metastasis of nasopharyngeal carcinoma (40) and induces apoptosis. For example, INHBB expression is significantly elevated in endometrial carcinoma tissues and enhances cancer cell invasion by activating the SMAD2/3 and integrin β3 signaling pathways. INHBB promotes gastric cancer (GC) by reprogramming fibroblasts into cancer-associated fibroblasts (CAFs) and activating the NF-κB pathway, which enhances gastric cancer cell proliferation, migration, and invasion (41). However, research on the mechanisms of INHBB in colon cancer is relatively limited. Some studies suggest that increased INHBB expression may promote epithelial-mesenchymal transition (EMT) in cancer cells, thereby enhancing their metastatic potential (42). In our study, we observed a negative correlation between INHBB levels and OS in high-risk COAD patients. We also validated the expression level and functional role of INHBB in COAD, finding that knocking down INHBB significantly reduced cell proliferation, further supporting its potential as a prognostic biomarker (43). We also explored the therapeutic potential of INHBB and used molecular docking technology to identify risperidone as the compound with the lowest binding energy to INHBB. Risperidone may indirectly affect COAD cell proliferation by regulating dopamine and serotonin receptor signaling pathways. On the other hand, CCDC80 has been shown to reduce COAD cell proliferation by negatively regulating the ERK1/2 signaling pathway, thereby inhibiting tumor progression (44). SPP1 is overexpressed in COAD, significantly enhancing tumor cell invasion and metastasis, and is associated with poor prognosis (45). GZMB plays a key role in the immune system, and studies have shown that it participates in regulating cancer cell apoptosis during COAD immune responses, indicating its potential application in immunotherapy (46).

This study, however, does have certain limitations. Our analysis relies on transcriptomic data from public databases, which may introduce selection bias, as these datasets may not fully represent the diversity of all COAD patients. The predictive capability of this model requires further assessment and validation in independent, diverse patient cohorts through prospective studies.

## 5 Conclusions

In conclusion, our research, based on the features of ARGs, offers novel perspectives on the molecular subtyping and prognostic evaluation of COAD, as well as highlighting potential targets for future therapeutic interventions and drug development. The findings of this research hold promise for advancing the field of personalized treatment for COAD, offering patients more precise and effective therapeutic options.

## Data Availability

The datasets presented in this study can be found in online repositories. The names of the repository/repositories and accession number(s) can be found in the article/**Supplementary Material**.

## Supplementary material

Supplementary Figure 1 Exploration of Anoikis-Related Prognostic Genes. **(A)** Correlation network diagram of ARGs. **(B)** The histogram displays the gain or loss of CNV. **(C)** Copy loop diagram showing the position of ARGs on chromosomes. **(D)** The PPI network of ARGs. **(E)** The correlation network diagram shows the correlation between ARGs.

Supplementary Figure 2 The impact of expression levels of six genes on OS in COAD patients, as compared by ROC curves and survival analysis.

Supplementary Figure 3 The WB blot of INHBB after siRNA transfection to indicate its knockdown efficiency.
