# Supplementary figures and images for: Combining single-cell analysis and molecular docking techniques to construct a prognostic model for colon adenocarcinoma and uncovering inhibin subunit βb as a novel therapeutic target
Source: Front Immunol. 2025 Jan 9;15:1524560. doi: 10.3389/fimmu.2024.1524560 (PMC11754261; doi:10.3389/fimmu.2024.1524560)

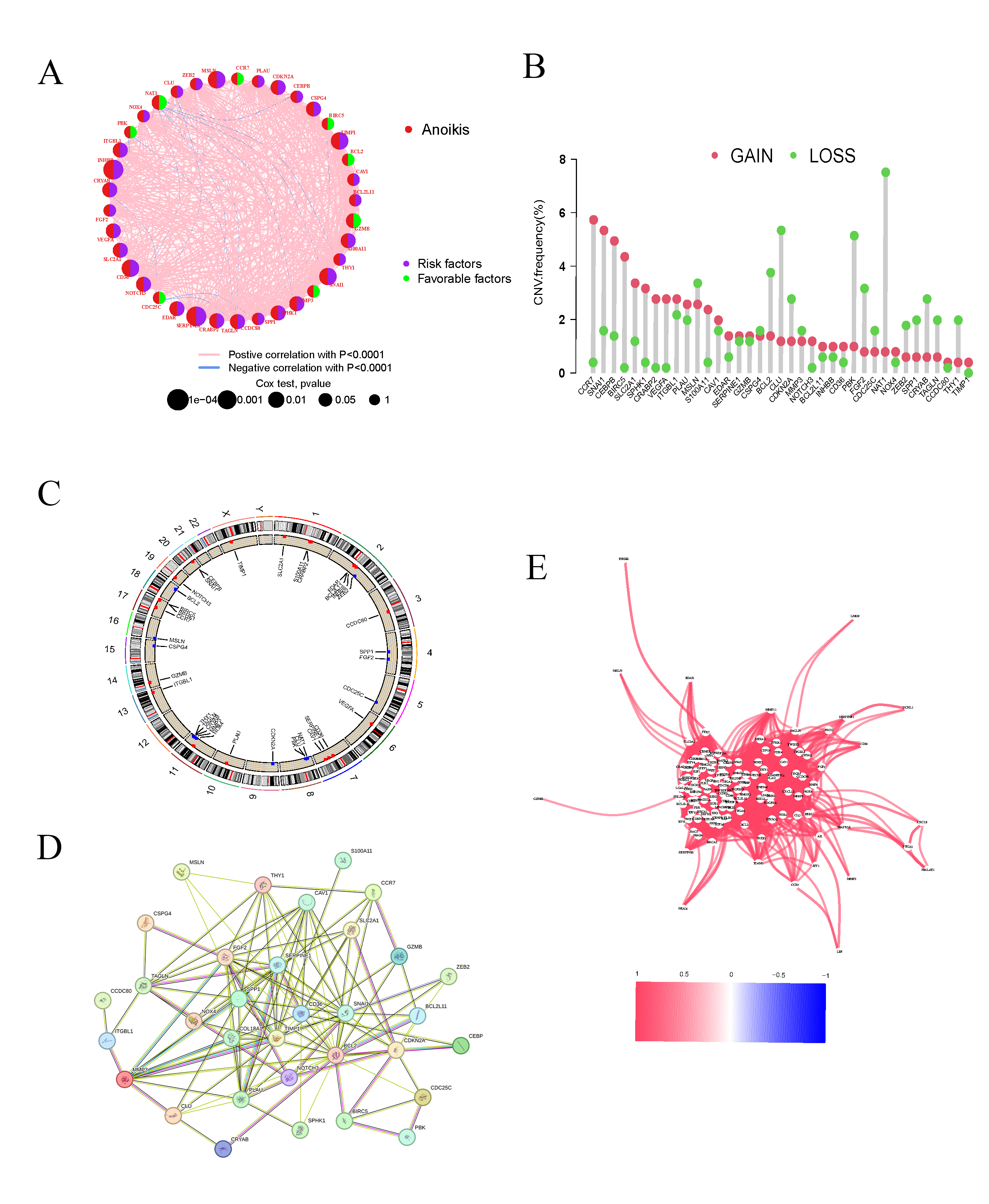

Supplement: Supplementary Figure 1 — Exploration of Anoikis-Related Prognostic Genes. (A) Correlation network diagram of ARGs. (B) The histogram displays the gain or loss of CNV. (C) Copy loop diagram showing the position of ARGs on chromosomes. (D) The PPI network of ARGs. (E) The correlation network diagram shows the correlation between ARGs. [file Image1.tiff]

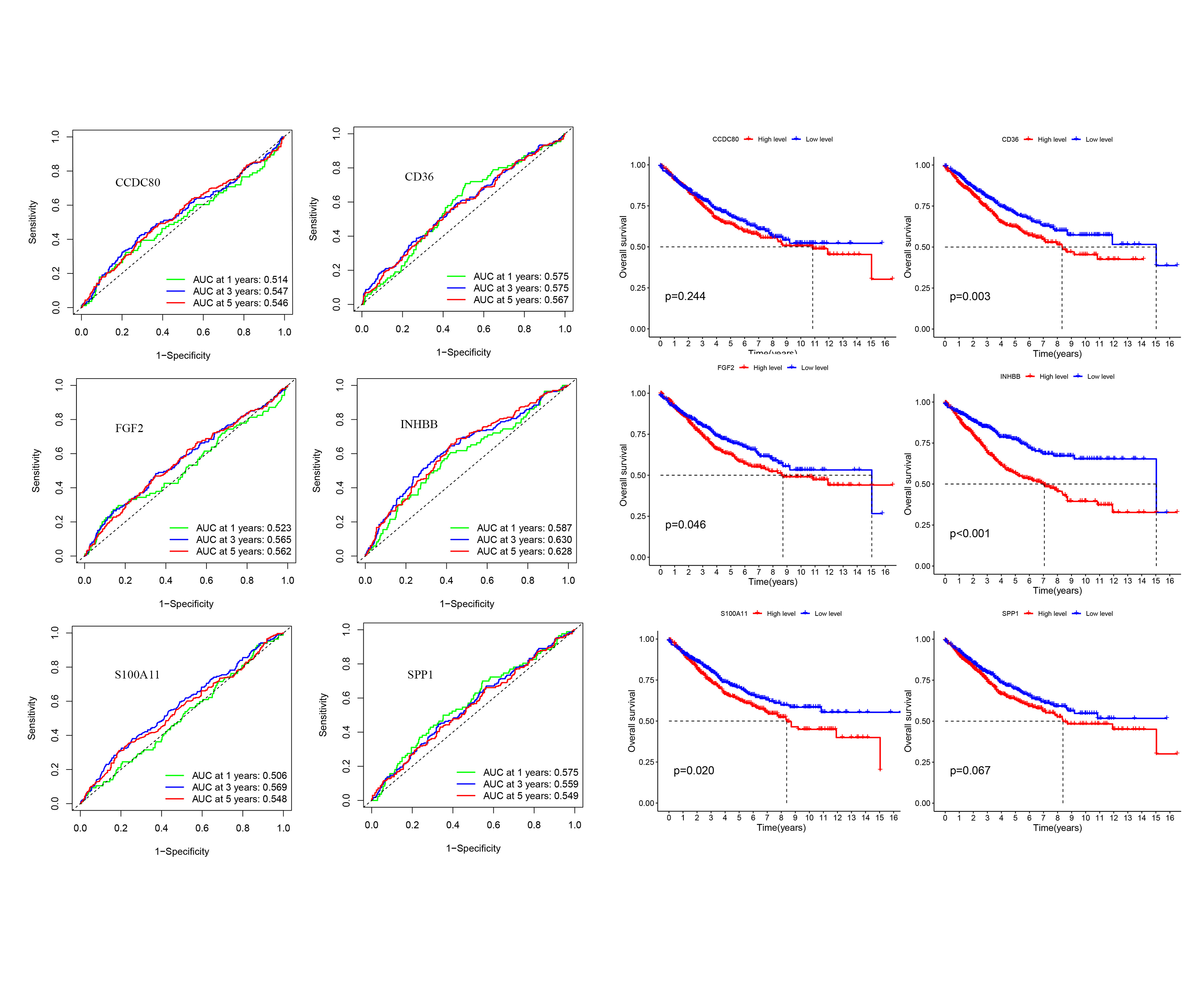

Supplement: Supplementary Figure 2 — The impact of expression levels of six genes on OS in COAD patients, as compared by ROC curves and survival analysis. [file Image2.tiff]

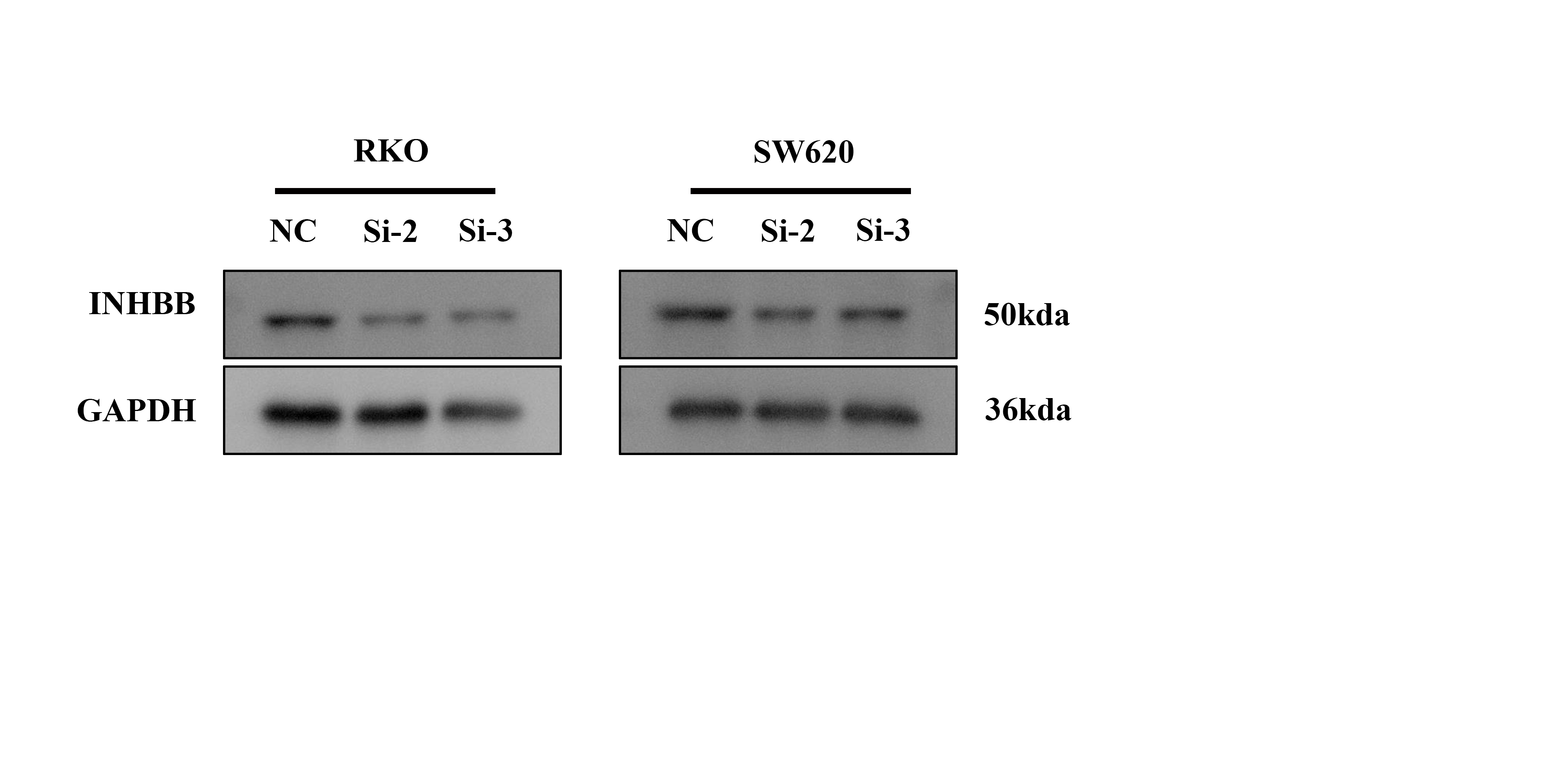

Supplement: Supplementary Figure 3 — The WB blot of INHBB after siRNA transfection to indicate its knockdown efficiency. [file Image3.tif]
