# Supplementary material for: Combining single-cell analysis and molecular docking techniques to construct a prognostic model for colon adenocarcinoma and uncovering inhibin subunit βb as a novel therapeutic target
Source: Front Immunol. 2025 Jan 9;15:1524560. doi: 10.3389/fimmu.2024.1524560 (PMC11754261; doi:10.3389/fimmu.2024.1524560)
Supplement: Supplementary file 4 [file Table1.docx]

| **Oligonucleotides** | **Nucleotide sequence (5'-3')** |
| --- | --- |
| **siRNA** |  |
| Scramble control | GCUUCGCGCCGUAGUCUUA |
| Si-INHBB-1 | GCTGTACTTCGATGATGAGTA |
| Si-INHBB-2 | CCTATACTTCTTCATCTCCAA |
|  |  |
| **Primer** |  |
| GAPDH | GGCCTCCAAGGAGTAAGACC (forward) |
|  | AGGGGAGATTCAGTGTGGTG (reverse) |
| INHBB | GCGAGAACCCTCAACTGACA (forward) |
|  | ACCGCATCCATTTGCTGGTA (reverse) |
|  |  |

**Oligonucleotides used in research**
