# Supplementary material for: Combining single-cell analysis and molecular docking techniques to construct a prognostic model for colon adenocarcinoma and uncovering inhibin subunit βb as a novel therapeutic target
Source: Front Immunol. 2025 Jan 9;15:1524560. doi: 10.3389/fimmu.2024.1524560 (PMC11754261; doi:10.3389/fimmu.2024.1524560)
Supplement: Supplementary file 5 [file Table2.docx]

Marker genes used for identifying and distinguishing different cell types

| \| gene \| p_val \| avg_log2FC \| p_val_adj \| cluster \| \| --- \| --- \| --- \| --- \| --- \| |
| --- | --- | --- | --- | --- | --- |
| \| IL7R \| 0 \| 3.25362244 \| 0 \| 0 \| \| --- \| --- \| --- \| --- \| --- \| \| LTB \| 0 \| 2.470251469 \| 0 \| 0 \| \| KLRB1 \| 0 \| 2.391357351 \| 0 \| 0 \| \| TRBC1 \| 0 \| 2.38726136 \| 0 \| 0 \| \| TRAC \| 0 \| 2.274588423 \| 0 \| 0 \| \| CD2 \| 0 \| 2.239092683 \| 0 \| 0 \| \| TRBC2 \| 0 \| 2.190839415 \| 0 \| 0 \| \| CD3D \| 0 \| 2.158223425 \| 0 \| 0 \| \| SPOCK2 \| 0 \| 2.092724191 \| 0 \| 0 \| \| CD3E \| 0 \| 2.037453278 \| 0 \| 0 \| \| FYB1 \| 0 \| 1.980510093 \| 0 \| 0 \| \| CD3G \| 0 \| 1.889559682 \| 0 \| 0 \| \| RORA \| 0 \| 1.880858066 \| 0 \| 0 \| \| TNFAIP3 \| 0 \| 1.86525443 \| 0 \| 0 \| \| ICOS \| 0 \| 1.85800115 \| 0 \| 0 \| \| CTLA4 \| 0 \| 1.848598366 \| 0 \| 0 \| \| CREM \| 0 \| 1.788679806 \| 0 \| 0 \| \| CXCR4 \| 0 \| 1.784633338 \| 0 \| 0 \| \| S100A4 \| 0 \| 1.765557602 \| 0 \| 0 \| \| SARAF \| 0 \| 1.738369657 \| 0 \| 0 \| \| CCR6 \| 0 \| 1.716015311 \| 0 \| 0 \| \| AC058791.1 \| 0 \| 1.715695047 \| 0 \| 0 \| \| PTPRC \| 0 \| 1.710784054 \| 0 \| 0 \| \| BCL11B \| 0 \| 1.703633596 \| 0 \| 0 \| \| RGCC \| 0 \| 1.615813673 \| 0 \| 0 \| \| TSC22D3 \| 0 \| 1.60926882 \| 0 \| 0 \| \| BTG1 \| 0 \| 1.584181268 \| 0 \| 0 \| \| CD7 \| 0 \| 1.57351844 \| 0 \| 0 \| \| CD69 \| 0 \| 1.563341648 \| 0 \| 0 \| \| TNFRSF18 \| 0 \| 1.560585032 \| 0 \| 0 \| \| ARHGDIB \| 0 \| 1.514077064 \| 0 \| 0 \| \| CYTIP \| 0 \| 1.514011466 \| 0 \| 0 \| \| TIGIT \| 0 \| 1.504829797 \| 0 \| 0 \| \| CNOT6L \| 0 \| 1.480876907 \| 0 \| 0 \| \| STK17B \| 0 \| 1.479196073 \| 0 \| 0 \| \| ETS1 \| 0 \| 1.460689254 \| 0 \| 0 \| \| RGS1 \| 0 \| 1.456057163 \| 0 \| 0 \| \| DNAJA1 \| 0 \| 1.45275246 \| 0 \| 0 \| \| FYN \| 0 \| 1.445359462 \| 0 \| 0 \| \| PBXIP1 \| 0 \| 1.442308378 \| 0 \| 0 \| \| SRGN \| 0 \| 1.441320858 \| 0 \| 0 \| \| PIK3IP1 \| 0 \| 1.413868313 \| 0 \| 0 \| \| ARL4C \| 0 \| 1.413613574 \| 0 \| 0 \| \| JUNB \| 0 \| 1.413225016 \| 0 \| 0 \| \| CD52 \| 0 \| 1.412993718 \| 0 \| 0 \| \| CLEC2D \| 0 \| 1.407982627 \| 0 \| 0 \| \| ARID5B \| 0 \| 1.40370652 \| 0 \| 0 \| \| LCK \| 0 \| 1.398440152 \| 0 \| 0 \| \| GMFG \| 0 \| 1.397134442 \| 0 \| 0 \| \| GIMAP7 \| 0 \| 1.39549125 \| 0 \| 0 \| \| CD48 \| 0 \| 1.345763905 \| 0 \| 0 \| \| SMAP2 \| 0 \| 1.322494458 \| 0 \| 0 \| \| ITM2A \| 0 \| 1.315917966 \| 0 \| 0 \| \| GPR171 \| 0 \| 1.314226879 \| 0 \| 0 \| \| RASGRP1 \| 0 \| 1.310948092 \| 0 \| 0 \| \| GPR183 \| 0 \| 1.298868375 \| 0 \| 0 \| \| DNAJB1 \| 0 \| 1.296692786 \| 0 \| 0 \| \| ZC3HAV1 \| 0 \| 1.296339958 \| 0 \| 0 \| \| FXYD5 \| 0 \| 1.265960697 \| 0 \| 0 \| \| CD28 \| 0 \| 1.231053186 \| 0 \| 0 \| \| SKAP1 \| 0 \| 1.210265126 \| 0 \| 0 \| \| LINC00513 \| 0 \| 1.204463285 \| 0 \| 0 \| \| ANKRD12 \| 0 \| 1.203608874 \| 0 \| 0 \| \| CD247 \| 0 \| 1.19028496 \| 0 \| 0 \| \| RPS27 \| 0 \| 1.188097126 \| 0 \| 0 \| \| EIF1 \| 0 \| 1.172030517 \| 0 \| 0 \| \| ITK \| 0 \| 1.16911823 \| 0 \| 0 \| \| RPLP1 \| 0 \| 1.15318847 \| 0 \| 0 \| \| IKZF1 \| 0 \| 1.147052248 \| 0 \| 0 \| \| RPS27A \| 0 \| 1.140555941 \| 0 \| 0 \| \| SAMSN1 \| 0 \| 1.134604621 \| 0 \| 0 \| \| AC026979.2 \| 0 \| 1.13227655 \| 0 \| 0 \| \| DDX5 \| 0 \| 1.130350101 \| 0 \| 0 \| \| RPS4Y1 \| 0 \| 1.127581791 \| 0 \| 0 \| \| CD6 \| 0 \| 1.124517157 \| 0 \| 0 \| \| RUNX3 \| 0 \| 1.123257753 \| 0 \| 0 \| \| SLA \| 0 \| 1.113110859 \| 0 \| 0 \| \| RAC2 \| 0 \| 1.102441999 \| 0 \| 0 \| \| ZNF331 \| 0 \| 1.101894502 \| 0 \| 0 \| \| RPS3A \| 0 \| 1.088086235 \| 0 \| 0 \| \| RPS15A \| 0 \| 1.086857373 \| 0 \| 0 \| \| APBB1IP \| 0 \| 1.083946406 \| 0 \| 0 \| \| HSPA8 \| 0 \| 1.081578594 \| 0 \| 0 \| \| CELF2 \| 0 \| 1.080715362 \| 0 \| 0 \| \| ZFP36L2 \| 0 \| 1.07932918 \| 0 \| 0 \| \| TMSB4X \| 0 \| 1.076245587 \| 0 \| 0 \| \| IRF1 \| 0 \| 1.067567956 \| 0 \| 0 \| \| RPL30 \| 0 \| 1.066124538 \| 0 \| 0 \| \| LAT \| 0 \| 1.055539212 \| 0 \| 0 \| \| TNFAIP8 \| 0 \| 1.04757802 \| 0 \| 0 \| \| RHOH \| 0 \| 1.044633094 \| 0 \| 0 \| \| RPS12 \| 0 \| 1.04271773 \| 0 \| 0 \| \| JUND \| 0 \| 1.038973752 \| 0 \| 0 \| \| TPT1 \| 0 \| 1.019491127 \| 0 \| 0 \| \| P2RY10 \| 0 \| 1.005288304 \| 0 \| 0 \| \| CEBPD \| 0 \| -1.006627298 \| 0 \| 0 \| \| CHCHD10 \| 0 \| -1.028093042 \| 0 \| 0 \| \| PTPRF \| 0 \| -1.036882359 \| 0 \| 0 \| \| JUP \| 0 \| -1.052000346 \| 0 \| 0 \| \| RHOB \| 0 \| -1.057369005 \| 0 \| 0 \| \| ATP8B1 \| 0 \| -1.062521116 \| 0 \| 0 \| \| FKBP2 \| 0 \| -1.064927766 \| 0 \| 0 \| \| SPTBN1 \| 0 \| -1.076483081 \| 0 \| 0 \| \| XBP1 \| 0 \| -1.081210802 \| 0 \| 0 \| \| SERINC2 \| 0 \| -1.091041531 \| 0 \| 0 \| \| MYH14 \| 0 \| -1.109919622 \| 0 \| 0 \| \| DSC2 \| 0 \| -1.113843918 \| 0 \| 0 \| \| SPINT1 \| 0 \| -1.114363498 \| 0 \| 0 \| \| CTNND1 \| 0 \| -1.117077492 \| 0 \| 0 \| \| FAT1 \| 0 \| -1.128155953 \| 0 \| 0 \| \| S100A16 \| 0 \| -1.134232176 \| 0 \| 0 \| \| ALDH2 \| 0 \| -1.135720121 \| 0 \| 0 \| \| TCF7L2 \| 0 \| -1.138819277 \| 0 \| 0 \| \| MGLL \| 0 \| -1.139493039 \| 0 \| 0 \| \| PRSS8 \| 0 \| -1.1461562 \| 0 \| 0 \| \| DSTN \| 0 \| -1.146500009 \| 0 \| 0 \| \| TSPAN3 \| 0 \| -1.150770281 \| 0 \| 0 \| \| TST \| 0 \| -1.163333804 \| 0 \| 0 \| \| CES2 \| 0 \| -1.169193989 \| 0 \| 0 \| \| CDH1 \| 0 \| -1.173886286 \| 0 \| 0 \| \| GOLIM4 \| 0 \| -1.175742518 \| 0 \| 0 \| \| RBM47 \| 0 \| -1.187196627 \| 0 \| 0 \| \| CTSZ \| 0 \| -1.187275316 \| 0 \| 0 \| \| ID1 \| 0 \| -1.202061469 \| 0 \| 0 \| \| ITGA6 \| 0 \| -1.210206966 \| 0 \| 0 \| \| TMEM176A \| 0 \| -1.23255166 \| 0 \| 0 \| \| GOLM1 \| 0 \| -1.236604794 \| 0 \| 0 \| \| ANXA2 \| 0 \| -1.276353119 \| 0 \| 0 \| \| TSC22D1 \| 0 \| -1.277736148 \| 0 \| 0 \| \| ETS2 \| 0 \| -1.286629544 \| 0 \| 0 \| \| GLUL \| 0 \| -1.298680776 \| 0 \| 0 \| \| DSG2 \| 0 \| -1.30484916 \| 0 \| 0 \| \| SLC12A2 \| 0 \| -1.310364746 \| 0 \| 0 \| \| ATP1B1 \| 0 \| -1.310395581 \| 0 \| 0 \| \| YBX3 \| 0 \| -1.362942302 \| 0 \| 0 \| \| APP \| 0 \| -1.373906213 \| 0 \| 0 \| \| CAMK2N1 \| 0 \| -1.374949053 \| 0 \| 0 \| \| PDLIM1 \| 0 \| -1.377427845 \| 0 \| 0 \| \| CDH17 \| 0 \| -1.390416084 \| 0 \| 0 \| \| ST14 \| 0 \| -1.417926532 \| 0 \| 0 \| \| FCGRT \| 0 \| -1.422496604 \| 0 \| 0 \| \| MISP \| 0 \| -1.423247397 \| 0 \| 0 \| \| S100P \| 0 \| -1.439802938 \| 0 \| 0 \| \| SFN \| 0 \| -1.440487223 \| 0 \| 0 \| \| TMEM176B \| 0 \| -1.449317174 \| 0 \| 0 \| \| KLF5 \| 0 \| -1.469639979 \| 0 \| 0 \| \| GPRC5A \| 0 \| -1.487104482 \| 0 \| 0 \| \| PSAP \| 0 \| -1.491633105 \| 0 \| 0 \| \| SLC44A4 \| 0 \| -1.51345252 \| 0 \| 0 \| \| VEGFA \| 0 \| -1.523467264 \| 0 \| 0 \| \| KLF4 \| 0 \| -1.525445051 \| 0 \| 0 \| \| LGALS3BP \| 0 \| -1.536722095 \| 0 \| 0 \| \| S100A14 \| 0 \| -1.541664234 \| 0 \| 0 \| \| DST \| 0 \| -1.553361959 \| 0 \| 0 \| \| TPM1 \| 0 \| -1.57393712 \| 0 \| 0 \| \| TIMP1 \| 0 \| -1.663209856 \| 0 \| 0 \| \| DSP \| 0 \| -1.674609871 \| 0 \| 0 \| \| RRBP1 \| 0 \| -1.686114338 \| 0 \| 0 \| \| GRN \| 0 \| -1.691483982 \| 0 \| 0 \| \| GSTP1 \| 0 \| -1.700505159 \| 0 \| 0 \| \| IER3 \| 0 \| -1.730956531 \| 0 \| 0 \| \| CDC42EP5 \| 0 \| -1.748396391 \| 0 \| 0 \| \| CD63 \| 0 \| -1.752599924 \| 0 \| 0 \| \| GPX2 \| 0 \| -1.769438912 \| 0 \| 0 \| \| MUC13 \| 0 \| -1.802689563 \| 0 \| 0 \| \| LGALS3 \| 0 \| -1.829009726 \| 0 \| 0 \| \| IFITM3 \| 0 \| -1.839181579 \| 0 \| 0 \| \| TMEM54 \| 0 \| -1.881738076 \| 0 \| 0 \| \| SMIM22 \| 0 \| -1.882810662 \| 0 \| 0 \| \| HES1 \| 0 \| -1.885939238 \| 0 \| 0 \| \| ITM2C \| 0 \| -1.919315158 \| 0 \| 0 \| \| SOX4 \| 0 \| -1.929678951 \| 0 \| 0 \| \| FAM3D \| 0 \| -1.946868208 \| 0 \| 0 \| \| CD24 \| 0 \| -1.972110345 \| 0 \| 0 \| \| GSN \| 0 \| -2.023977586 \| 0 \| 0 \| \| CD9 \| 0 \| -2.079086276 \| 0 \| 0 \| \| S100A6 \| 0 \| -2.135693328 \| 0 \| 0 \| \| MARCKS \| 0 \| -2.166817716 \| 0 \| 0 \| \| MUC12 \| 0 \| -2.174512835 \| 0 \| 0 \| \| EPCAM \| 0 \| -2.345687984 \| 0 \| 0 \| \| CKB \| 0 \| -2.348624722 \| 0 \| 0 \| \| TSPAN1 \| 0 \| -2.390718107 \| 0 \| 0 \| \| CLDN3 \| 0 \| -2.392242256 \| 0 \| 0 \| \| LCN2 \| 0 \| -2.540066942 \| 0 \| 0 \| \| CLDN4 \| 0 \| -2.579440303 \| 0 \| 0 \| \| SELENOP \| 0 \| -2.581241322 \| 0 \| 0 \| \| TSPAN8 \| 0 \| -2.625942557 \| 0 \| 0 \| \| ELF3 \| 0 \| -2.64074175 \| 0 \| 0 \| \| FXYD3 \| 0 \| -2.781819305 \| 0 \| 0 \| \| AGR2 \| 0 \| -2.913289327 \| 0 \| 0 \| \| KRT18 \| 0 \| -3.086577682 \| 0 \| 0 \| \| KRT19 \| 0 \| -3.136660431 \| 0 \| 0 \| \| LGALS4 \| 0 \| -3.144248278 \| 0 \| 0 \| \| KRT8 \| 0 \| -3.199663529 \| 0 \| 0 \| \| LYZ \| 0 \| -3.258221216 \| 0 \| 0 \| \| IFI27 \| 0 \| -3.273190966 \| 0 \| 0 \| \| FABP1 \| 0 \| -3.675878703 \| 0 \| 0 \| \| TFF3 \| 0 \| -3.92985803 \| 0 \| 0 \| \| PIGR \| 0 \| -4.378053293 \| 0 \| 0 \| \| PHGR1 \| 0 \| -4.861784638 \| 0 \| 0 \| \| ANXA1 \| 2.2647E-307 \| 1.566708421 \| 4.9432E-303 \| 0 \| \| FNBP1 \| 1.079E-297 \| 1.001090828 \| 2.3551E-293 \| 0 \| \| TXNIP \| 1.2485E-296 \| 1.022982816 \| 2.7251E-292 \| 0 \| \| SOCS1 \| 2.8969E-293 \| 1.010371731 \| 6.323E-289 \| 0 \| \| JCHAIN \| 2.6727E-292 \| -5.239966478 \| 5.8337E-288 \| 0 \| \| EMB \| 1.5667E-290 \| 1.004802356 \| 3.4197E-286 \| 0 \| \| STK4 \| 1.3024E-272 \| 1.046515804 \| 2.8428E-268 \| 0 \| \| SSR4 \| 2.9193E-269 \| -1.387499461 \| 6.3719E-265 \| 0 \| \| FAM107B \| 5.6717E-268 \| 1.09348671 \| 1.238E-263 \| 0 \| \| PPP1R2 \| 3.8185E-267 \| 1.199229296 \| 8.3346E-263 \| 0 \| \| TBC1D4 \| 5.3623E-263 \| 1.219315232 \| 1.1704E-258 \| 0 \| \| TAGAP \| 2.4828E-254 \| 1.03132739 \| 5.4192E-250 \| 0 \| \| GLIPR1 \| 7.0221E-244 \| 1.054255659 \| 1.5327E-239 \| 0 \| \| HLA-DRA \| 5.689E-239 \| -3.33084334 \| 1.2417E-234 \| 0 \| \| SLFN5 \| 2.6733E-232 \| 1.050257327 \| 5.8351E-228 \| 0 \| \| DUSP4 \| 5.5242E-224 \| 1.254950267 \| 1.2058E-219 \| 0 \| \| IGHA1 \| 9.0501E-214 \| -4.94780872 \| 1.9754E-209 \| 0 \| \| FKBP5 \| 1.8914E-213 \| 1.104896348 \| 4.1283E-209 \| 0 \| \| LGALS1 \| 3.2143E-186 \| -1.06400716 \| 7.0159E-182 \| 0 \| \| CDKN1B \| 1.7826E-184 \| 1.041089516 \| 3.891E-180 \| 0 \| \| CACYBP \| 1.4704E-177 \| 1.200358261 \| 3.2094E-173 \| 0 \| \| S100A10 \| 3.1761E-148 \| -1.035003668 \| 6.9324E-144 \| 0 \| \| CD74 \| 8.9681E-128 \| -1.853504149 \| 1.9575E-123 \| 0 \| \| HLA-DRB1 \| 2.2638E-125 \| -1.753395981 \| 4.9412E-121 \| 0 \| \| FTL \| 9.9672E-116 \| -1.152276736 \| 2.1755E-111 \| 0 \| \| PLAC8 \| 4.1959E-110 \| -1.188912702 \| 9.1584E-106 \| 0 \| \| HLA-DPA1 \| 4.23169E-83 \| -1.738627535 \| 9.2365E-79 \| 0 \| \| HLA-DPB1 \| 6.77195E-66 \| -1.709559802 \| 1.47811E-61 \| 0 \| \| IGLC3 \| 9.47313E-61 \| -5.106578777 \| 2.0677E-56 \| 0 \| \| IGLC2 \| 5.28872E-51 \| -5.438822774 \| 1.15437E-46 \| 0 \| \| IGKC \| 9.48695E-50 \| -5.396101873 \| 2.07072E-45 \| 0 \| \| CCL5 \| 0 \| 4.336759242 \| 0 \| 1 \| \| GNLY \| 0 \| 4.108466837 \| 0 \| 1 \| \| GZMA \| 0 \| 3.9347918 \| 0 \| 1 \| \| CCL4 \| 0 \| 3.883184444 \| 0 \| 1 \| \| NKG7 \| 0 \| 3.816865599 \| 0 \| 1 \| \| GZMK \| 0 \| 3.185470975 \| 0 \| 1 \| \| GZMB \| 0 \| 2.951055388 \| 0 \| 1 \| \| IFNG \| 0 \| 2.875951664 \| 0 \| 1 \| \| CST7 \| 0 \| 2.596097416 \| 0 \| 1 \| \| GZMH \| 0 \| 2.592741023 \| 0 \| 1 \| \| CD8A \| 0 \| 2.476977947 \| 0 \| 1 \| \| HCST \| 0 \| 2.245816702 \| 0 \| 1 \| \| KLRD1 \| 0 \| 2.180619837 \| 0 \| 1 \| \| CD7 \| 0 \| 2.06072854 \| 0 \| 1 \| \| HOPX \| 0 \| 2.044373248 \| 0 \| 1 \| \| CD3D \| 0 \| 2.037960154 \| 0 \| 1 \| \| DUSP2 \| 0 \| 2.021995297 \| 0 \| 1 \| \| CTSW \| 0 \| 2.001934042 \| 0 \| 1 \| \| PRF1 \| 0 \| 1.99717002 \| 0 \| 1 \| \| CD52 \| 0 \| 1.936624761 \| 0 \| 1 \| \| CD3G \| 0 \| 1.903496501 \| 0 \| 1 \| \| CRTAM \| 0 \| 1.898370967 \| 0 \| 1 \| \| CLEC2B \| 0 \| 1.896060386 \| 0 \| 1 \| \| GZMM \| 0 \| 1.858690006 \| 0 \| 1 \| \| CD2 \| 0 \| 1.852553016 \| 0 \| 1 \| \| PTPN22 \| 0 \| 1.849805691 \| 0 \| 1 \| \| CD3E \| 0 \| 1.777005287 \| 0 \| 1 \| \| KLRB1 \| 0 \| 1.767032753 \| 0 \| 1 \| \| DUSP4 \| 0 \| 1.746599262 \| 0 \| 1 \| \| PTPRC \| 0 \| 1.730042033 \| 0 \| 1 \| \| CXCR4 \| 0 \| 1.701587479 \| 0 \| 1 \| \| TRBC2 \| 0 \| 1.683149498 \| 0 \| 1 \| \| CD69 \| 0 \| 1.679380368 \| 0 \| 1 \| \| TRGC2 \| 0 \| 1.666016799 \| 0 \| 1 \| \| TRBC1 \| 0 \| 1.66101346 \| 0 \| 1 \| \| APOBEC3G \| 0 \| 1.648267705 \| 0 \| 1 \| \| ID2 \| 0 \| 1.625253526 \| 0 \| 1 \| \| PIK3R1 \| 0 \| 1.615591156 \| 0 \| 1 \| \| SLA \| 0 \| 1.613687438 \| 0 \| 1 \| \| RARRES3 \| 0 \| 1.610702228 \| 0 \| 1 \| \| TRAC \| 0 \| 1.606696389 \| 0 \| 1 \| \| CD247 \| 0 \| 1.606500912 \| 0 \| 1 \| \| LINC01871 \| 0 \| 1.553496675 \| 0 \| 1 \| \| RUNX3 \| 0 \| 1.546071727 \| 0 \| 1 \| \| GPR65 \| 0 \| 1.535481941 \| 0 \| 1 \| \| RGCC \| 0 \| 1.527059991 \| 0 \| 1 \| \| GPR171 \| 0 \| 1.503241336 \| 0 \| 1 \| \| TNFAIP3 \| 0 \| 1.472256175 \| 0 \| 1 \| \| FYN \| 0 \| 1.458730817 \| 0 \| 1 \| \| LCP1 \| 0 \| 1.456960538 \| 0 \| 1 \| \| CLEC2D \| 0 \| 1.453444125 \| 0 \| 1 \| \| CREM \| 0 \| 1.450384319 \| 0 \| 1 \| \| ANXA1 \| 0 \| 1.44827449 \| 0 \| 1 \| \| IL2RB \| 0 \| 1.429464912 \| 0 \| 1 \| \| ITGA1 \| 0 \| 1.394461455 \| 0 \| 1 \| \| LAG3 \| 0 \| 1.377223264 \| 0 \| 1 \| \| TMSB4X \| 0 \| 1.371312556 \| 0 \| 1 \| \| CNOT6L \| 0 \| 1.3683346 \| 0 \| 1 \| \| PIP4K2A \| 0 \| 1.332326087 \| 0 \| 1 \| \| LCK \| 0 \| 1.312514707 \| 0 \| 1 \| \| ARL4C \| 0 \| 1.298913334 \| 0 \| 1 \| \| REL \| 0 \| 1.292791604 \| 0 \| 1 \| \| GIMAP7 \| 0 \| 1.291844581 \| 0 \| 1 \| \| DNAJA1 \| 0 \| 1.291501731 \| 0 \| 1 \| \| IKZF3 \| 0 \| 1.289671525 \| 0 \| 1 \| \| RGS1 \| 0 \| 1.288207174 \| 0 \| 1 \| \| NR4A2 \| 0 \| 1.266874579 \| 0 \| 1 \| \| EVL \| 0 \| 1.263645212 \| 0 \| 1 \| \| ITM2A \| 0 \| 1.251993361 \| 0 \| 1 \| \| STK17A \| 0 \| 1.250100694 \| 0 \| 1 \| \| CORO1A \| 0 \| 1.249863957 \| 0 \| 1 \| \| RAC2 \| 0 \| 1.245363528 \| 0 \| 1 \| \| ALOX5AP \| 0 \| 1.244201257 \| 0 \| 1 \| \| PARP8 \| 0 \| 1.237818348 \| 0 \| 1 \| \| ARHGDIB \| 0 \| 1.220930317 \| 0 \| 1 \| \| CD48 \| 0 \| 1.219995902 \| 0 \| 1 \| \| TIGIT \| 0 \| 1.217673865 \| 0 \| 1 \| \| MSN \| 0 \| 1.212150238 \| 0 \| 1 \| \| STK17B \| 0 \| 1.210405547 \| 0 \| 1 \| \| AKNA \| 0 \| 1.205013301 \| 0 \| 1 \| \| CYTIP \| 0 \| 1.199871948 \| 0 \| 1 \| \| EMB \| 0 \| 1.186807383 \| 0 \| 1 \| \| APBB1IP \| 0 \| 1.16796111 \| 0 \| 1 \| \| FYB1 \| 0 \| 1.163606815 \| 0 \| 1 \| \| DOK2 \| 0 \| 1.157304044 \| 0 \| 1 \| \| BTG1 \| 0 \| 1.148574125 \| 0 \| 1 \| \| ZNF331 \| 0 \| 1.148239039 \| 0 \| 1 \| \| SRGN \| 0 \| 1.147661529 \| 0 \| 1 \| \| SYTL3 \| 0 \| 1.142390148 \| 0 \| 1 \| \| ETS1 \| 0 \| 1.141298952 \| 0 \| 1 \| \| ITGAL \| 0 \| 1.135975603 \| 0 \| 1 \| \| TRAT1 \| 0 \| 1.13424104 \| 0 \| 1 \| \| SKAP1 \| 0 \| 1.134174742 \| 0 \| 1 \| \| S100A4 \| 0 \| 1.099661728 \| 0 \| 1 \| \| TSC22D3 \| 0 \| 1.088737864 \| 0 \| 1 \| \| 7-Sep \| 0 \| 1.085163525 \| 0 \| 1 \| \| SH3BGRL3 \| 0 \| 1.084828971 \| 0 \| 1 \| \| RASGRP1 \| 0 \| 1.069797876 \| 0 \| 1 \| \| CD96 \| 0 \| 1.059715494 \| 0 \| 1 \| \| COTL1 \| 0 \| 1.054724666 \| 0 \| 1 \| \| ZFP36L2 \| 0 \| 1.040799367 \| 0 \| 1 \| \| CD53 \| 0 \| 1.026358502 \| 0 \| 1 \| \| ALDH2 \| 0 \| -1.07145284 \| 0 \| 1 \| \| RBM47 \| 0 \| -1.108587846 \| 0 \| 1 \| \| PDLIM1 \| 0 \| -1.164629534 \| 0 \| 1 \| \| CTSZ \| 0 \| -1.178628763 \| 0 \| 1 \| \| ITGA6 \| 0 \| -1.273681173 \| 0 \| 1 \| \| FCGRT \| 0 \| -1.320501945 \| 0 \| 1 \| \| ST14 \| 0 \| -1.342430206 \| 0 \| 1 \| \| APP \| 0 \| -1.396224097 \| 0 \| 1 \| \| PSAP \| 0 \| -1.413642987 \| 0 \| 1 \| \| VEGFA \| 0 \| -1.416922724 \| 0 \| 1 \| \| RRBP1 \| 0 \| -1.445081729 \| 0 \| 1 \| \| DST \| 0 \| -1.47628291 \| 0 \| 1 \| \| TPM1 \| 0 \| -1.491717073 \| 0 \| 1 \| \| LGALS3BP \| 0 \| -1.589478744 \| 0 \| 1 \| \| DSP \| 0 \| -1.603307138 \| 0 \| 1 \| \| KLF4 \| 0 \| -1.637643907 \| 0 \| 1 \| \| GRN \| 0 \| -1.658214899 \| 0 \| 1 \| \| CD9 \| 0 \| -1.661180376 \| 0 \| 1 \| \| CDC42EP5 \| 0 \| -1.687579607 \| 0 \| 1 \| \| IFITM3 \| 0 \| -1.748601559 \| 0 \| 1 \| \| TMEM54 \| 0 \| -1.763520146 \| 0 \| 1 \| \| SMIM22 \| 0 \| -1.811050596 \| 0 \| 1 \| \| HES1 \| 0 \| -1.894951134 \| 0 \| 1 \| \| GSN \| 0 \| -1.896174596 \| 0 \| 1 \| \| SOX4 \| 0 \| -1.988863196 \| 0 \| 1 \| \| MARCKS \| 0 \| -2.091800638 \| 0 \| 1 \| \| EPCAM \| 0 \| -2.256511824 \| 0 \| 1 \| \| CLDN3 \| 0 \| -2.336745022 \| 0 \| 1 \| \| CLDN4 \| 0 \| -2.452179994 \| 0 \| 1 \| \| TSPAN8 \| 0 \| -2.474273979 \| 0 \| 1 \| \| ELF3 \| 0 \| -2.528395744 \| 0 \| 1 \| \| LGALS4 \| 0 \| -2.984704919 \| 0 \| 1 \| \| KRT19 \| 0 \| -2.989985343 \| 0 \| 1 \| \| KRT18 \| 0 \| -2.992796143 \| 0 \| 1 \| \| KRT8 \| 0 \| -3.095612154 \| 0 \| 1 \| \| IFI27 \| 0 \| -3.132499191 \| 0 \| 1 \| \| PHGR1 \| 0 \| -4.732204664 \| 0 \| 1 \| \| IER3 \| 3.1278E-302 \| -1.4125518 \| 6.8271E-298 \| 1 \| \| PIGR \| 1.2573E-301 \| -4.215381953 \| 2.7443E-297 \| 1 \| \| TSPAN3 \| 6.1427E-297 \| -1.023036983 \| 1.3408E-292 \| 1 \| \| FXYD3 \| 6.2749E-297 \| -2.66556293 \| 1.3696E-292 \| 1 \| \| TMEM176B \| 1.5234E-295 \| -1.377160008 \| 3.325E-291 \| 1 \| \| ARHGAP9 \| 7.8235E-295 \| 1.013900771 \| 1.7076E-290 \| 1 \| \| LGALS3 \| 9.5901E-294 \| -1.804059808 \| 2.0932E-289 \| 1 \| \| KLF5 \| 3.8069E-293 \| -1.475507874 \| 8.3093E-289 \| 1 \| \| GMFG \| 1.7136E-291 \| 1.016866729 \| 3.7403E-287 \| 1 \| \| CTNND1 \| 2.9801E-290 \| -1.013528813 \| 6.5046E-286 \| 1 \| \| AGR2 \| 5.398E-290 \| -2.699786605 \| 1.1782E-285 \| 1 \| \| TIMP1 \| 5.6836E-289 \| -1.896791101 \| 1.2406E-284 \| 1 \| \| LSP1 \| 7.5523E-288 \| 1.107939457 \| 1.6484E-283 \| 1 \| \| BCL11B \| 6.2032E-287 \| 1.019870048 \| 1.354E-282 \| 1 \| \| FAM3D \| 1.1411E-279 \| -1.848084011 \| 2.4907E-275 \| 1 \| \| CD24 \| 2.5978E-279 \| -1.832294208 \| 5.6703E-275 \| 1 \| \| SLC44A4 \| 6.356E-277 \| -1.425405011 \| 1.3873E-272 \| 1 \| \| IL7R \| 4.3551E-275 \| 1.030144448 \| 9.5059E-271 \| 1 \| \| MUC13 \| 4.4077E-274 \| -1.666467361 \| 9.6206E-270 \| 1 \| \| TMEM176A \| 1.5332E-269 \| -1.165589156 \| 3.3465E-265 \| 1 \| \| NET1 \| 3.5528E-264 \| -1.023909791 \| 7.7546E-260 \| 1 \| \| SPINT1 \| 2.0027E-262 \| -1.086936409 \| 4.3713E-258 \| 1 \| \| GIMAP4 \| 3.2728E-261 \| 1.097292633 \| 7.1434E-257 \| 1 \| \| S100A14 \| 3.4484E-260 \| -1.404927677 \| 7.5268E-256 \| 1 \| \| TSPAN1 \| 3.9417E-260 \| -2.253395812 \| 8.6035E-256 \| 1 \| \| ETS2 \| 5.2066E-260 \| -1.081650627 \| 1.1364E-255 \| 1 \| \| PDE4D \| 6.293E-258 \| 1.144825587 \| 1.3736E-253 \| 1 \| \| CAMK2N1 \| 5.5991E-256 \| -1.280678062 \| 1.2221E-251 \| 1 \| \| AC044849.1 \| 2.5746E-255 \| 1.086894159 \| 5.6196E-251 \| 1 \| \| GPX2 \| 8.6115E-255 \| -1.700632039 \| 1.8796E-250 \| 1 \| \| DSG2 \| 1.0784E-254 \| -1.224741592 \| 2.3539E-250 \| 1 \| \| TFF3 \| 1.6512E-253 \| -3.752438698 \| 3.604E-249 \| 1 \| \| TST \| 7.1614E-253 \| -1.080748246 \| 1.5631E-248 \| 1 \| \| ATP1B1 \| 7.9851E-251 \| -1.331379162 \| 1.7429E-246 \| 1 \| \| SELENOP \| 1.1542E-249 \| -2.474115263 \| 2.5193E-245 \| 1 \| \| TCF7L2 \| 2.3164E-248 \| -1.015995204 \| 5.056E-244 \| 1 \| \| AC058791.1 \| 3.9443E-247 \| 1.075997434 \| 8.6093E-243 \| 1 \| \| CD99 \| 4.1975E-247 \| 1.03208526 \| 9.1618E-243 \| 1 \| \| MISP \| 5.5511E-247 \| -1.349512817 \| 1.2116E-242 \| 1 \| \| CDH17 \| 1.5886E-245 \| -1.228402306 \| 3.4674E-241 \| 1 \| \| JUP \| 1.6176E-245 \| -1.003933717 \| 3.5308E-241 \| 1 \| \| MGLL \| 1.198E-243 \| -1.013340691 \| 2.6149E-239 \| 1 \| \| MUC12 \| 2.8301E-237 \| -2.049552228 \| 6.1773E-233 \| 1 \| \| RAB27A \| 2.7519E-236 \| 1.104413481 \| 6.0065E-232 \| 1 \| \| S100A16 \| 1.5896E-235 \| -1.022554695 \| 3.4697E-231 \| 1 \| \| RNF125 \| 9.3681E-235 \| 1.02656515 \| 2.0448E-230 \| 1 \| \| CKB \| 3.5472E-234 \| -2.205941898 \| 7.7425E-230 \| 1 \| \| LCN2 \| 2.2373E-230 \| -2.450386563 \| 4.8833E-226 \| 1 \| \| SPTBN1 \| 4.0934E-225 \| -1.048593016 \| 8.9347E-221 \| 1 \| \| PHLDA2 \| 2.5998E-218 \| -1.068044848 \| 5.6747E-214 \| 1 \| \| TAGAP \| 2.9279E-215 \| 1.068504028 \| 6.3908E-211 \| 1 \| \| CELF2 \| 3.2988E-212 \| 1.029599867 \| 7.2003E-208 \| 1 \| \| ZC3HAV1 \| 1.6974E-208 \| 1.02615821 \| 3.705E-204 \| 1 \| \| GLIPR1 \| 1.0982E-203 \| 1.023126319 \| 2.3971E-199 \| 1 \| \| PSMB9 \| 4.2276E-203 \| 1.06947264 \| 9.2275E-199 \| 1 \| \| WIPF1 \| 4.1476E-199 \| 1.019267669 \| 9.0529E-195 \| 1 \| \| CKLF \| 1.2751E-194 \| 1.158353947 \| 2.7831E-190 \| 1 \| \| CBLB \| 2.1475E-194 \| 1.022197124 \| 4.6873E-190 \| 1 \| \| CES2 \| 1.7781E-193 \| -1.085090158 \| 3.881E-189 \| 1 \| \| SLC12A2 \| 4.98E-192 \| -1.174270913 \| 1.087E-187 \| 1 \| \| UBE2S \| 4.1229E-178 \| 1.085954063 \| 8.999E-174 \| 1 \| \| DNAJB1 \| 3.6379E-170 \| 1.038425377 \| 7.9404E-166 \| 1 \| \| LYAR \| 5.2373E-167 \| 1.107239441 \| 1.1431E-162 \| 1 \| \| MYADM \| 4.9511E-164 \| 1.016076076 \| 1.0807E-159 \| 1 \| \| FABP1 \| 3.6528E-162 \| -3.552329958 \| 7.973E-158 \| 1 \| \| JCHAIN \| 1.746E-161 \| -5.505884853 \| 3.811E-157 \| 1 \| \| ADGRE5 \| 3.0178E-161 \| 1.077165806 \| 6.5869E-157 \| 1 \| \| LYZ \| 2.3966E-159 \| -3.071235682 \| 5.2311E-155 \| 1 \| \| S100A6 \| 1.4076E-155 \| -2.006237754 \| 3.0723E-151 \| 1 \| \| RNF19A \| 4.5017E-146 \| 1.038111343 \| 9.8258E-142 \| 1 \| \| TUBA4A \| 7.5289E-140 \| 1.171470044 \| 1.6433E-135 \| 1 \| \| IGHA1 \| 1.5869E-133 \| -5.287072177 \| 3.4638E-129 \| 1 \| \| METRNL \| 7.0608E-100 \| 1.087206582 \| 1.54116E-95 \| 1 \| \| PLAC8 \| 7.35057E-84 \| -1.137586726 \| 1.60441E-79 \| 1 \| \| SSR4 \| 6.07711E-75 \| -1.127392677 \| 1.32645E-70 \| 1 \| \| CMC1 \| 1.19608E-61 \| 1.28410907 \| 2.61067E-57 \| 1 \| \| FTL \| 8.8856E-43 \| -1.017772419 \| 1.93946E-38 \| 1 \| \| IGLC2 \| 2.02796E-17 \| -5.341495807 \| 4.42643E-13 \| 1 \| \| IGKC \| 3.63313E-17 \| -5.390456799 \| 7.93004E-13 \| 1 \| \| IGLC3 \| 5.1454E-16 \| -5.19778326 \| 1.12309E-11 \| 1 \| \| JCHAIN \| 0 \| 3.930776197 \| 0 \| 2 \| \| IGHA1 \| 0 \| 3.77722691 \| 0 \| 2 \| \| IGKC \| 0 \| 3.619135905 \| 0 \| 2 \| \| IGHA2 \| 0 \| 3.02788453 \| 0 \| 2 \| \| MZB1 \| 0 \| 2.308316777 \| 0 \| 2 \| \| SSR4 \| 0 \| 2.087189946 \| 0 \| 2 \| \| TNFRSF17 \| 0 \| 1.663520761 \| 0 \| 2 \| \| DERL3 \| 0 \| 1.657734048 \| 0 \| 2 \| \| SEC11C \| 0 \| 1.58314937 \| 0 \| 2 \| \| FKBP11 \| 0 \| 1.541489028 \| 0 \| 2 \| \| HERPUD1 \| 0 \| 1.394431811 \| 0 \| 2 \| \| XBP1 \| 0 \| 1.159978452 \| 0 \| 2 \| \| HSP90B1 \| 0 \| 1.128285834 \| 0 \| 2 \| \| SDF2L1 \| 0 \| 1.084717016 \| 0 \| 2 \| \| IGKJ5 \| 0 \| 1.065097536 \| 0 \| 2 \| \| DUSP5 \| 0 \| 1.024202111 \| 0 \| 2 \| \| UBE2J1 \| 0 \| 1.02234175 \| 0 \| 2 \| \| RPS8 \| 0 \| -1.005095021 \| 0 \| 2 \| \| RPS15 \| 0 \| -1.032037023 \| 0 \| 2 \| \| UBA52 \| 0 \| -1.037486079 \| 0 \| 2 \| \| RPS16 \| 0 \| -1.042547295 \| 0 \| 2 \| \| HLA-C \| 0 \| -1.04494327 \| 0 \| 2 \| \| CHCHD2 \| 0 \| -1.047481528 \| 0 \| 2 \| \| NACA \| 0 \| -1.060580941 \| 0 \| 2 \| \| RPS15A \| 0 \| -1.068102914 \| 0 \| 2 \| \| RPL23A \| 0 \| -1.079825458 \| 0 \| 2 \| \| RPS7 \| 0 \| -1.104793518 \| 0 \| 2 \| \| RACK1 \| 0 \| -1.105391647 \| 0 \| 2 \| \| RPS19 \| 0 \| -1.107062807 \| 0 \| 2 \| \| RPL29 \| 0 \| -1.112947145 \| 0 \| 2 \| \| RPL14 \| 0 \| -1.113072594 \| 0 \| 2 \| \| RPS14 \| 0 \| -1.113650055 \| 0 \| 2 \| \| RPL27 \| 0 \| -1.127041357 \| 0 \| 2 \| \| RPL35 \| 0 \| -1.152273395 \| 0 \| 2 \| \| RPS27A \| 0 \| -1.157119727 \| 0 \| 2 \| \| RPL9 \| 0 \| -1.161025003 \| 0 \| 2 \| \| RPL37A \| 0 \| -1.177843254 \| 0 \| 2 \| \| RPS13 \| 0 \| -1.188170326 \| 0 \| 2 \| \| RPL7 \| 0 \| -1.208269875 \| 0 \| 2 \| \| YBX1 \| 0 \| -1.213269798 \| 0 \| 2 \| \| EEF1A1 \| 0 \| -1.217093572 \| 0 \| 2 \| \| RPL21 \| 0 \| -1.224227526 \| 0 \| 2 \| \| TPT1 \| 0 \| -1.230522154 \| 0 \| 2 \| \| RPL10A \| 0 \| -1.246526736 \| 0 \| 2 \| \| RPLP2 \| 0 \| -1.252474897 \| 0 \| 2 \| \| RPL27A \| 0 \| -1.254917658 \| 0 \| 2 \| \| RPSA \| 0 \| -1.260338117 \| 0 \| 2 \| \| RPS3A \| 0 \| -1.261482216 \| 0 \| 2 \| \| DYNLL1 \| 0 \| -1.270196236 \| 0 \| 2 \| \| RPL11 \| 0 \| -1.270502947 \| 0 \| 2 \| \| RPS6 \| 0 \| -1.271128177 \| 0 \| 2 \| \| RPL13 \| 0 \| -1.273584061 \| 0 \| 2 \| \| UQCRB \| 0 \| -1.281954977 \| 0 \| 2 \| \| RPL32 \| 0 \| -1.282610907 \| 0 \| 2 \| \| DDX5 \| 0 \| -1.287820079 \| 0 \| 2 \| \| RPL19 \| 0 \| -1.288660211 \| 0 \| 2 \| \| SRP14 \| 0 \| -1.290309179 \| 0 \| 2 \| \| TOMM7 \| 0 \| -1.315931448 \| 0 \| 2 \| \| SLC25A3 \| 0 \| -1.321735245 \| 0 \| 2 \| \| RPS23 \| 0 \| -1.338151373 \| 0 \| 2 \| \| RPS18 \| 0 \| -1.345536147 \| 0 \| 2 \| \| RPL36 \| 0 \| -1.34907039 \| 0 \| 2 \| \| CALM1 \| 0 \| -1.349482035 \| 0 \| 2 \| \| HNRNPA2B1 \| 0 \| -1.351315781 \| 0 \| 2 \| \| ARPC3 \| 0 \| -1.362654234 \| 0 \| 2 \| \| RPL13A \| 0 \| -1.36305678 \| 0 \| 2 \| \| CAPZB \| 0 \| -1.363299221 \| 0 \| 2 \| \| RPS20 \| 0 \| -1.371148031 \| 0 \| 2 \| \| ATP5F1E \| 0 \| -1.376671458 \| 0 \| 2 \| \| HNRNPK \| 0 \| -1.380887735 \| 0 \| 2 \| \| RPS27 \| 0 \| -1.381399274 \| 0 \| 2 \| \| APLP2 \| 0 \| -1.385367348 \| 0 \| 2 \| \| PRRC2C \| 0 \| -1.388365893 \| 0 \| 2 \| \| RPS21 \| 0 \| -1.391534618 \| 0 \| 2 \| \| CAP1 \| 0 \| -1.400139136 \| 0 \| 2 \| \| RTN4 \| 0 \| -1.400764276 \| 0 \| 2 \| \| BTF3 \| 0 \| -1.402529329 \| 0 \| 2 \| \| RPL12 \| 0 \| -1.411880061 \| 0 \| 2 \| \| COX6C \| 0 \| -1.415067581 \| 0 \| 2 \| \| KIF5B \| 0 \| -1.421783177 \| 0 \| 2 \| \| RPL38 \| 0 \| -1.430271406 \| 0 \| 2 \| \| RPS2 \| 0 \| -1.430685743 \| 0 \| 2 \| \| RPL23 \| 0 \| -1.433836383 \| 0 \| 2 \| \| RPL37 \| 0 \| -1.444689685 \| 0 \| 2 \| \| SOD1 \| 0 \| -1.446800127 \| 0 \| 2 \| \| JPT1 \| 0 \| -1.452633229 \| 0 \| 2 \| \| ACTR2 \| 0 \| -1.455821342 \| 0 \| 2 \| \| TPM3 \| 0 \| -1.459567388 \| 0 \| 2 \| \| APP \| 0 \| -1.460451902 \| 0 \| 2 \| \| RHOC \| 0 \| -1.471443406 \| 0 \| 2 \| \| CLIC1 \| 0 \| -1.48758983 \| 0 \| 2 \| \| RPS3 \| 0 \| -1.488764099 \| 0 \| 2 \| \| RPS12 \| 0 \| -1.489596834 \| 0 \| 2 \| \| RPS29 \| 0 \| -1.490200894 \| 0 \| 2 \| \| RPL30 \| 0 \| -1.507616882 \| 0 \| 2 \| \| SDCBP \| 0 \| -1.512772035 \| 0 \| 2 \| \| ETS2 \| 0 \| -1.521929742 \| 0 \| 2 \| \| MALAT1 \| 0 \| -1.526612696 \| 0 \| 2 \| \| HLA-E \| 0 \| -1.527194086 \| 0 \| 2 \| \| RPL28 \| 0 \| -1.548885557 \| 0 \| 2 \| \| ARGLU1 \| 0 \| -1.553521874 \| 0 \| 2 \| \| HMGB1 \| 0 \| -1.578914757 \| 0 \| 2 \| \| RPS24 \| 0 \| -1.580480426 \| 0 \| 2 \| \| CIB1 \| 0 \| -1.583540985 \| 0 \| 2 \| \| CAMK2N1 \| 0 \| -1.585295088 \| 0 \| 2 \| \| MYL6 \| 0 \| -1.593491775 \| 0 \| 2 \| \| RAC1 \| 0 \| -1.616463332 \| 0 \| 2 \| \| RPL31 \| 0 \| -1.637852664 \| 0 \| 2 \| \| TPI1 \| 0 \| -1.64396818 \| 0 \| 2 \| \| PTMA \| 0 \| -1.654716952 \| 0 \| 2 \| \| TPM1 \| 0 \| -1.665377537 \| 0 \| 2 \| \| LDHA \| 0 \| -1.69485239 \| 0 \| 2 \| \| LGALS3BP \| 0 \| -1.710857994 \| 0 \| 2 \| \| TUBA1B \| 0 \| -1.721112372 \| 0 \| 2 \| \| GLUL \| 0 \| -1.743084347 \| 0 \| 2 \| \| YWHAB \| 0 \| -1.763699478 \| 0 \| 2 \| \| MYH9 \| 0 \| -1.770962333 \| 0 \| 2 \| \| ENO1 \| 0 \| -1.774127616 \| 0 \| 2 \| \| COTL1 \| 0 \| -1.803900655 \| 0 \| 2 \| \| SAT1 \| 0 \| -1.806914608 \| 0 \| 2 \| \| VMP1 \| 0 \| -1.80916391 \| 0 \| 2 \| \| DEK \| 0 \| -1.816890252 \| 0 \| 2 \| \| PPDPF \| 0 \| -1.88054952 \| 0 \| 2 \| \| GSN \| 0 \| -2.001380953 \| 0 \| 2 \| \| MYL12A \| 0 \| -2.016826508 \| 0 \| 2 \| \| GAPDH \| 0 \| -2.035852036 \| 0 \| 2 \| \| PFN1 \| 0 \| -2.073175015 \| 0 \| 2 \| \| REL \| 0 \| -2.086868599 \| 0 \| 2 \| \| HES1 \| 0 \| -2.093660885 \| 0 \| 2 \| \| TMSB4X \| 0 \| -2.094800096 \| 0 \| 2 \| \| FTH1 \| 0 \| -2.167047507 \| 0 \| 2 \| \| ACTG1 \| 0 \| -2.186411025 \| 0 \| 2 \| \| MYL12B \| 0 \| -2.194013287 \| 0 \| 2 \| \| BTG1 \| 0 \| -2.208695371 \| 0 \| 2 \| \| SH3BGRL3 \| 0 \| -2.322334717 \| 0 \| 2 \| \| EPCAM \| 0 \| -2.355103633 \| 0 \| 2 \| \| S100A11 \| 0 \| -2.369874687 \| 0 \| 2 \| \| TMSB10 \| 0 \| -2.398539689 \| 0 \| 2 \| \| SOX4 \| 0 \| -2.507779201 \| 0 \| 2 \| \| HLA-A \| 0 \| -2.520639603 \| 0 \| 2 \| \| CLDN4 \| 0 \| -2.56848717 \| 0 \| 2 \| \| ACTB \| 0 \| -2.639459194 \| 0 \| 2 \| \| ELF3 \| 0 \| -2.686648535 \| 0 \| 2 \| \| LGALS3 \| 0 \| -2.841781841 \| 0 \| 2 \| \| S100A10 \| 0 \| -3.129359794 \| 0 \| 2 \| \| KRT19 \| 0 \| -3.206248537 \| 0 \| 2 \| \| LGALS4 \| 0 \| -3.22060123 \| 0 \| 2 \| \| IFI27 \| 0 \| -3.32374918 \| 0 \| 2 \| \| KRT8 \| 0 \| -3.331792563 \| 0 \| 2 \| \| KRT18 \| 0 \| -3.342332351 \| 0 \| 2 \| \| S100A6 \| 0 \| -4.158579103 \| 0 \| 2 \| \| ANXA2 \| 1.5402E-307 \| -1.858921761 \| 3.3618E-303 \| 2 \| \| WSB1 \| 1.2547E-305 \| -1.433155847 \| 2.7385E-301 \| 2 \| \| H2AFY \| 1.8627E-305 \| -1.311389792 \| 4.0657E-301 \| 2 \| \| COX5B \| 4.3538E-305 \| -1.587242401 \| 9.5031E-301 \| 2 \| \| EFHD2 \| 1.3741E-304 \| -1.504304644 \| 2.9993E-300 \| 2 \| \| COMMD6 \| 1.4795E-304 \| -1.396840723 \| 3.2293E-300 \| 2 \| \| IFITM3 \| 1.6065E-304 \| -1.890199831 \| 3.5065E-300 \| 2 \| \| ST14 \| 3.1819E-303 \| -1.384927799 \| 6.9452E-299 \| 2 \| \| CDC42EP5 \| 6.5966E-303 \| -1.700331761 \| 1.4398E-298 \| 2 \| \| SKP1 \| 3.4905E-302 \| -1.221937991 \| 7.6188E-298 \| 2 \| \| CLDN3 \| 4.3133E-302 \| -2.351393885 \| 9.4146E-298 \| 2 \| \| YWHAZ \| 2.3955E-300 \| -1.300621559 \| 5.2286E-296 \| 2 \| \| KLF4 \| 1.3247E-299 \| -1.653023812 \| 2.8915E-295 \| 2 \| \| CSTB \| 1.3837E-299 \| -1.62215879 \| 3.0202E-295 \| 2 \| \| TSPAN8 \| 1.7389E-298 \| -2.641162459 \| 3.7954E-294 \| 2 \| \| TSC22D1 \| 2.2638E-298 \| -1.527006167 \| 4.9411E-294 \| 2 \| \| CEBPD \| 1.0831E-297 \| -1.655240561 \| 2.3641E-293 \| 2 \| \| TSPO \| 1.6671E-296 \| -1.501955832 \| 3.6387E-292 \| 2 \| \| LPP \| 3.0225E-296 \| -1.301308301 \| 6.5973E-292 \| 2 \| \| CDC42 \| 4.5535E-296 \| -1.27504103 \| 9.939E-292 \| 2 \| \| PABPC1 \| 2.5007E-294 \| -1.455715158 \| 5.4584E-290 \| 2 \| \| ARPC2 \| 3.0644E-294 \| -1.231001495 \| 6.6887E-290 \| 2 \| \| PKM \| 4.8273E-291 \| -1.497389065 \| 1.0536E-286 \| 2 \| \| ACTN4 \| 1.641E-290 \| -1.307700972 \| 3.5819E-286 \| 2 \| \| CD9 \| 9.7529E-290 \| -1.958725854 \| 2.1288E-285 \| 2 \| \| CTNND1 \| 1.8283E-288 \| -1.117272419 \| 3.9906E-284 \| 2 \| \| SMIM22 \| 2.3683E-288 \| -1.848782856 \| 5.1693E-284 \| 2 \| \| DSP \| 3.5192E-288 \| -1.65093032 \| 7.6813E-284 \| 2 \| \| EIF1 \| 7.3934E-284 \| -1.033018934 \| 1.6138E-279 \| 2 \| \| AGR2 \| 2.3836E-282 \| -2.967383809 \| 5.2027E-278 \| 2 \| \| CCNI \| 5.8216E-278 \| -1.11644738 \| 1.2707E-273 \| 2 \| \| ANXA11 \| 8.8004E-277 \| -1.32366766 \| 1.9209E-272 \| 2 \| \| CXCR4 \| 1.7908E-276 \| -2.789457613 \| 3.9087E-272 \| 2 \| \| PTBP3 \| 7.0716E-276 \| -1.261998993 \| 1.5435E-271 \| 2 \| \| COX7B \| 1.0847E-275 \| -1.366936339 \| 2.3676E-271 \| 2 \| \| PSMA7 \| 2.9369E-275 \| -1.287061823 \| 6.4104E-271 \| 2 \| \| IQGAP1 \| 4.4874E-274 \| -1.284116684 \| 9.7947E-270 \| 2 \| \| SPTBN1 \| 5.3112E-274 \| -1.592840554 \| 1.1593E-269 \| 2 \| \| EIF4G2 \| 1.0703E-273 \| -1.228408563 \| 2.3363E-269 \| 2 \| \| SERBP1 \| 5.7737E-273 \| -1.268413131 \| 1.2602E-268 \| 2 \| \| TMEM54 \| 1.9023E-270 \| -1.8062157 \| 4.1522E-266 \| 2 \| \| CD24 \| 4.215E-270 \| -2.018517636 \| 9.2001E-266 \| 2 \| \| AES \| 1.8537E-269 \| -1.274536857 \| 4.046E-265 \| 2 \| \| TGOLN2 \| 3.0226E-268 \| -1.26503967 \| 6.5974E-264 \| 2 \| \| FXYD3 \| 3.9217E-267 \| -2.856763196 \| 8.5598E-263 \| 2 \| \| PIGR \| 7.0008E-267 \| -4.557649875 \| 1.5281E-262 \| 2 \| \| HNRNPU \| 4.0759E-266 \| -1.320173256 \| 8.8964E-262 \| 2 \| \| IRS2 \| 1.6212E-265 \| -1.267376124 \| 3.5385E-261 \| 2 \| \| SLC25A5 \| 2.8033E-265 \| -1.432992263 \| 6.1189E-261 \| 2 \| \| TMEM176B \| 9.192E-262 \| -1.398898594 \| 2.0063E-257 \| 2 \| \| LAMP1 \| 1.9281E-261 \| -1.266687316 \| 4.2085E-257 \| 2 \| \| ATP1B1 \| 2.7731E-261 \| -1.845721151 \| 6.0529E-257 \| 2 \| \| FOXP1 \| 1.2117E-259 \| -1.310026452 \| 2.6448E-255 \| 2 \| \| RHOA \| 2.9655E-259 \| -1.257490063 \| 6.4729E-255 \| 2 \| \| ANXA1 \| 7.4233E-259 \| -2.327724604 \| 1.6203E-254 \| 2 \| \| AKAP13 \| 6.2496E-258 \| -1.384100019 \| 1.3641E-253 \| 2 \| \| MUC13 \| 7.5081E-257 \| -1.770438071 \| 1.6388E-252 \| 2 \| \| SERPINB1 \| 4.9033E-254 \| -1.230940253 \| 1.0702E-249 \| 2 \| \| ANXA4 \| 9.1472E-254 \| -1.123152253 \| 1.9966E-249 \| 2 \| \| KLF5 \| 4.3709E-253 \| -1.524675542 \| 9.5403E-249 \| 2 \| \| ID2 \| 1.1937E-252 \| -1.608589172 \| 2.6054E-248 \| 2 \| \| CEMIP2 \| 2.4651E-252 \| -1.302767652 \| 5.3805E-248 \| 2 \| \| S100A14 \| 6.7745E-252 \| -1.508761292 \| 1.4787E-247 \| 2 \| \| FAM3D \| 1.2753E-251 \| -1.891079309 \| 2.7836E-247 \| 2 \| \| GCC2 \| 4.4216E-251 \| -1.391271485 \| 9.651E-247 \| 2 \| \| SLC44A4 \| 4.8582E-251 \| -1.463833745 \| 1.0604E-246 \| 2 \| \| PGK1 \| 1.5322E-250 \| -1.342614157 \| 3.3442E-246 \| 2 \| \| MARCKSL1 \| 9.4754E-250 \| -1.448147964 \| 2.0682E-245 \| 2 \| \| IVNS1ABP \| 2.2292E-249 \| -1.396725448 \| 4.8656E-245 \| 2 \| \| ELOB \| 5.0408E-245 \| -1.265948092 \| 1.1003E-240 \| 2 \| \| TCF7L2 \| 7.0829E-245 \| -1.115588131 \| 1.546E-240 \| 2 \| \| PHGR1 \| 2.8289E-244 \| -5.0009504 \| 6.1746E-240 \| 2 \| \| CDH17 \| 8.2404E-243 \| -1.339446008 \| 1.7986E-238 \| 2 \| \| YBX3 \| 1.0325E-242 \| -1.353675339 \| 2.2537E-238 \| 2 \| \| NBL1 \| 3.2198E-242 \| -1.106866079 \| 7.0279E-238 \| 2 \| \| RNF213 \| 5.6897E-242 \| -1.408866785 \| 1.2419E-237 \| 2 \| \| HNRNPF \| 8.9756E-242 \| -1.19109763 \| 1.9591E-237 \| 2 \| \| HSPA8 \| 1.1016E-241 \| -1.204989704 \| 2.4045E-237 \| 2 \| \| TMEM176A \| 5.5461E-241 \| -1.192023671 \| 1.2105E-236 \| 2 \| \| SERPINB6 \| 1.9663E-240 \| -1.064544433 \| 4.2917E-236 \| 2 \| \| LAPTM5 \| 2.3087E-240 \| -1.958456554 \| 5.0392E-236 \| 2 \| \| LIMA1 \| 5.136E-239 \| -1.28984509 \| 1.121E-234 \| 2 \| \| DSG2 \| 2.3452E-237 \| -1.277074985 \| 5.1189E-233 \| 2 \| \| SRI \| 2.9565E-237 \| -1.619442081 \| 6.4532E-233 \| 2 \| \| CAST \| 1.5758E-236 \| -1.265742432 \| 3.4395E-232 \| 2 \| \| CTNNA1 \| 3.1494E-235 \| -1.049712643 \| 6.8743E-231 \| 2 \| \| TXN \| 1.4392E-231 \| -1.637452001 \| 3.1413E-227 \| 2 \| \| ZFP36L1 \| 1.5916E-231 \| -1.455134068 \| 3.474E-227 \| 2 \| \| S100A16 \| 2.7577E-231 \| -1.110139199 \| 6.0193E-227 \| 2 \| \| ATP1B3 \| 7.3329E-230 \| -1.595675487 \| 1.6006E-225 \| 2 \| \| GRN \| 6.1861E-229 \| -1.558378942 \| 1.3502E-224 \| 2 \| \| ACTR3 \| 1.333E-228 \| -1.22552544 \| 2.9096E-224 \| 2 \| \| COX6B1 \| 5.354E-228 \| -1.150995245 \| 1.1686E-223 \| 2 \| \| TFF3 \| 8.7448E-228 \| -4.094041798 \| 1.9087E-223 \| 2 \| \| MISP \| 1.4303E-226 \| -1.380249127 \| 3.122E-222 \| 2 \| \| SPINT1 \| 4.7614E-226 \| -1.110294619 \| 1.0393E-221 \| 2 \| \| TSPAN1 \| 1.2051E-224 \| -2.33588335 \| 2.6303E-220 \| 2 \| \| DST \| 3.1427E-224 \| -1.426601324 \| 6.8595E-220 \| 2 \| \| SELENOP \| 2.2612E-222 \| -2.535857108 \| 4.9356E-218 \| 2 \| \| SON \| 5.1874E-222 \| -1.073939265 \| 1.1323E-217 \| 2 \| \| GPX2 \| 5.8748E-222 \| -1.800340489 \| 1.2823E-217 \| 2 \| \| KLF3 \| 1.2362E-221 \| -1.070211936 \| 2.6982E-217 \| 2 \| \| HSP90AB1 \| 3.1828E-221 \| -1.09296739 \| 6.9472E-217 \| 2 \| \| DYNC1H1 \| 1.3167E-220 \| -1.145111548 \| 2.874E-216 \| 2 \| \| DBI \| 2.0992E-220 \| -1.295558651 \| 4.582E-216 \| 2 \| \| WDR1 \| 2.251E-220 \| -1.05073298 \| 4.9132E-216 \| 2 \| \| SAP18 \| 2.3842E-219 \| -1.085329943 \| 5.2039E-215 \| 2 \| \| PTMS \| 5.5316E-219 \| -1.259962539 \| 1.2074E-214 \| 2 \| \| MTCH1 \| 2.1267E-217 \| -1.03694997 \| 4.6419E-213 \| 2 \| \| MUC12 \| 8.9868E-217 \| -2.151686035 \| 1.9616E-212 \| 2 \| \| RAB11FIP1 \| 1.5173E-216 \| -1.421713402 \| 3.3118E-212 \| 2 \| \| ITGB1 \| 5.058E-215 \| -1.058402255 \| 1.104E-210 \| 2 \| \| ITM2B \| 2.7702E-214 \| -1.336399116 \| 6.0464E-210 \| 2 \| \| EML4 \| 8.9188E-214 \| -1.124133765 \| 1.9467E-209 \| 2 \| \| NDUFS5 \| 4.5018E-213 \| -1.165888901 \| 9.826E-209 \| 2 \| \| SCP2 \| 5.505E-213 \| -1.152831187 \| 1.2016E-208 \| 2 \| \| PIK3R1 \| 1.8479E-212 \| -1.394672112 \| 4.0335E-208 \| 2 \| \| TUBA1A \| 5.396E-209 \| -1.509234923 \| 1.1778E-204 \| 2 \| \| ATP5F1B \| 4.1309E-208 \| -1.27675912 \| 9.0166E-204 \| 2 \| \| SPATS2L \| 1.4369E-207 \| -1.038676904 \| 3.1363E-203 \| 2 \| \| MVP \| 6.1299E-207 \| -1.105983471 \| 1.338E-202 \| 2 \| \| LCN2 \| 3.1923E-206 \| -2.597087883 \| 6.9678E-202 \| 2 \| \| TRIR \| 6.2347E-206 \| -1.09305086 \| 1.3608E-201 \| 2 \| \| RAN \| 1.0056E-205 \| -1.212953398 \| 2.1949E-201 \| 2 \| \| PERP \| 2.7027E-205 \| -1.507556702 \| 5.8992E-201 \| 2 \| \| NPM1 \| 3.7549E-205 \| -1.159045974 \| 8.1959E-201 \| 2 \| \| RPS10 \| 4.6309E-205 \| -1.051678894 \| 1.0108E-200 \| 2 \| \| VASP \| 6.5978E-205 \| -1.00586462 \| 1.4401E-200 \| 2 \| \| ARL4A \| 1.3221E-204 \| -1.175944736 \| 2.8857E-200 \| 2 \| \| FRYL \| 3.8779E-204 \| -1.251848377 \| 8.4643E-200 \| 2 \| \| COX4I1 \| 4.108E-204 \| -1.191797791 \| 8.9666E-200 \| 2 \| \| SELENOW \| 3.9719E-202 \| -1.068558326 \| 8.6695E-198 \| 2 \| \| ZFAS1 \| 6.4176E-202 \| -1.24943254 \| 1.4008E-197 \| 2 \| \| NCOR1 \| 4.3796E-201 \| -1.139215172 \| 9.5594E-197 \| 2 \| \| NAP1L1 \| 1.6221E-200 \| -1.308225009 \| 3.5406E-196 \| 2 \| \| LCP1 \| 8.4259E-197 \| -1.681948454 \| 1.8391E-192 \| 2 \| \| CSNK1A1 \| 6.7333E-196 \| -1.098605797 \| 1.4697E-191 \| 2 \| \| FYB1 \| 1.547E-195 \| -1.742775606 \| 3.3767E-191 \| 2 \| \| TAGLN2 \| 4.2026E-195 \| -1.174920643 \| 9.1729E-191 \| 2 \| \| PRR13 \| 7.0201E-195 \| -1.018501903 \| 1.5323E-190 \| 2 \| \| ZBTB7A \| 2.7677E-194 \| -1.135598608 \| 6.0411E-190 \| 2 \| \| HNRNPAB \| 1.0666E-193 \| -1.191423874 \| 2.328E-189 \| 2 \| \| NUCKS1 \| 6.8874E-193 \| -1.251433942 \| 1.5033E-188 \| 2 \| \| CDC37 \| 2.8971E-192 \| -1.106480969 \| 6.3236E-188 \| 2 \| \| SNX6 \| 3.3271E-191 \| -1.034350293 \| 7.2621E-187 \| 2 \| \| H2AFJ \| 6.761E-191 \| -1.068360177 \| 1.4757E-186 \| 2 \| \| SRSF3 \| 1.2493E-189 \| -1.010779747 \| 2.7268E-185 \| 2 \| \| METRNL \| 1.3273E-189 \| -1.065877079 \| 2.8972E-185 \| 2 \| \| HADHA \| 1.7825E-189 \| -1.014760408 \| 3.8906E-185 \| 2 \| \| NCL \| 3.9032E-189 \| -1.059289106 \| 8.5195E-185 \| 2 \| \| SRSF11 \| 1.0162E-188 \| -1.096462851 \| 2.2181E-184 \| 2 \| \| ARL6IP5 \| 4.0399E-188 \| -1.117621719 \| 8.8178E-184 \| 2 \| \| BAZ1A \| 1.6512E-186 \| -1.08610473 \| 3.6041E-182 \| 2 \| \| TNFSF10 \| 2.1139E-186 \| -1.107089009 \| 4.6141E-182 \| 2 \| \| DYNLT1 \| 3.6633E-186 \| -1.013533781 \| 7.9959E-182 \| 2 \| \| EIF4A2 \| 4.0823E-186 \| -1.035752235 \| 8.9104E-182 \| 2 \| \| CCT6A \| 4.6499E-186 \| -1.03514753 \| 1.0149E-181 \| 2 \| \| HNRNPA3 \| 4.8615E-186 \| -1.115503414 \| 1.0611E-181 \| 2 \| \| GNB1 \| 5.6471E-186 \| -1.043202195 \| 1.2326E-181 \| 2 \| \| CES2 \| 9.6105E-186 \| -1.269028282 \| 2.0977E-181 \| 2 \| \| VAMP8 \| 1.3912E-185 \| -1.341036866 \| 3.0365E-181 \| 2 \| \| CHD1 \| 4.1532E-185 \| -1.126389339 \| 9.0653E-181 \| 2 \| \| PTGER4 \| 6.9577E-185 \| -1.072141135 \| 1.5187E-180 \| 2 \| \| SF3B1 \| 8.8953E-185 \| -1.076434007 \| 1.9416E-180 \| 2 \| \| MTPN \| 1.859E-184 \| -1.019581053 \| 4.0576E-180 \| 2 \| \| MT2A \| 1.496E-183 \| -2.145098754 \| 3.2654E-179 \| 2 \| \| SLC12A2 \| 2.1913E-183 \| -1.50832924 \| 4.7829E-179 \| 2 \| \| WASF2 \| 6.7443E-183 \| -1.002015657 \| 1.4721E-178 \| 2 \| \| PLEC \| 2.933E-182 \| -1.023156007 \| 6.4019E-178 \| 2 \| \| PAK2 \| 5.4279E-182 \| -1.063425151 \| 1.1847E-177 \| 2 \| \| CRYBG1 \| 7.3002E-182 \| -1.045392941 \| 1.5934E-177 \| 2 \| \| RPS26 \| 1.5572E-181 \| -1.017466499 \| 3.3988E-177 \| 2 \| \| IRF2BP2 \| 2.8808E-179 \| -1.108276749 \| 6.288E-175 \| 2 \| \| LUC7L3 \| 8.2152E-179 \| -1.199966841 \| 1.7931E-174 \| 2 \| \| IER5 \| 1.3561E-178 \| -1.344493344 \| 2.9599E-174 \| 2 \| \| DDX17 \| 1.8613E-178 \| -1.047836299 \| 4.0626E-174 \| 2 \| \| PLAC8 \| 6.5904E-178 \| -1.890239023 \| 1.4385E-173 \| 2 \| \| PTGES3 \| 7.0009E-178 \| -1.077894976 \| 1.5281E-173 \| 2 \| \| S100A4 \| 1.135E-177 \| -2.116040079 \| 2.4774E-173 \| 2 \| \| TUBB \| 4.2798E-177 \| -1.229052341 \| 9.3415E-173 \| 2 \| \| ZKSCAN1 \| 5.9375E-177 \| -1.182445909 \| 1.296E-172 \| 2 \| \| ROCK1 \| 9.2606E-177 \| -1.100635645 \| 2.0213E-172 \| 2 \| \| BPTF \| 8.1519E-176 \| -1.178502078 \| 1.7793E-171 \| 2 \| \| TMA7 \| 1.7091E-174 \| -1.020566555 \| 3.7304E-170 \| 2 \| \| PDLIM1 \| 5.56E-174 \| -1.172604517 \| 1.2136E-169 \| 2 \| \| CD46 \| 3.2519E-173 \| -1.062714569 \| 7.098E-169 \| 2 \| \| TNFRSF1B \| 6.9763E-173 \| -1.203568301 \| 1.5227E-168 \| 2 \| \| ATP5MC3 \| 2.938E-171 \| -1.209741614 \| 6.4127E-167 \| 2 \| \| VDAC1 \| 2.5859E-170 \| -1.110696802 \| 5.6443E-166 \| 2 \| \| FOSL2 \| 4.8432E-170 \| -1.149312841 \| 1.0571E-165 \| 2 \| \| CKB \| 1.0327E-169 \| -2.346016432 \| 2.2541E-165 \| 2 \| \| TOB1 \| 3.3012E-169 \| -1.127264658 \| 7.2055E-165 \| 2 \| \| PRPF40A \| 4.7354E-168 \| -1.069312691 \| 1.0336E-163 \| 2 \| \| PSMB8 \| 6.2054E-168 \| -1.033941402 \| 1.3545E-163 \| 2 \| \| HSPD1 \| 1.7653E-167 \| -1.385704892 \| 3.853E-163 \| 2 \| \| SRSF7 \| 1.8558E-167 \| -1.135619862 \| 4.0506E-163 \| 2 \| \| DDX21 \| 3.7241E-167 \| -1.159540753 \| 8.1287E-163 \| 2 \| \| UBE2D3 \| 5.6827E-167 \| -1.056558635 \| 1.2404E-162 \| 2 \| \| ETHE1 \| 1.7924E-166 \| -1.102721327 \| 3.9123E-162 \| 2 \| \| CD99 \| 6.0518E-166 \| -1.197075542 \| 1.3209E-161 \| 2 \| \| 7-Sep \| 6.4951E-166 \| -1.153102427 \| 1.4177E-161 \| 2 \| \| PPP1R12A \| 2.5693E-165 \| -1.047411094 \| 5.608E-161 \| 2 \| \| CLTB \| 3.9849E-165 \| -1.2156118 \| 8.6978E-161 \| 2 \| \| PHLDA2 \| 9.0318E-165 \| -1.199882715 \| 1.9714E-160 \| 2 \| \| DNAJA1 \| 1.0286E-164 \| -1.474650188 \| 2.2451E-160 \| 2 \| \| ATP6V1G1 \| 1.5257E-164 \| -1.008193234 \| 3.3301E-160 \| 2 \| \| CD52 \| 1.0043E-163 \| -2.052126166 \| 2.192E-159 \| 2 \| \| ATP5MD \| 1.5606E-163 \| -1.044851398 \| 3.4063E-159 \| 2 \| \| ATP5ME \| 2.5084E-163 \| -1.122418835 \| 5.475E-159 \| 2 \| \| SET \| 9.6518E-163 \| -1.111840703 \| 2.1067E-158 \| 2 \| \| JAK1 \| 2.7776E-162 \| -1.11604191 \| 6.0627E-158 \| 2 \| \| KTN1 \| 2.2006E-161 \| -1.033854776 \| 4.8032E-157 \| 2 \| \| HLA-DPB1 \| 4.2702E-161 \| -2.563185845 \| 9.3206E-157 \| 2 \| \| AP2S1 \| 2.0775E-160 \| -1.039891229 \| 4.5346E-156 \| 2 \| \| CYCS \| 3.2075E-160 \| -1.075988945 \| 7.0009E-156 \| 2 \| \| WNK1 \| 6.9884E-160 \| -1.047394841 \| 1.5253E-155 \| 2 \| \| XRCC5 \| 7.2975E-159 \| -1.023955543 \| 1.5928E-154 \| 2 \| \| UQCR10 \| 1.9895E-158 \| -1.082511951 \| 4.3425E-154 \| 2 \| \| HIF1A \| 3.8678E-158 \| -1.109055322 \| 8.4422E-154 \| 2 \| \| LYZ \| 7.9744E-157 \| -3.409377412 \| 1.7406E-152 \| 2 \| \| VPS28 \| 2.11E-156 \| -1.034244311 \| 4.6056E-152 \| 2 \| \| CAPG \| 1.0074E-154 \| -1.134011534 \| 2.1987E-150 \| 2 \| \| GNG5 \| 1.1735E-154 \| -1.055259445 \| 2.5613E-150 \| 2 \| \| CD164 \| 2.6301E-154 \| -1.066679076 \| 5.7407E-150 \| 2 \| \| RSRC2 \| 5.8979E-154 \| -1.046735236 \| 1.2873E-149 \| 2 \| \| RPS25 \| 1.3983E-153 \| -1.036780633 \| 3.0521E-149 \| 2 \| \| CCND2 \| 1.8708E-153 \| -1.127911823 \| 4.0833E-149 \| 2 \| \| AMD1 \| 1.2899E-152 \| -1.165541798 \| 2.8154E-148 \| 2 \| \| UGP2 \| 2.7133E-152 \| -1.019424773 \| 5.9222E-148 \| 2 \| \| ST13 \| 1.5633E-151 \| -1.006694811 \| 3.4123E-147 \| 2 \| \| SYNE2 \| 1.7211E-151 \| -1.35126961 \| 3.7567E-147 \| 2 \| \| HLA-DRA \| 2.7892E-151 \| -3.688006152 \| 6.0879E-147 \| 2 \| \| MBNL1 \| 7.6638E-151 \| -1.10754059 \| 1.6728E-146 \| 2 \| \| RAD21 \| 5.9715E-150 \| -1.024153343 \| 1.3034E-145 \| 2 \| \| VEGFA \| 1.7136E-149 \| -1.264927909 \| 3.7404E-145 \| 2 \| \| PHLDA1 \| 2.0104E-149 \| -1.138377518 \| 4.3881E-145 \| 2 \| \| IER5L \| 1.0151E-147 \| -1.158520632 \| 2.2156E-143 \| 2 \| \| IL7R \| 7.8234E-147 \| -2.603800204 \| 1.7076E-142 \| 2 \| \| BSG \| 2.6762E-146 \| -1.257218377 \| 5.8414E-142 \| 2 \| \| RPL22 \| 3.1986E-145 \| -1.028405028 \| 6.9817E-141 \| 2 \| \| LSM7 \| 3.2106E-145 \| -1.035578001 \| 7.0078E-141 \| 2 \| \| PTP4A1 \| 2.4915E-144 \| -1.042874564 \| 5.4383E-140 \| 2 \| \| IER3 \| 5.745E-143 \| -1.556762902 \| 1.254E-138 \| 2 \| \| LIMS1 \| 5.7604E-143 \| -1.114998572 \| 1.2573E-138 \| 2 \| \| ARID4B \| 9.9222E-143 \| -1.092038902 \| 2.1657E-138 \| 2 \| \| JMJD1C \| 2.2722E-142 \| -1.006089378 \| 4.9596E-138 \| 2 \| \| HIST1H4C \| 1.1595E-141 \| -1.488289487 \| 2.5308E-137 \| 2 \| \| IGLC2 \| 5.2548E-141 \| 2.965629637 \| 1.147E-136 \| 2 \| \| CD3D \| 7.5147E-141 \| -1.865867472 \| 1.6402E-136 \| 2 \| \| ZC3HAV1 \| 9.7668E-141 \| -1.286228228 \| 2.1318E-136 \| 2 \| \| ARL4C \| 3.2562E-140 \| -1.510898937 \| 7.1073E-136 \| 2 \| \| TST \| 7.9306E-140 \| -1.037026593 \| 1.731E-135 \| 2 \| \| TPR \| 8.3453E-139 \| -1.040042523 \| 1.8215E-134 \| 2 \| \| ANP32B \| 1.0773E-138 \| -1.01989817 \| 2.3515E-134 \| 2 \| \| CD2 \| 1.4073E-138 \| -1.860387101 \| 3.0717E-134 \| 2 \| \| CACYBP \| 7.3159E-138 \| -1.276351503 \| 1.5968E-133 \| 2 \| \| ABRACL \| 3.3862E-137 \| -1.015247006 \| 7.3911E-133 \| 2 \| \| HMGN1 \| 4.5772E-136 \| -1.007207129 \| 9.9907E-132 \| 2 \| \| ARF6 \| 9.7454E-135 \| -1.009998099 \| 2.1271E-130 \| 2 \| \| BDP1 \| 4.6877E-134 \| -1.02523216 \| 1.0232E-129 \| 2 \| \| C4orf48 \| 6.5043E-134 \| -1.064989841 \| 1.4197E-129 \| 2 \| \| PRPF38B \| 3.8265E-133 \| -1.044930078 \| 8.3521E-129 \| 2 \| \| SARAF \| 1.2505E-132 \| -1.284010988 \| 2.7294E-128 \| 2 \| \| TLN1 \| 1.5948E-132 \| -1.044216299 \| 3.481E-128 \| 2 \| \| KMT2E \| 9.731E-132 \| -1.031345696 \| 2.124E-127 \| 2 \| \| BLOC1S1 \| 4.2107E-131 \| -1.063651881 \| 9.1907E-127 \| 2 \| \| SNRPD2 \| 7.0357E-131 \| -1.004309041 \| 1.5357E-126 \| 2 \| \| FABP1 \| 9.6581E-131 \| -3.923871374 \| 2.1081E-126 \| 2 \| \| HMGB2 \| 7.7213E-130 \| -1.250138402 \| 1.6853E-125 \| 2 \| \| NKTR \| 1.1367E-129 \| -1.077167912 \| 2.4811E-125 \| 2 \| \| CMTM6 \| 1.1616E-129 \| -1.028805797 \| 2.5354E-125 \| 2 \| \| HMGA1 \| 3.5358E-129 \| -1.00734822 \| 7.7175E-125 \| 2 \| \| TM9SF3 \| 2.1962E-128 \| -1.03071049 \| 4.7936E-124 \| 2 \| \| AKAP9 \| 6.0049E-128 \| -1.02851681 \| 1.3107E-123 \| 2 \| \| CREM \| 8.135E-127 \| -1.722686904 \| 1.7756E-122 \| 2 \| \| POMP \| 8.8891E-125 \| -1.02622944 \| 1.9402E-120 \| 2 \| \| ARHGDIB \| 1.3322E-124 \| -1.595463365 \| 2.9078E-120 \| 2 \| \| TRAC \| 2.0512E-124 \| -1.676145003 \| 4.4771E-120 \| 2 \| \| MIDN \| 2.8906E-124 \| -1.044183476 \| 6.3094E-120 \| 2 \| \| PTPRC \| 8.3133E-122 \| -1.880792602 \| 1.8145E-117 \| 2 \| \| SFPQ \| 2.2539E-120 \| -1.013298069 \| 4.9196E-116 \| 2 \| \| H2AFZ \| 5.968E-120 \| -1.190251839 \| 1.3026E-115 \| 2 \| \| TSPAN3 \| 8.3665E-120 \| -1.130145426 \| 1.8262E-115 \| 2 \| \| ATRX \| 9.2332E-120 \| -1.01463951 \| 2.0153E-115 \| 2 \| \| CD63 \| 1.0354E-117 \| -1.182258722 \| 2.2599E-113 \| 2 \| \| UQCRC1 \| 3.9812E-117 \| -1.008040666 \| 8.6897E-113 \| 2 \| \| SLC2A3 \| 5.4077E-117 \| -1.35331575 \| 1.1803E-112 \| 2 \| \| LITAF \| 8.421E-117 \| -1.252882551 \| 1.838E-112 \| 2 \| \| ELF1 \| 8.0832E-116 \| -1.119902705 \| 1.7643E-111 \| 2 \| \| FKBP5 \| 1.6413E-113 \| -1.108571937 \| 3.5825E-109 \| 2 \| \| FCGRT \| 2.2974E-112 \| -1.224974385 \| 5.0146E-108 \| 2 \| \| PNRC1 \| 8.0291E-112 \| -1.198277007 \| 1.7525E-107 \| 2 \| \| G3BP2 \| 2.2659E-109 \| -1.005969465 \| 4.9458E-105 \| 2 \| \| RPL39 \| 5.4599E-108 \| -1.157684694 \| 1.1917E-103 \| 2 \| \| RGS10 \| 5.9481E-108 \| -1.024885175 \| 1.2983E-103 \| 2 \| \| HSPH1 \| 3.8686E-107 \| -1.340597108 \| 8.4441E-103 \| 2 \| \| PPA1 \| 1.7199E-103 \| -1.096696395 \| 3.7539E-99 \| 2 \| \| CD81 \| 7.0375E-103 \| -1.299868669 \| 1.53608E-98 \| 2 \| \| HSPE1 \| 1.544E-102 \| -1.089214336 \| 3.36998E-98 \| 2 \| \| TNFAIP3 \| 1.247E-101 \| -1.974202251 \| 2.72179E-97 \| 2 \| \| HCST \| 2.47619E-98 \| -1.387793867 \| 5.40478E-94 \| 2 \| \| IDI1 \| 3.34122E-97 \| -1.013019602 \| 7.29288E-93 \| 2 \| \| SOCS1 \| 6.0663E-95 \| -1.071118777 \| 1.32409E-90 \| 2 \| \| HSPB1 \| 1.09502E-94 \| -1.036821527 \| 2.39009E-90 \| 2 \| \| JUNB \| 4.73687E-94 \| -1.040860187 \| 1.03392E-89 \| 2 \| \| IGLC3 \| 4.22467E-92 \| 2.865254054 \| 9.22118E-88 \| 2 \| \| DSTN \| 6.00753E-91 \| -1.25806496 \| 1.31126E-86 \| 2 \| \| ISG15 \| 1.61288E-90 \| -1.412828149 \| 3.52043E-86 \| 2 \| \| RHOB \| 2.61248E-88 \| -1.126282041 \| 5.70226E-84 \| 2 \| \| PDCD4 \| 9.85423E-88 \| -1.106960944 \| 2.15088E-83 \| 2 \| \| HEXIM1 \| 4.29995E-85 \| -1.242847062 \| 9.38549E-81 \| 2 \| \| NFKBIZ \| 2.14509E-84 \| -1.110349558 \| 4.6821E-80 \| 2 \| \| MT1X \| 3.33093E-84 \| -1.179622906 \| 7.27041E-80 \| 2 \| \| CEBPB \| 6.38823E-80 \| -1.219906304 \| 1.39436E-75 \| 2 \| \| GNAI2 \| 3.23443E-78 \| -1.124515973 \| 7.05978E-74 \| 2 \| \| PMEPA1 \| 6.30222E-78 \| -1.095357836 \| 1.37559E-73 \| 2 \| \| MARCKS \| 1.62953E-76 \| -1.249711444 \| 3.55677E-72 \| 2 \| \| AREG \| 5.17559E-76 \| -1.390129118 \| 1.12968E-71 \| 2 \| \| PRDX5 \| 6.27809E-73 \| -1.065766918 \| 1.37032E-68 \| 2 \| \| PSAP \| 1.89834E-69 \| -1.438484716 \| 4.14351E-65 \| 2 \| \| MSN \| 7.53063E-67 \| -1.043617458 \| 1.64371E-62 \| 2 \| \| LDHB \| 1.87913E-65 \| -1.060007975 \| 4.10157E-61 \| 2 \| \| RPS4Y1 \| 5.30731E-63 \| -1.153026935 \| 1.15843E-58 \| 2 \| \| SERTAD1 \| 2.53802E-62 \| -1.022087252 \| 5.53973E-58 \| 2 \| \| SAMSN1 \| 8.55224E-61 \| -1.323385993 \| 1.8667E-56 \| 2 \| \| CD37 \| 4.62619E-56 \| -1.275022197 \| 1.00976E-51 \| 2 \| \| RARRES3 \| 1.91717E-50 \| -1.051829163 \| 4.18461E-46 \| 2 \| \| SGK1 \| 2.56215E-49 \| -1.030547765 \| 5.59241E-45 \| 2 \| \| HLA-DPA1 \| 1.61521E-44 \| -2.35670781 \| 3.52552E-40 \| 2 \| \| CNOT6L \| 5.00013E-44 \| -1.06712402 \| 1.09138E-39 \| 2 \| \| DNAJB1 \| 5.09492E-42 \| -1.058170264 \| 1.11207E-37 \| 2 \| \| NR4A2 \| 1.48091E-40 \| -1.353260141 \| 3.23238E-36 \| 2 \| \| CLEC2B \| 5.52922E-33 \| -1.036852273 \| 1.20686E-28 \| 2 \| \| CD53 \| 8.70971E-23 \| -1.003301546 \| 1.90107E-18 \| 2 \| \| STK17B \| 4.18749E-22 \| -1.026573254 \| 9.14004E-18 \| 2 \| \| CD74 \| 9.54544E-21 \| -1.784021243 \| 2.08348E-16 \| 2 \| \| GPR183 \| 2.56855E-18 \| -1.499812574 \| 5.60638E-14 \| 2 \| \| CD69 \| 2.79909E-18 \| -1.481737508 \| 6.10958E-14 \| 2 \| \| SOX4 \| 0 \| 1.922422619 \| 0 \| 3 \| \| OLFM4 \| 0 \| 1.872946525 \| 0 \| 3 \| \| GPX2 \| 0 \| 1.822569057 \| 0 \| 3 \| \| SLC12A2 \| 0 \| 1.654168054 \| 0 \| 3 \| \| SOX9 \| 0 \| 1.64711439 \| 0 \| 3 \| \| RNF43 \| 0 \| 1.639743204 \| 0 \| 3 \| \| GDF15 \| 0 \| 1.616420508 \| 0 \| 3 \| \| DPEP1 \| 0 \| 1.579857344 \| 0 \| 3 \| \| KRT18 \| 0 \| 1.578756087 \| 0 \| 3 \| \| SCD \| 0 \| 1.556102328 \| 0 \| 3 \| \| CD24 \| 0 \| 1.459641335 \| 0 \| 3 \| \| VEGFA \| 0 \| 1.424176304 \| 0 \| 3 \| \| PERP \| 0 \| 1.41275371 \| 0 \| 3 \| \| PRDX5 \| 0 \| 1.347882896 \| 0 \| 3 \| \| AC103702.2 \| 0 \| 1.331647154 \| 0 \| 3 \| \| CLDN4 \| 0 \| 1.326033811 \| 0 \| 3 \| \| S100P \| 0 \| 1.306050729 \| 0 \| 3 \| \| HES1 \| 0 \| 1.302582017 \| 0 \| 3 \| \| ASCL2 \| 0 \| 1.287315835 \| 0 \| 3 \| \| PCCA \| 0 \| 1.278236345 \| 0 \| 3 \| \| FERMT1 \| 0 \| 1.272189555 \| 0 \| 3 \| \| DSP \| 0 \| 1.265207709 \| 0 \| 3 \| \| EPCAM \| 0 \| 1.242037063 \| 0 \| 3 \| \| FAT1 \| 0 \| 1.201229656 \| 0 \| 3 \| \| ZKSCAN1 \| 0 \| 1.195794051 \| 0 \| 3 \| \| ELF3 \| 0 \| 1.194190075 \| 0 \| 3 \| \| CLDN3 \| 0 \| 1.181689532 \| 0 \| 3 \| \| CEACAM6 \| 0 \| 1.179297193 \| 0 \| 3 \| \| PHLDA2 \| 0 \| 1.177540426 \| 0 \| 3 \| \| PLCB4 \| 0 \| 1.176734691 \| 0 \| 3 \| \| MACC1 \| 0 \| 1.156551459 \| 0 \| 3 \| \| LCN2 \| 0 \| 1.139703539 \| 0 \| 3 \| \| MYC \| 0 \| 1.121904236 \| 0 \| 3 \| \| AZGP1 \| 0 \| 1.111858229 \| 0 \| 3 \| \| MYO6 \| 0 \| 1.105890281 \| 0 \| 3 \| \| TSPAN8 \| 0 \| 1.078135977 \| 0 \| 3 \| \| TMC5 \| 0 \| 1.060067136 \| 0 \| 3 \| \| KRT8 \| 0 \| 1.05945155 \| 0 \| 3 \| \| FOXQ1 \| 0 \| 1.055158052 \| 0 \| 3 \| \| ANO9 \| 0 \| 1.046059917 \| 0 \| 3 \| \| TRIM2 \| 0 \| 1.030802521 \| 0 \| 3 \| \| RPL12 \| 0 \| 1.024789918 \| 0 \| 3 \| \| KNOP1 \| 0 \| 1.020547945 \| 0 \| 3 \| \| VIL1 \| 0 \| 1.019547201 \| 0 \| 3 \| \| EPHB3 \| 0 \| 1.019128631 \| 0 \| 3 \| \| MARCKSL1 \| 0 \| 1.017763423 \| 0 \| 3 \| \| ZNF703 \| 0 \| 1.015104808 \| 0 \| 3 \| \| ATP1B1 \| 0 \| 1.010423506 \| 0 \| 3 \| \| QPRT \| 0 \| 1.005858467 \| 0 \| 3 \| \| RPS18 \| 0 \| 1.00477274 \| 0 \| 3 \| \| PHGR1 \| 0 \| -1.352192858 \| 0 \| 3 \| \| TMSB4X \| 0 \| -1.407070745 \| 0 \| 3 \| \| B2M \| 0 \| -1.424676481 \| 0 \| 3 \| \| EMP3 \| 0 \| -1.451583541 \| 0 \| 3 \| \| JUNB \| 0 \| -1.494282293 \| 0 \| 3 \| \| GMFG \| 0 \| -1.551446313 \| 0 \| 3 \| \| DUSP1 \| 0 \| -1.557966131 \| 0 \| 3 \| \| CYTIP \| 0 \| -1.680976805 \| 0 \| 3 \| \| CD48 \| 0 \| -1.795505435 \| 0 \| 3 \| \| ARHGDIB \| 0 \| -1.998135993 \| 0 \| 3 \| \| CD52 \| 0 \| -2.433304843 \| 0 \| 3 \| \| PTPRC \| 0 \| -2.469201499 \| 0 \| 3 \| \| RGS1 \| 0 \| -2.492996411 \| 0 \| 3 \| \| TSC22D3 \| 0 \| -2.513633236 \| 0 \| 3 \| \| CXCR4 \| 0 \| -2.937736863 \| 0 \| 3 \| \| VIM \| 0 \| -3.368085259 \| 0 \| 3 \| \| SRGN \| 0 \| -3.402834097 \| 0 \| 3 \| \| LAPTM5 \| 6.6025E-290 \| -2.08848521 \| 1.4411E-285 \| 3 \| \| TNFAIP3 \| 4.1943E-288 \| -2.470116379 \| 9.1549E-284 \| 3 \| \| JUND \| 1.31E-280 \| -1.093610661 \| 2.8594E-276 \| 3 \| \| CD53 \| 4.0664E-272 \| -1.492457209 \| 8.8757E-268 \| 3 \| \| LCP1 \| 4.4919E-265 \| -1.81407402 \| 9.8044E-261 \| 3 \| \| ARGLU1 \| 8.9515E-265 \| 1.079193032 \| 1.9538E-260 \| 3 \| \| SMAP2 \| 7.9216E-263 \| -1.571539125 \| 1.729E-258 \| 3 \| \| LGALS1 \| 1.6842E-258 \| -2.250357103 \| 3.6761E-254 \| 3 \| \| SELENOK \| 2.7336E-258 \| -1.202875596 \| 5.9666E-254 \| 3 \| \| STK17B \| 4.0613E-254 \| -1.510230112 \| 8.8646E-250 \| 3 \| \| HLA-E \| 1.0589E-244 \| -1.008998889 \| 2.3112E-240 \| 3 \| \| BTG1 \| 2.8043E-244 \| -1.460083516 \| 6.121E-240 \| 3 \| \| PGGHG \| 8.2473E-243 \| 1.042416638 \| 1.8001E-238 \| 3 \| \| GYPC \| 5.0783E-241 \| -1.07391182 \| 1.1084E-236 \| 3 \| \| NFKBIA \| 1.1628E-240 \| -1.49940834 \| 2.5379E-236 \| 3 \| \| HERPUD1 \| 2.9637E-238 \| -1.473613234 \| 6.4688E-234 \| 3 \| \| CD69 \| 8.6636E-236 \| -2.426535183 \| 1.891E-231 \| 3 \| \| ZNF331 \| 1.131E-235 \| -1.910819069 \| 2.4686E-231 \| 3 \| \| CD37 \| 4.5039E-235 \| -1.531996788 \| 9.8306E-231 \| 3 \| \| LSP1 \| 4.8085E-234 \| -1.25657797 \| 1.0496E-229 \| 3 \| \| MSN \| 6.1201E-234 \| -1.334133383 \| 1.3358E-229 \| 3 \| \| SARAF \| 1.74E-229 \| -1.307474363 \| 3.798E-225 \| 3 \| \| NR4A2 \| 1.553E-228 \| -1.838554414 \| 3.3897E-224 \| 3 \| \| GPR183 \| 8.7491E-227 \| -1.941551125 \| 1.9097E-222 \| 3 \| \| HSPA1A \| 3.5131E-225 \| -1.778607856 \| 7.6681E-221 \| 3 \| \| HCST \| 6.55E-223 \| -1.687229496 \| 1.4297E-218 \| 3 \| \| PRDM1 \| 4.59E-222 \| -1.374253096 \| 1.0019E-217 \| 3 \| \| EVI2B \| 1.2031E-219 \| -1.082762851 \| 2.6261E-215 \| 3 \| \| CREM \| 2.9945E-218 \| -1.858793508 \| 6.5361E-214 \| 3 \| \| ISG20 \| 1.8998E-216 \| -1.254983852 \| 4.1467E-212 \| 3 \| \| FOSB \| 5.7782E-213 \| -1.065056718 \| 1.2612E-208 \| 3 \| \| FAM107B \| 2.0613E-211 \| -1.231119721 \| 4.4991E-207 \| 3 \| \| CLEC2B \| 2.1968E-211 \| -1.439257126 \| 4.795E-207 \| 3 \| \| SAMSN1 \| 2.3448E-202 \| -1.526267818 \| 5.118E-198 \| 3 \| \| SH3BGRL3 \| 4.0296E-199 \| -1.14908665 \| 8.7954E-195 \| 3 \| \| RGCC \| 7.1308E-197 \| -1.727580771 \| 1.5564E-192 \| 3 \| \| HCLS1 \| 1.1769E-196 \| -1.048419257 \| 2.5688E-192 \| 3 \| \| IFI16 \| 3.2936E-196 \| -1.118281582 \| 7.189E-192 \| 3 \| \| IL7R \| 1.6971E-194 \| -2.757873193 \| 3.7042E-190 \| 3 \| \| YPEL5 \| 2.1672E-194 \| -1.323925025 \| 4.7304E-190 \| 3 \| \| RAC2 \| 1.7337E-193 \| -1.326799037 \| 3.7841E-189 \| 3 \| \| CD3D \| 3.3571E-192 \| -2.043286342 \| 7.3276E-188 \| 3 \| \| IER5L \| 6.4043E-191 \| 1.040904561 \| 1.3979E-186 \| 3 \| \| SLC2A3 \| 8.9732E-187 \| -1.41378538 \| 1.9586E-182 \| 3 \| \| TXNIP \| 1.5481E-184 \| -1.375118323 \| 3.379E-180 \| 3 \| \| RHOH \| 2.4888E-184 \| -1.151921751 \| 5.4324E-180 \| 3 \| \| GLIPR1 \| 9.769E-184 \| -1.206796228 \| 2.1323E-179 \| 3 \| \| TRAC \| 1.4425E-183 \| -1.802026645 \| 3.1486E-179 \| 3 \| \| TAGAP \| 9.9123E-182 \| -1.15305917 \| 2.1636E-177 \| 3 \| \| CD2 \| 3.876E-180 \| -1.903589812 \| 8.4602E-176 \| 3 \| \| S100A4 \| 3.1787E-176 \| -1.939561108 \| 6.9382E-172 \| 3 \| \| ZFP36 \| 1.0764E-174 \| -1.03588782 \| 2.3494E-170 \| 3 \| \| PNRC1 \| 1.2628E-174 \| -1.149946988 \| 2.7563E-170 \| 3 \| \| CD3E \| 4.8347E-174 \| -1.627790503 \| 1.0553E-169 \| 3 \| \| FYB1 \| 2.0014E-172 \| -1.276552046 \| 4.3684E-168 \| 3 \| \| PDE4B \| 1.6848E-170 \| -1.064051204 \| 3.6774E-166 \| 3 \| \| TENT5C \| 1.0021E-168 \| -1.046646873 \| 2.1874E-164 \| 3 \| \| SOCS3 \| 2.467E-163 \| -1.058397849 \| 5.3848E-159 \| 3 \| \| FOS \| 8.96E-162 \| -1.123322877 \| 1.9557E-157 \| 3 \| \| GNAI2 \| 1.1802E-158 \| -1.140465322 \| 2.576E-154 \| 3 \| \| HLA-DPA1 \| 7.9778E-158 \| -2.430156485 \| 1.7413E-153 \| 3 \| \| KLF2 \| 2.2735E-157 \| -1.441692963 \| 4.9623E-153 \| 3 \| \| HLA-DPB1 \| 1.7231E-155 \| -2.500999665 \| 3.761E-151 \| 3 \| \| DNAJA1 \| 1.7844E-153 \| -1.325354101 \| 3.8949E-149 \| 3 \| \| DUSP2 \| 1.5478E-149 \| -1.678702253 \| 3.3784E-145 \| 3 \| \| FNBP1 \| 1.3945E-147 \| -1.006000405 \| 3.0437E-143 \| 3 \| \| AC058791.1 \| 1.8171E-145 \| -1.282388502 \| 3.9663E-141 \| 3 \| \| RARRES3 \| 2.9746E-143 \| -1.12652125 \| 6.4926E-139 \| 3 \| \| CD74 \| 4.5952E-143 \| -2.157830074 \| 1.003E-138 \| 3 \| \| FKBP5 \| 2.6934E-142 \| -1.092383633 \| 5.879E-138 \| 3 \| \| ARL4C \| 3.1501E-134 \| -1.407906175 \| 6.8757E-130 \| 3 \| \| PDCD4 \| 8.8218E-125 \| -1.012595437 \| 1.9255E-120 \| 3 \| \| RGS2 \| 5.9658E-117 \| -1.24445173 \| 1.3021E-112 \| 3 \| \| ANXA1 \| 1.5338E-106 \| -1.80471331 \| 3.3477E-102 \| 3 \| \| CNOT6L \| 9.54413E-95 \| -1.089692411 \| 2.0832E-90 \| 3 \| \| REL \| 1.49432E-87 \| -1.415218274 \| 3.26165E-83 \| 3 \| \| COTL1 \| 5.51782E-87 \| -1.275517721 \| 1.20438E-82 \| 3 \| \| MT2A \| 1.96927E-69 \| -1.173144309 \| 4.29833E-65 \| 3 \| \| IGKC \| 1.10718E-68 \| -3.850746402 \| 2.41663E-64 \| 3 \| \| HLA-DRA \| 3.98619E-64 \| -3.209916624 \| 8.70065E-60 \| 3 \| \| JCHAIN \| 2.17901E-51 \| -3.861463142 \| 4.75613E-47 \| 3 \| \| SSR4 \| 7.9892E-46 \| -1.091643564 \| 1.7438E-41 \| 3 \| \| MUC2 \| 3.23005E-26 \| -1.379618907 \| 7.05022E-22 \| 3 \| \| IGHA1 \| 1.45139E-19 \| -3.575556323 \| 3.16796E-15 \| 3 \| \| IGHG1 \| 0 \| 5.96315728 \| 0 \| 4 \| \| IGHG2 \| 0 \| 5.579664733 \| 0 \| 4 \| \| IGHGP \| 0 \| 4.574724368 \| 0 \| 4 \| \| IGHG3 \| 0 \| 4.474390712 \| 0 \| 4 \| \| IGLC3 \| 0 \| 3.691232248 \| 0 \| 4 \| \| IGLC2 \| 0 \| 3.66372227 \| 0 \| 4 \| \| IGHG4 \| 0 \| 3.437336923 \| 0 \| 4 \| \| IGHA2 \| 0 \| 3.40462181 \| 0 \| 4 \| \| MZB1 \| 0 \| 3.173004469 \| 0 \| 4 \| \| IGKC \| 0 \| 2.969037674 \| 0 \| 4 \| \| IGHA1 \| 0 \| 2.7473899 \| 0 \| 4 \| \| JCHAIN \| 0 \| 2.64489449 \| 0 \| 4 \| \| SSR4 \| 0 \| 2.396688162 \| 0 \| 4 \| \| DERL3 \| 0 \| 2.277197667 \| 0 \| 4 \| \| IGKJ5 \| 0 \| 2.135047148 \| 0 \| 4 \| \| XBP1 \| 0 \| 2.109272243 \| 0 \| 4 \| \| IGLV3-1 \| 0 \| 2.049097692 \| 0 \| 4 \| \| FKBP11 \| 0 \| 1.968975491 \| 0 \| 4 \| \| SEC11C \| 0 \| 1.824946618 \| 0 \| 4 \| \| TNFRSF17 \| 0 \| 1.711758128 \| 0 \| 4 \| \| HERPUD1 \| 0 \| 1.649189188 \| 0 \| 4 \| \| PRDX4 \| 0 \| 1.637373757 \| 0 \| 4 \| \| IGKV4-1 \| 0 \| 1.622649895 \| 0 \| 4 \| \| JSRP1 \| 0 \| 1.431625002 \| 0 \| 4 \| \| HSP90B1 \| 0 \| 1.35081065 \| 0 \| 4 \| \| FKBP2 \| 0 \| 1.317655994 \| 0 \| 4 \| \| PIM2 \| 0 \| 1.23039879 \| 0 \| 4 \| \| SDF2L1 \| 0 \| 1.217370478 \| 0 \| 4 \| \| CD79A \| 0 \| 1.129887406 \| 0 \| 4 \| \| SSR3 \| 0 \| 1.08880221 \| 0 \| 4 \| \| POU2AF1 \| 0 \| 1.067388855 \| 0 \| 4 \| \| UBE2J1 \| 0 \| 1.04165133 \| 0 \| 4 \| \| SPCS3 \| 0 \| 1.024866839 \| 0 \| 4 \| \| RPS4X \| 0 \| -1.000737914 \| 0 \| 4 \| \| RPS11 \| 0 \| -1.015777987 \| 0 \| 4 \| \| RPS9 \| 0 \| -1.019369742 \| 0 \| 4 \| \| RPL6 \| 0 \| -1.074451683 \| 0 \| 4 \| \| PFDN5 \| 0 \| -1.103058536 \| 0 \| 4 \| \| RPL24 \| 0 \| -1.104617048 \| 0 \| 4 \| \| RPS28 \| 0 \| -1.11734699 \| 0 \| 4 \| \| RPS15 \| 0 \| -1.143141354 \| 0 \| 4 \| \| RPL8 \| 0 \| -1.159501915 \| 0 \| 4 \| \| TPT1 \| 0 \| -1.16495978 \| 0 \| 4 \| \| RPS8 \| 0 \| -1.171304759 \| 0 \| 4 \| \| RPL35A \| 0 \| -1.190103061 \| 0 \| 4 \| \| H3F3B \| 0 \| -1.19859379 \| 0 \| 4 \| \| MALAT1 \| 0 \| -1.202644319 \| 0 \| 4 \| \| RPL29 \| 0 \| -1.208514663 \| 0 \| 4 \| \| UBA52 \| 0 \| -1.213732016 \| 0 \| 4 \| \| UQCRB \| 0 \| -1.215083024 \| 0 \| 4 \| \| RPL5 \| 0 \| -1.216875303 \| 0 \| 4 \| \| RPS19 \| 0 \| -1.224320104 \| 0 \| 4 \| \| RPS16 \| 0 \| -1.238729848 \| 0 \| 4 \| \| NACA \| 0 \| -1.258762722 \| 0 \| 4 \| \| RPL35 \| 0 \| -1.261342083 \| 0 \| 4 \| \| RPL14 \| 0 \| -1.306568256 \| 0 \| 4 \| \| RPL37A \| 0 \| -1.313426285 \| 0 \| 4 \| \| RPL27 \| 0 \| -1.317966613 \| 0 \| 4 \| \| RPL34 \| 0 \| -1.32774426 \| 0 \| 4 \| \| RPL7 \| 0 \| -1.341436753 \| 0 \| 4 \| \| RPS7 \| 0 \| -1.344740382 \| 0 \| 4 \| \| ATP5F1E \| 0 \| -1.346038088 \| 0 \| 4 \| \| DDX5 \| 0 \| -1.349484231 \| 0 \| 4 \| \| RPL21 \| 0 \| -1.352632419 \| 0 \| 4 \| \| RPL23A \| 0 \| -1.354826388 \| 0 \| 4 \| \| RPS14 \| 0 \| -1.370945898 \| 0 \| 4 \| \| RPS15A \| 0 \| -1.372037136 \| 0 \| 4 \| \| EEF1A1 \| 0 \| -1.375323089 \| 0 \| 4 \| \| SRP14 \| 0 \| -1.387447431 \| 0 \| 4 \| \| RPL27A \| 0 \| -1.394250524 \| 0 \| 4 \| \| RPS13 \| 0 \| -1.407787315 \| 0 \| 4 \| \| RPSA \| 0 \| -1.408291844 \| 0 \| 4 \| \| PRRC2C \| 0 \| -1.410729974 \| 0 \| 4 \| \| HSP90AB1 \| 0 \| -1.423611596 \| 0 \| 4 \| \| RPS27A \| 0 \| -1.431166817 \| 0 \| 4 \| \| RPL13 \| 0 \| -1.432661773 \| 0 \| 4 \| \| RPL9 \| 0 \| -1.442635177 \| 0 \| 4 \| \| CALM1 \| 0 \| -1.443138704 \| 0 \| 4 \| \| TOMM7 \| 0 \| -1.454576079 \| 0 \| 4 \| \| RPL19 \| 0 \| -1.475924461 \| 0 \| 4 \| \| RPS2 \| 0 \| -1.482861803 \| 0 \| 4 \| \| HLA-E \| 0 \| -1.506925641 \| 0 \| 4 \| \| RPS3A \| 0 \| -1.50863705 \| 0 \| 4 \| \| RPL10A \| 0 \| -1.51494571 \| 0 \| 4 \| \| RPL13A \| 0 \| -1.521702993 \| 0 \| 4 \| \| RPS18 \| 0 \| -1.527390725 \| 0 \| 4 \| \| RPLP2 \| 0 \| -1.530738505 \| 0 \| 4 \| \| BTF3 \| 0 \| -1.535227484 \| 0 \| 4 \| \| RPL11 \| 0 \| -1.540247754 \| 0 \| 4 \| \| RPL23 \| 0 \| -1.542758303 \| 0 \| 4 \| \| MYL6 \| 0 \| -1.544047781 \| 0 \| 4 \| \| RPS23 \| 0 \| -1.551218716 \| 0 \| 4 \| \| RPS6 \| 0 \| -1.55539748 \| 0 \| 4 \| \| RPS20 \| 0 \| -1.558412045 \| 0 \| 4 \| \| CLIC1 \| 0 \| -1.559603235 \| 0 \| 4 \| \| RPL36 \| 0 \| -1.567098729 \| 0 \| 4 \| \| RPS3 \| 0 \| -1.576967526 \| 0 \| 4 \| \| TPM3 \| 0 \| -1.617998874 \| 0 \| 4 \| \| RPL28 \| 0 \| -1.626651239 \| 0 \| 4 \| \| CIB1 \| 0 \| -1.652801174 \| 0 \| 4 \| \| HMGB1 \| 0 \| -1.653627603 \| 0 \| 4 \| \| RPS21 \| 0 \| -1.656343984 \| 0 \| 4 \| \| RPL32 \| 0 \| -1.66011466 \| 0 \| 4 \| \| RPS29 \| 0 \| -1.670905658 \| 0 \| 4 \| \| RPL38 \| 0 \| -1.685567237 \| 0 \| 4 \| \| RPL12 \| 0 \| -1.685672169 \| 0 \| 4 \| \| RPL37 \| 0 \| -1.71557284 \| 0 \| 4 \| \| TPI1 \| 0 \| -1.722724191 \| 0 \| 4 \| \| RPL30 \| 0 \| -1.724932797 \| 0 \| 4 \| \| RPL31 \| 0 \| -1.771791323 \| 0 \| 4 \| \| RPS12 \| 0 \| -1.785069945 \| 0 \| 4 \| \| RPS24 \| 0 \| -1.791154458 \| 0 \| 4 \| \| RPS27 \| 0 \| -1.794488571 \| 0 \| 4 \| \| YWHAB \| 0 \| -1.819746087 \| 0 \| 4 \| \| MYH9 \| 0 \| -1.826619196 \| 0 \| 4 \| \| DEK \| 0 \| -1.871975893 \| 0 \| 4 \| \| PPDPF \| 0 \| -1.962096187 \| 0 \| 4 \| \| PTMA \| 0 \| -1.976418601 \| 0 \| 4 \| \| ID2 \| 0 \| -2.016477863 \| 0 \| 4 \| \| GAPDH \| 0 \| -2.123259111 \| 0 \| 4 \| \| PFN1 \| 0 \| -2.246554355 \| 0 \| 4 \| \| MYL12A \| 0 \| -2.266263479 \| 0 \| 4 \| \| MYL12B \| 0 \| -2.337517546 \| 0 \| 4 \| \| FTH1 \| 0 \| -2.340637128 \| 0 \| 4 \| \| REL \| 0 \| -2.364041989 \| 0 \| 4 \| \| S100A11 \| 0 \| -2.394394321 \| 0 \| 4 \| \| ACTG1 \| 0 \| -2.460834008 \| 0 \| 4 \| \| HLA-A \| 0 \| -2.555928814 \| 0 \| 4 \| \| TMSB4X \| 0 \| -2.619308111 \| 0 \| 4 \| \| SH3BGRL3 \| 0 \| -2.627156591 \| 0 \| 4 \| \| BTG1 \| 0 \| -2.725201182 \| 0 \| 4 \| \| TMSB10 \| 0 \| -2.813447434 \| 0 \| 4 \| \| LGALS3 \| 0 \| -2.949654843 \| 0 \| 4 \| \| ACTB \| 0 \| -3.133806461 \| 0 \| 4 \| \| S100A10 \| 0 \| -3.288631469 \| 0 \| 4 \| \| S100A6 \| 0 \| -4.340895842 \| 0 \| 4 \| \| DYNLL1 \| 2.3778E-305 \| -1.372802974 \| 5.19E-301 \| 4 \| \| COX6C \| 4.9914E-305 \| -1.453879486 \| 1.0895E-300 \| 4 \| \| EIF1 \| 7.6413E-304 \| -1.14576892 \| 1.6679E-299 \| 4 \| \| ENO1 \| 2.0477E-297 \| -1.739384325 \| 4.4694E-293 \| 4 \| \| RACK1 \| 3.7109E-296 \| -1.159636453 \| 8.0997E-292 \| 4 \| \| KRT8 \| 8.5659E-294 \| -3.321443192 \| 1.8697E-289 \| 4 \| \| SKP1 \| 5.5884E-286 \| -1.362129272 \| 1.2198E-281 \| 4 \| \| HNRNPK \| 2.2044E-285 \| -1.380238463 \| 4.8116E-281 \| 4 \| \| COTL1 \| 2.7419E-283 \| -1.912949398 \| 5.9848E-279 \| 4 \| \| YWHAZ \| 4.4149E-279 \| -1.438604375 \| 9.6365E-275 \| 4 \| \| YBX1 \| 8.8126E-278 \| -1.290802381 \| 1.9235E-273 \| 4 \| \| SOX4 \| 1.6544E-274 \| -2.451021895 \| 3.6111E-270 \| 4 \| \| ETS2 \| 5.2088E-274 \| -1.500422935 \| 1.1369E-269 \| 4 \| \| RHOC \| 8.639E-273 \| -1.492606104 \| 1.8856E-268 \| 4 \| \| ARPC3 \| 2.9016E-272 \| -1.444908745 \| 6.3333E-268 \| 4 \| \| VMP1 \| 5.7941E-271 \| -1.760953454 \| 1.2647E-266 \| 4 \| \| ZFAS1 \| 7.0001E-271 \| -1.591051736 \| 1.5279E-266 \| 4 \| \| CDC42 \| 1.2289E-270 \| -1.411934492 \| 2.6823E-266 \| 4 \| \| FOXP1 \| 6.9587E-270 \| -1.46791807 \| 1.5189E-265 \| 4 \| \| SOD1 \| 8.4457E-269 \| -1.550498083 \| 1.8434E-264 \| 4 \| \| HINT1 \| 2.1449E-266 \| -1.134151626 \| 4.6817E-262 \| 4 \| \| COMMD6 \| 4.0453E-266 \| -1.533442802 \| 8.8297E-262 \| 4 \| \| IFI27 \| 1.7441E-260 \| -3.232891345 \| 3.8069E-256 \| 4 \| \| SDCBP \| 3.5592E-259 \| -1.560915006 \| 7.7686E-255 \| 4 \| \| LGALS4 \| 4.6695E-259 \| -3.191447542 \| 1.0192E-254 \| 4 \| \| ELF3 \| 5.0951E-259 \| -2.627499968 \| 1.1121E-254 \| 4 \| \| LGALS3BP \| 2.458E-258 \| -1.668703899 \| 5.3651E-254 \| 4 \| \| CAPZB \| 1.9213E-257 \| -1.400505632 \| 4.1936E-253 \| 4 \| \| ARPC2 \| 2.4677E-256 \| -1.321002043 \| 5.3864E-252 \| 4 \| \| WSB1 \| 1.0206E-251 \| -1.486175792 \| 2.2277E-247 \| 4 \| \| EPCAM \| 1.0586E-250 \| -2.341594338 \| 2.3107E-246 \| 4 \| \| KRT19 \| 1.7478E-248 \| -3.210904639 \| 3.8149E-244 \| 4 \| \| CLDN4 \| 3.8824E-248 \| -2.543801156 \| 8.4742E-244 \| 4 \| \| TUBA1B \| 7.4728E-248 \| -1.74596662 \| 1.6311E-243 \| 4 \| \| SAT1 \| 8.9278E-248 \| -1.605372666 \| 1.9487E-243 \| 4 \| \| KLF4 \| 4.4518E-247 \| -1.681715454 \| 9.717E-243 \| 4 \| \| KRT18 \| 3.6945E-246 \| -3.261920479 \| 8.064E-242 \| 4 \| \| ACTR2 \| 4.8027E-245 \| -1.498559094 \| 1.0483E-240 \| 4 \| \| APP \| 2.8071E-244 \| -1.447962169 \| 6.1272E-240 \| 4 \| \| HES1 \| 3.7502E-244 \| -1.995356475 \| 8.1855E-240 \| 4 \| \| TPM1 \| 1.6111E-243 \| -1.647381508 \| 3.5167E-239 \| 4 \| \| SLC25A3 \| 4.3914E-243 \| -1.30182702 \| 9.5851E-239 \| 4 \| \| IRS2 \| 4.2339E-242 \| -1.32039778 \| 9.2413E-238 \| 4 \| \| DNAJA1 \| 2.5206E-241 \| -1.830915041 \| 5.5018E-237 \| 4 \| \| ANXA1 \| 1.2951E-240 \| -2.531334147 \| 2.8269E-236 \| 4 \| \| CAMK2N1 \| 5.044E-237 \| -1.568388409 \| 1.101E-232 \| 4 \| \| CEBPD \| 2.0674E-236 \| -1.66141476 \| 4.5125E-232 \| 4 \| \| PHGR1 \| 2.8031E-236 \| -5.164007611 \| 6.1184E-232 \| 4 \| \| H2AFY \| 1.2389E-234 \| -1.303897155 \| 2.7041E-230 \| 4 \| \| LDHA \| 1.6652E-234 \| -1.640509451 \| 3.6346E-230 \| 4 \| \| CLDN3 \| 4.1479E-234 \| -2.3341708 \| 9.0537E-230 \| 4 \| \| HNRNPA2B1 \| 5.3878E-234 \| -1.372071085 \| 1.176E-229 \| 4 \| \| HSPA8 \| 8.4862E-234 \| -1.345560754 \| 1.8523E-229 \| 4 \| \| JPT1 \| 1.2621E-233 \| -1.476294481 \| 2.7547E-229 \| 4 \| \| CDC42EP5 \| 1.7156E-233 \| -1.685114469 \| 3.7447E-229 \| 4 \| \| GLUL \| 3.2043E-233 \| -1.724687324 \| 6.9941E-229 \| 4 \| \| PSMA7 \| 4.4347E-233 \| -1.364192143 \| 9.6796E-229 \| 4 \| \| GSN \| 7.5216E-232 \| -1.97225586 \| 1.6417E-227 \| 4 \| \| IFITM3 \| 1.0578E-231 \| -1.860052096 \| 2.3088E-227 \| 4 \| \| RTN4 \| 4.5329E-231 \| -1.388423379 \| 9.8939E-227 \| 4 \| \| PABPC1 \| 5.1477E-231 \| -1.508939683 \| 1.1236E-226 \| 4 \| \| CEMIP2 \| 1.5596E-230 \| -1.367465229 \| 3.4042E-226 \| 4 \| \| MT2A \| 1.6804E-230 \| -2.371247557 \| 3.6678E-226 \| 4 \| \| TAGLN2 \| 1.723E-230 \| -1.386148766 \| 3.7608E-226 \| 4 \| \| RAC1 \| 3.2811E-228 \| -1.590067633 \| 7.1618E-224 \| 4 \| \| S100A4 \| 7.1343E-228 \| -2.750889561 \| 1.5572E-223 \| 4 \| \| FYB1 \| 1.1929E-227 \| -1.974679591 \| 2.6038E-223 \| 4 \| \| SRSF3 \| 2.3988E-227 \| -1.235426059 \| 5.236E-223 \| 4 \| \| LPP \| 3.1335E-227 \| -1.304470103 \| 6.8395E-223 \| 4 \| \| SAP18 \| 4.3994E-227 \| -1.263610377 \| 9.6026E-223 \| 4 \| \| ARGLU1 \| 9.681E-224 \| -1.514466258 \| 2.1131E-219 \| 4 \| \| TSPAN8 \| 1.9279E-223 \| -2.612700863 \| 4.2079E-219 \| 4 \| \| ST14 \| 4.9916E-223 \| -1.347295085 \| 1.0895E-218 \| 4 \| \| EFHD2 \| 9.7095E-221 \| -1.4933645 \| 2.1193E-216 \| 4 \| \| NAP1L1 \| 8.8385E-220 \| -1.495295544 \| 1.9292E-215 \| 4 \| \| RPL4 \| 1.0883E-219 \| -1.099464151 \| 2.3753E-215 \| 4 \| \| RPS26 \| 2.7604E-219 \| -1.200634971 \| 6.0252E-215 \| 4 \| \| APLP2 \| 4.1151E-219 \| -1.337576034 \| 8.982E-215 \| 4 \| \| IQGAP1 \| 1.7081E-218 \| -1.334725189 \| 3.7283E-214 \| 4 \| \| KIF5B \| 8.0888E-218 \| -1.385152491 \| 1.7655E-213 \| 4 \| \| SMIM22 \| 2.8721E-217 \| -1.818657116 \| 6.2689E-213 \| 4 \| \| DSP \| 1.7365E-216 \| -1.624531043 \| 3.7903E-212 \| 4 \| \| ZFP36L1 \| 2.0666E-215 \| -1.558129208 \| 4.5107E-211 \| 4 \| \| TSC22D1 \| 5.9718E-214 \| -1.498516092 \| 1.3035E-209 \| 4 \| \| AGR2 \| 9.3581E-213 \| -2.951719191 \| 2.0426E-208 \| 4 \| \| COX5B \| 3.3807E-212 \| -1.55022556 \| 7.379E-208 \| 4 \| \| CTNND1 \| 9.461E-212 \| -1.082769599 \| 2.065E-207 \| 4 \| \| TMEM54 \| 1.5622E-211 \| -1.796593372 \| 3.4098E-207 \| 4 \| \| LAPTM5 \| 1.8479E-211 \| -2.066189924 \| 4.0334E-207 \| 4 \| \| SERPINB6 \| 1.3245E-210 \| -1.097169581 \| 2.8909E-206 \| 4 \| \| CCNI \| 1.4713E-210 \| -1.113599322 \| 3.2113E-206 \| 4 \| \| PKM \| 1.4069E-209 \| -1.511807288 \| 3.0708E-205 \| 4 \| \| RPS10 \| 3.8261E-207 \| -1.188393787 \| 8.3513E-203 \| 4 \| \| HNRNPU \| 4.4737E-207 \| -1.353751466 \| 9.7648E-203 \| 4 \| \| CD52 \| 1.4903E-206 \| -2.431757032 \| 3.2528E-202 \| 4 \| \| NCL \| 1.5337E-206 \| -1.236328433 \| 3.3475E-202 \| 4 \| \| CAP1 \| 1.9975E-206 \| -1.365097799 \| 4.36E-202 \| 4 \| \| AES \| 1.5914E-205 \| -1.308051213 \| 3.4735E-201 \| 4 \| \| CSTB \| 2.2744E-205 \| -1.582443718 \| 4.9644E-201 \| 4 \| \| TMEM176B \| 3.5447E-204 \| -1.376272126 \| 7.737E-200 \| 4 \| \| FXYD3 \| 9.6471E-204 \| -2.840569606 \| 2.1057E-199 \| 4 \| \| IVNS1ABP \| 1.9018E-202 \| -1.437284485 \| 4.1511E-198 \| 4 \| \| PTBP3 \| 2.0251E-202 \| -1.258442983 \| 4.4201E-198 \| 4 \| \| SERBP1 \| 3.3998E-202 \| -1.290372265 \| 7.4208E-198 \| 4 \| \| TSPO \| 5.1764E-202 \| -1.469268067 \| 1.1299E-197 \| 4 \| \| ACTN4 \| 8.5113E-202 \| -1.271193979 \| 1.8578E-197 \| 4 \| \| MARCKSL1 \| 1.422E-201 \| -1.466506948 \| 3.1038E-197 \| 4 \| \| NUCKS1 \| 8.9517E-200 \| -1.416255851 \| 1.9539E-195 \| 4 \| \| KLF5 \| 1.2484E-199 \| -1.527171038 \| 2.7248E-195 \| 4 \| \| ANXA2 \| 1.5535E-199 \| -1.807260553 \| 3.3908E-195 \| 4 \| \| SPTBN1 \| 7.6184E-199 \| -1.562649574 \| 1.6629E-194 \| 4 \| \| FAM3D \| 1.0849E-198 \| -1.877786938 \| 2.368E-194 \| 4 \| \| CD9 \| 1.4171E-196 \| -1.917566018 \| 3.0931E-192 \| 4 \| \| AKAP13 \| 8.5521E-196 \| -1.397248644 \| 1.8667E-191 \| 4 \| \| S100A14 \| 1.6828E-195 \| -1.491381949 \| 3.6729E-191 \| 4 \| \| SRI \| 3.3382E-195 \| -1.665339409 \| 7.2864E-191 \| 4 \| \| TUBA1A \| 1.2608E-194 \| -1.568541704 \| 2.752E-190 \| 4 \| \| ANXA4 \| 1.2678E-194 \| -1.112139838 \| 2.7672E-190 \| 4 \| \| CD24 \| 1.1061E-193 \| -1.9790505 \| 2.4143E-189 \| 4 \| \| ARL4A \| 1.6524E-192 \| -1.238304798 \| 3.6066E-188 \| 4 \| \| ATP1B1 \| 5.0793E-191 \| -1.83860575 \| 1.1087E-186 \| 4 \| \| TCF7L2 \| 1.3738E-190 \| -1.102139112 \| 2.9985E-186 \| 4 \| \| LAMP1 \| 3.9142E-190 \| -1.247298688 \| 8.5436E-186 \| 4 \| \| CAST \| 2.4232E-189 \| -1.323559988 \| 5.2891E-185 \| 4 \| \| NPM1 \| 3.8204E-189 \| -1.281842654 \| 8.3388E-185 \| 4 \| \| ACTR3 \| 6.2293E-189 \| -1.319351972 \| 1.3597E-184 \| 4 \| \| RHOA \| 6.838E-189 \| -1.251402942 \| 1.4925E-184 \| 4 \| \| SLC44A4 \| 3.1651E-188 \| -1.4257697 \| 6.9085E-184 \| 4 \| \| IL7R \| 8.5101E-188 \| -3.104527602 \| 1.8575E-183 \| 4 \| \| EIF4G2 \| 1.4592E-187 \| -1.185167802 \| 3.185E-183 \| 4 \| \| TGOLN2 \| 7.1157E-187 \| -1.245580611 \| 1.5531E-182 \| 4 \| \| CD3D \| 6.6592E-186 \| -2.19543162 \| 1.4535E-181 \| 4 \| \| MUC13 \| 1.0726E-185 \| -1.729643847 \| 2.3413E-181 \| 4 \| \| TMEM176A \| 4.1735E-185 \| -1.167608628 \| 9.1094E-181 \| 4 \| \| NBL1 \| 6.6204E-185 \| -1.087460815 \| 1.445E-180 \| 4 \| \| SRSF7 \| 4.7608E-184 \| -1.281260157 \| 1.0391E-179 \| 4 \| \| WDR1 \| 7.0065E-184 \| -1.096497968 \| 1.5293E-179 \| 4 \| \| RAB11FIP1 \| 1.5545E-183 \| -1.494628965 \| 3.393E-179 \| 4 \| \| ANXA11 \| 3.4615E-183 \| -1.270536234 \| 7.5555E-179 \| 4 \| \| CRYBG1 \| 1.1816E-182 \| -1.125117912 \| 2.5792E-178 \| 4 \| \| CDH17 \| 1.2205E-182 \| -1.319543729 \| 2.6639E-178 \| 4 \| \| TXN \| 5.3016E-182 \| -1.667399908 \| 1.1572E-177 \| 4 \| \| CD2 \| 4.0929E-181 \| -2.102272342 \| 8.9335E-177 \| 4 \| \| RPS6KA3 \| 9.3744E-181 \| -1.057861006 \| 2.0461E-176 \| 4 \| \| FOSL2 \| 1.0158E-179 \| -1.272644778 \| 2.2172E-175 \| 4 \| \| PTGER4 \| 1.1025E-178 \| -1.143203252 \| 2.4065E-174 \| 4 \| \| SLC25A5 \| 5.5631E-178 \| -1.414356188 \| 1.2143E-173 \| 4 \| \| COX6B1 \| 2.5362E-177 \| -1.183804323 \| 5.5358E-173 \| 4 \| \| PIGR \| 4.7959E-177 \| -4.496000479 \| 1.0468E-172 \| 4 \| \| COX7B \| 2.8795E-176 \| -1.307845264 \| 6.2851E-172 \| 4 \| \| DSG2 \| 1.4784E-175 \| -1.246074428 \| 3.2268E-171 \| 4 \| \| ELOB \| 1.3972E-173 \| -1.238585456 \| 3.0496E-169 \| 4 \| \| SON \| 1.0979E-172 \| -1.098592853 \| 2.3963E-168 \| 4 \| \| METRNL \| 2.401E-172 \| -1.088397594 \| 5.2407E-168 \| 4 \| \| SERPINB1 \| 6.3587E-171 \| -1.196772915 \| 1.3879E-166 \| 4 \| \| TUBB4B \| 1.0977E-170 \| -1.187940213 \| 2.3959E-166 \| 4 \| \| PIK3R1 \| 1.4898E-170 \| -1.471018466 \| 3.2517E-166 \| 4 \| \| SCP2 \| 2.4297E-170 \| -1.190242341 \| 5.3032E-166 \| 4 \| \| TFF3 \| 1.1566E-169 \| -4.081972805 \| 2.5246E-165 \| 4 \| \| TNFRSF1B \| 4.5042E-169 \| -1.276378961 \| 9.8313E-165 \| 4 \| \| GPX2 \| 1.113E-168 \| -1.791873004 \| 2.4294E-164 \| 4 \| \| SPATS2L \| 4.6766E-168 \| -1.04636158 \| 1.0208E-163 \| 4 \| \| SPINT1 \| 5.1606E-168 \| -1.091309812 \| 1.1264E-163 \| 4 \| \| NT5C \| 7.8681E-168 \| -1.024057014 \| 1.7174E-163 \| 4 \| \| ATP1B3 \| 1.6549E-167 \| -1.612192153 \| 3.6121E-163 \| 4 \| \| RPS25 \| 5.0792E-167 \| -1.213512233 \| 1.1086E-162 \| 4 \| \| ITM2B \| 6.6139E-166 \| -1.330425656 \| 1.4436E-161 \| 4 \| \| ARL4C \| 1.0829E-165 \| -1.666022466 \| 2.3637E-161 \| 4 \| \| TRIR \| 1.7302E-165 \| -1.12606792 \| 3.7766E-161 \| 4 \| \| HNRNPF \| 3.1897E-165 \| -1.16088717 \| 6.9621E-161 \| 4 \| \| EML4 \| 1.099E-164 \| -1.145244663 \| 2.3987E-160 \| 4 \| \| RPL39 \| 1.4947E-164 \| -1.432495848 \| 3.2625E-160 \| 4 \| \| EIF4A2 \| 1.5945E-164 \| -1.104867264 \| 3.4803E-160 \| 4 \| \| PERP \| 4.8493E-164 \| -1.510230183 \| 1.0585E-159 \| 4 \| \| TRAC \| 7.0764E-164 \| -1.946314522 \| 1.5446E-159 \| 4 \| \| SELENOP \| 8.0427E-164 \| -2.508171298 \| 1.7555E-159 \| 4 \| \| DBI \| 9.9479E-164 \| -1.312740073 \| 2.1713E-159 \| 4 \| \| LIMA1 \| 2.6155E-163 \| -1.271722982 \| 5.7089E-159 \| 4 \| \| TOB1 \| 4.0905E-163 \| -1.242296626 \| 8.9283E-159 \| 4 \| \| CXCR4 \| 8.1416E-163 \| -2.85574086 \| 1.7771E-158 \| 4 \| \| VASP \| 1.2681E-162 \| -1.014291417 \| 2.7678E-158 \| 4 \| \| MBNL1 \| 1.1184E-161 \| -1.238894637 \| 2.441E-157 \| 4 \| \| MUC12 \| 4.459E-161 \| -2.127411109 \| 9.7328E-157 \| 4 \| \| RPL22 \| 4.7869E-161 \| -1.206119514 \| 1.0448E-156 \| 4 \| \| ZFP36L2 \| 6.603E-161 \| -1.03005097 \| 1.4412E-156 \| 4 \| \| TSPAN1 \| 2.1387E-159 \| -2.305916049 \| 4.6681E-155 \| 4 \| \| PTMS \| 1.0933E-158 \| -1.253668695 \| 2.3863E-154 \| 4 \| \| ARL6IP5 \| 3.9519E-158 \| -1.172040792 \| 8.6258E-154 \| 4 \| \| IER5 \| 1.0712E-157 \| -1.378137675 \| 2.3381E-153 \| 4 \| \| KLF3 \| 1.3652E-157 \| -1.055318299 \| 2.9798E-153 \| 4 \| \| DYNLT1 \| 1.3778E-157 \| -1.050392415 \| 3.0074E-153 \| 4 \| \| PTPRC \| 1.3843E-157 \| -2.27103065 \| 3.0215E-153 \| 4 \| \| 7-Sep \| 4.1678E-157 \| -1.285736179 \| 9.097E-153 \| 4 \| \| CSNK1A1 \| 4.2327E-157 \| -1.132273083 \| 9.2387E-153 \| 4 \| \| CTNNA1 \| 4.3641E-157 \| -1.006691056 \| 9.5254E-153 \| 4 \| \| ZBTB7A \| 7.0923E-157 \| -1.189379101 \| 1.548E-152 \| 4 \| \| PTGES3 \| 1.8984E-156 \| -1.16276614 \| 4.1436E-152 \| 4 \| \| LCP1 \| 4.1E-156 \| -1.81579186 \| 8.9491E-152 \| 4 \| \| PAK2 \| 4.8919E-154 \| -1.137944264 \| 1.0677E-149 \| 4 \| \| LPIN2 \| 4.6536E-153 \| -1.035583669 \| 1.0157E-148 \| 4 \| \| SNX6 \| 8.4596E-153 \| -1.054378168 \| 1.8465E-148 \| 4 \| \| PRR13 \| 7.7306E-152 \| -1.058449877 \| 1.6874E-147 \| 4 \| \| MTPN \| 9.0784E-152 \| -1.065200467 \| 1.9815E-147 \| 4 \| \| TMA7 \| 2.8895E-151 \| -1.095194979 \| 6.307E-147 \| 4 \| \| PGK1 \| 6.3754E-151 \| -1.293562666 \| 1.3916E-146 \| 4 \| \| PLAC8 \| 4.5372E-150 \| -1.902862394 \| 9.9033E-146 \| 4 \| \| JAK1 \| 1.7436E-149 \| -1.23174366 \| 3.8057E-145 \| 4 \| \| DST \| 3.4481E-148 \| -1.380866725 \| 7.5262E-144 \| 4 \| \| HNRNPA3 \| 3.7315E-148 \| -1.155361061 \| 8.1447E-144 \| 4 \| \| NDUFS5 \| 7.8975E-146 \| -1.14337358 \| 1.7238E-141 \| 4 \| \| HADHA \| 1.2909E-145 \| -1.033008026 \| 2.8176E-141 \| 4 \| \| GCC2 \| 3.7033E-145 \| -1.291648175 \| 8.0831E-141 \| 4 \| \| SELENOW \| 4.7975E-145 \| -1.075815579 \| 1.0471E-140 \| 4 \| \| PHLDA1 \| 7.8684E-145 \| -1.199549925 \| 1.7174E-140 \| 4 \| \| YBX3 \| 1.607E-144 \| -1.277708265 \| 3.5076E-140 \| 4 \| \| RAN \| 2.0467E-144 \| -1.216513026 \| 4.4674E-140 \| 4 \| \| CCT6A \| 2.6255E-144 \| -1.038925058 \| 5.7306E-140 \| 4 \| \| COX4I1 \| 4.2136E-144 \| -1.20704893 \| 9.197E-140 \| 4 \| \| JUNB \| 9.8611E-144 \| -1.199248213 \| 2.1524E-139 \| 4 \| \| DIAPH1 \| 8.0585E-143 \| -1.000327078 \| 1.7589E-138 \| 4 \| \| AMD1 \| 1.2459E-142 \| -1.263564373 \| 2.7194E-138 \| 4 \| \| FRYL \| 1.9774E-142 \| -1.225477442 \| 4.316E-138 \| 4 \| \| PCBP1 \| 7.6542E-141 \| -1.008050274 \| 1.6707E-136 \| 4 \| \| LUC7L3 \| 1.6888E-140 \| -1.211027794 \| 3.6862E-136 \| 4 \| \| CAPG \| 3.7264E-139 \| -1.17186941 \| 8.1335E-135 \| 4 \| \| ARHGDIB \| 2.9445E-138 \| -1.797150798 \| 6.427E-134 \| 4 \| \| IER5L \| 3.4644E-138 \| -1.246640364 \| 7.5617E-134 \| 4 \| \| HNRNPAB \| 3.7688E-138 \| -1.213493343 \| 8.2261E-134 \| 4 \| \| CYCS \| 1.8786E-137 \| -1.134608611 \| 4.1004E-133 \| 4 \| \| PHLDA2 \| 6.8441E-137 \| -1.197043048 \| 1.4939E-132 \| 4 \| \| CMTM6 \| 8.609E-137 \| -1.15647739 \| 1.8791E-132 \| 4 \| \| ATP5MD \| 3.9757E-136 \| -1.087653202 \| 8.6778E-132 \| 4 \| \| ATP5F1B \| 4.6816E-136 \| -1.23568467 \| 1.0219E-131 \| 4 \| \| HCST \| 7.0369E-136 \| -1.598099716 \| 1.536E-131 \| 4 \| \| ETHE1 \| 5.568E-135 \| -1.107041313 \| 1.2153E-130 \| 4 \| \| PLEC \| 4.5105E-133 \| -1.001487281 \| 9.8451E-129 \| 4 \| \| CDC37 \| 4.8374E-133 \| -1.080689429 \| 1.0559E-128 \| 4 \| \| PPP1R12A \| 2.3992E-132 \| -1.075551856 \| 5.2368E-128 \| 4 \| \| SET \| 2.9871E-132 \| -1.164083654 \| 6.5198E-128 \| 4 \| \| WASF2 \| 3.3715E-131 \| -1.024666302 \| 7.3589E-127 \| 4 \| \| FUS \| 3.5428E-131 \| -1.02663213 \| 7.7329E-127 \| 4 \| \| TNFSF10 \| 3.7455E-131 \| -1.091929159 \| 8.1753E-127 \| 4 \| \| CHD1 \| 7.085E-131 \| -1.137815978 \| 1.5464E-126 \| 4 \| \| H2AFJ \| 2.3822E-130 \| -1.049325612 \| 5.1996E-126 \| 4 \| \| ZKSCAN1 \| 1.7351E-128 \| -1.149877747 \| 3.7872E-124 \| 4 \| \| HMGB2 \| 7.9122E-128 \| -1.327825458 \| 1.727E-123 \| 4 \| \| HSPD1 \| 8.3011E-128 \| -1.379656498 \| 1.8119E-123 \| 4 \| \| MVP \| 6.3572E-127 \| -1.053858712 \| 1.3876E-122 \| 4 \| \| ST13 \| 1.1077E-125 \| -1.054410946 \| 2.4177E-121 \| 4 \| \| SRSF11 \| 1.2557E-125 \| -1.075910905 \| 2.7409E-121 \| 4 \| \| SNRPF \| 6.2824E-125 \| -1.027426748 \| 1.3713E-120 \| 4 \| \| XRCC5 \| 1.0308E-124 \| -1.055388911 \| 2.2499E-120 \| 4 \| \| LCN2 \| 1.1096E-123 \| -2.534647251 \| 2.4219E-119 \| 4 \| \| SRRM1 \| 1.1331E-123 \| -1.066256909 \| 2.4733E-119 \| 4 \| \| UQCR10 \| 1.316E-123 \| -1.139936818 \| 2.8723E-119 \| 4 \| \| DDX21 \| 3.5652E-123 \| -1.170420616 \| 7.7817E-119 \| 4 \| \| TLN1 \| 7.9076E-122 \| -1.108545717 \| 1.726E-117 \| 4 \| \| HLA-DPB1 \| 8.6083E-121 \| -2.608615861 \| 1.8789E-116 \| 4 \| \| ATP6V0E1 \| 1.0983E-119 \| -1.054483333 \| 2.3974E-115 \| 4 \| \| COX7C \| 2.7896E-119 \| -1.013363824 \| 6.0888E-115 \| 4 \| \| VAMP8 \| 4.0466E-119 \| -1.324434209 \| 8.8325E-115 \| 4 \| \| NCOR1 \| 1.2043E-118 \| -1.090108867 \| 2.6286E-114 \| 4 \| \| SLC12A2 \| 1.3445E-118 \| -1.462040685 \| 2.9346E-114 \| 4 \| \| PRPF40A \| 1.4766E-118 \| -1.065413411 \| 3.223E-114 \| 4 \| \| SH3KBP1 \| 4.2539E-118 \| -1.034391311 \| 9.2851E-114 \| 4 \| \| CD164 \| 2.8351E-117 \| -1.09535138 \| 6.1881E-113 \| 4 \| \| UGP2 \| 3.2701E-117 \| -1.051385142 \| 7.1376E-113 \| 4 \| \| CREM \| 7.0555E-117 \| -1.806553229 \| 1.54E-112 \| 4 \| \| HSPE1 \| 4.8272E-116 \| -1.202951878 \| 1.0536E-111 \| 4 \| \| MIDN \| 1.0107E-115 \| -1.110461037 \| 2.2061E-111 \| 4 \| \| HMGN1 \| 3.2817E-115 \| -1.073004647 \| 7.163E-111 \| 4 \| \| SRSF2 \| 7.9422E-115 \| -1.031207258 \| 1.7336E-110 \| 4 \| \| ATP5ME \| 8.2334E-115 \| -1.083756997 \| 1.7971E-110 \| 4 \| \| TUBB \| 2.3334E-114 \| -1.203161139 \| 5.093E-110 \| 4 \| \| ANP32B \| 3.3058E-114 \| -1.087757725 \| 7.2156E-110 \| 4 \| \| TPR \| 8.7948E-114 \| -1.095133038 \| 1.9196E-109 \| 4 \| \| ARF6 \| 4.9139E-113 \| -1.048326668 \| 1.0725E-108 \| 4 \| \| GNB1 \| 5.1004E-113 \| -1.01033349 \| 1.1133E-108 \| 4 \| \| PDLIM1 \| 1.1493E-112 \| -1.138625301 \| 2.5086E-108 \| 4 \| \| H2AFZ \| 1.7571E-112 \| -1.274927356 \| 3.8353E-108 \| 4 \| \| LIMS1 \| 3.076E-112 \| -1.163585486 \| 6.7139E-108 \| 4 \| \| WNK1 \| 7.2103E-112 \| -1.065239334 \| 1.5738E-107 \| 4 \| \| RBM25 \| 1.1614E-110 \| -1.023672496 \| 2.5349E-106 \| 4 \| \| RGS10 \| 1.1994E-110 \| -1.102696579 \| 2.6178E-106 \| 4 \| \| RNF213 \| 2.6097E-110 \| -1.237438333 \| 5.6963E-106 \| 4 \| \| SNRPD2 \| 3.4992E-110 \| -1.053099846 \| 7.6376E-106 \| 4 \| \| DYNC1H1 \| 9.8344E-110 \| -1.011220907 \| 2.1465E-105 \| 4 \| \| RSRC2 \| 1.0966E-108 \| -1.048752646 \| 2.3935E-104 \| 4 \| \| LITAF \| 4.1879E-108 \| -1.340716305 \| 9.141E-104 \| 4 \| \| EIF3H \| 1.0775E-107 \| -1.021021367 \| 2.3518E-103 \| 4 \| \| HNRNPM \| 1.5869E-107 \| -1.01554595 \| 3.4637E-103 \| 4 \| \| ROCK1 \| 1.7916E-107 \| -1.063728216 \| 3.9104E-103 \| 4 \| \| HNRNPDL \| 3.0842E-107 \| -1.014695932 \| 6.7318E-103 \| 4 \| \| ARID4B \| 3.9824E-107 \| -1.089028576 \| 8.6925E-103 \| 4 \| \| ZC3HAV1 \| 5.5013E-107 \| -1.350278268 \| 1.2008E-102 \| 4 \| \| YME1L1 \| 9.3261E-107 \| -1.109648719 \| 2.0356E-102 \| 4 \| \| VPS28 \| 4.096E-106 \| -1.011214543 \| 8.9404E-102 \| 4 \| \| AREG \| 1.237E-105 \| -1.985106255 \| 2.6999E-101 \| 4 \| \| VDAC1 \| 1.7801E-105 \| -1.072930944 \| 3.8854E-101 \| 4 \| \| CD99 \| 2.6035E-105 \| -1.177872145 \| 5.6826E-101 \| 4 \| \| TST \| 8.2238E-105 \| -1.036081678 \| 1.795E-100 \| 4 \| \| FABP1 \| 4.2717E-103 \| -3.954474737 \| 9.3237E-99 \| 4 \| \| FLNA \| 5.3657E-103 \| -1.006781293 \| 1.17118E-98 \| 4 \| \| CLTB \| 7.6984E-103 \| -1.182441668 \| 1.68034E-98 \| 4 \| \| KMT2E \| 1.5244E-102 \| -1.035871641 \| 3.3274E-98 \| 4 \| \| BPTF \| 4.7297E-102 \| -1.104457924 \| 1.03236E-97 \| 4 \| \| PNRC1 \| 1.2751E-101 \| -1.283761228 \| 2.78307E-97 \| 4 \| \| BAZ1A \| 1.8395E-101 \| -1.026954278 \| 4.01503E-97 \| 4 \| \| CD69 \| 2.2145E-101 \| -2.068944077 \| 4.83368E-97 \| 4 \| \| ELF1 \| 4.2753E-101 \| -1.180424926 \| 9.33163E-97 \| 4 \| \| UBE2D3 \| 4.3052E-101 \| -1.02294677 \| 9.39696E-97 \| 4 \| \| ABRACL \| 1.2054E-100 \| -1.05442163 \| 2.63096E-96 \| 4 \| \| JMJD1C \| 2.329E-100 \| -1.003124231 \| 5.08352E-96 \| 4 \| \| KTN1 \| 2.7805E-100 \| -1.003355401 \| 6.06907E-96 \| 4 \| \| IER3 \| 4.878E-100 \| -1.410898964 \| 1.06472E-95 \| 4 \| \| CKB \| 1.0263E-99 \| -2.294321043 \| 2.24014E-95 \| 4 \| \| IRF2BP2 \| 1.3972E-99 \| -1.011009704 \| 3.04961E-95 \| 4 \| \| CORO1B \| 6.55044E-98 \| -1.000185624 \| 1.42976E-93 \| 4 \| \| BLOC1S1 \| 1.12548E-96 \| -1.078122777 \| 2.45657E-92 \| 4 \| \| NUFIP2 \| 3.92424E-96 \| -1.002410426 \| 8.56544E-92 \| 4 \| \| HLA-F \| 5.47768E-96 \| -1.012677052 \| 1.19561E-91 \| 4 \| \| EIF3E \| 1.23888E-95 \| -1.02891477 \| 2.70411E-91 \| 4 \| \| HSPH1 \| 3.4083E-95 \| -1.430565812 \| 7.4393E-91 \| 4 \| \| IDI1 \| 9.25137E-95 \| -1.120240816 \| 2.0193E-90 \| 4 \| \| LSM7 \| 1.10728E-94 \| -1.026250221 \| 2.41686E-90 \| 4 \| \| PPIG \| 1.42247E-94 \| -1.020631595 \| 3.10483E-90 \| 4 \| \| CACYBP \| 2.44648E-94 \| -1.290511355 \| 5.33994E-90 \| 4 \| \| TXNIP \| 4.24787E-94 \| -1.049919712 \| 9.27182E-90 \| 4 \| \| SOCS1 \| 1.22564E-93 \| -1.138793984 \| 2.67519E-89 \| 4 \| \| SFPQ \| 1.73765E-93 \| -1.045649371 \| 3.79277E-89 \| 4 \| \| SLC2A3 \| 6.11073E-91 \| -1.36266507 \| 1.33379E-86 \| 4 \| \| LYZ \| 1.24381E-90 \| -3.348434527 \| 2.71487E-86 \| 4 \| \| HIST1H4C \| 3.24202E-90 \| -1.405148917 \| 7.07636E-86 \| 4 \| \| CELF2 \| 4.19511E-90 \| -1.197778643 \| 9.15666E-86 \| 4 \| \| ATP5MC3 \| 1.09989E-89 \| -1.117549123 \| 2.40073E-85 \| 4 \| \| SARAF \| 1.97442E-89 \| -1.26953593 \| 4.30957E-85 \| 4 \| \| HLA-DRA \| 6.56161E-89 \| -3.812368591 \| 1.4322E-84 \| 4 \| \| PDCD4 \| 1.91882E-87 \| -1.194774667 \| 4.18821E-83 \| 4 \| \| MSN \| 3.12261E-86 \| -1.223237992 \| 6.81572E-82 \| 4 \| \| PMAIP1 \| 5.24151E-86 \| -1.177332868 \| 1.14406E-81 \| 4 \| \| MT1X \| 5.46891E-86 \| -1.266578216 \| 1.1937E-81 \| 4 \| \| CD81 \| 2.49392E-84 \| -1.336957423 \| 5.44347E-80 \| 4 \| \| HEXIM1 \| 3.26639E-84 \| -1.310461192 \| 7.12955E-80 \| 4 \| \| HIF1A \| 2.03892E-83 \| -1.046073144 \| 4.45036E-79 \| 4 \| \| G3BP2 \| 9.7064E-82 \| -1.063680058 \| 2.11862E-77 \| 4 \| \| STK17A \| 2.00266E-80 \| -1.049964319 \| 4.3712E-76 \| 4 \| \| VEGFA \| 3.10732E-80 \| -1.166881345 \| 6.78234E-76 \| 4 \| \| SERTAD1 \| 6.20472E-76 \| -1.156361909 \| 1.3543E-71 \| 4 \| \| NFKBIZ \| 7.66586E-75 \| -1.165227619 \| 1.67323E-70 \| 4 \| \| NR4A2 \| 4.74652E-72 \| -1.582368486 \| 1.03602E-67 \| 4 \| \| ISG15 \| 7.90391E-71 \| -1.288625048 \| 1.72519E-66 \| 4 \| \| TSPAN3 \| 1.15787E-70 \| -1.101177461 \| 2.52729E-66 \| 4 \| \| DSTN \| 3.59296E-70 \| -1.30303388 \| 7.84236E-66 \| 4 \| \| KDM6B \| 6.68163E-70 \| -1.12093834 \| 1.4584E-65 \| 4 \| \| DNAJB1 \| 2.06846E-68 \| -1.106031529 \| 4.51482E-64 \| 4 \| \| CD37 \| 1.15837E-67 \| -1.404157449 \| 2.52838E-63 \| 4 \| \| CEBPB \| 2.03551E-67 \| -1.272628056 \| 4.4429E-63 \| 4 \| \| RARRES3 \| 3.68981E-65 \| -1.137378834 \| 8.05375E-61 \| 4 \| \| TNFAIP3 \| 2.5702E-64 \| -1.925311897 \| 5.60998E-60 \| 4 \| \| FXYD5 \| 4.93088E-62 \| -1.027347157 \| 1.07626E-57 \| 4 \| \| GNAI2 \| 1.44818E-59 \| -1.160236691 \| 3.16094E-55 \| 4 \| \| FCGRT \| 1.57373E-54 \| -1.126814387 \| 3.43498E-50 \| 4 \| \| GRN \| 1.72786E-54 \| -1.263284289 \| 3.77141E-50 \| 4 \| \| BSG \| 6.36251E-54 \| -1.088641484 \| 1.38875E-49 \| 4 \| \| FKBP5 \| 3.78136E-52 \| -1.08295797 \| 8.25357E-48 \| 4 \| \| RPS4Y1 \| 1.14778E-48 \| -1.176291654 \| 2.50527E-44 \| 4 \| \| CD48 \| 5.53896E-47 \| -1.202093836 \| 1.20899E-42 \| 4 \| \| PPA1 \| 2.04125E-46 \| -1.01027481 \| 4.45543E-42 \| 4 \| \| SGK1 \| 1.00009E-43 \| -1.020227063 \| 2.1829E-39 \| 4 \| \| SYNE2 \| 8.77005E-43 \| -1.104064767 \| 1.91424E-38 \| 4 \| \| CD74 \| 1.38009E-41 \| -1.655596311 \| 3.01233E-37 \| 4 \| \| CD53 \| 5.08564E-39 \| -1.180143904 \| 1.11004E-34 \| 4 \| \| CNOT6L \| 2.45066E-37 \| -1.167455048 \| 5.34905E-33 \| 4 \| \| ZNF331 \| 2.93252E-37 \| -1.196862125 \| 6.40081E-33 \| 4 \| \| LDHB \| 6.48047E-35 \| -1.026091166 \| 1.41449E-30 \| 4 \| \| ID1 \| 4.76002E-34 \| -1.082208394 \| 1.03897E-29 \| 4 \| \| MARCKS \| 3.12626E-25 \| -1.112338681 \| 6.82369E-21 \| 4 \| \| STK17B \| 3.60819E-25 \| -1.175077502 \| 7.87559E-21 \| 4 \| \| SAMSN1 \| 2.06257E-21 \| -1.252257892 \| 4.50198E-17 \| 4 \| \| HLA-DPA1 \| 4.02091E-19 \| -2.358759175 \| 8.77644E-15 \| 4 \| \| CLEC2B \| 4.52729E-18 \| -1.097219434 \| 9.88172E-14 \| 4 \| \| GPR183 \| 7.30479E-18 \| -1.527687229 \| 1.59442E-13 \| 4 \| \| DUSP2 \| 1.02091E-16 \| -1.063911503 \| 2.22834E-12 \| 4 \| \| SRGN \| 8.96937E-15 \| -1.921492057 \| 1.95775E-10 \| 4 \| \| TIMP1 \| 4.73409E-13 \| -1.337287977 \| 1.03331E-08 \| 4 \| \| PSAP \| 7.32006E-12 \| -1.208003984 \| 1.59775E-07 \| 4 \| \| PLA2G2A \| 0 \| 3.682337364 \| 0 \| 5 \| \| PIGR \| 0 \| 3.491259755 \| 0 \| 5 \| \| MT1G \| 0 \| 3.022714066 \| 0 \| 5 \| \| OLFM4 \| 0 \| 2.633406232 \| 0 \| 5 \| \| CA1 \| 0 \| 2.604482963 \| 0 \| 5 \| \| LGALS4 \| 0 \| 2.57790366 \| 0 \| 5 \| \| SELENBP1 \| 0 \| 2.524141349 \| 0 \| 5 \| \| CKB \| 0 \| 2.427721 \| 0 \| 5 \| \| CA2 \| 0 \| 2.423449684 \| 0 \| 5 \| \| MT1E \| 0 \| 2.393993988 \| 0 \| 5 \| \| PCK1 \| 0 \| 2.36593477 \| 0 \| 5 \| \| UGT2B17 \| 0 \| 2.361827967 \| 0 \| 5 \| \| KRT19 \| 0 \| 2.296836548 \| 0 \| 5 \| \| MUC12 \| 0 \| 2.261653096 \| 0 \| 5 \| \| LEFTY1 \| 0 \| 2.255388054 \| 0 \| 5 \| \| GOLM1 \| 0 \| 2.213449341 \| 0 \| 5 \| \| FABP1 \| 0 \| 2.18668774 \| 0 \| 5 \| \| PHGR1 \| 0 \| 2.149664308 \| 0 \| 5 \| \| FAM3D \| 0 \| 2.138541962 \| 0 \| 5 \| \| TSPAN8 \| 0 \| 2.089986087 \| 0 \| 5 \| \| C10orf99 \| 0 \| 2.017760326 \| 0 \| 5 \| \| NXPE1 \| 0 \| 1.997033153 \| 0 \| 5 \| \| B3GNT7 \| 0 \| 1.986037024 \| 0 \| 5 \| \| CD24 \| 0 \| 1.964573122 \| 0 \| 5 \| \| LGALS3 \| 0 \| 1.934237591 \| 0 \| 5 \| \| HMGCS2 \| 0 \| 1.931136713 \| 0 \| 5 \| \| CES2 \| 0 \| 1.927418932 \| 0 \| 5 \| \| NXPE4 \| 0 \| 1.922018235 \| 0 \| 5 \| \| ELF3 \| 0 \| 1.860458881 \| 0 \| 5 \| \| LCN2 \| 0 \| 1.829634363 \| 0 \| 5 \| \| NUPR1 \| 0 \| 1.814606449 \| 0 \| 5 \| \| MUC4 \| 0 \| 1.797839888 \| 0 \| 5 \| \| SULT1B1 \| 0 \| 1.794426839 \| 0 \| 5 \| \| FXYD3 \| 0 \| 1.794265095 \| 0 \| 5 \| \| KLF5 \| 0 \| 1.792902402 \| 0 \| 5 \| \| CDHR5 \| 0 \| 1.761431457 \| 0 \| 5 \| \| ITM2C \| 0 \| 1.758195122 \| 0 \| 5 \| \| MT1M \| 0 \| 1.75526457 \| 0 \| 5 \| \| CDC42EP5 \| 0 \| 1.730648926 \| 0 \| 5 \| \| PLCE1 \| 0 \| 1.726807967 \| 0 \| 5 \| \| SLPI \| 0 \| 1.722558606 \| 0 \| 5 \| \| KRT8 \| 0 \| 1.722338064 \| 0 \| 5 \| \| HSD11B2 \| 0 \| 1.709559469 \| 0 \| 5 \| \| DMBT1 \| 0 \| 1.683588712 \| 0 \| 5 \| \| SLC44A4 \| 0 \| 1.677780107 \| 0 \| 5 \| \| TMEM54 \| 0 \| 1.661800543 \| 0 \| 5 \| \| MT1H \| 0 \| 1.651983974 \| 0 \| 5 \| \| CA12 \| 0 \| 1.65194046 \| 0 \| 5 \| \| CLDN3 \| 0 \| 1.64193092 \| 0 \| 5 \| \| ATP8B1 \| 0 \| 1.621076202 \| 0 \| 5 \| \| AMN \| 0 \| 1.620023656 \| 0 \| 5 \| \| VSIG2 \| 0 \| 1.612170236 \| 0 \| 5 \| \| TCIM \| 0 \| 1.610210874 \| 0 \| 5 \| \| CDH17 \| 0 \| 1.606882036 \| 0 \| 5 \| \| TST \| 0 \| 1.60234493 \| 0 \| 5 \| \| LIMA1 \| 0 \| 1.597923315 \| 0 \| 5 \| \| AGR2 \| 0 \| 1.593223754 \| 0 \| 5 \| \| CHCHD10 \| 0 \| 1.581626304 \| 0 \| 5 \| \| GPX2 \| 0 \| 1.571717655 \| 0 \| 5 \| \| AGR3 \| 0 \| 1.55877234 \| 0 \| 5 \| \| MAOA \| 0 \| 1.557212317 \| 0 \| 5 \| \| SLC4A4 \| 0 \| 1.550224591 \| 0 \| 5 \| \| EPCAM \| 0 \| 1.538658559 \| 0 \| 5 \| \| TFCP2L1 \| 0 \| 1.523569728 \| 0 \| 5 \| \| S100A14 \| 0 \| 1.517166181 \| 0 \| 5 \| \| B3GALT5 \| 0 \| 1.515154015 \| 0 \| 5 \| \| CDX1 \| 0 \| 1.514288581 \| 0 \| 5 \| \| HES1 \| 0 \| 1.503417027 \| 0 \| 5 \| \| SMIM22 \| 0 \| 1.496197509 \| 0 \| 5 \| \| AKR1C3 \| 0 \| 1.484819662 \| 0 \| 5 \| \| AOC1 \| 0 \| 1.481143924 \| 0 \| 5 \| \| LGALS2 \| 0 \| 1.476787302 \| 0 \| 5 \| \| ETHE1 \| 0 \| 1.476705195 \| 0 \| 5 \| \| SRI \| 0 \| 1.454523918 \| 0 \| 5 \| \| TCEA3 \| 0 \| 1.452219634 \| 0 \| 5 \| \| TMEM238 \| 0 \| 1.447685224 \| 0 \| 5 \| \| UQCRC1 \| 0 \| 1.442827054 \| 0 \| 5 \| \| GPA33 \| 0 \| 1.438295784 \| 0 \| 5 \| \| S100A6 \| 0 \| 1.438160304 \| 0 \| 5 \| \| DSP \| 0 \| 1.418044852 \| 0 \| 5 \| \| AC103702.2 \| 0 \| 1.406996223 \| 0 \| 5 \| \| ABCC3 \| 0 \| 1.397485111 \| 0 \| 5 \| \| COX5B \| 0 \| 1.396977545 \| 0 \| 5 \| \| PRAC1 \| 0 \| 1.368203763 \| 0 \| 5 \| \| PRSS3 \| 0 \| 1.355846355 \| 0 \| 5 \| \| TXN \| 0 \| 1.355496851 \| 0 \| 5 \| \| FAM162A \| 0 \| 1.346923596 \| 0 \| 5 \| \| LGALS3BP \| 0 \| 1.343439366 \| 0 \| 5 \| \| ID1 \| 0 \| 1.342674308 \| 0 \| 5 \| \| SPINK5 \| 0 \| 1.336039902 \| 0 \| 5 \| \| PPP1R1B \| 0 \| 1.333804757 \| 0 \| 5 \| \| FLNB \| 0 \| 1.329645134 \| 0 \| 5 \| \| S100A10 \| 0 \| 1.326532753 \| 0 \| 5 \| \| GPT \| 0 \| 1.324507932 \| 0 \| 5 \| \| SPINK1 \| 0 \| 1.320658275 \| 0 \| 5 \| \| ACADS \| 0 \| 1.308970598 \| 0 \| 5 \| \| PAPSS2 \| 0 \| 1.292888104 \| 0 \| 5 \| \| FRYL \| 0 \| 1.288508434 \| 0 \| 5 \| \| UQCRQ \| 0 \| 1.283269686 \| 0 \| 5 \| \| UGT2A3 \| 0 \| 1.277508645 \| 0 \| 5 \| \| TSPAN1 \| 0 \| 1.274642289 \| 0 \| 5 \| \| SLC39A5 \| 0 \| 1.267519973 \| 0 \| 5 \| \| CES3 \| 0 \| 1.267322597 \| 0 \| 5 \| \| MIR194-2HG \| 0 \| 1.263525326 \| 0 \| 5 \| \| TP53I3 \| 0 \| 1.263049255 \| 0 \| 5 \| \| SLC26A2 \| 0 \| 1.261272383 \| 0 \| 5 \| \| SI \| 0 \| 1.2459473 \| 0 \| 5 \| \| NANS \| 0 \| 1.240613678 \| 0 \| 5 \| \| CLDN4 \| 0 \| 1.238764213 \| 0 \| 5 \| \| ADH1C \| 0 \| 1.237941278 \| 0 \| 5 \| \| ATP5F1D \| 0 \| 1.231881833 \| 0 \| 5 \| \| CISD3 \| 0 \| 1.231278088 \| 0 \| 5 \| \| DSG2 \| 0 \| 1.22654848 \| 0 \| 5 \| \| ATP5ME \| 0 \| 1.226358108 \| 0 \| 5 \| \| ATP5MC3 \| 0 \| 1.220579385 \| 0 \| 5 \| \| ARL14 \| 0 \| 1.219988139 \| 0 \| 5 \| \| ERN2 \| 0 \| 1.219752102 \| 0 \| 5 \| \| CAMK2N1 \| 0 \| 1.216852761 \| 0 \| 5 \| \| RBM47 \| 0 \| 1.211978485 \| 0 \| 5 \| \| MTRNR2L12 \| 0 \| 1.206455646 \| 0 \| 5 \| \| GGT6 \| 0 \| 1.204567776 \| 0 \| 5 \| \| DSC2 \| 0 \| 1.203114501 \| 0 \| 5 \| \| SIAE \| 0 \| 1.197237825 \| 0 \| 5 \| \| SFN \| 0 \| 1.189607317 \| 0 \| 5 \| \| NDUFB7 \| 0 \| 1.187492335 \| 0 \| 5 \| \| C15orf48 \| 0 \| 1.178880317 \| 0 \| 5 \| \| MVP \| 0 \| 1.175543692 \| 0 \| 5 \| \| EPS8L3 \| 0 \| 1.172940488 \| 0 \| 5 \| \| MLXIP \| 0 \| 1.164776317 \| 0 \| 5 \| \| MTUS1 \| 0 \| 1.161671137 \| 0 \| 5 \| \| FZD5 \| 0 \| 1.158901451 \| 0 \| 5 \| \| MISP \| 0 \| 1.151344407 \| 0 \| 5 \| \| MT1F \| 0 \| 1.149555169 \| 0 \| 5 \| \| DGAT1 \| 0 \| 1.148212509 \| 0 \| 5 \| \| TRPM4 \| 0 \| 1.146549553 \| 0 \| 5 \| \| PLCB4 \| 0 \| 1.141372984 \| 0 \| 5 \| \| PKP2 \| 0 \| 1.137848976 \| 0 \| 5 \| \| MPST \| 0 \| 1.132186884 \| 0 \| 5 \| \| ST14 \| 0 \| 1.127995026 \| 0 \| 5 \| \| CYC1 \| 0 \| 1.126970498 \| 0 \| 5 \| \| PTPRF \| 0 \| 1.126551888 \| 0 \| 5 \| \| DHRS11 \| 0 \| 1.124708288 \| 0 \| 5 \| \| TLCD2 \| 0 \| 1.119352464 \| 0 \| 5 \| \| CHP2 \| 0 \| 1.118917991 \| 0 \| 5 \| \| CFTR \| 0 \| 1.114322421 \| 0 \| 5 \| \| NET1 \| 0 \| 1.11321596 \| 0 \| 5 \| \| PPDPF \| 0 \| 1.1126129 \| 0 \| 5 \| \| CDX2 \| 0 \| 1.111498471 \| 0 \| 5 \| \| MECOM \| 0 \| 1.103477794 \| 0 \| 5 \| \| PRSS8 \| 0 \| 1.101967303 \| 0 \| 5 \| \| BICDL2 \| 0 \| 1.096528875 \| 0 \| 5 \| \| CCL28 \| 0 \| 1.092269141 \| 0 \| 5 \| \| APP \| 0 \| 1.089835937 \| 0 \| 5 \| \| SNORC \| 0 \| 1.088060941 \| 0 \| 5 \| \| CTNND1 \| 0 \| 1.087661059 \| 0 \| 5 \| \| TMC5 \| 0 \| 1.08346742 \| 0 \| 5 \| \| COX6C \| 0 \| 1.082685878 \| 0 \| 5 \| \| KRT18 \| 0 \| 1.082653612 \| 0 \| 5 \| \| MYH14 \| 0 \| 1.082593043 \| 0 \| 5 \| \| PLA2G10 \| 0 \| 1.080561164 \| 0 \| 5 \| \| HNF4A \| 0 \| 1.074981787 \| 0 \| 5 \| \| COX6B1 \| 0 \| 1.071226718 \| 0 \| 5 \| \| AC254629.1 \| 0 \| 1.070143583 \| 0 \| 5 \| \| MARCKS \| 0 \| 1.067919679 \| 0 \| 5 \| \| MUC5B \| 0 \| 1.065068481 \| 0 \| 5 \| \| SQOR \| 0 \| 1.063061411 \| 0 \| 5 \| \| CD9 \| 0 \| 1.062536617 \| 0 \| 5 \| \| LLGL2 \| 0 \| 1.055757589 \| 0 \| 5 \| \| CAPN5 \| 0 \| 1.055504702 \| 0 \| 5 \| \| SEMA6A \| 0 \| 1.05358376 \| 0 \| 5 \| \| PADI2 \| 0 \| 1.052761728 \| 0 \| 5 \| \| KLF3 \| 0 \| 1.051554482 \| 0 \| 5 \| \| GOLIM4 \| 0 \| 1.04940304 \| 0 \| 5 \| \| SHROOM3 \| 0 \| 1.049193 \| 0 \| 5 \| \| SATB2 \| 0 \| 1.047129168 \| 0 \| 5 \| \| CLMN \| 0 \| 1.046132243 \| 0 \| 5 \| \| EFNB2 \| 0 \| 1.045948539 \| 0 \| 5 \| \| PARM1 \| 0 \| 1.044161966 \| 0 \| 5 \| \| MGAT4B \| 0 \| 1.044083084 \| 0 \| 5 \| \| NDUFA1 \| 0 \| 1.041334737 \| 0 \| 5 \| \| S100A16 \| 0 \| 1.040454822 \| 0 \| 5 \| \| COX7B \| 0 \| 1.039727566 \| 0 \| 5 \| \| ZBTB7A \| 0 \| 1.039723171 \| 0 \| 5 \| \| UGDH \| 0 \| 1.035341755 \| 0 \| 5 \| \| MAP2K6 \| 0 \| 1.021528294 \| 0 \| 5 \| \| RAPGEFL1 \| 0 \| 1.021296216 \| 0 \| 5 \| \| RAB25 \| 0 \| 1.021147123 \| 0 \| 5 \| \| MUC13 \| 0 \| 1.019800183 \| 0 \| 5 \| \| EHF \| 0 \| 1.018045472 \| 0 \| 5 \| \| SERINC2 \| 0 \| 1.015992191 \| 0 \| 5 \| \| CDH1 \| 0 \| 1.009138085 \| 0 \| 5 \| \| SLC26A3 \| 0 \| 1.004256577 \| 0 \| 5 \| \| TMC4 \| 0 \| 1.004250546 \| 0 \| 5 \| \| GNAI2 \| 0 \| -1.576793987 \| 0 \| 5 \| \| EMP3 \| 0 \| -1.590878854 \| 0 \| 5 \| \| CYTIP \| 0 \| -1.813949524 \| 0 \| 5 \| \| ARHGDIB \| 0 \| -2.130175125 \| 0 \| 5 \| \| RGS1 \| 0 \| -2.612384805 \| 0 \| 5 \| \| PTPRC \| 0 \| -2.649984976 \| 0 \| 5 \| \| TNFAIP3 \| 0 \| -2.729859406 \| 0 \| 5 \| \| TSC22D3 \| 0 \| -2.77416073 \| 0 \| 5 \| \| S100A4 \| 0 \| -2.991671643 \| 0 \| 5 \| \| CXCR4 \| 0 \| -3.416223496 \| 0 \| 5 \| \| VIM \| 0 \| -3.573404407 \| 0 \| 5 \| \| SRGN \| 0 \| -3.719845677 \| 0 \| 5 \| \| LGALS1 \| 8.9237E-305 \| -2.498099906 \| 1.9478E-300 \| 5 \| \| EFNA1 \| 1.4892E-304 \| 1.131212105 \| 3.2504E-300 \| 5 \| \| GMFG \| 2.3019E-301 \| -1.59070296 \| 5.0244E-297 \| 5 \| \| ATP5IF1 \| 6.4032E-301 \| 1.070193848 \| 1.3976E-296 \| 5 \| \| NAP1L1 \| 1.4272E-297 \| -1.432970756 \| 3.1152E-293 \| 5 \| \| CD48 \| 8.3633E-297 \| -1.82274506 \| 1.8255E-292 \| 5 \| \| ACADVL \| 2.6423E-291 \| 1.027238996 \| 5.7674E-287 \| 5 \| \| NRARP \| 2.911E-291 \| 1.023841881 \| 6.3537E-287 \| 5 \| \| COX5A \| 3.842E-290 \| 1.055978557 \| 8.3858E-286 \| 5 \| \| CD52 \| 9.8817E-286 \| -2.52787114 \| 2.1569E-281 \| 5 \| \| MT1X \| 2.1757E-284 \| 1.64502703 \| 4.749E-280 \| 5 \| \| ATP5MD \| 1.3618E-276 \| 1.008501264 \| 2.9724E-272 \| 5 \| \| LAPTM5 \| 6.0058E-274 \| -2.161321883 \| 1.3109E-269 \| 5 \| \| BST2 \| 1.4628E-255 \| -1.262607748 \| 3.1929E-251 \| 5 \| \| FXYD5 \| 5.2888E-255 \| -1.334433137 \| 1.1544E-250 \| 5 \| \| CD53 \| 3.5474E-253 \| -1.559055109 \| 7.7429E-249 \| 5 \| \| SOX4 \| 1.0142E-252 \| 1.089160077 \| 2.2138E-248 \| 5 \| \| KRT20 \| 4.4722E-247 \| 1.103216542 \| 9.7615E-243 \| 5 \| \| PRDX5 \| 5.646E-247 \| 1.011620365 \| 1.2324E-242 \| 5 \| \| MSN \| 9.9969E-245 \| -1.445782651 \| 2.182E-240 \| 5 \| \| LCP1 \| 1.6345E-242 \| -1.872953982 \| 3.5676E-238 \| 5 \| \| RGCC \| 1.4794E-239 \| -1.992771899 \| 3.2291E-235 \| 5 \| \| MT2A \| 1.7473E-239 \| 1.361204617 \| 3.8138E-235 \| 5 \| \| AC023157.3 \| 2.3882E-239 \| 1.182413962 \| 5.2127E-235 \| 5 \| \| STK17B \| 2.457E-238 \| -1.53142279 \| 5.3628E-234 \| 5 \| \| ISG20 \| 9.5032E-234 \| -1.345361892 \| 2.0743E-229 \| 5 \| \| PTP4A1 \| 1.8971E-232 \| 1.024650208 \| 4.1407E-228 \| 5 \| \| CD44 \| 6.3211E-229 \| -1.287963333 \| 1.3797E-224 \| 5 \| \| SMAP2 \| 7.8228E-229 \| -1.550341309 \| 1.7075E-224 \| 5 \| \| CREM \| 3.2781E-227 \| -2.013962102 \| 7.1551E-223 \| 5 \| \| FCGBP \| 4.3614E-227 \| -1.238424893 \| 9.5195E-223 \| 5 \| \| LSP1 \| 1.4812E-222 \| -1.317939842 \| 3.2331E-218 \| 5 \| \| GYPC \| 6.7423E-222 \| -1.111847187 \| 1.4716E-217 \| 5 \| \| RAC2 \| 7.2267E-221 \| -1.424448034 \| 1.5774E-216 \| 5 \| \| CD69 \| 1.2429E-219 \| -2.523023377 \| 2.7129E-215 \| 5 \| \| CD37 \| 2.2494E-216 \| -1.594370766 \| 4.9098E-212 \| 5 \| \| FYB1 \| 5.0274E-211 \| -1.857814635 \| 1.0973E-206 \| 5 \| \| DNAJA1 \| 1.717E-207 \| -1.548757774 \| 3.7477E-203 \| 5 \| \| ZNF331 \| 2.3413E-207 \| -1.943721549 \| 5.1104E-203 \| 5 \| \| DUSP2 \| 1.3326E-205 \| -1.902839185 \| 2.9087E-201 \| 5 \| \| PRDM1 \| 3.6402E-205 \| -1.412809452 \| 7.9456E-201 \| 5 \| \| GPR183 \| 7.3555E-204 \| -1.991169287 \| 1.6055E-199 \| 5 \| \| RGS2 \| 4.9862E-203 \| -1.66612346 \| 1.0883E-198 \| 5 \| \| AC058791.1 \| 7.2382E-199 \| -1.512283405 \| 1.5799E-194 \| 5 \| \| HCST \| 2.6211E-197 \| -1.684249385 \| 5.7212E-193 \| 5 \| \| ANXA1 \| 3.6829E-197 \| -2.335214364 \| 8.0386E-193 \| 5 \| \| ARL4C \| 2.3267E-196 \| -1.682584419 \| 5.0785E-192 \| 5 \| \| TUBA1A \| 2.8303E-196 \| -1.541164516 \| 6.1778E-192 \| 5 \| \| SELENOM \| 3.2575E-196 \| -1.136399953 \| 7.1102E-192 \| 5 \| \| CLEC2B \| 7.9025E-193 \| -1.477507802 \| 1.7249E-188 \| 5 \| \| HERPUD1 \| 2.6963E-192 \| -1.440890015 \| 5.8853E-188 \| 5 \| \| SAMSN1 \| 2.1081E-191 \| -1.589752303 \| 4.6014E-187 \| 5 \| \| PNRC1 \| 3.3418E-187 \| -1.247102293 \| 7.2942E-183 \| 5 \| \| GLIPR1 \| 6.4439E-186 \| -1.254083659 \| 1.4065E-181 \| 5 \| \| EVI2B \| 1.3377E-185 \| -1.093649642 \| 2.9198E-181 \| 5 \| \| IL7R \| 5.9678E-183 \| -3.076988713 \| 1.3026E-178 \| 5 \| \| HLA-DPA1 \| 3.2131E-182 \| -2.622820539 \| 7.0133E-178 \| 5 \| \| CD3D \| 1.9674E-179 \| -2.104664135 \| 4.2943E-175 \| 5 \| \| SELENOK \| 1.4515E-176 \| -1.044279211 \| 3.1681E-172 \| 5 \| \| RGS10 \| 4.4852E-176 \| -1.15672523 \| 9.7899E-172 \| 5 \| \| DUSP1 \| 9.1681E-175 \| -1.255303022 \| 2.0011E-170 \| 5 \| \| HCLS1 \| 2.0386E-174 \| -1.053190241 \| 4.4496E-170 \| 5 \| \| CD2 \| 5.8416E-174 \| -2.009470678 \| 1.2751E-169 \| 5 \| \| SLC2A3 \| 5.0288E-173 \| -1.469096464 \| 1.0976E-168 \| 5 \| \| TRAC \| 1.7955E-172 \| -1.890924421 \| 3.919E-168 \| 5 \| \| RHOH \| 1.8649E-171 \| -1.197619717 \| 4.0704E-167 \| 5 \| \| HSP90AB1 \| 2.2293E-165 \| -1.087109568 \| 4.8659E-161 \| 5 \| \| TAGAP \| 9.0446E-165 \| -1.18518763 \| 1.9742E-160 \| 5 \| \| GPSM3 \| 1.3031E-164 \| -1.025065019 \| 2.8442E-160 \| 5 \| \| B2M \| 4.5504E-161 \| -1.005801759 \| 9.9322E-157 \| 5 \| \| CELF2 \| 1.3033E-159 \| -1.189279039 \| 2.8447E-155 \| 5 \| \| NR4A2 \| 2.6175E-159 \| -1.740227194 \| 5.7131E-155 \| 5 \| \| DUSP5 \| 2.1221E-156 \| -1.050835919 \| 4.6319E-152 \| 5 \| \| CD81 \| 2.6658E-156 \| -1.217965746 \| 5.8186E-152 \| 5 \| \| KLF2 \| 4.028E-151 \| -1.492275221 \| 8.7919E-147 \| 5 \| \| PDE4B \| 2.3051E-148 \| -1.062571724 \| 5.0312E-144 \| 5 \| \| FLNA \| 1.6825E-146 \| -1.032583636 \| 3.6724E-142 \| 5 \| \| YPEL5 \| 2.0779E-146 \| -1.160335162 \| 4.5355E-142 \| 5 \| \| HLA-DPB1 \| 9.7149E-146 \| -2.542686824 \| 2.1205E-141 \| 5 \| \| IFI16 \| 1.7752E-145 \| -1.07565455 \| 3.8747E-141 \| 5 \| \| TNFAIP8 \| 1.3366E-144 \| -1.032856636 \| 2.9174E-140 \| 5 \| \| H2AFZ \| 1.9994E-136 \| -1.071540603 \| 4.3641E-132 \| 5 \| \| HSP90AA1 \| 1.9903E-133 \| -1.05667021 \| 4.3443E-129 \| 5 \| \| SERPINB9 \| 3.4397E-131 \| -1.046573107 \| 7.5079E-127 \| 5 \| \| PHLDA1 \| 2.6818E-130 \| -1.084774977 \| 5.8535E-126 \| 5 \| \| MYADM \| 3.576E-123 \| -1.116335569 \| 7.8053E-119 \| 5 \| \| ARID5B \| 4.4446E-123 \| -1.191645048 \| 9.7013E-119 \| 5 \| \| SARAF \| 3.6245E-122 \| -1.069964378 \| 7.9112E-118 \| 5 \| \| CD74 \| 1.987E-119 \| -2.154434748 \| 4.337E-115 \| 5 \| \| FKBP11 \| 1.595E-118 \| -1.089106716 \| 3.4814E-114 \| 5 \| \| NFKBIA \| 3.459E-117 \| -1.244118947 \| 7.5499E-113 \| 5 \| \| RARRES3 \| 8.0096E-108 \| -1.083249875 \| 1.7482E-103 \| 5 \| \| HEXIM1 \| 3.4746E-105 \| 1.08081429 \| 7.5839E-101 \| 5 \| \| COTL1 \| 4.54722E-90 \| -1.341555822 \| 9.92523E-86 \| 5 \| \| REL \| 4.33932E-88 \| -1.39820844 \| 9.47143E-84 \| 5 \| \| CNOT6L \| 5.78366E-87 \| -1.070165116 \| 1.2624E-82 \| 5 \| \| TFF3 \| 6.96218E-87 \| -1.690322016 \| 1.51963E-82 \| 5 \| \| TIMP1 \| 2.64656E-86 \| -1.630267766 \| 5.77665E-82 \| 5 \| \| ISG15 \| 2.14024E-85 \| -1.137898291 \| 4.67151E-81 \| 5 \| \| BTG1 \| 2.77615E-81 \| -1.268126829 \| 6.05951E-77 \| 5 \| \| JUNB \| 7.06141E-81 \| -1.044449949 \| 1.54129E-76 \| 5 \| \| HSPH1 \| 7.09554E-71 \| -1.097561245 \| 1.54874E-66 \| 5 \| \| DNAJB1 \| 4.83868E-42 \| -1.093255435 \| 1.05614E-37 \| 5 \| \| MUC2 \| 1.41839E-36 \| -1.631871693 \| 3.09593E-32 \| 5 \| \| JCHAIN \| 6.23061E-35 \| -4.380754589 \| 1.35995E-30 \| 5 \| \| IGKC \| 5.72823E-32 \| -4.297727383 \| 1.2503E-27 \| 5 \| \| SSR4 \| 1.87212E-20 \| -1.028860274 \| 4.08628E-16 \| 5 \| \| HLA-DRA \| 4.18306E-20 \| -3.003417011 \| 9.13036E-16 \| 5 \| \| LYZ \| 4.02035E-15 \| -2.277539468 \| 8.77521E-11 \| 5 \| \| AREG \| 9.57751E-15 \| -1.289134377 \| 2.09048E-10 \| 5 \| \| IGLC2 \| 1.00681E-14 \| -4.986850491 \| 2.19756E-10 \| 5 \| \| IGHA1 \| 8.43835E-09 \| -3.78748111 \| 0.000184184 \| 5 \| \| IGLC3 \| 7.55247E-07 \| -4.150975524 \| 0.016484783 \| 5 \| \| SPINK4 \| 0 \| 6.679076219 \| 0 \| 6 \| \| MUC2 \| 0 \| 5.91967503 \| 0 \| 6 \| \| CLCA1 \| 0 \| 5.786534431 \| 0 \| 6 \| \| ZG16 \| 0 \| 5.750947335 \| 0 \| 6 \| \| FCGBP \| 0 \| 5.579651492 \| 0 \| 6 \| \| TFF3 \| 0 \| 5.437720787 \| 0 \| 6 \| \| ITLN1 \| 0 \| 5.405604002 \| 0 \| 6 \| \| REG4 \| 0 \| 3.805379628 \| 0 \| 6 \| \| REP15 \| 0 \| 3.635326564 \| 0 \| 6 \| \| KLK1 \| 0 \| 3.376399441 \| 0 \| 6 \| \| AGR2 \| 0 \| 3.269911645 \| 0 \| 6 \| \| ST6GALNAC1 \| 0 \| 3.186281909 \| 0 \| 6 \| \| WFDC2 \| 0 \| 2.913021959 \| 0 \| 6 \| \| SPINK1 \| 0 \| 2.898924351 \| 0 \| 6 \| \| SERPINA1 \| 0 \| 2.786381939 \| 0 \| 6 \| \| FAM3D \| 0 \| 2.2173359 \| 0 \| 6 \| \| L1TD1 \| 0 \| 2.145937477 \| 0 \| 6 \| \| TFF1 \| 0 \| 2.137876929 \| 0 \| 6 \| \| SPDEF \| 0 \| 2.125559777 \| 0 \| 6 \| \| MUC4 \| 0 \| 2.121129799 \| 0 \| 6 \| \| HEPACAM2 \| 0 \| 2.112744203 \| 0 \| 6 \| \| LGALS4 \| 0 \| 2.090653142 \| 0 \| 6 \| \| GSN \| 0 \| 2.078254025 \| 0 \| 6 \| \| CDC42EP5 \| 0 \| 1.981301112 \| 0 \| 6 \| \| MLPH \| 0 \| 1.869184519 \| 0 \| 6 \| \| ERN2 \| 0 \| 1.838801919 \| 0 \| 6 \| \| SYTL2 \| 0 \| 1.835617036 \| 0 \| 6 \| \| FRYL \| 0 \| 1.784470165 \| 0 \| 6 \| \| ATOH1 \| 0 \| 1.781710889 \| 0 \| 6 \| \| RASD1 \| 0 \| 1.776914014 \| 0 \| 6 \| \| SCNN1A \| 0 \| 1.773011594 \| 0 \| 6 \| \| KIAA1324 \| 0 \| 1.765848237 \| 0 \| 6 \| \| CREB3L1 \| 0 \| 1.763471647 \| 0 \| 6 \| \| KRT18 \| 0 \| 1.745673357 \| 0 \| 6 \| \| TCEA3 \| 0 \| 1.681123007 \| 0 \| 6 \| \| KLF4 \| 0 \| 1.681051751 \| 0 \| 6 \| \| B3GNT6 \| 0 \| 1.670048166 \| 0 \| 6 \| \| RAP1GAP \| 0 \| 1.654967389 \| 0 \| 6 \| \| TCIM \| 0 \| 1.652233469 \| 0 \| 6 \| \| ENTPD8 \| 0 \| 1.646526757 \| 0 \| 6 \| \| SLC12A2 \| 0 \| 1.628597938 \| 0 \| 6 \| \| MT1G \| 0 \| 1.61754116 \| 0 \| 6 \| \| TPSG1 \| 0 \| 1.593747463 \| 0 \| 6 \| \| ELF3 \| 0 \| 1.563974139 \| 0 \| 6 \| \| GMDS \| 0 \| 1.563691332 \| 0 \| 6 \| \| NEDD4L \| 0 \| 1.556837067 \| 0 \| 6 \| \| ARFGEF3 \| 0 \| 1.550918205 \| 0 \| 6 \| \| GALNT7 \| 0 \| 1.533503142 \| 0 \| 6 \| \| HSD11B2 \| 0 \| 1.531746461 \| 0 \| 6 \| \| IGFBP2 \| 0 \| 1.520205089 \| 0 \| 6 \| \| RETNLB \| 0 \| 1.514451493 \| 0 \| 6 \| \| ANO7 \| 0 \| 1.496546813 \| 0 \| 6 \| \| GALNT5 \| 0 \| 1.487772033 \| 0 \| 6 \| \| NUPR1 \| 0 \| 1.486837716 \| 0 \| 6 \| \| SMIM14 \| 0 \| 1.475224302 \| 0 \| 6 \| \| VSIG2 \| 0 \| 1.474273139 \| 0 \| 6 \| \| TPM1 \| 0 \| 1.462908403 \| 0 \| 6 \| \| S100P \| 0 \| 1.444861006 \| 0 \| 6 \| \| AGR3 \| 0 \| 1.428023886 \| 0 \| 6 \| \| FABP2 \| 0 \| 1.422703184 \| 0 \| 6 \| \| CRACR2B \| 0 \| 1.420712089 \| 0 \| 6 \| \| BCAS1 \| 0 \| 1.393220143 \| 0 \| 6 \| \| NXPE1 \| 0 \| 1.389897753 \| 0 \| 6 \| \| GALNT12 \| 0 \| 1.387244161 \| 0 \| 6 \| \| TSPAN13 \| 0 \| 1.374058112 \| 0 \| 6 \| \| PRUNE2 \| 0 \| 1.371784895 \| 0 \| 6 \| \| WNK4 \| 0 \| 1.355565046 \| 0 \| 6 \| \| SLC44A4 \| 0 \| 1.343761058 \| 0 \| 6 \| \| GNE \| 0 \| 1.334167091 \| 0 \| 6 \| \| CKAP4 \| 0 \| 1.297115943 \| 0 \| 6 \| \| NEURL1 \| 0 \| 1.293781986 \| 0 \| 6 \| \| GALNT3 \| 0 \| 1.290671274 \| 0 \| 6 \| \| ATP2A3 \| 0 \| 1.280438399 \| 0 \| 6 \| \| GFPT1 \| 0 \| 1.279725827 \| 0 \| 6 \| \| MCF2L \| 0 \| 1.266567524 \| 0 \| 6 \| \| FOXA3 \| 0 \| 1.264163373 \| 0 \| 6 \| \| ATP2C2 \| 0 \| 1.253381306 \| 0 \| 6 \| \| HES6 \| 0 \| 1.238792346 \| 0 \| 6 \| \| TSTA3 \| 0 \| 1.230208277 \| 0 \| 6 \| \| EHF \| 0 \| 1.229520558 \| 0 \| 6 \| \| BEST2 \| 0 \| 1.215225826 \| 0 \| 6 \| \| CLDN4 \| 0 \| 1.204588224 \| 0 \| 6 \| \| CA12 \| 0 \| 1.202140625 \| 0 \| 6 \| \| MGLL \| 0 \| 1.201184029 \| 0 \| 6 \| \| AKAP9 \| 0 \| 1.198129469 \| 0 \| 6 \| \| SMIM22 \| 0 \| 1.194623116 \| 0 \| 6 \| \| KRT8 \| 0 \| 1.193386037 \| 0 \| 6 \| \| NXPE4 \| 0 \| 1.183196703 \| 0 \| 6 \| \| TM9SF3 \| 0 \| 1.179765884 \| 0 \| 6 \| \| SH3PXD2A \| 0 \| 1.177116741 \| 0 \| 6 \| \| QSOX1 \| 0 \| 1.166341346 \| 0 \| 6 \| \| PTPRF \| 0 \| 1.148656515 \| 0 \| 6 \| \| RAB26 \| 0 \| 1.135738336 \| 0 \| 6 \| \| TRPM4 \| 0 \| 1.132338651 \| 0 \| 6 \| \| ACSS2 \| 0 \| 1.129457243 \| 0 \| 6 \| \| IFI27 \| 0 \| 1.122065506 \| 0 \| 6 \| \| ESRP1 \| 0 \| 1.117512095 \| 0 \| 6 \| \| HID1 \| 0 \| 1.107915089 \| 0 \| 6 \| \| S100A6 \| 0 \| 1.102836468 \| 0 \| 6 \| \| S100A14 \| 0 \| 1.101626879 \| 0 \| 6 \| \| CLDN3 \| 0 \| 1.098443214 \| 0 \| 6 \| \| HMGCS2 \| 0 \| 1.092212931 \| 0 \| 6 \| \| TSPAN1 \| 0 \| 1.080941596 \| 0 \| 6 \| \| AC103702.2 \| 0 \| 1.069533191 \| 0 \| 6 \| \| MUC13 \| 0 \| 1.066006868 \| 0 \| 6 \| \| ATP8B1 \| 0 \| 1.060101383 \| 0 \| 6 \| \| MUC5B \| 0 \| 1.054284408 \| 0 \| 6 \| \| B4GALNT2 \| 0 \| 1.049787665 \| 0 \| 6 \| \| FAM83E \| 0 \| 1.048005479 \| 0 \| 6 \| \| KLF5 \| 0 \| 1.039722811 \| 0 \| 6 \| \| FAM13A \| 0 \| 1.036860334 \| 0 \| 6 \| \| UNC13B \| 0 \| 1.019659515 \| 0 \| 6 \| \| MBOAT2 \| 0 \| 1.01822961 \| 0 \| 6 \| \| RRBP1 \| 0 \| 1.017261338 \| 0 \| 6 \| \| CANT1 \| 0 \| 1.01402164 \| 0 \| 6 \| \| MAP7 \| 0 \| 1.012152039 \| 0 \| 6 \| \| ANXA13 \| 0 \| 1.011511464 \| 0 \| 6 \| \| DOPEY2 \| 0 \| 1.011244176 \| 0 \| 6 \| \| AOC1 \| 0 \| 1.00858235 \| 0 \| 6 \| \| LGALS9 \| 0 \| 1.00663407 \| 0 \| 6 \| \| PTPRC \| 0 \| -2.48588686 \| 0 \| 6 \| \| TSC22D3 \| 0 \| -2.595689182 \| 0 \| 6 \| \| VIM \| 0 \| -3.309810488 \| 0 \| 6 \| \| SRGN \| 0 \| -3.40222088 \| 0 \| 6 \| \| ARHGDIB \| 2.4881E-303 \| -2.065921615 \| 5.4307E-299 \| 6 \| \| KCNK6 \| 3.4661E-299 \| 1.17157515 \| 7.5655E-295 \| 6 \| \| FTL \| 9.0321E-296 \| -1.739784909 \| 1.9714E-291 \| 6 \| \| S100A4 \| 1.0501E-293 \| -2.728248446 \| 2.2921E-289 \| 6 \| \| MIA3 \| 1.5342E-281 \| 1.188599689 \| 3.3488E-277 \| 6 \| \| RGS1 \| 4.0753E-281 \| -2.48385374 \| 8.8953E-277 \| 6 \| \| CLMN \| 1.3056E-276 \| 1.020605451 \| 2.8498E-272 \| 6 \| \| NANS \| 1.9514E-276 \| 1.087981088 \| 4.2593E-272 \| 6 \| \| CXCR4 \| 1.4445E-264 \| -3.042707301 \| 3.1529E-260 \| 6 \| \| CD48 \| 3.1924E-261 \| -1.812132539 \| 6.968E-257 \| 6 \| \| GMFG \| 7.2477E-259 \| -1.540955246 \| 1.5819E-254 \| 6 \| \| DNAJC10 \| 1.0848E-255 \| 1.179077336 \| 2.3678E-251 \| 6 \| \| HDLBP \| 7.2559E-253 \| 1.083303492 \| 1.5837E-248 \| 6 \| \| EMP3 \| 2.4435E-249 \| -1.467534035 \| 5.3333E-245 \| 6 \| \| CYTIP \| 5.3592E-249 \| -1.677275073 \| 1.1698E-244 \| 6 \| \| CD52 \| 3.3585E-239 \| -2.383384222 \| 7.3306E-235 \| 6 \| \| LGALS1 \| 1.4028E-238 \| -2.309410844 \| 3.062E-234 \| 6 \| \| ZKSCAN1 \| 1.7346E-229 \| 1.009624731 \| 3.7862E-225 \| 6 \| \| BST2 \| 3.3308E-229 \| -1.244683355 \| 7.2702E-225 \| 6 \| \| CTBP2 \| 4.4479E-224 \| 1.118713405 \| 9.7083E-220 \| 6 \| \| MSN \| 6.2084E-218 \| -1.409533213 \| 1.3551E-213 \| 6 \| \| SMAP2 \| 1.5192E-216 \| -1.541125091 \| 3.316E-212 \| 6 \| \| LAPTM5 \| 1.706E-215 \| -2.013864822 \| 3.7236E-211 \| 6 \| \| TMSB4X \| 6.2007E-214 \| -1.345589041 \| 1.3534E-209 \| 6 \| \| LCP1 \| 1.8999E-211 \| -1.849558964 \| 4.147E-207 \| 6 \| \| TNFAIP3 \| 2.4474E-208 \| -2.333090179 \| 5.342E-204 \| 6 \| \| BTG1 \| 7.6763E-208 \| -1.645405571 \| 1.6755E-203 \| 6 \| \| CD53 \| 2.8371E-206 \| -1.451661539 \| 6.1926E-202 \| 6 \| \| STK17B \| 3.8687E-206 \| -1.544648053 \| 8.4443E-202 \| 6 \| \| GYPC \| 1.9005E-196 \| -1.099342087 \| 4.1483E-192 \| 6 \| \| CREM \| 4.1764E-193 \| -1.957636152 \| 9.1158E-189 \| 6 \| \| FYB1 \| 1.629E-190 \| -1.848614608 \| 3.5557E-186 \| 6 \| \| RAC2 \| 2.9225E-190 \| -1.383011669 \| 6.379E-186 \| 6 \| \| LSP1 \| 7.433E-190 \| -1.292478763 \| 1.6224E-185 \| 6 \| \| CD37 \| 1.4698E-186 \| -1.516252262 \| 3.2081E-182 \| 6 \| \| CD69 \| 1.6804E-184 \| -2.385010353 \| 3.6678E-180 \| 6 \| \| GPR183 \| 1.0724E-176 \| -1.901050473 \| 2.3408E-172 \| 6 \| \| HSP90AB1 \| 5.3533E-173 \| -1.134552914 \| 1.1685E-168 \| 6 \| \| GNAI2 \| 1.3886E-171 \| -1.265401455 \| 3.0309E-167 \| 6 \| \| HCST \| 6.4442E-170 \| -1.604319809 \| 1.4066E-165 \| 6 \| \| EVI2B \| 2.9464E-169 \| -1.091009599 \| 6.4311E-165 \| 6 \| \| PRDM1 \| 2.0082E-167 \| -1.321893736 \| 4.3834E-163 \| 6 \| \| ZNF331 \| 5.2775E-167 \| -1.852927465 \| 1.1519E-162 \| 6 \| \| ARL4C \| 9.2103E-167 \| -1.650381146 \| 2.0103E-162 \| 6 \| \| FTH1 \| 1.6652E-166 \| -1.607887918 \| 3.6347E-162 \| 6 \| \| ANXA1 \| 8.1922E-166 \| -2.208760979 \| 1.7881E-161 \| 6 \| \| CLEC2B \| 1.3192E-165 \| -1.450198989 \| 2.8794E-161 \| 6 \| \| GLIPR1 \| 3.2592E-163 \| -1.223869093 \| 7.1138E-159 \| 6 \| \| SAMSN1 \| 2.3753E-161 \| -1.538846749 \| 5.1845E-157 \| 6 \| \| DUSP2 \| 3.3491E-161 \| -1.889033017 \| 7.3101E-157 \| 6 \| \| FNBP1 \| 5.3462E-161 \| -1.089659881 \| 1.1669E-156 \| 6 \| \| IL7R \| 3.8359E-158 \| -2.790316983 \| 8.3726E-154 \| 6 \| \| ACTB \| 9.954E-156 \| -1.056274588 \| 2.1727E-151 \| 6 \| \| NFKBIA \| 1.4175E-154 \| -1.399954686 \| 3.094E-150 \| 6 \| \| HLA-DPA1 \| 2.8145E-154 \| -2.520982237 \| 6.1431E-150 \| 6 \| \| PNRC1 \| 1.6742E-153 \| -1.195414444 \| 3.6543E-149 \| 6 \| \| SLC2A3 \| 8.1274E-151 \| -1.44053341 \| 1.774E-146 \| 6 \| \| COTL1 \| 5.3197E-150 \| -1.567349429 \| 1.1611E-145 \| 6 \| \| CD3D \| 2.4689E-149 \| -2.026483681 \| 5.3889E-145 \| 6 \| \| ARID5B \| 2.6521E-147 \| -1.347215241 \| 5.7887E-143 \| 6 \| \| TAGAP \| 3.8389E-147 \| -1.179828109 \| 8.3792E-143 \| 6 \| \| HCLS1 \| 3.9819E-145 \| -1.01389658 \| 8.6912E-141 \| 6 \| \| TRAC \| 9.6465E-144 \| -1.821666744 \| 2.1055E-139 \| 6 \| \| CD2 \| 2.0532E-143 \| -1.922309704 \| 4.4815E-139 \| 6 \| \| RHOH \| 1.0849E-142 \| -1.151497496 \| 2.3679E-138 \| 6 \| \| SOCS3 \| 1.446E-142 \| -1.190791381 \| 3.1561E-138 \| 6 \| \| FXYD5 \| 2.8402E-142 \| -1.100186798 \| 6.1993E-138 \| 6 \| \| RGCC \| 4.1044E-142 \| -1.749731839 \| 8.9586E-138 \| 6 \| \| ATP1B3 \| 2.3289E-141 \| -1.327681586 \| 5.0833E-137 \| 6 \| \| TUBA1A \| 1.141E-134 \| -1.314345775 \| 2.4904E-130 \| 6 \| \| SARAF \| 9.9083E-133 \| -1.126793773 \| 2.1627E-128 \| 6 \| \| HLA-DPB1 \| 4.0526E-132 \| -2.459316639 \| 8.8456E-128 \| 6 \| \| RGS2 \| 6.8755E-131 \| -1.516119787 \| 1.5007E-126 \| 6 \| \| AC058791.1 \| 2.8071E-128 \| -1.293576894 \| 6.127E-124 \| 6 \| \| DUSP5 \| 3.9814E-121 \| -1.049639707 \| 8.6902E-117 \| 6 \| \| DNAJA1 \| 6.411E-110 \| -1.304286787 \| 1.3993E-105 \| 6 \| \| YPEL5 \| 3.2076E-106 \| -1.062254338 \| 7.0012E-102 \| 6 \| \| REL \| 1.83957E-90 \| -1.408852612 \| 4.01524E-86 \| 6 \| \| RARRES3 \| 1.94473E-88 \| -1.005059994 \| 4.24476E-84 \| 6 \| \| JUNB \| 1.30493E-80 \| -1.066958822 \| 2.84827E-76 \| 6 \| \| HLA-DRA \| 2.16251E-72 \| -3.367370122 \| 4.7201E-68 \| 6 \| \| GADD45B \| 1.31771E-68 \| -1.042435821 \| 2.87617E-64 \| 6 \| \| NR4A2 \| 2.7854E-66 \| -1.354658271 \| 6.07969E-62 \| 6 \| \| KLF2 \| 8.1664E-56 \| -1.151551772 \| 1.78248E-51 \| 6 \| \| CD74 \| 4.51643E-54 \| -2.006804448 \| 9.85802E-50 \| 6 \| \| DNAJB1 \| 8.62247E-43 \| -1.056268895 \| 1.88203E-38 \| 6 \| \| JCHAIN \| 1.51063E-39 \| -3.694927934 \| 3.29724E-35 \| 6 \| \| IGKC \| 6.72792E-38 \| -3.882100116 \| 1.4685E-33 \| 6 \| \| AREG \| 6.34159E-18 \| -1.189366487 \| 1.38418E-13 \| 6 \| \| IGHA1 \| 2.86327E-15 \| -3.417030371 \| 6.24966E-11 \| 6 \| \| IGLC2 \| 2.373E-12 \| -4.602553898 \| 5.17955E-08 \| 6 \| \| TIMP1 \| 5.27873E-09 \| -1.080914053 \| 0.000115219 \| 6 \| \| IGLC3 \| 7.98502E-07 \| -4.237929271 \| 0.017428913 \| 6 \| \| GUCA2A \| 0 \| 5.352762745 \| 0 \| 7 \| \| CLCA4 \| 0 \| 5.232710052 \| 0 \| 7 \| \| SLC26A3 \| 0 \| 4.877838757 \| 0 \| 7 \| \| CEACAM7 \| 0 \| 4.656941299 \| 0 \| 7 \| \| GUCA2B \| 0 \| 4.529660797 \| 0 \| 7 \| \| PHGR1 \| 0 \| 4.496263208 \| 0 \| 7 \| \| AQP8 \| 0 \| 4.317257526 \| 0 \| 7 \| \| FABP1 \| 0 \| 4.093787547 \| 0 \| 7 \| \| SELENOP \| 0 \| 4.080711599 \| 0 \| 7 \| \| TSPAN1 \| 0 \| 3.878917425 \| 0 \| 7 \| \| PLAC8 \| 0 \| 3.728967322 \| 0 \| 7 \| \| FXYD3 \| 0 \| 3.657060517 \| 0 \| 7 \| \| IFI27 \| 0 \| 3.603502955 \| 0 \| 7 \| \| CDHR5 \| 0 \| 3.428702772 \| 0 \| 7 \| \| KRT20 \| 0 \| 3.398239613 \| 0 \| 7 \| \| CEACAM1 \| 0 \| 3.361425029 \| 0 \| 7 \| \| SLC9A3 \| 0 \| 3.307364465 \| 0 \| 7 \| \| MS4A12 \| 0 \| 3.238728079 \| 0 \| 7 \| \| CA2 \| 0 \| 3.217882935 \| 0 \| 7 \| \| MUC12 \| 0 \| 3.202809422 \| 0 \| 7 \| \| ANPEP \| 0 \| 3.142717409 \| 0 \| 7 \| \| TMEM54 \| 0 \| 3.125602109 \| 0 \| 7 \| \| CA1 \| 0 \| 3.082159723 \| 0 \| 7 \| \| CA4 \| 0 \| 2.946246921 \| 0 \| 7 \| \| GPA33 \| 0 \| 2.940796494 \| 0 \| 7 \| \| DUOX2 \| 0 \| 2.915739368 \| 0 \| 7 \| \| MISP \| 0 \| 2.887899819 \| 0 \| 7 \| \| ISG15 \| 0 \| 2.88702017 \| 0 \| 7 \| \| PRSS3 \| 0 \| 2.869228597 \| 0 \| 7 \| \| KRT19 \| 0 \| 2.85536624 \| 0 \| 7 \| \| SMIM22 \| 0 \| 2.851214526 \| 0 \| 7 \| \| SDCBP2 \| 0 \| 2.834375664 \| 0 \| 7 \| \| MUC13 \| 0 \| 2.781616766 \| 0 \| 7 \| \| TMPRSS2 \| 0 \| 2.7171162 \| 0 \| 7 \| \| CD177 \| 0 \| 2.661487423 \| 0 \| 7 \| \| HIST1H1C \| 0 \| 2.630757165 \| 0 \| 7 \| \| CKB \| 0 \| 2.626630994 \| 0 \| 7 \| \| MDK \| 0 \| 2.607274894 \| 0 \| 7 \| \| SFN \| 0 \| 2.592786865 \| 0 \| 7 \| \| CEACAM6 \| 0 \| 2.587718747 \| 0 \| 7 \| \| PIGR \| 0 \| 2.565192691 \| 0 \| 7 \| \| ITM2C \| 0 \| 2.56145952 \| 0 \| 7 \| \| PKIB \| 0 \| 2.507717703 \| 0 \| 7 \| \| KRT8 \| 0 \| 2.479119678 \| 0 \| 7 \| \| PLS1 \| 0 \| 2.45903862 \| 0 \| 7 \| \| TRIM31 \| 0 \| 2.434742284 \| 0 \| 7 \| \| AOC1 \| 0 \| 2.431497964 \| 0 \| 7 \| \| DHRS9 \| 0 \| 2.388689584 \| 0 \| 7 \| \| S100A6 \| 0 \| 2.378768321 \| 0 \| 7 \| \| PRSS8 \| 0 \| 2.359398362 \| 0 \| 7 \| \| CLDN3 \| 0 \| 2.357320713 \| 0 \| 7 \| \| RHOC \| 0 \| 2.344757835 \| 0 \| 7 \| \| DST \| 0 \| 2.310555797 \| 0 \| 7 \| \| CLDN4 \| 0 \| 2.307610109 \| 0 \| 7 \| \| LGALS3 \| 0 \| 2.305812457 \| 0 \| 7 \| \| SERINC2 \| 0 \| 2.299958281 \| 0 \| 7 \| \| TSPAN8 \| 0 \| 2.289019623 \| 0 \| 7 \| \| EPCAM \| 0 \| 2.265658301 \| 0 \| 7 \| \| MEP1A \| 0 \| 2.255450661 \| 0 \| 7 \| \| EMP1 \| 0 \| 2.237389327 \| 0 \| 7 \| \| C2orf88 \| 0 \| 2.190344704 \| 0 \| 7 \| \| TMIGD1 \| 0 \| 2.18497635 \| 0 \| 7 \| \| SRI \| 0 \| 2.180217266 \| 0 \| 7 \| \| HPGD \| 0 \| 2.163773748 \| 0 \| 7 \| \| CDHR2 \| 0 \| 2.161538364 \| 0 \| 7 \| \| LGALS4 \| 0 \| 2.160580458 \| 0 \| 7 \| \| SLC44A4 \| 0 \| 2.136960251 \| 0 \| 7 \| \| ST14 \| 0 \| 2.110804747 \| 0 \| 7 \| \| DSC2 \| 0 \| 2.100817367 \| 0 \| 7 \| \| TST \| 0 \| 2.096865073 \| 0 \| 7 \| \| CLTB \| 0 \| 2.091237317 \| 0 \| 7 \| \| MYH14 \| 0 \| 2.069340466 \| 0 \| 7 \| \| SPINT1 \| 0 \| 2.066374526 \| 0 \| 7 \| \| CGN \| 0 \| 2.066311536 \| 0 \| 7 \| \| MGLL \| 0 \| 2.06248684 \| 0 \| 7 \| \| GPRC5A \| 0 \| 2.040832276 \| 0 \| 7 \| \| TSPAN3 \| 0 \| 2.039238744 \| 0 \| 7 \| \| SLC6A8 \| 0 \| 2.035262997 \| 0 \| 7 \| \| SPATS2L \| 0 \| 2.034064945 \| 0 \| 7 \| \| CDKN2B-AS1 \| 0 \| 2.032654536 \| 0 \| 7 \| \| PI3 \| 0 \| 2.030340244 \| 0 \| 7 \| \| ENTPD8 \| 0 \| 2.026519266 \| 0 \| 7 \| \| TMEM37 \| 0 \| 2.022325765 \| 0 \| 7 \| \| TCF7L2 \| 0 \| 2.021738694 \| 0 \| 7 \| \| TFF1 \| 0 \| 2.017254696 \| 0 \| 7 \| \| SMPDL3A \| 0 \| 2.00842227 \| 0 \| 7 \| \| PDE9A \| 0 \| 2.005670056 \| 0 \| 7 \| \| SLC17A4 \| 0 \| 1.998518033 \| 0 \| 7 \| \| SLC51B \| 0 \| 1.996112618 \| 0 \| 7 \| \| CAMK2N1 \| 0 \| 1.966407363 \| 0 \| 7 \| \| PPP1R14D \| 0 \| 1.964622294 \| 0 \| 7 \| \| GSN \| 0 \| 1.952140789 \| 0 \| 7 \| \| S100A10 \| 0 \| 1.938665508 \| 0 \| 7 \| \| SLC4A4 \| 0 \| 1.927443142 \| 0 \| 7 \| \| CDA \| 0 \| 1.92700264 \| 0 \| 7 \| \| AMN \| 0 \| 1.923077259 \| 0 \| 7 \| \| ASS1 \| 0 \| 1.906613005 \| 0 \| 7 \| \| SGK1 \| 0 \| 1.904811794 \| 0 \| 7 \| \| ALDOB \| 0 \| 1.893659536 \| 0 \| 7 \| \| MXI1 \| 0 \| 1.893469882 \| 0 \| 7 \| \| CFDP1 \| 0 \| 1.883855275 \| 0 \| 7 \| \| ATP1B1 \| 0 \| 1.880836491 \| 0 \| 7 \| \| XDH \| 0 \| 1.880456446 \| 0 \| 7 \| \| CES2 \| 0 \| 1.851142383 \| 0 \| 7 \| \| FLNB \| 0 \| 1.836405437 \| 0 \| 7 \| \| CYP3A5 \| 0 \| 1.83552074 \| 0 \| 7 \| \| LMO7 \| 0 \| 1.832599402 \| 0 \| 7 \| \| HK2 \| 0 \| 1.83009816 \| 0 \| 7 \| \| TNFSF10 \| 0 \| 1.823815554 \| 0 \| 7 \| \| ETHE1 \| 0 \| 1.818627478 \| 0 \| 7 \| \| BLOC1S1 \| 0 \| 1.809263246 \| 0 \| 7 \| \| DUOXA2 \| 0 \| 1.805134753 \| 0 \| 7 \| \| HHLA2 \| 0 \| 1.798903192 \| 0 \| 7 \| \| LGALS3BP \| 0 \| 1.782072656 \| 0 \| 7 \| \| CDH1 \| 0 \| 1.778052428 \| 0 \| 7 \| \| C10orf99 \| 0 \| 1.773016572 \| 0 \| 7 \| \| DHRS11 \| 0 \| 1.769116609 \| 0 \| 7 \| \| ARL14 \| 0 \| 1.769050616 \| 0 \| 7 \| \| FCGRT \| 0 \| 1.767466593 \| 0 \| 7 \| \| DSG2 \| 0 \| 1.758541032 \| 0 \| 7 \| \| CDKN2B \| 0 \| 1.74054504 \| 0 \| 7 \| \| TMC5 \| 0 \| 1.728697554 \| 0 \| 7 \| \| HSD17B2 \| 0 \| 1.727260957 \| 0 \| 7 \| \| PCK1 \| 0 \| 1.726119578 \| 0 \| 7 \| \| SAMD9 \| 0 \| 1.723317723 \| 0 \| 7 \| \| ACAA2 \| 0 \| 1.722466158 \| 0 \| 7 \| \| COL17A1 \| 0 \| 1.716097452 \| 0 \| 7 \| \| UGT2B17 \| 0 \| 1.715856968 \| 0 \| 7 \| \| GIPC1 \| 0 \| 1.706990111 \| 0 \| 7 \| \| CDH17 \| 0 \| 1.702890302 \| 0 \| 7 \| \| TRPM6 \| 0 \| 1.702228783 \| 0 \| 7 \| \| ELF3 \| 0 \| 1.699154129 \| 0 \| 7 \| \| MIER3 \| 0 \| 1.698461907 \| 0 \| 7 \| \| BSG \| 0 \| 1.696334746 \| 0 \| 7 \| \| CDC42EP5 \| 0 \| 1.693528832 \| 0 \| 7 \| \| TJP3 \| 0 \| 1.690922969 \| 0 \| 7 \| \| C11orf86 \| 0 \| 1.678634862 \| 0 \| 7 \| \| EFHD2 \| 0 \| 1.67766706 \| 0 \| 7 \| \| PPDPF \| 0 \| 1.675433142 \| 0 \| 7 \| \| MYL12B \| 0 \| 1.674245552 \| 0 \| 7 \| \| MVP \| 0 \| 1.672938446 \| 0 \| 7 \| \| LAMB3 \| 0 \| 1.668597072 \| 0 \| 7 \| \| TDP2 \| 0 \| 1.661522544 \| 0 \| 7 \| \| C1QTNF12 \| 0 \| 1.660880968 \| 0 \| 7 \| \| RFK \| 0 \| 1.659952248 \| 0 \| 7 \| \| CHMP2A \| 0 \| 1.654876936 \| 0 \| 7 \| \| TMEM45B \| 0 \| 1.654854094 \| 0 \| 7 \| \| TP53I3 \| 0 \| 1.652318845 \| 0 \| 7 \| \| VAMP8 \| 0 \| 1.644917348 \| 0 \| 7 \| \| TPRN \| 0 \| 1.641425362 \| 0 \| 7 \| \| ABCA5 \| 0 \| 1.640330465 \| 0 \| 7 \| \| CCDC68 \| 0 \| 1.63124812 \| 0 \| 7 \| \| USP53 \| 0 \| 1.627206002 \| 0 \| 7 \| \| CLDN23 \| 0 \| 1.622453724 \| 0 \| 7 \| \| CA12 \| 0 \| 1.606519844 \| 0 \| 7 \| \| LSR \| 0 \| 1.603828428 \| 0 \| 7 \| \| PLA2G10 \| 0 \| 1.601682558 \| 0 \| 7 \| \| SCNN1B \| 0 \| 1.596782214 \| 0 \| 7 \| \| DDX60 \| 0 \| 1.595809744 \| 0 \| 7 \| \| VILL \| 0 \| 1.594455221 \| 0 \| 7 \| \| PDZK1IP1 \| 0 \| 1.580142564 \| 0 \| 7 \| \| EPS8 \| 0 \| 1.5785144 \| 0 \| 7 \| \| C4orf48 \| 0 \| 1.578079724 \| 0 \| 7 \| \| GGT6 \| 0 \| 1.577815307 \| 0 \| 7 \| \| SELENBP1 \| 0 \| 1.577427633 \| 0 \| 7 \| \| APPL2 \| 0 \| 1.57401891 \| 0 \| 7 \| \| MAPK3 \| 0 \| 1.573555467 \| 0 \| 7 \| \| CHP1 \| 0 \| 1.569596719 \| 0 \| 7 \| \| RBM47 \| 0 \| 1.55689899 \| 0 \| 7 \| \| UGP2 \| 0 \| 1.555728032 \| 0 \| 7 \| \| LIPH \| 0 \| 1.554509531 \| 0 \| 7 \| \| EPS8L2 \| 0 \| 1.552414916 \| 0 \| 7 \| \| GNG12 \| 0 \| 1.540326429 \| 0 \| 7 \| \| PDCD6IP \| 0 \| 1.539730803 \| 0 \| 7 \| \| RETSAT \| 0 \| 1.536091886 \| 0 \| 7 \| \| GNA11 \| 0 \| 1.535788829 \| 0 \| 7 \| \| NEAT1 \| 0 \| 1.534941025 \| 0 \| 7 \| \| LAMA3 \| 0 \| 1.533127587 \| 0 \| 7 \| \| LCN2 \| 0 \| 1.528272447 \| 0 \| 7 \| \| CTSA \| 0 \| 1.527905495 \| 0 \| 7 \| \| SSFA2 \| 0 \| 1.523195383 \| 0 \| 7 \| \| ACVRL1 \| 0 \| 1.516869306 \| 0 \| 7 \| \| HNF4A \| 0 \| 1.513447669 \| 0 \| 7 \| \| SMPD1 \| 0 \| 1.510365687 \| 0 \| 7 \| \| MAL2 \| 0 \| 1.506945642 \| 0 \| 7 \| \| KRT18 \| 0 \| 1.506022667 \| 0 \| 7 \| \| AHCYL2 \| 0 \| 1.502118943 \| 0 \| 7 \| \| SLC40A1 \| 0 \| 1.490514428 \| 0 \| 7 \| \| CTNND1 \| 0 \| 1.49024262 \| 0 \| 7 \| \| MXD1 \| 0 \| 1.488962443 \| 0 \| 7 \| \| LINC02023 \| 0 \| 1.479178892 \| 0 \| 7 \| \| SLC26A2 \| 0 \| 1.478167025 \| 0 \| 7 \| \| LIMA1 \| 0 \| 1.474468284 \| 0 \| 7 \| \| SLC22A18 \| 0 \| 1.470454846 \| 0 \| 7 \| \| SECTM1 \| 0 \| 1.466331652 \| 0 \| 7 \| \| FAM3D \| 0 \| 1.46379522 \| 0 \| 7 \| \| TMBIM1 \| 0 \| 1.459073273 \| 0 \| 7 \| \| EFNA1 \| 0 \| 1.458117205 \| 0 \| 7 \| \| NBL1 \| 0 \| 1.456200258 \| 0 \| 7 \| \| VSIG2 \| 0 \| 1.45534581 \| 0 \| 7 \| \| FUT3 \| 0 \| 1.453094491 \| 0 \| 7 \| \| NR2F6 \| 0 \| 1.451926623 \| 0 \| 7 \| \| MYO7B \| 0 \| 1.451029971 \| 0 \| 7 \| \| INF2 \| 0 \| 1.445663009 \| 0 \| 7 \| \| PDLIM1 \| 0 \| 1.44329727 \| 0 \| 7 \| \| SCNN1A \| 0 \| 1.439691063 \| 0 \| 7 \| \| SEMA3B \| 0 \| 1.432925438 \| 0 \| 7 \| \| PKP3 \| 0 \| 1.429754353 \| 0 \| 7 \| \| FBLIM1 \| 0 \| 1.426611125 \| 0 \| 7 \| \| LRRC19 \| 0 \| 1.420860035 \| 0 \| 7 \| \| EPS8L3 \| 0 \| 1.415037573 \| 0 \| 7 \| \| JUP \| 0 \| 1.411637568 \| 0 \| 7 \| \| NET1 \| 0 \| 1.408869626 \| 0 \| 7 \| \| PLEC \| 0 \| 1.406760649 \| 0 \| 7 \| \| PTTG1IP \| 0 \| 1.397897409 \| 0 \| 7 \| \| RHOF \| 0 \| 1.397200178 \| 0 \| 7 \| \| PARM1 \| 0 \| 1.394038285 \| 0 \| 7 \| \| TP53INP2 \| 0 \| 1.392960579 \| 0 \| 7 \| \| CASP7 \| 0 \| 1.390691857 \| 0 \| 7 \| \| LINC01133 \| 0 \| 1.377848316 \| 0 \| 7 \| \| GOLM1 \| 0 \| 1.375840356 \| 0 \| 7 \| \| DGAT1 \| 0 \| 1.37465801 \| 0 \| 7 \| \| C19orf33 \| 0 \| 1.374158003 \| 0 \| 7 \| \| H2AFJ \| 0 \| 1.371012784 \| 0 \| 7 \| \| COMTD1 \| 0 \| 1.36708386 \| 0 \| 7 \| \| PRR13 \| 0 \| 1.360954677 \| 0 \| 7 \| \| TNFRSF1A \| 0 \| 1.354902511 \| 0 \| 7 \| \| CLSTN1 \| 0 \| 1.345985719 \| 0 \| 7 \| \| PTPRF \| 0 \| 1.342318713 \| 0 \| 7 \| \| PNPLA2 \| 0 \| 1.341130453 \| 0 \| 7 \| \| APOBR \| 0 \| 1.332819946 \| 0 \| 7 \| \| SULT1B1 \| 0 \| 1.331830198 \| 0 \| 7 \| \| PLA2G2A \| 0 \| 1.321017405 \| 0 \| 7 \| \| BICDL2 \| 0 \| 1.31956553 \| 0 \| 7 \| \| HDHD3 \| 0 \| 1.319452759 \| 0 \| 7 \| \| JPT1 \| 0 \| 1.313819669 \| 0 \| 7 \| \| TRANK1 \| 0 \| 1.312887523 \| 0 \| 7 \| \| RETREG1 \| 0 \| 1.311528726 \| 0 \| 7 \| \| HSD17B11 \| 0 \| 1.309681099 \| 0 \| 7 \| \| PLOD2 \| 0 \| 1.305565457 \| 0 \| 7 \| \| ABHD17C \| 0 \| 1.304804343 \| 0 \| 7 \| \| CHP2 \| 0 \| 1.301802543 \| 0 \| 7 \| \| RIOK3 \| 0 \| 1.300868398 \| 0 \| 7 \| \| C1orf115 \| 0 \| 1.299578717 \| 0 \| 7 \| \| TMC4 \| 0 \| 1.296704374 \| 0 \| 7 \| \| NAAA \| 0 \| 1.295439274 \| 0 \| 7 \| \| CASP5 \| 0 \| 1.293514284 \| 0 \| 7 \| \| KLF4 \| 0 \| 1.292334574 \| 0 \| 7 \| \| AC023157.3 \| 0 \| 1.291455833 \| 0 \| 7 \| \| PAPSS2 \| 0 \| 1.290290789 \| 0 \| 7 \| \| EGLN3 \| 0 \| 1.290049171 \| 0 \| 7 \| \| ZBTB7B \| 0 \| 1.283925041 \| 0 \| 7 \| \| SMIM5 \| 0 \| 1.28011692 \| 0 \| 7 \| \| LINC01559 \| 0 \| 1.278895803 \| 0 \| 7 \| \| ABCB1 \| 0 \| 1.278120526 \| 0 \| 7 \| \| ACADS \| 0 \| 1.272509884 \| 0 \| 7 \| \| RRBP1 \| 0 \| 1.271723684 \| 0 \| 7 \| \| MTRNR2L12 \| 0 \| 1.269742601 \| 0 \| 7 \| \| SHROOM1 \| 0 \| 1.262558905 \| 0 \| 7 \| \| FUCA1 \| 0 \| 1.260065484 \| 0 \| 7 \| \| IGSF9 \| 0 \| 1.251482268 \| 0 \| 7 \| \| CD2AP \| 0 \| 1.250415986 \| 0 \| 7 \| \| GRN \| 0 \| 1.249281135 \| 0 \| 7 \| \| STAP2 \| 0 \| 1.249035841 \| 0 \| 7 \| \| PRR15 \| 0 \| 1.2463554 \| 0 \| 7 \| \| CIB1 \| 0 \| 1.244195636 \| 0 \| 7 \| \| LRRC1 \| 0 \| 1.242828139 \| 0 \| 7 \| \| MYL6 \| 0 \| 1.240386985 \| 0 \| 7 \| \| HEPH \| 0 \| 1.239199633 \| 0 \| 7 \| \| CEACAM5 \| 0 \| 1.2355148 \| 0 \| 7 \| \| BAIAP2L1 \| 0 \| 1.234719674 \| 0 \| 7 \| \| PLCD3 \| 0 \| 1.234421657 \| 0 \| 7 \| \| PTPRH \| 0 \| 1.228903511 \| 0 \| 7 \| \| HSD11B2 \| 0 \| 1.225109844 \| 0 \| 7 \| \| BTNL3 \| 0 \| 1.219100472 \| 0 \| 7 \| \| EDN3 \| 0 \| 1.213337309 \| 0 \| 7 \| \| SIRT6 \| 0 \| 1.212431356 \| 0 \| 7 \| \| PDZD3 \| 0 \| 1.210403619 \| 0 \| 7 \| \| CLRN3 \| 0 \| 1.203709191 \| 0 \| 7 \| \| MAST2 \| 0 \| 1.20200601 \| 0 \| 7 \| \| CCL28 \| 0 \| 1.201916165 \| 0 \| 7 \| \| TFCP2L1 \| 0 \| 1.196974403 \| 0 \| 7 \| \| LLGL2 \| 0 \| 1.196707793 \| 0 \| 7 \| \| TMEM236 \| 0 \| 1.196237832 \| 0 \| 7 \| \| TMCC3 \| 0 \| 1.189106143 \| 0 \| 7 \| \| ATP8B1 \| 0 \| 1.188234936 \| 0 \| 7 \| \| SULT1A2 \| 0 \| 1.179896632 \| 0 \| 7 \| \| C15orf48 \| 0 \| 1.179785413 \| 0 \| 7 \| \| PLXNB2 \| 0 \| 1.174426085 \| 0 \| 7 \| \| RASSF7 \| 0 \| 1.158966673 \| 0 \| 7 \| \| HRCT1 \| 0 \| 1.158258476 \| 0 \| 7 \| \| MIR194-2HG \| 0 \| 1.158192718 \| 0 \| 7 \| \| SCIN \| 0 \| 1.15558473 \| 0 \| 7 \| \| B3GALT5 \| 0 \| 1.15276122 \| 0 \| 7 \| \| MARCKS \| 0 \| 1.150605144 \| 0 \| 7 \| \| PRAP1 \| 0 \| 1.146315585 \| 0 \| 7 \| \| SULT1A1 \| 0 \| 1.144824547 \| 0 \| 7 \| \| SPTBN1 \| 0 \| 1.144459566 \| 0 \| 7 \| \| MAOA \| 0 \| 1.144439331 \| 0 \| 7 \| \| CAPN5 \| 0 \| 1.122816026 \| 0 \| 7 \| \| PDGFA \| 0 \| 1.122069326 \| 0 \| 7 \| \| LYPD8 \| 0 \| 1.12162423 \| 0 \| 7 \| \| AKR1B10 \| 0 \| 1.116592949 \| 0 \| 7 \| \| SMIM31 \| 0 \| 1.100021411 \| 0 \| 7 \| \| INAVA \| 0 \| 1.097980789 \| 0 \| 7 \| \| TLCD2 \| 0 \| 1.092261602 \| 0 \| 7 \| \| MYO15B \| 0 \| 1.087427889 \| 0 \| 7 \| \| AC254629.1 \| 0 \| 1.083547101 \| 0 \| 7 \| \| NR3C2 \| 0 \| 1.07342858 \| 0 \| 7 \| \| DOK4 \| 0 \| 1.071834405 \| 0 \| 7 \| \| TTC22 \| 0 \| 1.069647443 \| 0 \| 7 \| \| TMEM171 \| 0 \| 1.063845487 \| 0 \| 7 \| \| FABP2 \| 0 \| 1.062962008 \| 0 \| 7 \| \| SLC1A1 \| 0 \| 1.062705002 \| 0 \| 7 \| \| SEMA6A \| 0 \| 1.06085421 \| 0 \| 7 \| \| VIPR1 \| 0 \| 1.056929078 \| 0 \| 7 \| \| SGK2 \| 0 \| 1.053090661 \| 0 \| 7 \| \| RHOU \| 0 \| 1.042259952 \| 0 \| 7 \| \| CD63 \| 0 \| 1.019041157 \| 0 \| 7 \| \| B4GALNT3 \| 0 \| 1.013973173 \| 0 \| 7 \| \| GPT \| 0 \| 1.011180538 \| 0 \| 7 \| \| FSIP2 \| 0 \| 1.009825245 \| 0 \| 7 \| \| SIPA1L3 \| 0 \| 1.009147175 \| 0 \| 7 \| \| PLEKHG6 \| 0 \| 1.007484551 \| 0 \| 7 \| \| KIAA1522 \| 0 \| 1.003057956 \| 0 \| 7 \| \| FRMD1 \| 0 \| 1.002609863 \| 0 \| 7 \| \| B3GNT3 \| 0 \| 1.000066732 \| 0 \| 7 \| \| FAU \| 0 \| -1.137429531 \| 0 \| 7 \| \| RPL38 \| 0 \| -1.335174373 \| 0 \| 7 \| \| YBX1 \| 0 \| -1.368657803 \| 0 \| 7 \| \| RPL36 \| 0 \| -1.406878578 \| 0 \| 7 \| \| RPS15 \| 0 \| -1.413002343 \| 0 \| 7 \| \| RPS10 \| 0 \| -1.431878178 \| 0 \| 7 \| \| RACK1 \| 0 \| -1.442755511 \| 0 \| 7 \| \| RPL8 \| 0 \| -1.451201316 \| 0 \| 7 \| \| RPL18 \| 0 \| -1.500353116 \| 0 \| 7 \| \| RPL31 \| 0 \| -1.522043608 \| 0 \| 7 \| \| RPL27A \| 0 \| -1.526003971 \| 0 \| 7 \| \| RPS11 \| 0 \| -1.54488203 \| 0 \| 7 \| \| RPS25 \| 0 \| -1.548542593 \| 0 \| 7 \| \| RPS28 \| 0 \| -1.562699165 \| 0 \| 7 \| \| RPL28 \| 0 \| -1.571559626 \| 0 \| 7 \| \| RPS29 \| 0 \| -1.58822141 \| 0 \| 7 \| \| EEF1A1 \| 0 \| -1.6022533 \| 0 \| 7 \| \| NCL \| 0 \| -1.613670611 \| 0 \| 7 \| \| RPS3 \| 0 \| -1.621626953 \| 0 \| 7 \| \| RPL29 \| 0 \| -1.637453983 \| 0 \| 7 \| \| RPLP2 \| 0 \| -1.65240556 \| 0 \| 7 \| \| EEF1B2 \| 0 \| -1.665330902 \| 0 \| 7 \| \| RPL11 \| 0 \| -1.665588772 \| 0 \| 7 \| \| RPL27 \| 0 \| -1.6710633 \| 0 \| 7 \| \| RPL22 \| 0 \| -1.675318518 \| 0 \| 7 \| \| RPS9 \| 0 \| -1.704639672 \| 0 \| 7 \| \| RPS14 \| 0 \| -1.705378939 \| 0 \| 7 \| \| CD81 \| 0 \| -1.726280022 \| 0 \| 7 \| \| NPM1 \| 0 \| -1.739224554 \| 0 \| 7 \| \| RPL35 \| 0 \| -1.740899007 \| 0 \| 7 \| \| NACA \| 0 \| -1.812434962 \| 0 \| 7 \| \| RPL13A \| 0 \| -1.818095324 \| 0 \| 7 \| \| RPL7 \| 0 \| -1.818842596 \| 0 \| 7 \| \| RPL9 \| 0 \| -1.827550615 \| 0 \| 7 \| \| RPL34 \| 0 \| -1.832318447 \| 0 \| 7 \| \| RPL24 \| 0 \| -1.837576053 \| 0 \| 7 \| \| RPL14 \| 0 \| -1.854088573 \| 0 \| 7 \| \| RPL37A \| 0 \| -1.859816579 \| 0 \| 7 \| \| RPS27 \| 0 \| -1.870633908 \| 0 \| 7 \| \| RPS5 \| 0 \| -1.873601808 \| 0 \| 7 \| \| RPS21 \| 0 \| -1.876523299 \| 0 \| 7 \| \| NAP1L1 \| 0 \| -1.878632898 \| 0 \| 7 \| \| RPLP0 \| 0 \| -1.890927463 \| 0 \| 7 \| \| RPL23 \| 0 \| -1.895061941 \| 0 \| 7 \| \| RPL12 \| 0 \| -1.912454236 \| 0 \| 7 \| \| HSPD1 \| 0 \| -1.946913025 \| 0 \| 7 \| \| HSP90AB1 \| 0 \| -1.948823219 \| 0 \| 7 \| \| RPL35A \| 0 \| -1.965559287 \| 0 \| 7 \| \| RPS16 \| 0 \| -1.995461452 \| 0 \| 7 \| \| RPL7A \| 0 \| -2.015854348 \| 0 \| 7 \| \| RPL30 \| 0 \| -2.025248147 \| 0 \| 7 \| \| RPL4 \| 0 \| -2.031470533 \| 0 \| 7 \| \| RPL19 \| 0 \| -2.038890048 \| 0 \| 7 \| \| RPL10 \| 0 \| -2.044827239 \| 0 \| 7 \| \| RPL23A \| 0 \| -2.080434549 \| 0 \| 7 \| \| RPL18A \| 0 \| -2.104861667 \| 0 \| 7 \| \| RPS13 \| 0 \| -2.106079616 \| 0 \| 7 \| \| RPL3 \| 0 \| -2.110793889 \| 0 \| 7 \| \| RPS24 \| 0 \| -2.12400281 \| 0 \| 7 \| \| RPS20 \| 0 \| -2.124211997 \| 0 \| 7 \| \| RPS27A \| 0 \| -2.142064894 \| 0 \| 7 \| \| RPS23 \| 0 \| -2.158495559 \| 0 \| 7 \| \| RPS4X \| 0 \| -2.166513807 \| 0 \| 7 \| \| RPL21 \| 0 \| -2.169038573 \| 0 \| 7 \| \| RPS3A \| 0 \| -2.223531675 \| 0 \| 7 \| \| RPSA \| 0 \| -2.230815938 \| 0 \| 7 \| \| TSC22D3 \| 0 \| -2.233315296 \| 0 \| 7 \| \| RPS19 \| 0 \| -2.248194105 \| 0 \| 7 \| \| RPS18 \| 0 \| -2.258409429 \| 0 \| 7 \| \| RPS15A \| 0 \| -2.287081821 \| 0 \| 7 \| \| RPL37 \| 0 \| -2.303983803 \| 0 \| 7 \| \| RPLP1 \| 0 \| -2.317505815 \| 0 \| 7 \| \| RPL5 \| 0 \| -2.344229788 \| 0 \| 7 \| \| RPS7 \| 0 \| -2.37575737 \| 0 \| 7 \| \| RPS12 \| 0 \| -2.388593692 \| 0 \| 7 \| \| RPL32 \| 0 \| -2.4044701 \| 0 \| 7 \| \| RPS6 \| 0 \| -2.412449661 \| 0 \| 7 \| \| RPL13 \| 0 \| -2.412677851 \| 0 \| 7 \| \| RPL6 \| 0 \| -2.444706454 \| 0 \| 7 \| \| RPS8 \| 0 \| -2.479751122 \| 0 \| 7 \| \| RPL10A \| 0 \| -2.551054195 \| 0 \| 7 \| \| RPS2 \| 0 \| -2.603759734 \| 0 \| 7 \| \| SRGN \| 0 \| -2.867523642 \| 0 \| 7 \| \| VIM \| 0 \| -3.016676059 \| 0 \| 7 \| \| USH1C \| 9.9746E-306 \| 1.030158985 \| 2.1772E-301 \| 7 \| \| PTMA \| 8.1006E-303 \| -1.150536953 \| 1.7681E-298 \| 7 \| \| ADIRF \| 1.5606E-302 \| 1.501015233 \| 3.4062E-298 \| 7 \| \| MUC3A \| 2.2207E-302 \| 1.083419918 \| 4.8471E-298 \| 7 \| \| GBP3 \| 5.1503E-299 \| 1.097903967 \| 1.1242E-294 \| 7 \| \| FBXW5 \| 9.3806E-299 \| 1.259877995 \| 2.0475E-294 \| 7 \| \| HSP90AA1 \| 1.1979E-298 \| -1.525493932 \| 2.6147E-294 \| 7 \| \| CD44 \| 3.0379E-298 \| -1.579321977 \| 6.6307E-294 \| 7 \| \| EIF3E \| 1.069E-297 \| -1.407804438 \| 2.3332E-293 \| 7 \| \| OASL \| 3.5908E-297 \| 1.041093901 \| 7.8375E-293 \| 7 \| \| MPST \| 6.077E-296 \| 1.245008711 \| 1.3264E-291 \| 7 \| \| ZZEF1 \| 7.147E-296 \| 1.108361714 \| 1.56E-291 \| 7 \| \| RND3 \| 1.6571E-295 \| 1.13464049 \| 3.6169E-291 \| 7 \| \| CAPN2 \| 5.7203E-293 \| 1.19227186 \| 1.2486E-288 \| 7 \| \| CHMP4B \| 8.6903E-293 \| 1.250432485 \| 1.8968E-288 \| 7 \| \| TMEM176A \| 1.1037E-291 \| 1.047799966 \| 2.4091E-287 \| 7 \| \| SYTL2 \| 1.2668E-291 \| 1.028975604 \| 2.765E-287 \| 7 \| \| PRDX6 \| 6.4273E-290 \| 1.575634987 \| 1.4029E-285 \| 7 \| \| TSC22D1 \| 3.8603E-289 \| 1.207677494 \| 8.426E-285 \| 7 \| \| FBXO32 \| 9.3793E-289 \| 1.180861766 \| 2.0472E-284 \| 7 \| \| GSKIP \| 3.5211E-288 \| 1.090648357 \| 7.6856E-284 \| 7 \| \| ANXA4 \| 1.5486E-285 \| 1.155153509 \| 3.38E-281 \| 7 \| \| PDK4 \| 2.3755E-285 \| 1.119325848 \| 5.185E-281 \| 7 \| \| SHROOM3 \| 6.4307E-284 \| 1.028019921 \| 1.4036E-279 \| 7 \| \| PRR15L \| 3.6197E-281 \| 1.027514259 \| 7.9007E-277 \| 7 \| \| ERBIN \| 1.8213E-280 \| 1.311104405 \| 3.9754E-276 \| 7 \| \| P2RX4 \| 2.4408E-280 \| 1.053634474 \| 5.3274E-276 \| 7 \| \| LDHB \| 2.5907E-280 \| -1.486768742 \| 5.6548E-276 \| 7 \| \| KIF13B \| 4.7143E-280 \| 1.14586154 \| 1.029E-275 \| 7 \| \| RPL39 \| 8.8097E-279 \| -1.55617616 \| 1.9229E-274 \| 7 \| \| TMPRSS4 \| 6.2679E-278 \| 1.031105922 \| 1.3681E-273 \| 7 \| \| COX5B \| 3.7242E-277 \| 1.203197989 \| 8.1288E-273 \| 7 \| \| HLA-A \| 1.2523E-276 \| 1.09425521 \| 2.7334E-272 \| 7 \| \| ABHD3 \| 1.4652E-272 \| 1.120183957 \| 3.1981E-268 \| 7 \| \| IL18 \| 1.7148E-272 \| 1.109114036 \| 3.7429E-268 \| 7 \| \| RRAS \| 8.4914E-271 \| 1.137908219 \| 1.8534E-266 \| 7 \| \| PTPRC \| 2.3012E-268 \| -2.188662506 \| 5.0229E-264 \| 7 \| \| HIST1H2AC \| 3.7077E-266 \| 1.229077896 \| 8.0929E-262 \| 7 \| \| EDF1 \| 3.3257E-264 \| 1.001604128 \| 7.259E-260 \| 7 \| \| S100A4 \| 8.6677E-264 \| -2.469683059 \| 1.8919E-259 \| 7 \| \| PINK1 \| 1.1368E-262 \| 1.110536767 \| 2.4812E-258 \| 7 \| \| DYRK2 \| 1.0184E-261 \| 1.096973579 \| 2.2228E-257 \| 7 \| \| APLP2 \| 5.4128E-259 \| 1.106323146 \| 1.1814E-254 \| 7 \| \| SLC20A1 \| 2.3539E-256 \| 1.586834171 \| 5.1378E-252 \| 7 \| \| TMEM59 \| 7.0905E-256 \| 1.101570938 \| 1.5476E-251 \| 7 \| \| CXCR4 \| 6.4557E-255 \| -2.847457744 \| 1.4091E-250 \| 7 \| \| WASL \| 1.265E-254 \| 1.14107254 \| 2.761E-250 \| 7 \| \| LGALS2 \| 3.444E-254 \| 1.035859303 \| 7.5173E-250 \| 7 \| \| MT1M \| 5.747E-253 \| 1.680081564 \| 1.2544E-248 \| 7 \| \| TMEM8A \| 2.4361E-252 \| 1.078988349 \| 5.3173E-248 \| 7 \| \| ACSL5 \| 9.7099E-252 \| 1.113755935 \| 2.1194E-247 \| 7 \| \| ARHGDIB \| 3.8287E-250 \| -1.841850887 \| 8.3569E-246 \| 7 \| \| GSDMD \| 2.5056E-249 \| 1.199956284 \| 5.4689E-245 \| 7 \| \| DDX21 \| 1.4867E-248 \| -1.351199966 \| 3.2451E-244 \| 7 \| \| CD24 \| 7.1976E-248 \| 1.136249949 \| 1.571E-243 \| 7 \| \| BCL10 \| 1.7279E-246 \| 1.148781543 \| 3.7715E-242 \| 7 \| \| MT1E \| 9.7995E-246 \| 1.643682659 \| 2.1389E-241 \| 7 \| \| CTNNA1 \| 9.7604E-245 \| 1.169358178 \| 2.1304E-240 \| 7 \| \| MYADM \| 4.3148E-244 \| -1.533883 \| 9.4179E-240 \| 7 \| \| ACTN4 \| 1.4619E-241 \| 1.059237447 \| 3.1908E-237 \| 7 \| \| SLC9A3R1 \| 2.1043E-241 \| 1.192529916 \| 4.5931E-237 \| 7 \| \| MGAT4B \| 7.0738E-238 \| 1.125651611 \| 1.544E-233 \| 7 \| \| UPP1 \| 2.3953E-235 \| 1.174771862 \| 5.2283E-231 \| 7 \| \| NUCKS1 \| 4.3826E-235 \| -1.333509403 \| 9.566E-231 \| 7 \| \| PLIN3 \| 3.2134E-234 \| 1.244324773 \| 7.0138E-230 \| 7 \| \| FEM1C \| 1.035E-231 \| 1.073422756 \| 2.259E-227 \| 7 \| \| ETNK1 \| 5.2093E-228 \| 1.864747203 \| 1.137E-223 \| 7 \| \| ELOB \| 1.7086E-227 \| 1.008700149 \| 3.7293E-223 \| 7 \| \| PYGB \| 2.6808E-227 \| 1.043413172 \| 5.8514E-223 \| 7 \| \| CD74 \| 3.9283E-226 \| -2.560709223 \| 8.5743E-222 \| 7 \| \| UACA \| 4.5771E-226 \| 1.032808586 \| 9.9904E-222 \| 7 \| \| F11R \| 1.0238E-224 \| 1.059417154 \| 2.2346E-220 \| 7 \| \| UQCRQ \| 8.9379E-224 \| 1.195687671 \| 1.9509E-219 \| 7 \| \| ZG16 \| 7.7765E-223 \| 1.170445999 \| 1.6974E-218 \| 7 \| \| RPL22L1 \| 7.947E-221 \| -1.168888766 \| 1.7346E-216 \| 7 \| \| KLF3 \| 2.8526E-220 \| 1.046402986 \| 6.2263E-216 \| 7 \| \| EMP3 \| 3.5349E-220 \| -1.429961899 \| 7.7156E-216 \| 7 \| \| NR4A2 \| 6.6936E-218 \| -1.924471642 \| 1.461E-213 \| 7 \| \| CDKN1A \| 1.4092E-217 \| 1.110038331 \| 3.0758E-213 \| 7 \| \| PARK7 \| 9.2443E-217 \| -1.133504758 \| 2.0178E-212 \| 7 \| \| TOMM7 \| 2.7023E-216 \| -1.021738139 \| 5.8982E-212 \| 7 \| \| ARL4A \| 9.2472E-215 \| 1.065442771 \| 2.0184E-210 \| 7 \| \| CYTIP \| 7.5287E-212 \| -1.589713172 \| 1.6433E-207 \| 7 \| \| ARID5B \| 7.0533E-211 \| -1.478045033 \| 1.5395E-206 \| 7 \| \| RPL36A \| 1.7145E-210 \| -1.173390398 \| 3.7423E-206 \| 7 \| \| GNAI2 \| 9.3452E-210 \| -1.362991422 \| 2.0398E-205 \| 7 \| \| SET \| 2.5544E-209 \| -1.160383476 \| 5.5756E-205 \| 7 \| \| SDC4 \| 8.0347E-209 \| 1.083102641 \| 1.7537E-204 \| 7 \| \| HSPE1 \| 1.3208E-206 \| -1.328871417 \| 2.8829E-202 \| 7 \| \| CD99 \| 3.1228E-204 \| -1.251453365 \| 6.8161E-200 \| 7 \| \| RGS1 \| 2.0968E-201 \| -1.956813854 \| 4.5768E-197 \| 7 \| \| HSPA8 \| 8.3829E-201 \| -1.207053694 \| 1.8297E-196 \| 7 \| \| GTF3A \| 1.1153E-199 \| -1.019968895 \| 2.4343E-195 \| 7 \| \| DUSP2 \| 3.2012E-199 \| -1.937830219 \| 6.9873E-195 \| 7 \| \| SMAP2 \| 7.7154E-199 \| -1.486463481 \| 1.684E-194 \| 7 \| \| MYCBP2 \| 3.0359E-198 \| -1.108778296 \| 6.6265E-194 \| 7 \| \| CD48 \| 4.8261E-197 \| -1.586607685 \| 1.0534E-192 \| 7 \| \| TOMM20 \| 1.685E-194 \| -1.026485793 \| 3.6778E-190 \| 7 \| \| KTN1 \| 6.907E-193 \| 1.116209003 \| 1.5076E-188 \| 7 \| \| GMFG \| 9.6E-192 \| -1.379343957 \| 2.0954E-187 \| 7 \| \| FXYD5 \| 1.9609E-190 \| -1.258499213 \| 4.2801E-186 \| 7 \| \| HNRNPDL \| 1.4288E-189 \| -1.003312792 \| 3.1187E-185 \| 7 \| \| HSPH1 \| 6.4366E-188 \| -1.611146293 \| 1.4049E-183 \| 7 \| \| ZFAS1 \| 6.3042E-187 \| -1.223439447 \| 1.376E-182 \| 7 \| \| DDX18 \| 1.1433E-186 \| -1.02421376 \| 2.4954E-182 \| 7 \| \| LAPTM5 \| 1.6876E-186 \| -1.925033284 \| 3.6835E-182 \| 7 \| \| ADM \| 2.8161E-186 \| 1.146069104 \| 6.1467E-182 \| 7 \| \| PABPC1 \| 4.683E-186 \| -1.20173706 \| 1.0222E-181 \| 7 \| \| CD52 \| 1.9219E-185 \| -2.027242462 \| 4.195E-181 \| 7 \| \| PKN2 \| 3.1807E-185 \| 1.002295801 \| 6.9425E-181 \| 7 \| \| ANXA5 \| 7.9407E-185 \| -1.260797529 \| 1.7332E-180 \| 7 \| \| RSL1D1 \| 9.2761E-185 \| -1.0276229 \| 2.0247E-180 \| 7 \| \| EIF3L \| 2.9281E-184 \| -1.012360978 \| 6.3911E-180 \| 7 \| \| H2AFZ \| 6.671E-180 \| -1.24354879 \| 1.4561E-175 \| 7 \| \| DNAJA1 \| 3.8056E-179 \| -1.56631154 \| 8.3065E-175 \| 7 \| \| SSR4 \| 1.4687E-178 \| -1.355302474 \| 3.2058E-174 \| 7 \| \| PLSCR1 \| 1.8485E-178 \| 1.065256253 \| 4.0347E-174 \| 7 \| \| CCND2 \| 1.0877E-177 \| -1.263467551 \| 2.3742E-173 \| 7 \| \| BST2 \| 1.469E-176 \| -1.145998758 \| 3.2064E-172 \| 7 \| \| COMMD6 \| 1.8289E-176 \| -1.072452379 \| 3.9919E-172 \| 7 \| \| HNRNPA1 \| 1.3976E-174 \| -1.00675957 \| 3.0506E-170 \| 7 \| \| RGCC \| 2.3895E-174 \| -1.760410685 \| 5.2157E-170 \| 7 \| \| RAN \| 4.1754E-172 \| -1.057671679 \| 9.1137E-168 \| 7 \| \| CCDC85B \| 8.0005E-170 \| -1.016781802 \| 1.7463E-165 \| 7 \| \| CD53 \| 4.2289E-169 \| -1.359004439 \| 9.2304E-165 \| 7 \| \| LGALS1 \| 8.3298E-167 \| -2.018694873 \| 1.8182E-162 \| 7 \| \| NR4A1 \| 3.5511E-166 \| -1.236147503 \| 7.751E-162 \| 7 \| \| CELF2 \| 4.2317E-166 \| -1.177158144 \| 9.2366E-162 \| 7 \| \| RAC2 \| 1.9306E-164 \| -1.237058623 \| 4.214E-160 \| 7 \| \| FOSB \| 9.203E-163 \| -1.215014034 \| 2.0087E-158 \| 7 \| \| RGS2 \| 1.2423E-162 \| -1.630611011 \| 2.7115E-158 \| 7 \| \| LCP1 \| 1.308E-162 \| -1.639407153 \| 2.855E-158 \| 7 \| \| CD37 \| 2.3229E-161 \| -1.503293918 \| 5.0703E-157 \| 7 \| \| STK24 \| 1.9536E-157 \| 1.00062769 \| 4.264E-153 \| 7 \| \| ZNF331 \| 1.1325E-154 \| -1.744570119 \| 2.4719E-150 \| 7 \| \| XBP1 \| 1.4255E-152 \| -1.137241087 \| 3.1115E-148 \| 7 \| \| CREM \| 2.2095E-151 \| -1.720938002 \| 4.8227E-147 \| 7 \| \| TUBA1A \| 4.2528E-149 \| -1.465363862 \| 9.2826E-145 \| 7 \| \| FYB1 \| 2.0064E-146 \| -1.62071597 \| 4.3794E-142 \| 7 \| \| LSP1 \| 6.8081E-146 \| -1.175174953 \| 1.486E-141 \| 7 \| \| BTG2 \| 4.1626E-145 \| -1.074043269 \| 9.0858E-141 \| 7 \| \| CD69 \| 2.4004E-144 \| -2.089290389 \| 5.2395E-140 \| 7 \| \| MSN \| 1.1868E-142 \| -1.168542382 \| 2.5903E-138 \| 7 \| \| RGS10 \| 6.7712E-141 \| -1.071203636 \| 1.478E-136 \| 7 \| \| AC058791.1 \| 7.8681E-141 \| -1.262537738 \| 1.7174E-136 \| 7 \| \| SQSTM1 \| 5.8984E-139 \| 1.060118712 \| 1.2874E-134 \| 7 \| \| RPS4Y1 \| 1.7883E-138 \| -1.206005093 \| 3.9034E-134 \| 7 \| \| GPR183 \| 1.6463E-136 \| -1.800198144 \| 3.5934E-132 \| 7 \| \| SELENOM \| 2.2925E-136 \| -1.057527983 \| 5.0038E-132 \| 7 \| \| CACYBP \| 9.4525E-136 \| -1.194578936 \| 2.0632E-131 \| 7 \| \| PNRC1 \| 1.5316E-135 \| -1.064153727 \| 3.3431E-131 \| 7 \| \| HCST \| 2.7917E-134 \| -1.43114896 \| 6.0935E-130 \| 7 \| \| ARL4C \| 3.73E-134 \| -1.497331647 \| 8.1414E-130 \| 7 \| \| DNAJB1 \| 6.1936E-133 \| -1.61413262 \| 1.3519E-128 \| 7 \| \| GLIPR1 \| 1.3116E-131 \| -1.122946233 \| 2.8628E-127 \| 7 \| \| MT1G \| 2.6697E-131 \| 2.029154351 \| 5.8272E-127 \| 7 \| \| SAMSN1 \| 3.5839E-131 \| -1.410209402 \| 7.8226E-127 \| 7 \| \| SLC2A3 \| 4.9698E-130 \| -1.381980735 \| 1.0848E-125 \| 7 \| \| IFI16 \| 1.6892E-129 \| -1.004820092 \| 3.6871E-125 \| 7 \| \| HLA-DPA1 \| 8.558E-128 \| -2.354338975 \| 1.868E-123 \| 7 \| \| COTL1 \| 6.0808E-127 \| -1.364059669 \| 1.3272E-122 \| 7 \| \| CLEC2B \| 1.1115E-126 \| -1.256609534 \| 2.426E-122 \| 7 \| \| FKBP11 \| 2.3023E-125 \| -1.026251575 \| 5.0252E-121 \| 7 \| \| HERPUD1 \| 5.6103E-125 \| -1.13049091 \| 1.2246E-120 \| 7 \| \| UBE2S \| 8.5773E-124 \| -1.041326586 \| 1.8722E-119 \| 7 \| \| SOCS3 \| 2.4094E-123 \| -1.047644141 \| 5.259E-119 \| 7 \| \| PDE4B \| 1.6824E-122 \| -1.046638895 \| 3.6723E-118 \| 7 \| \| TNFAIP3 \| 2.6959E-121 \| -1.726845329 \| 5.8844E-117 \| 7 \| \| REL \| 1.2156E-120 \| -1.413067229 \| 2.6533E-116 \| 7 \| \| TENT5C \| 4.4617E-120 \| -1.002960933 \| 9.7386E-116 \| 7 \| \| PTMS \| 8.8763E-118 \| -1.033834717 \| 1.9374E-113 \| 7 \| \| IL7R \| 1.2464E-115 \| -2.31485745 \| 2.7204E-111 \| 7 \| \| TRAC \| 3.6446E-114 \| -1.635251641 \| 7.9551E-110 \| 7 \| \| CD3D \| 1.5515E-112 \| -1.729636854 \| 3.3864E-108 \| 7 \| \| CD2 \| 2.9759E-111 \| -1.67272366 \| 6.4954E-107 \| 7 \| \| HLA-DPB1 \| 4.5179E-108 \| -2.331172723 \| 9.8612E-104 \| 7 \| \| CNOT6L \| 1.8433E-107 \| -1.05133707 \| 4.0234E-103 \| 7 \| \| TAGAP \| 2.0597E-107 \| -1.059985363 \| 4.4957E-103 \| 7 \| \| SERPINB9 \| 7.0969E-107 \| -1.001458474 \| 1.549E-102 \| 7 \| \| RHOH \| 1.3994E-106 \| -1.034021928 \| 3.0544E-102 \| 7 \| \| MT2A \| 4.0607E-101 \| 1.673201925 \| 8.86338E-97 \| 7 \| \| ANXA1 \| 3.57664E-96 \| -1.442577683 \| 7.80673E-92 \| 7 \| \| BTG1 \| 1.21923E-91 \| -1.307234606 \| 2.66121E-87 \| 7 \| \| SLC12A2 \| 7.3548E-84 \| -1.173697031 \| 1.60533E-79 \| 7 \| \| JUNB \| 1.13194E-78 \| -1.094727985 \| 2.47068E-74 \| 7 \| \| TIMP1 \| 1.00995E-72 \| -1.589436389 \| 2.20441E-68 \| 7 \| \| KLF2 \| 5.66606E-64 \| -1.126931896 \| 1.23673E-59 \| 7 \| \| HSPA1A \| 1.31264E-60 \| -1.872170596 \| 2.8651E-56 \| 7 \| \| HSPA1B \| 1.47324E-51 \| -1.185934992 \| 3.21565E-47 \| 7 \| \| HLA-DRA \| 2.7281E-48 \| -2.889779827 \| 5.95463E-44 \| 7 \| \| LYZ \| 2.79372E-38 \| -2.667523568 \| 6.09786E-34 \| 7 \| \| CENPF \| 0 \| 3.15018066 \| 0 \| 8 \| \| MKI67 \| 0 \| 3.071003792 \| 0 \| 8 \| \| TOP2A \| 0 \| 2.592568571 \| 0 \| 8 \| \| PCCA \| 0 \| 1.808049464 \| 0 \| 8 \| \| HMGB1 \| 0 \| 1.724103432 \| 0 \| 8 \| \| GPX2 \| 0 \| 1.667077158 \| 0 \| 8 \| \| RNF43 \| 0 \| 1.653421843 \| 0 \| 8 \| \| TPX2 \| 0 \| 1.599165804 \| 0 \| 8 \| \| LCN15 \| 0 \| 1.581893871 \| 0 \| 8 \| \| SLC12A2 \| 0 \| 1.575170986 \| 0 \| 8 \| \| HMGA1 \| 0 \| 1.574372053 \| 0 \| 8 \| \| ATAD5 \| 0 \| 1.56119317 \| 0 \| 8 \| \| DSP \| 0 \| 1.54315301 \| 0 \| 8 \| \| CENPE \| 0 \| 1.45481824 \| 0 \| 8 \| \| SOX9 \| 0 \| 1.454132006 \| 0 \| 8 \| \| PTMS \| 0 \| 1.450354402 \| 0 \| 8 \| \| GSTP1 \| 0 \| 1.450336849 \| 0 \| 8 \| \| CENPJ \| 0 \| 1.447785652 \| 0 \| 8 \| \| KRT18 \| 0 \| 1.441756649 \| 0 \| 8 \| \| WDR34 \| 0 \| 1.438823122 \| 0 \| 8 \| \| BRCA2 \| 0 \| 1.438283496 \| 0 \| 8 \| \| ASPM \| 0 \| 1.434561372 \| 0 \| 8 \| \| BIRC5 \| 0 \| 1.409039098 \| 0 \| 8 \| \| SOX4 \| 0 \| 1.379939463 \| 0 \| 8 \| \| TUBA1B \| 0 \| 1.377986754 \| 0 \| 8 \| \| SCD \| 0 \| 1.364578258 \| 0 \| 8 \| \| ENO1 \| 0 \| 1.362907101 \| 0 \| 8 \| \| DEK \| 0 \| 1.356242377 \| 0 \| 8 \| \| CCNB1 \| 0 \| 1.351737666 \| 0 \| 8 \| \| EIF3B \| 0 \| 1.337309862 \| 0 \| 8 \| \| IGFBP2 \| 0 \| 1.333553112 \| 0 \| 8 \| \| FERMT1 \| 0 \| 1.331668 \| 0 \| 8 \| \| GOLIM4 \| 0 \| 1.328647424 \| 0 \| 8 \| \| HMGB3 \| 0 \| 1.32709228 \| 0 \| 8 \| \| PTTG1 \| 0 \| 1.316237561 \| 0 \| 8 \| \| GGH \| 0 \| 1.306022613 \| 0 \| 8 \| \| DKC1 \| 0 \| 1.299313056 \| 0 \| 8 \| \| CYC1 \| 0 \| 1.29138938 \| 0 \| 8 \| \| MCM7 \| 0 \| 1.288475014 \| 0 \| 8 \| \| CDC20 \| 0 \| 1.277335625 \| 0 \| 8 \| \| CDX2 \| 0 \| 1.272215083 \| 0 \| 8 \| \| TRAP1 \| 0 \| 1.271044555 \| 0 \| 8 \| \| HSPD1 \| 0 \| 1.266441143 \| 0 \| 8 \| \| KNOP1 \| 0 \| 1.264706771 \| 0 \| 8 \| \| SMC1A \| 0 \| 1.261981267 \| 0 \| 8 \| \| FASN \| 0 \| 1.259613293 \| 0 \| 8 \| \| CD24 \| 0 \| 1.258892958 \| 0 \| 8 \| \| PHB \| 0 \| 1.256766105 \| 0 \| 8 \| \| HMGB2 \| 0 \| 1.245098797 \| 0 \| 8 \| \| GAPDH \| 0 \| 1.244598533 \| 0 \| 8 \| \| MLEC \| 0 \| 1.223814876 \| 0 \| 8 \| \| HES1 \| 0 \| 1.214280572 \| 0 \| 8 \| \| NOP56 \| 0 \| 1.212254744 \| 0 \| 8 \| \| MCM4 \| 0 \| 1.211313354 \| 0 \| 8 \| \| FAT1 \| 0 \| 1.209181643 \| 0 \| 8 \| \| SURF6 \| 0 \| 1.205130904 \| 0 \| 8 \| \| NUCKS1 \| 0 \| 1.204952808 \| 0 \| 8 \| \| PAICS \| 0 \| 1.203855183 \| 0 \| 8 \| \| HNRNPAB \| 0 \| 1.201010207 \| 0 \| 8 \| \| CCDC34 \| 0 \| 1.198224897 \| 0 \| 8 \| \| RPL35 \| 0 \| 1.195707017 \| 0 \| 8 \| \| PRKDC \| 0 \| 1.190808637 \| 0 \| 8 \| \| S100A14 \| 0 \| 1.183725723 \| 0 \| 8 \| \| LCN2 \| 0 \| 1.182939436 \| 0 \| 8 \| \| GGCT \| 0 \| 1.180022905 \| 0 \| 8 \| \| ECT2 \| 0 \| 1.176799201 \| 0 \| 8 \| \| UBE2C \| 0 \| 1.173788528 \| 0 \| 8 \| \| SET \| 0 \| 1.165343489 \| 0 \| 8 \| \| CDCA7 \| 0 \| 1.162245175 \| 0 \| 8 \| \| PHLDA2 \| 0 \| 1.159522833 \| 0 \| 8 \| \| FBL \| 0 \| 1.159345463 \| 0 \| 8 \| \| MCM3 \| 0 \| 1.150856909 \| 0 \| 8 \| \| GDF15 \| 0 \| 1.146130935 \| 0 \| 8 \| \| VIL1 \| 0 \| 1.145403503 \| 0 \| 8 \| \| PSMB7 \| 0 \| 1.143590091 \| 0 \| 8 \| \| CENPX \| 0 \| 1.139578468 \| 0 \| 8 \| \| DPEP1 \| 0 \| 1.137762843 \| 0 \| 8 \| \| MRPS34 \| 0 \| 1.136686406 \| 0 \| 8 \| \| PKP3 \| 0 \| 1.135263667 \| 0 \| 8 \| \| TPI1 \| 0 \| 1.134019887 \| 0 \| 8 \| \| ASCL2 \| 0 \| 1.128338411 \| 0 \| 8 \| \| PERP \| 0 \| 1.126215888 \| 0 \| 8 \| \| NCL \| 0 \| 1.124914981 \| 0 \| 8 \| \| RABL6 \| 0 \| 1.121586128 \| 0 \| 8 \| \| POLD2 \| 0 \| 1.118818373 \| 0 \| 8 \| \| TXN \| 0 \| 1.117665291 \| 0 \| 8 \| \| PLCB4 \| 0 \| 1.117624961 \| 0 \| 8 \| \| IPO5 \| 0 \| 1.117394768 \| 0 \| 8 \| \| TUBB \| 0 \| 1.117171449 \| 0 \| 8 \| \| XRCC2 \| 0 \| 1.116430832 \| 0 \| 8 \| \| ATP5MC1 \| 0 \| 1.111028276 \| 0 \| 8 \| \| TIMM13 \| 0 \| 1.109261152 \| 0 \| 8 \| \| SRPK1 \| 0 \| 1.107449616 \| 0 \| 8 \| \| KIF20B \| 0 \| 1.100927234 \| 0 \| 8 \| \| BRI3BP \| 0 \| 1.099770755 \| 0 \| 8 \| \| RANBP1 \| 0 \| 1.098783986 \| 0 \| 8 \| \| MYC \| 0 \| 1.092235607 \| 0 \| 8 \| \| EPCAM \| 0 \| 1.091632932 \| 0 \| 8 \| \| PCNA \| 0 \| 1.090086913 \| 0 \| 8 \| \| ATP5MC3 \| 0 \| 1.086368198 \| 0 \| 8 \| \| TOMM40 \| 0 \| 1.085919469 \| 0 \| 8 \| \| PPP1R1B \| 0 \| 1.070445267 \| 0 \| 8 \| \| SMC4 \| 0 \| 1.070406399 \| 0 \| 8 \| \| SNRPF \| 0 \| 1.069433677 \| 0 \| 8 \| \| HMMR \| 0 \| 1.067871153 \| 0 \| 8 \| \| EHF \| 0 \| 1.066290855 \| 0 \| 8 \| \| AHCY \| 0 \| 1.065451049 \| 0 \| 8 \| \| TK1 \| 0 \| 1.064831703 \| 0 \| 8 \| \| CCT5 \| 0 \| 1.062613193 \| 0 \| 8 \| \| CKAP5 \| 0 \| 1.058274922 \| 0 \| 8 \| \| ATP5F1B \| 0 \| 1.055769976 \| 0 \| 8 \| \| HELLS \| 0 \| 1.052639797 \| 0 \| 8 \| \| S100P \| 0 \| 1.051547987 \| 0 \| 8 \| \| LRPPRC \| 0 \| 1.051409933 \| 0 \| 8 \| \| EIF4G1 \| 0 \| 1.050376977 \| 0 \| 8 \| \| PKM \| 0 \| 1.045193964 \| 0 \| 8 \| \| NHP2 \| 0 \| 1.033080156 \| 0 \| 8 \| \| JUP \| 0 \| 1.032269503 \| 0 \| 8 \| \| HIST1H1E \| 0 \| 1.031620901 \| 0 \| 8 \| \| CBX5 \| 0 \| 1.02479029 \| 0 \| 8 \| \| TRIM2 \| 0 \| 1.024056481 \| 0 \| 8 \| \| SMC2 \| 0 \| 1.013843508 \| 0 \| 8 \| \| CCT7 \| 0 \| 1.012423255 \| 0 \| 8 \| \| TKT \| 0 \| 1.011451216 \| 0 \| 8 \| \| ITGA6 \| 0 \| 1.007312845 \| 0 \| 8 \| \| TUFM \| 0 \| 1.005789217 \| 0 \| 8 \| \| PHGR1 \| 0 \| -1.21649703 \| 0 \| 8 \| \| TSC22D3 \| 0 \| -2.746099499 \| 0 \| 8 \| \| VIM \| 0 \| -3.649419862 \| 0 \| 8 \| \| SRGN \| 0 \| -3.844644944 \| 0 \| 8 \| \| DUSP1 \| 4.7398E-308 \| -1.984535151 \| 1.0346E-303 \| 8 \| \| JUND \| 3.6883E-304 \| -1.486256709 \| 8.0505E-300 \| 8 \| \| PTPRC \| 4.8225E-302 \| -2.714957351 \| 1.0526E-297 \| 8 \| \| JUNB \| 9.4317E-267 \| -1.764799157 \| 2.0586E-262 \| 8 \| \| B2M \| 1.7733E-251 \| -1.587727302 \| 3.8706E-247 \| 8 \| \| RGS1 \| 1.214E-242 \| -2.663630831 \| 2.6497E-238 \| 8 \| \| ARHGDIB \| 2.3382E-233 \| -2.097851056 \| 5.1035E-229 \| 8 \| \| CYTIP \| 4.0781E-231 \| -1.79571056 \| 8.9012E-227 \| 8 \| \| BTG1 \| 6.1385E-231 \| -1.962365498 \| 1.3398E-226 \| 8 \| \| CD48 \| 1.6988E-230 \| -1.844818276 \| 3.7079E-226 \| 8 \| \| HLA-C \| 6.5486E-230 \| -1.121510642 \| 1.4294E-225 \| 8 \| \| LDHB \| 6.1533E-226 \| 1.012414749 \| 1.3431E-221 \| 8 \| \| EMP3 \| 2.6776E-224 \| -1.533961663 \| 5.8444E-220 \| 8 \| \| GMFG \| 6.6873E-224 \| -1.583003137 \| 1.4596E-219 \| 8 \| \| HLA-E \| 1.4938E-221 \| -1.290460915 \| 3.2605E-217 \| 8 \| \| CD52 \| 3.1866E-211 \| -2.54496144 \| 6.9553E-207 \| 8 \| \| TXNIP \| 2.5303E-204 \| -1.934590713 \| 5.5229E-200 \| 8 \| \| TNFAIP3 \| 2.2065E-202 \| -2.737248936 \| 4.8162E-198 \| 8 \| \| FOSB \| 4.8186E-191 \| -1.440717651 \| 1.0517E-186 \| 8 \| \| CXCR4 \| 1.014E-190 \| -3.273787168 \| 2.2133E-186 \| 8 \| \| HERPUD1 \| 4.7026E-184 \| -1.74355904 \| 1.0264E-179 \| 8 \| \| CD53 \| 4.4445E-183 \| -1.541255087 \| 9.7011E-179 \| 8 \| \| LAPTM5 \| 3.38E-179 \| -2.060032445 \| 7.3775E-175 \| 8 \| \| LCP1 \| 1.5131E-178 \| -1.880868704 \| 3.3027E-174 \| 8 \| \| OLFM4 \| 7.6988E-176 \| 1.621765422 \| 1.6804E-171 \| 8 \| \| PNRC1 \| 1.4052E-173 \| -1.504669426 \| 3.067E-169 \| 8 \| \| ISG20 \| 3.3024E-172 \| -1.464524738 \| 7.2081E-168 \| 8 \| \| GYPC \| 9.7484E-171 \| -1.122104144 \| 2.1278E-166 \| 8 \| \| YPEL5 \| 2.4886E-169 \| -1.555649404 \| 5.4319E-165 \| 8 \| \| CD37 \| 6.9416E-166 \| -1.62255345 \| 1.5152E-161 \| 8 \| \| CD69 \| 2.4691E-162 \| -2.571084977 \| 5.3892E-158 \| 8 \| \| SMAP2 \| 1.3153E-159 \| -1.585495978 \| 2.8709E-155 \| 8 \| \| ANKRD12 \| 4.2394E-158 \| -1.272831304 \| 9.2532E-154 \| 8 \| \| NFKBIA \| 9.7309E-158 \| -1.624096351 \| 2.124E-153 \| 8 \| \| LSP1 \| 2.2506E-157 \| -1.285909769 \| 4.9124E-153 \| 8 \| \| PRDM1 \| 6.4557E-157 \| -1.470178366 \| 1.4091E-152 \| 8 \| \| EIF1 \| 1.3198E-156 \| -1.027399764 \| 2.8807E-152 \| 8 \| \| MCL1 \| 3.6783E-156 \| -1.160279577 \| 8.0287E-152 \| 8 \| \| GPR183 \| 1.1883E-155 \| -1.997654235 \| 2.5936E-151 \| 8 \| \| ZNF331 \| 3.3348E-155 \| -1.96986003 \| 7.2789E-151 \| 8 \| \| RGCC \| 2.7779E-152 \| -1.965033076 \| 6.0633E-148 \| 8 \| \| HCST \| 6.1191E-151 \| -1.709119342 \| 1.3356E-146 \| 8 \| \| RAC2 \| 8.1053E-148 \| -1.409286294 \| 1.7691E-143 \| 8 \| \| CREM \| 3.3865E-147 \| -2.018511503 \| 7.3918E-143 \| 8 \| \| ZFP36 \| 8.9376E-146 \| -1.289220355 \| 1.9508E-141 \| 8 \| \| HLA-A \| 2.1523E-143 \| -1.018115975 \| 4.6977E-139 \| 8 \| \| CLEC2B \| 4.1334E-143 \| -1.462425281 \| 9.0221E-139 \| 8 \| \| EVI2B \| 1.3082E-141 \| -1.080308819 \| 2.8555E-137 \| 8 \| \| SELENOK \| 2.8519E-140 \| -1.216568396 \| 6.2247E-136 \| 8 \| \| IL7R \| 3.5242E-136 \| -2.979565411 \| 7.6923E-132 \| 8 \| \| TMSB4X \| 1.4972E-135 \| -1.185937059 \| 3.268E-131 \| 8 \| \| CD3D \| 1.7271E-135 \| -2.160804182 \| 3.7698E-131 \| 8 \| \| FAM107B \| 2.2561E-135 \| -1.289104777 \| 4.9243E-131 \| 8 \| \| FOS \| 3.4133E-135 \| -1.311486815 \| 7.4502E-131 \| 8 \| \| SH3BGRL3 \| 8.6023E-135 \| -1.282936217 \| 1.8776E-130 \| 8 \| \| SAMSN1 \| 1.3555E-134 \| -1.567856153 \| 2.9585E-130 \| 8 \| \| GLIPR1 \| 7.8266E-133 \| -1.265982664 \| 1.7083E-128 \| 8 \| \| CD74 \| 8.8954E-132 \| -2.461221095 \| 1.9416E-127 \| 8 \| \| HCLS1 \| 3.8103E-131 \| -1.03977662 \| 8.3168E-127 \| 8 \| \| TRAC \| 7.7755E-131 \| -1.941835104 \| 1.6972E-126 \| 8 \| \| SARAF \| 1.127E-127 \| -1.394684025 \| 2.4599E-123 \| 8 \| \| CD2 \| 3.9926E-127 \| -2.030737152 \| 8.7146E-123 \| 8 \| \| SLC2A3 \| 1.0108E-126 \| -1.480654583 \| 2.2063E-122 \| 8 \| \| RHOH \| 1.2701E-126 \| -1.197800736 \| 2.7723E-122 \| 8 \| \| MYL12A \| 1.5532E-126 \| -1.135612774 \| 3.3902E-122 \| 8 \| \| FYB1 \| 2.1317E-126 \| -1.843393574 \| 4.6529E-122 \| 8 \| \| TAGAP \| 6.0032E-124 \| -1.199857813 \| 1.3103E-119 \| 8 \| \| STK17B \| 1.2183E-123 \| -1.524954226 \| 2.6592E-119 \| 8 \| \| SOCS3 \| 6.315E-122 \| -1.253432842 \| 1.3784E-117 \| 8 \| \| LGALS1 \| 2.2513E-118 \| -2.204709075 \| 4.914E-114 \| 8 \| \| PDE4B \| 2.3778E-118 \| -1.098378299 \| 5.1901E-114 \| 8 \| \| AC058791.1 \| 3.662E-118 \| -1.55303944 \| 7.993E-114 \| 8 \| \| FNBP1 \| 1.2032E-115 \| -1.107636716 \| 2.6263E-111 \| 8 \| \| SELENOM \| 5.01E-114 \| -1.055983593 \| 1.0935E-109 \| 8 \| \| HLA-DPA1 \| 7.6531E-112 \| -2.53532494 \| 1.6704E-107 \| 8 \| \| GPSM3 \| 7.7564E-112 \| -1.027400708 \| 1.693E-107 \| 8 \| \| IFI16 \| 3.0291E-111 \| -1.120261934 \| 6.6117E-107 \| 8 \| \| KLF2 \| 8.9172E-110 \| -1.677619982 \| 1.9464E-105 \| 8 \| \| ITM2B \| 3.489E-109 \| -1.17247365 \| 7.6155E-105 \| 8 \| \| RPS4Y1 \| 6.2204E-108 \| -1.163494569 \| 1.3577E-103 \| 8 \| \| TENT5C \| 6.4127E-106 \| -1.186708494 \| 1.3997E-101 \| 8 \| \| NR4A2 \| 2.2207E-104 \| -1.86941675 \| 4.8472E-100 \| 8 \| \| TUBA1A \| 2.9335E-102 \| -1.390561891 \| 6.40287E-98 \| 8 \| \| MSN \| 3.0126E-102 \| -1.209636606 \| 6.57551E-98 \| 8 \| \| HLA-DPB1 \| 7.6323E-101 \| -2.529899661 \| 1.6659E-96 \| 8 \| \| RARRES3 \| 8.1664E-99 \| -1.244490933 \| 1.78249E-94 \| 8 \| \| FTL \| 1.36087E-98 \| -1.265169342 \| 2.97037E-94 \| 8 \| \| DUSP5 \| 1.6237E-87 \| -1.056894312 \| 3.54406E-83 \| 8 \| \| S100A4 \| 6.30417E-83 \| -2.137612942 \| 1.37601E-78 \| 8 \| \| IGKC \| 6.18028E-82 \| -5.125737202 \| 1.34897E-77 \| 8 \| \| HSPA1A \| 3.94938E-81 \| -1.600925234 \| 8.6203E-77 \| 8 \| \| MUC2 \| 7.83434E-81 \| -1.228057022 \| 1.71E-76 \| 8 \| \| IRF1 \| 1.58367E-80 \| -1.17968761 \| 3.45668E-76 \| 8 \| \| DNAJA1 \| 1.60523E-77 \| -1.415885442 \| 3.50373E-73 \| 8 \| \| GNAI2 \| 2.29408E-76 \| -1.13448284 \| 5.00729E-72 \| 8 \| \| DUSP2 \| 4.03099E-70 \| -1.651184577 \| 8.79844E-66 \| 8 \| \| ARL4C \| 1.05074E-61 \| -1.449643738 \| 2.29346E-57 \| 8 \| \| ANXA1 \| 7.46246E-55 \| -2.006629834 \| 1.62883E-50 \| 8 \| \| RGS2 \| 1.04434E-54 \| -1.21846532 \| 2.27948E-50 \| 8 \| \| FTH1 \| 5.98412E-51 \| -1.24242511 \| 1.30615E-46 \| 8 \| \| GADD45B \| 5.30519E-49 \| -1.124395195 \| 1.15796E-44 \| 8 \| \| TSPAN1 \| 3.75277E-42 \| -1.142856386 \| 8.19118E-38 \| 8 \| \| ARID5B \| 1.95617E-41 \| -1.094307324 \| 4.26973E-37 \| 8 \| \| KDM6B \| 3.28075E-41 \| -1.009653658 \| 7.16089E-37 \| 8 \| \| JCHAIN \| 2.30709E-38 \| -5.27387656 \| 5.03568E-34 \| 8 \| \| HLA-DRA \| 6.40764E-36 \| -3.296562306 \| 1.3986E-31 \| 8 \| \| CNOT6L \| 1.0945E-33 \| -1.067648513 \| 2.38896E-29 \| 8 \| \| REL \| 1.04226E-32 \| -1.458696835 \| 2.27493E-28 \| 8 \| \| IGHA1 \| 5.16694E-20 \| -4.818647219 \| 1.12779E-15 \| 8 \| \| TIMP1 \| 1.57621E-17 \| -1.015918896 \| 3.44038E-13 \| 8 \| \| SSR4 \| 7.89423E-11 \| -1.042829251 \| 1.72307E-06 \| 8 \| \| SELENOP \| 1.14587E-08 \| -1.537200449 \| 0.00025011 \| 8 \| \| IGLC2 \| 4.70179E-07 \| -4.986569072 \| 0.010262588 \| 8 \| \| MS4A1 \| 0 \| 3.505955062 \| 0 \| 9 \| \| BANK1 \| 0 \| 3.232879612 \| 0 \| 9 \| \| CD83 \| 0 \| 2.949369842 \| 0 \| 9 \| \| CD79A \| 0 \| 2.9313881 \| 0 \| 9 \| \| CD37 \| 0 \| 2.594655401 \| 0 \| 9 \| \| TNFRSF13C \| 0 \| 2.551555744 \| 0 \| 9 \| \| CD74 \| 0 \| 2.545526036 \| 0 \| 9 \| \| HLA-DRA \| 0 \| 2.529757854 \| 0 \| 9 \| \| VPREB3 \| 0 \| 2.494051657 \| 0 \| 9 \| \| IGHD \| 0 \| 2.214670306 \| 0 \| 9 \| \| HLA-DPB1 \| 0 \| 2.186839028 \| 0 \| 9 \| \| CD79B \| 0 \| 2.169054411 \| 0 \| 9 \| \| POU2F2 \| 0 \| 2.151487261 \| 0 \| 9 \| \| IRF8 \| 0 \| 1.933253676 \| 0 \| 9 \| \| HLA-DRB1 \| 0 \| 1.916872361 \| 0 \| 9 \| \| LINC01781 \| 0 \| 1.901953927 \| 0 \| 9 \| \| CXCR4 \| 0 \| 1.867766951 \| 0 \| 9 \| \| HLA-DPA1 \| 0 \| 1.854692757 \| 0 \| 9 \| \| HLA-DQA1 \| 0 \| 1.852677293 \| 0 \| 9 \| \| NR4A2 \| 0 \| 1.83904904 \| 0 \| 9 \| \| FCMR \| 0 \| 1.789425061 \| 0 \| 9 \| \| PAX5 \| 0 \| 1.778898955 \| 0 \| 9 \| \| BLK \| 0 \| 1.768213853 \| 0 \| 9 \| \| MEF2C \| 0 \| 1.753997057 \| 0 \| 9 \| \| SMAP2 \| 0 \| 1.730135947 \| 0 \| 9 \| \| KLF2 \| 0 \| 1.724147378 \| 0 \| 9 \| \| RALGPS2 \| 0 \| 1.711494465 \| 0 \| 9 \| \| CD52 \| 0 \| 1.709462358 \| 0 \| 9 \| \| LAPTM5 \| 0 \| 1.708925633 \| 0 \| 9 \| \| HLA-DQB1 \| 0 \| 1.665440496 \| 0 \| 9 \| \| REL \| 0 \| 1.625575357 \| 0 \| 9 \| \| GRASP \| 0 \| 1.613183508 \| 0 \| 9 \| \| HLA-DRB5 \| 0 \| 1.612422455 \| 0 \| 9 \| \| AFF3 \| 0 \| 1.605575621 \| 0 \| 9 \| \| 1-Mar \| 0 \| 1.603927707 \| 0 \| 9 \| \| CD22 \| 0 \| 1.578731139 \| 0 \| 9 \| \| BTG1 \| 0 \| 1.576559344 \| 0 \| 9 \| \| CCR7 \| 0 \| 1.549180346 \| 0 \| 9 \| \| BASP1 \| 0 \| 1.459747422 \| 0 \| 9 \| \| IGHM \| 0 \| 1.457114608 \| 0 \| 9 \| \| LY86 \| 0 \| 1.444058474 \| 0 \| 9 \| \| HLA-DQA2 \| 0 \| 1.430692538 \| 0 \| 9 \| \| CD69 \| 0 \| 1.419310059 \| 0 \| 9 \| \| EZR \| 0 \| 1.406763062 \| 0 \| 9 \| \| RPS27 \| 0 \| 1.398987465 \| 0 \| 9 \| \| SWAP70 \| 0 \| 1.378260153 \| 0 \| 9 \| \| FCRL1 \| 0 \| 1.37672687 \| 0 \| 9 \| \| LINC00926 \| 0 \| 1.368316037 \| 0 \| 9 \| \| CD19 \| 0 \| 1.344168374 \| 0 \| 9 \| \| LY9 \| 0 \| 1.334051046 \| 0 \| 9 \| \| SELL \| 0 \| 1.309799834 \| 0 \| 9 \| \| RPS23 \| 0 \| 1.280539081 \| 0 \| 9 \| \| LTB \| 0 \| 1.25068373 \| 0 \| 9 \| \| CD53 \| 0 \| 1.224701236 \| 0 \| 9 \| \| HLA-DMB \| 0 \| 1.220365043 \| 0 \| 9 \| \| RPS20 \| 0 \| 1.218499935 \| 0 \| 9 \| \| RPSA \| 0 \| 1.215108488 \| 0 \| 9 \| \| EEF1B2 \| 0 \| 1.211411003 \| 0 \| 9 \| \| RPL32 \| 0 \| 1.203581567 \| 0 \| 9 \| \| RPS8 \| 0 \| 1.2005127 \| 0 \| 9 \| \| RPLP2 \| 0 \| 1.190658971 \| 0 \| 9 \| \| RPL23A \| 0 \| 1.190420609 \| 0 \| 9 \| \| RPS3A \| 0 \| 1.183009683 \| 0 \| 9 \| \| RPL21 \| 0 \| 1.182680671 \| 0 \| 9 \| \| PARP15 \| 0 \| 1.182501091 \| 0 \| 9 \| \| BCAS4 \| 0 \| 1.179734757 \| 0 \| 9 \| \| EBF1 \| 0 \| 1.169334445 \| 0 \| 9 \| \| CD48 \| 0 \| 1.153161162 \| 0 \| 9 \| \| FAU \| 0 \| 1.141025132 \| 0 \| 9 \| \| RPS11 \| 0 \| 1.134095155 \| 0 \| 9 \| \| RPL34 \| 0 \| 1.121335919 \| 0 \| 9 \| \| RPL13A \| 0 \| 1.112130715 \| 0 \| 9 \| \| RPL18A \| 0 \| 1.103432248 \| 0 \| 9 \| \| RPL19 \| 0 \| 1.097765478 \| 0 \| 9 \| \| RPL9 \| 0 \| 1.096188144 \| 0 \| 9 \| \| TSC22D3 \| 0 \| 1.089123017 \| 0 \| 9 \| \| RPL30 \| 0 \| 1.083991674 \| 0 \| 9 \| \| RPL11 \| 0 \| 1.080468029 \| 0 \| 9 \| \| RPS29 \| 0 \| 1.069382869 \| 0 \| 9 \| \| RPS27A \| 0 \| 1.069031119 \| 0 \| 9 \| \| RPS13 \| 0 \| 1.040862596 \| 0 \| 9 \| \| RPS5 \| 0 \| 1.035683255 \| 0 \| 9 \| \| RPS28 \| 0 \| 1.034380644 \| 0 \| 9 \| \| RPL27 \| 0 \| 1.033030544 \| 0 \| 9 \| \| RPL10A \| 0 \| 1.028322774 \| 0 \| 9 \| \| RPL5 \| 0 \| 1.024163716 \| 0 \| 9 \| \| JUNB \| 0 \| 1.022202429 \| 0 \| 9 \| \| RASGRP2 \| 0 \| 1.015182769 \| 0 \| 9 \| \| RRBP1 \| 0 \| -1.688821169 \| 0 \| 9 \| \| NEAT1 \| 0 \| -1.802862555 \| 0 \| 9 \| \| S100A11 \| 0 \| -2.10668989 \| 0 \| 9 \| \| CD63 \| 0 \| -2.129508139 \| 0 \| 9 \| \| LGALS3 \| 0 \| -2.921877696 \| 0 \| 9 \| \| S100A6 \| 0 \| -3.352865239 \| 0 \| 9 \| \| RPS10 \| 4.6665E-305 \| 1.026264634 \| 1.0186E-300 \| 9 \| \| MIR29B2CHG \| 9.335E-300 \| 1.565668145 \| 2.0375E-295 \| 9 \| \| SNX2 \| 4.2364E-299 \| 1.487582569 \| 9.2468E-295 \| 9 \| \| S100A10 \| 9.5665E-297 \| -2.122352527 \| 2.0881E-292 \| 9 \| \| ADAM28 \| 1.8016E-294 \| 1.195936125 \| 3.9324E-290 \| 9 \| \| NFKBID \| 2.9361E-290 \| 1.439411792 \| 6.4085E-286 \| 9 \| \| TCF4 \| 5.8836E-287 \| 1.261500585 \| 1.2842E-282 \| 9 \| \| GSTP1 \| 2.8339E-286 \| -1.605666381 \| 6.1856E-282 \| 9 \| \| YPEL5 \| 1.5795E-285 \| 1.412969573 \| 3.4475E-281 \| 9 \| \| IER3 \| 1.6795E-284 \| -2.342092182 \| 3.6658E-280 \| 9 \| \| CD40 \| 9.3109E-281 \| 1.152433624 \| 2.0323E-276 \| 9 \| \| JUND \| 2.4713E-277 \| 1.033928131 \| 5.3941E-273 \| 9 \| \| GPR183 \| 7.4321E-277 \| 1.249316905 \| 1.6222E-272 \| 9 \| \| RHOH \| 1.9693E-273 \| 1.381335186 \| 4.2983E-269 \| 9 \| \| ZNF331 \| 1.6422E-272 \| 1.471152477 \| 3.5845E-268 \| 9 \| \| LYN \| 2.2943E-269 \| 1.338833194 \| 5.0077E-265 \| 9 \| \| KDM6B \| 1.3155E-268 \| 1.353342347 \| 2.8714E-264 \| 9 \| \| SP110 \| 5.3173E-267 \| 1.283277823 \| 1.1606E-262 \| 9 \| \| SMIM14 \| 2.6469E-265 \| 1.57189538 \| 5.7774E-261 \| 9 \| \| DSTN \| 2.5823E-264 \| -1.628552079 \| 5.6363E-260 \| 9 \| \| ID2 \| 3.2715E-264 \| -1.950420784 \| 7.1408E-260 \| 9 \| \| RASGEF1B \| 2.4386E-256 \| 1.455679526 \| 5.3228E-252 \| 9 \| \| RCSD1 \| 1.1859E-252 \| 1.211895833 \| 2.5885E-248 \| 9 \| \| TXN \| 1.1605E-251 \| -1.592159728 \| 2.5331E-247 \| 9 \| \| TMEM156 \| 9.7591E-249 \| 1.136790475 \| 2.1301E-244 \| 9 \| \| SLC2A3 \| 4.0756E-241 \| 1.135892272 \| 8.8958E-237 \| 9 \| \| STK17A \| 3.1836E-238 \| 1.167243103 \| 6.9487E-234 \| 9 \| \| AC004687.1 \| 1.8688E-235 \| 1.169443961 \| 4.0789E-231 \| 9 \| \| VMP1 \| 4.5244E-234 \| -1.592702734 \| 9.8754E-230 \| 9 \| \| ORAI2 \| 3.9761E-225 \| 1.395608275 \| 8.6787E-221 \| 9 \| \| CD9 \| 4.8872E-225 \| -1.969687205 \| 1.0667E-220 \| 9 \| \| P4HB \| 1.163E-222 \| -1.132389137 \| 2.5385E-218 \| 9 \| \| ITM2C \| 4.3344E-221 \| -1.840563667 \| 9.4607E-217 \| 9 \| \| ANXA2 \| 2.3033E-218 \| -1.607773219 \| 5.0275E-214 \| 9 \| \| SP140 \| 2.4695E-214 \| 1.002052774 \| 5.3901E-210 \| 9 \| \| TMEM154 \| 8.2069E-213 \| 1.074472667 \| 1.7913E-208 \| 9 \| \| IFT57 \| 7.5923E-211 \| 1.445746169 \| 1.6572E-206 \| 9 \| \| HLA-DMA \| 9.5159E-207 \| 1.082822724 \| 2.077E-202 \| 9 \| \| FKBP2 \| 3.8742E-204 \| -1.153480402 \| 8.4562E-200 \| 9 \| \| KRT8 \| 5.7289E-203 \| -3.100326257 \| 1.2504E-198 \| 9 \| \| HDLBP \| 4.2459E-201 \| -1.038570279 \| 9.2675E-197 \| 9 \| \| IFI27 \| 5.4588E-201 \| -3.10953395 \| 1.1915E-196 \| 9 \| \| TXNIP \| 1.1984E-200 \| 1.1747988 \| 2.6158E-196 \| 9 \| \| ZFAS1 \| 1.7581E-200 \| 1.133634871 \| 3.8374E-196 \| 9 \| \| KRT18 \| 1.426E-196 \| -2.969058678 \| 3.1124E-192 \| 9 \| \| RPS25 \| 3.6174E-196 \| 1.002189787 \| 7.8956E-192 \| 9 \| \| IGKC \| 5.9119E-196 \| -3.45049008 \| 1.2904E-191 \| 9 \| \| TIMP1 \| 9.2453E-195 \| -2.209942998 \| 2.018E-190 \| 9 \| \| SSR4 \| 2.599E-192 \| -1.778866225 \| 5.6728E-188 \| 9 \| \| ELF3 \| 1.4646E-190 \| -2.497735636 \| 3.1967E-186 \| 9 \| \| LGALS3BP \| 1.804E-190 \| -1.597425954 \| 3.9375E-186 \| 9 \| \| H1FX \| 3.5156E-188 \| -1.041755527 \| 7.6735E-184 \| 9 \| \| ITGA6 \| 8.6527E-186 \| -1.362526656 \| 1.8886E-181 \| 9 \| \| AL121944.1 \| 4.5887E-185 \| 1.259713437 \| 1.0016E-180 \| 9 \| \| LGALS4 \| 1.3041E-178 \| -2.97410272 \| 2.8465E-174 \| 9 \| \| KRT19 \| 1.7398E-178 \| -3.003704929 \| 3.7974E-174 \| 9 \| \| CLDN4 \| 2.0429E-178 \| -2.369211159 \| 4.4589E-174 \| 9 \| \| GAPDH \| 3.885E-178 \| -1.086013686 \| 8.4799E-174 \| 9 \| \| CLDN3 \| 8.2435E-178 \| -2.27872491 \| 1.7993E-173 \| 9 \| \| LIMA1 \| 1.7003E-176 \| -1.299488411 \| 3.7112E-172 \| 9 \| \| EPCAM \| 2.8501E-175 \| -2.243941315 \| 6.2209E-171 \| 9 \| \| XBP1 \| 1.0814E-174 \| -1.392602651 \| 2.3604E-170 \| 9 \| \| PHGR1 \| 1.9433E-172 \| -4.692281998 \| 4.2416E-168 \| 9 \| \| CEBPD \| 5.6791E-171 \| -1.596508439 \| 1.2396E-166 \| 9 \| \| VEGFA \| 3.6383E-170 \| -1.40943372 \| 7.9414E-166 \| 9 \| \| PPM1K \| 6.4508E-170 \| 1.133305247 \| 1.408E-165 \| 9 \| \| ANXA1 \| 1.6339E-168 \| -2.396584212 \| 3.5663E-164 \| 9 \| \| RHOC \| 1.0308E-167 \| -1.304715493 \| 2.2499E-163 \| 9 \| \| ELOB \| 8.0234E-166 \| -1.035345402 \| 1.7513E-161 \| 9 \| \| MT2A \| 6.2993E-165 \| -2.092913837 \| 1.3749E-160 \| 9 \| \| ETS2 \| 2.8428E-164 \| -1.309817518 \| 6.2049E-160 \| 9 \| \| UQCRQ \| 1.2661E-163 \| -1.020386396 \| 2.7635E-159 \| 9 \| \| IFITM3 \| 1.5966E-163 \| -1.792919416 \| 3.4849E-159 \| 9 \| \| BSG \| 3.1669E-163 \| -1.101481995 \| 6.9124E-159 \| 9 \| \| TPM1 \| 5.2283E-163 \| -1.491826701 \| 1.1412E-158 \| 9 \| \| DAPP1 \| 1.4334E-162 \| 1.070109397 \| 3.1286E-158 \| 9 \| \| TMEM238 \| 4.3092E-161 \| -1.209763513 \| 9.4056E-157 \| 9 \| \| ATP1B1 \| 7.1689E-161 \| -1.59398224 \| 1.5648E-156 \| 9 \| \| PRDM2 \| 1.6075E-158 \| 1.01800367 \| 3.5088E-154 \| 9 \| \| PERP \| 2.3819E-157 \| -1.491592155 \| 5.199E-153 \| 9 \| \| TNFAIP3 \| 3.3875E-156 \| -2.052536914 \| 7.3939E-152 \| 9 \| \| CAMK2N1 \| 4.1431E-156 \| -1.46351395 \| 9.0431E-152 \| 9 \| \| CDC42EP5 \| 1.9045E-155 \| -1.598339084 \| 4.1569E-151 \| 9 \| \| PTMS \| 1.4496E-151 \| -1.214570565 \| 3.1641E-147 \| 9 \| \| TMEM54 \| 2.1217E-150 \| -1.724351717 \| 4.6311E-146 \| 9 \| \| FXYD3 \| 5.3527E-150 \| -2.6912174 \| 1.1683E-145 \| 9 \| \| PRDM1 \| 2.8279E-149 \| -1.307675153 \| 6.1724E-145 \| 9 \| \| DST \| 1.3485E-148 \| -1.39126461 \| 2.9434E-144 \| 9 \| \| COX5B \| 7.5187E-148 \| -1.084795765 \| 1.6411E-143 \| 9 \| \| GNG7 \| 8.4119E-147 \| 1.105504038 \| 1.8361E-142 \| 9 \| \| TSPAN8 \| 2.3336E-146 \| -2.363795439 \| 5.0935E-142 \| 9 \| \| SMIM22 \| 1.1897E-145 \| -1.722386066 \| 2.5968E-141 \| 9 \| \| FYB1 \| 1.8245E-145 \| -1.787859855 \| 3.9823E-141 \| 9 \| \| SOX4 \| 7.7327E-145 \| -1.966069163 \| 1.6878E-140 \| 9 \| \| SPATS2L \| 2.4404E-143 \| -1.06129101 \| 5.3268E-139 \| 9 \| \| GSN \| 2.3471E-139 \| -1.755618421 \| 5.123E-135 \| 9 \| \| TMEM243 \| 3.6937E-139 \| 1.100847009 \| 8.0621E-135 \| 9 \| \| TMEM176B \| 1.1335E-138 \| -1.318803053 \| 2.474E-134 \| 9 \| \| RBM38 \| 9.0022E-138 \| 1.08114688 \| 1.9649E-133 \| 9 \| \| FKBP11 \| 1.537E-135 \| -1.174298334 \| 3.3549E-131 \| 9 \| \| SMCHD1 \| 2.3786E-135 \| 1.055167108 \| 5.1917E-131 \| 9 \| \| FCGRT \| 9.8415E-135 \| -1.097004019 \| 2.1481E-130 \| 9 \| \| SLC44A4 \| 1.0953E-134 \| -1.402887855 \| 2.3906E-130 \| 9 \| \| APP \| 2.8045E-134 \| -1.146243869 \| 6.1213E-130 \| 9 \| \| TSPAN1 \| 9.8537E-134 \| -2.266851772 \| 2.1508E-129 \| 9 \| \| AGR2 \| 2.4448E-132 \| -2.629132795 \| 5.3363E-128 \| 9 \| \| FAM3D \| 3.7389E-132 \| -1.787549588 \| 8.1608E-128 \| 9 \| \| TSC22D1 \| 4.6901E-132 \| -1.201179894 \| 1.0237E-127 \| 9 \| \| S100A14 \| 2.2484E-131 \| -1.405611784 \| 4.9076E-127 \| 9 \| \| TCF7L2 \| 2.6931E-130 \| -1.053388739 \| 5.8783E-126 \| 9 \| \| GLUL \| 1.3028E-128 \| -1.410388436 \| 2.8437E-124 \| 9 \| \| MUC13 \| 1.4941E-128 \| -1.593053101 \| 3.2612E-124 \| 9 \| \| MUC12 \| 3.0061E-126 \| -2.091051541 \| 6.5614E-122 \| 9 \| \| TRIM38 \| 2.2612E-125 \| 1.112502305 \| 4.9356E-121 \| 9 \| \| ST14 \| 4.136E-125 \| -1.134715864 \| 9.0275E-121 \| 9 \| \| PHLDA2 \| 6.4245E-125 \| -1.171590682 \| 1.4023E-120 \| 9 \| \| PIGR \| 6.4561E-125 \| -3.998647373 \| 1.4092E-120 \| 9 \| \| KLF5 \| 1.2962E-124 \| -1.327470027 \| 2.8292E-120 \| 9 \| \| TST \| 1.5679E-124 \| -1.058901433 \| 3.4223E-120 \| 9 \| \| MGLL \| 2.1938E-124 \| -1.035702457 \| 4.7884E-120 \| 9 \| \| NBL1 \| 3.0716E-124 \| -1.037060499 \| 6.7043E-120 \| 9 \| \| TMEM176A \| 8.2078E-124 \| -1.083738342 \| 1.7915E-119 \| 9 \| \| ID1 \| 5.8689E-123 \| -1.39031156 \| 1.281E-118 \| 9 \| \| ST6GAL1 \| 1.8777E-122 \| 1.11926381 \| 4.0985E-118 \| 9 \| \| POU2AF1 \| 2.2955E-122 \| 1.002382641 \| 5.0103E-118 \| 9 \| \| SELENOP \| 2.3126E-122 \| -2.428760472 \| 5.0476E-118 \| 9 \| \| DSP \| 3.7344E-121 \| -1.348512273 \| 8.1512E-117 \| 9 \| \| GPX2 \| 1.4466E-119 \| -1.654987806 \| 3.1574E-115 \| 9 \| \| IL7R \| 1.7998E-119 \| -2.588525523 \| 3.9284E-115 \| 9 \| \| CDH17 \| 3.6276E-119 \| -1.215712321 \| 7.918E-115 \| 9 \| \| NCOA3 \| 4.8146E-118 \| 1.111704723 \| 1.0509E-113 \| 9 \| \| CD3D \| 1.899E-117 \| -1.971759271 \| 4.1448E-113 \| 9 \| \| CKB \| 4.2939E-114 \| -2.200804209 \| 9.3723E-110 \| 9 \| \| PHLDA1 \| 5.7342E-114 \| -1.143613497 \| 1.2516E-109 \| 9 \| \| LCN2 \| 7.6059E-114 \| -2.408896174 \| 1.6601E-109 \| 9 \| \| CD2 \| 9.4035E-113 \| -1.858514081 \| 2.0525E-108 \| 9 \| \| STX7 \| 5.6864E-112 \| 1.165987914 \| 1.2412E-107 \| 9 \| \| KDM4B \| 2.8618E-111 \| 1.01101648 \| 6.2465E-107 \| 9 \| \| TFF3 \| 1.0284E-109 \| -3.701387086 \| 2.2447E-105 \| 9 \| \| MAP3K1 \| 5.2596E-103 \| 1.007160848 \| 1.14801E-98 \| 9 \| \| MT1X \| 1.022E-100 \| -1.203378807 \| 2.23063E-96 \| 9 \| \| ATP2B1 \| 8.3686E-98 \| 1.027064295 \| 1.82661E-93 \| 9 \| \| S100A4 \| 1.07627E-94 \| -1.693631648 \| 2.34918E-90 \| 9 \| \| DRAM2 \| 9.49189E-94 \| 1.022550501 \| 2.07179E-89 \| 9 \| \| PSAP \| 5.79292E-92 \| -1.174721832 \| 1.26442E-87 \| 9 \| \| FABP1 \| 7.00837E-89 \| -3.639175229 \| 1.52972E-84 \| 9 \| \| HES1 \| 2.86192E-83 \| -1.131857988 \| 6.24671E-79 \| 9 \| \| SLC12A2 \| 1.38772E-81 \| -1.098296099 \| 3.02898E-77 \| 9 \| \| LYZ \| 3.56865E-66 \| -2.931015784 \| 7.7893E-62 \| 9 \| \| TRAC \| 4.24281E-39 \| -1.209991356 \| 9.26078E-35 \| 9 \| \| VIM \| 1.5584E-30 \| -1.280817938 \| 3.40151E-26 \| 9 \| \| IGLC2 \| 1.51313E-26 \| -3.676138918 \| 3.30271E-22 \| 9 \| \| JCHAIN \| 9.95243E-12 \| -4.278784684 \| 2.17232E-07 \| 9 \| \| SRGN \| 4.06235E-11 \| -1.032380747 \| 8.8669E-07 \| 9 \| \| IGLC3 \| 1.46939E-10 \| -3.687459809 \| 3.20724E-06 \| 9 \| \| IGHA1 \| 1.07483E-07 \| -4.593686909 \| 0.002346038 \| 9 \| \| S100A9 \| 0 \| 5.57058416 \| 0 \| 10 \| \| S100A8 \| 0 \| 5.275735021 \| 0 \| 10 \| \| IL1B \| 0 \| 5.086094607 \| 0 \| 10 \| \| CXCL8 \| 0 \| 4.547548651 \| 0 \| 10 \| \| G0S2 \| 0 \| 4.145492065 \| 0 \| 10 \| \| EREG \| 0 \| 3.903652567 \| 0 \| 10 \| \| LYZ \| 0 \| 3.529610825 \| 0 \| 10 \| \| BCL2A1 \| 0 \| 3.403879488 \| 0 \| 10 \| \| AIF1 \| 0 \| 3.210020134 \| 0 \| 10 \| \| HLA-DRA \| 0 \| 3.118923823 \| 0 \| 10 \| \| PLEK \| 0 \| 3.100612499 \| 0 \| 10 \| \| CXCL2 \| 0 \| 3.066487877 \| 0 \| 10 \| \| TYROBP \| 0 \| 3.065221083 \| 0 \| 10 \| \| PTGS2 \| 0 \| 3.055579683 \| 0 \| 10 \| \| VCAN \| 0 \| 2.960632367 \| 0 \| 10 \| \| HLA-DPB1 \| 0 \| 2.939719537 \| 0 \| 10 \| \| HLA-DPA1 \| 0 \| 2.833612611 \| 0 \| 10 \| \| OLR1 \| 0 \| 2.652159188 \| 0 \| 10 \| \| IL1RN \| 0 \| 2.638991264 \| 0 \| 10 \| \| HLA-DRB1 \| 0 \| 2.600545192 \| 0 \| 10 \| \| MNDA \| 0 \| 2.590569349 \| 0 \| 10 \| \| NAMPT \| 0 \| 2.590559607 \| 0 \| 10 \| \| CCL3 \| 0 \| 2.578781673 \| 0 \| 10 \| \| C5AR1 \| 0 \| 2.568759261 \| 0 \| 10 \| \| TIMP1 \| 0 \| 2.558500817 \| 0 \| 10 \| \| FGL2 \| 0 \| 2.507157329 \| 0 \| 10 \| \| BASP1 \| 0 \| 2.49551552 \| 0 \| 10 \| \| CPVL \| 0 \| 2.48539707 \| 0 \| 10 \| \| LST1 \| 0 \| 2.424884818 \| 0 \| 10 \| \| S100A12 \| 0 \| 2.408948083 \| 0 \| 10 \| \| CD74 \| 0 \| 2.392697909 \| 0 \| 10 \| \| AC020656.1 \| 0 \| 2.384148112 \| 0 \| 10 \| \| HLA-DRB5 \| 0 \| 2.383145131 \| 0 \| 10 \| \| FPR1 \| 0 \| 2.342138799 \| 0 \| 10 \| \| COTL1 \| 0 \| 2.335954764 \| 0 \| 10 \| \| ACSL1 \| 0 \| 2.298535933 \| 0 \| 10 \| \| AREG \| 0 \| 2.289926217 \| 0 \| 10 \| \| FTH1 \| 0 \| 2.279333341 \| 0 \| 10 \| \| PPIF \| 0 \| 2.265642482 \| 0 \| 10 \| \| CD300E \| 0 \| 2.232878967 \| 0 \| 10 \| \| SRGN \| 0 \| 2.204700995 \| 0 \| 10 \| \| LITAF \| 0 \| 2.196595048 \| 0 \| 10 \| \| CYBB \| 0 \| 2.185511037 \| 0 \| 10 \| \| TYMP \| 0 \| 2.109880985 \| 0 \| 10 \| \| SAT1 \| 0 \| 2.084835194 \| 0 \| 10 \| \| CTSS \| 0 \| 2.082349557 \| 0 \| 10 \| \| NLRP3 \| 0 \| 2.074575249 \| 0 \| 10 \| \| NCF2 \| 0 \| 2.059402642 \| 0 \| 10 \| \| TNFAIP6 \| 0 \| 2.015458791 \| 0 \| 10 \| \| CD14 \| 0 \| 2.012908967 \| 0 \| 10 \| \| AQP9 \| 0 \| 1.998329186 \| 0 \| 10 \| \| SLC2A3 \| 0 \| 1.993405042 \| 0 \| 10 \| \| RGS2 \| 0 \| 1.991470179 \| 0 \| 10 \| \| GPR183 \| 0 \| 1.963371801 \| 0 \| 10 \| \| CCL3L1 \| 0 \| 1.9493656 \| 0 \| 10 \| \| MPEG1 \| 0 \| 1.94102385 \| 0 \| 10 \| \| CD83 \| 0 \| 1.914405018 \| 0 \| 10 \| \| HLA-DQA1 \| 0 \| 1.909497115 \| 0 \| 10 \| \| GLUL \| 0 \| 1.896922985 \| 0 \| 10 \| \| HLA-DQB1 \| 0 \| 1.878455397 \| 0 \| 10 \| \| TNFAIP2 \| 0 \| 1.870766764 \| 0 \| 10 \| \| MS4A6A \| 0 \| 1.856443091 \| 0 \| 10 \| \| RAB31 \| 0 \| 1.844188003 \| 0 \| 10 \| \| SPI1 \| 0 \| 1.822669897 \| 0 \| 10 \| \| SERPINB9 \| 0 \| 1.820633733 \| 0 \| 10 \| \| JAML \| 0 \| 1.812752291 \| 0 \| 10 \| \| LCP1 \| 0 \| 1.806234393 \| 0 \| 10 \| \| ICAM1 \| 0 \| 1.800072484 \| 0 \| 10 \| \| FTL \| 0 \| 1.77948987 \| 0 \| 10 \| \| PTPRE \| 0 \| 1.779168606 \| 0 \| 10 \| \| C1orf162 \| 0 \| 1.759286074 \| 0 \| 10 \| \| NR4A3 \| 0 \| 1.754655477 \| 0 \| 10 \| \| CLEC4E \| 0 \| 1.75168046 \| 0 \| 10 \| \| GK \| 0 \| 1.750324895 \| 0 \| 10 \| \| THBD \| 0 \| 1.741164312 \| 0 \| 10 \| \| CEBPB \| 0 \| 1.729055186 \| 0 \| 10 \| \| CLEC7A \| 0 \| 1.711412547 \| 0 \| 10 \| \| HBEGF \| 0 \| 1.697252397 \| 0 \| 10 \| \| ITGB2 \| 0 \| 1.684549145 \| 0 \| 10 \| \| CSTA \| 0 \| 1.679930453 \| 0 \| 10 \| \| LYN \| 0 \| 1.67124718 \| 0 \| 10 \| \| PSAP \| 0 \| 1.670668984 \| 0 \| 10 \| \| FAM49A \| 0 \| 1.654825762 \| 0 \| 10 \| \| SAMHD1 \| 0 \| 1.641288796 \| 0 \| 10 \| \| SAMSN1 \| 0 \| 1.639086046 \| 0 \| 10 \| \| IGSF6 \| 0 \| 1.635155006 \| 0 \| 10 \| \| ACTB \| 0 \| 1.630265229 \| 0 \| 10 \| \| PNRC1 \| 0 \| 1.623839138 \| 0 \| 10 \| \| RILPL2 \| 0 \| 1.620799805 \| 0 \| 10 \| \| VIM \| 0 \| 1.615699959 \| 0 \| 10 \| \| LAPTM5 \| 0 \| 1.607007685 \| 0 \| 10 \| \| FPR2 \| 0 \| 1.601761925 \| 0 \| 10 \| \| LCP2 \| 0 \| 1.582703295 \| 0 \| 10 \| \| CEBPD \| 0 \| 1.566049712 \| 0 \| 10 \| \| HIF1A \| 0 \| 1.559995199 \| 0 \| 10 \| \| OSM \| 0 \| 1.551023213 \| 0 \| 10 \| \| LILRB2 \| 0 \| 1.546023181 \| 0 \| 10 \| \| HLA-DMB \| 0 \| 1.544135988 \| 0 \| 10 \| \| TNFRSF1B \| 0 \| 1.541949188 \| 0 \| 10 \| \| ANXA5 \| 0 \| 1.537743591 \| 0 \| 10 \| \| IRAK3 \| 0 \| 1.53065265 \| 0 \| 10 \| \| PHACTR1 \| 0 \| 1.525956224 \| 0 \| 10 \| \| STX11 \| 0 \| 1.524142119 \| 0 \| 10 \| \| HLA-DQA2 \| 0 \| 1.520244645 \| 0 \| 10 \| \| CLEC10A \| 0 \| 1.509032664 \| 0 \| 10 \| \| NFKB1 \| 0 \| 1.508944693 \| 0 \| 10 \| \| KCTD12 \| 0 \| 1.487825596 \| 0 \| 10 \| \| AP1S2 \| 0 \| 1.486039975 \| 0 \| 10 \| \| FCN1 \| 0 \| 1.483637608 \| 0 \| 10 \| \| RNF144B \| 0 \| 1.480901499 \| 0 \| 10 \| \| SERPINA1 \| 0 \| 1.468913881 \| 0 \| 10 \| \| S100A11 \| 0 \| 1.459680529 \| 0 \| 10 \| \| SNX10 \| 0 \| 1.44983785 \| 0 \| 10 \| \| ARL5B \| 0 \| 1.448616233 \| 0 \| 10 \| \| CFP \| 0 \| 1.448452369 \| 0 \| 10 \| \| CD93 \| 0 \| 1.447406854 \| 0 \| 10 \| \| CD86 \| 0 \| 1.445324841 \| 0 \| 10 \| \| FGR \| 0 \| 1.444382497 \| 0 \| 10 \| \| LGALS2 \| 0 \| 1.442755447 \| 0 \| 10 \| \| CTSH \| 0 \| 1.436643239 \| 0 \| 10 \| \| KYNU \| 0 \| 1.427101806 \| 0 \| 10 \| \| ATP2B1-AS1 \| 0 \| 1.411265922 \| 0 \| 10 \| \| MIR3945HG \| 0 \| 1.407744609 \| 0 \| 10 \| \| TNFSF13B \| 0 \| 1.406225717 \| 0 \| 10 \| \| PPT1 \| 0 \| 1.404044024 \| 0 \| 10 \| \| ITGAX \| 0 \| 1.399904024 \| 0 \| 10 \| \| KDM6B \| 0 \| 1.397471296 \| 0 \| 10 \| \| HCK \| 0 \| 1.384592537 \| 0 \| 10 \| \| SOCS3 \| 0 \| 1.376579114 \| 0 \| 10 \| \| PDE4B \| 0 \| 1.376123694 \| 0 \| 10 \| \| SLC8A1 \| 0 \| 1.36787955 \| 0 \| 10 \| \| LSP1 \| 0 \| 1.361292559 \| 0 \| 10 \| \| CD68 \| 0 \| 1.360514607 \| 0 \| 10 \| \| RNASE6 \| 0 \| 1.351009686 \| 0 \| 10 \| \| DUSP1 \| 0 \| 1.338819551 \| 0 \| 10 \| \| GNAI2 \| 0 \| 1.335599005 \| 0 \| 10 \| \| VSIR \| 0 \| 1.33319338 \| 0 \| 10 \| \| SDCBP \| 0 \| 1.318086064 \| 0 \| 10 \| \| FCGR3A \| 0 \| 1.310533883 \| 0 \| 10 \| \| SMIM25 \| 0 \| 1.308779274 \| 0 \| 10 \| \| PLAUR \| 0 \| 1.306441935 \| 0 \| 10 \| \| ADGRE2 \| 0 \| 1.301564367 \| 0 \| 10 \| \| MRC1 \| 0 \| 1.29366976 \| 0 \| 10 \| \| NFIL3 \| 0 \| 1.287274419 \| 0 \| 10 \| \| SOD2 \| 0 \| 1.285236327 \| 0 \| 10 \| \| FAM49B \| 0 \| 1.280795116 \| 0 \| 10 \| \| PLXDC2 \| 0 \| 1.28013133 \| 0 \| 10 \| \| NINJ1 \| 0 \| 1.279996041 \| 0 \| 10 \| \| CSF1R \| 0 \| 1.277041301 \| 0 \| 10 \| \| HLA-DMA \| 0 \| 1.274251469 \| 0 \| 10 \| \| OGFRL1 \| 0 \| 1.273294819 \| 0 \| 10 \| \| CMTM6 \| 0 \| 1.272332969 \| 0 \| 10 \| \| ETS2 \| 0 \| 1.270486034 \| 0 \| 10 \| \| ARRB2 \| 0 \| 1.262686233 \| 0 \| 10 \| \| RIPK2 \| 0 \| 1.261698644 \| 0 \| 10 \| \| CCDC88A \| 0 \| 1.245151026 \| 0 \| 10 \| \| MCL1 \| 0 \| 1.241422731 \| 0 \| 10 \| \| AC245128.3 \| 0 \| 1.237622882 \| 0 \| 10 \| \| LGALS1 \| 0 \| 1.237457829 \| 0 \| 10 \| \| SLC43A2 \| 0 \| 1.236854758 \| 0 \| 10 \| \| BID \| 0 \| 1.236484463 \| 0 \| 10 \| \| CTSZ \| 0 \| 1.23204503 \| 0 \| 10 \| \| GRINA \| 0 \| 1.23073772 \| 0 \| 10 \| \| LILRB3 \| 0 \| 1.22634144 \| 0 \| 10 \| \| TET2 \| 0 \| 1.221330367 \| 0 \| 10 \| \| REL \| 0 \| 1.217351339 \| 0 \| 10 \| \| HCLS1 \| 0 \| 1.21675907 \| 0 \| 10 \| \| SH3BP5 \| 0 \| 1.215371711 \| 0 \| 10 \| \| IFNGR1 \| 0 \| 1.21069641 \| 0 \| 10 \| \| LILRA5 \| 0 \| 1.207158858 \| 0 \| 10 \| \| GRASP \| 0 \| 1.202974367 \| 0 \| 10 \| \| MAFB \| 0 \| 1.19183072 \| 0 \| 10 \| \| SLC11A1 \| 0 \| 1.179257317 \| 0 \| 10 \| \| RNF130 \| 0 \| 1.174447959 \| 0 \| 10 \| \| EMILIN2 \| 0 \| 1.173164848 \| 0 \| 10 \| \| PIK3AP1 \| 0 \| 1.16552909 \| 0 \| 10 \| \| CFD \| 0 \| 1.152550398 \| 0 \| 10 \| \| TPP1 \| 0 \| 1.133874261 \| 0 \| 10 \| \| THEMIS2 \| 0 \| 1.126753192 \| 0 \| 10 \| \| LRRK2 \| 0 \| 1.120920199 \| 0 \| 10 \| \| TLR2 \| 0 \| 1.115405453 \| 0 \| 10 \| \| SH2B3 \| 0 \| 1.112029307 \| 0 \| 10 \| \| CXCL16 \| 0 \| 1.108845457 \| 0 \| 10 \| \| C3AR1 \| 0 \| 1.107005795 \| 0 \| 10 \| \| IRF8 \| 0 \| 1.102864725 \| 0 \| 10 \| \| PLXNC1 \| 0 \| 1.100630791 \| 0 \| 10 \| \| ADA2 \| 0 \| 1.080978658 \| 0 \| 10 \| \| CCRL2 \| 0 \| 1.076205456 \| 0 \| 10 \| \| AOAH \| 0 \| 1.070204541 \| 0 \| 10 \| \| METRNL \| 0 \| 1.068843807 \| 0 \| 10 \| \| ATG3 \| 0 \| 1.066977679 \| 0 \| 10 \| \| GRN \| 0 \| 1.061411786 \| 0 \| 10 \| \| MAP3K8 \| 0 \| 1.051496566 \| 0 \| 10 \| \| RASSF2 \| 0 \| 1.048287138 \| 0 \| 10 \| \| CSF2RB \| 0 \| 1.047113891 \| 0 \| 10 \| \| SIRPA \| 0 \| 1.045011403 \| 0 \| 10 \| \| CD53 \| 0 \| 1.043221517 \| 0 \| 10 \| \| MXD1 \| 0 \| 1.040480359 \| 0 \| 10 \| \| LILRB1 \| 0 \| 1.039262455 \| 0 \| 10 \| \| 1-Mar \| 0 \| 1.03173782 \| 0 \| 10 \| \| RASSF4 \| 0 \| 1.028929643 \| 0 \| 10 \| \| SLC7A7 \| 0 \| 1.026686152 \| 0 \| 10 \| \| CD36 \| 0 \| 1.025810828 \| 0 \| 10 \| \| C9orf72 \| 0 \| 1.017568526 \| 0 \| 10 \| \| WARS \| 2.3943E-304 \| 1.333687732 \| 5.2261E-300 \| 10 \| \| ARPC3 \| 3.805E-304 \| 1.043610882 \| 8.3052E-300 \| 10 \| \| LIMS1 \| 6.9186E-303 \| 1.351596698 \| 1.5101E-298 \| 10 \| \| SERPINB1 \| 4.8847E-291 \| 1.275462565 \| 1.0662E-286 \| 10 \| \| ACTR2 \| 1.4853E-287 \| 1.132730262 \| 3.2419E-283 \| 10 \| \| NFKBIA \| 1.6314E-282 \| 1.679475528 \| 3.561E-278 \| 10 \| \| TGFBI \| 1.7909E-282 \| 1.04481923 \| 3.909E-278 \| 10 \| \| OAZ1 \| 1.6846E-267 \| 1.023326893 \| 3.6771E-263 \| 10 \| \| GPCPD1 \| 3.6112E-266 \| 1.118724323 \| 7.8822E-262 \| 10 \| \| UPP1 \| 9.0447E-265 \| 1.064168318 \| 1.9742E-260 \| 10 \| \| ATP1B3 \| 1.2622E-261 \| 1.49838528 \| 2.755E-257 \| 10 \| \| ATP6V0B \| 2.9268E-260 \| 1.030572834 \| 6.3883E-256 \| 10 \| \| S100A4 \| 4.2051E-258 \| 1.206700882 \| 9.1785E-254 \| 10 \| \| TNFAIP3 \| 9.4046E-258 \| 1.23326726 \| 2.0527E-253 \| 10 \| \| FOSL2 \| 2.6987E-252 \| 1.073632612 \| 5.8905E-248 \| 10 \| \| ATP13A3 \| 1.7735E-251 \| 1.137301453 \| 3.8711E-247 \| 10 \| \| NR4A2 \| 6.844E-250 \| 1.088297394 \| 1.4938E-245 \| 10 \| \| SGK1 \| 9.8032E-246 \| 1.155442942 \| 2.1398E-241 \| 10 \| \| PYCARD \| 1.2727E-245 \| 1.019389296 \| 2.7779E-241 \| 10 \| \| MGAT1 \| 6.5391E-242 \| 1.101516862 \| 1.4273E-237 \| 10 \| \| THBS1 \| 1.4644E-239 \| 2.74105587 \| 3.1963E-235 \| 10 \| \| CFLAR \| 8.9972E-236 \| 1.111668405 \| 1.9638E-231 \| 10 \| \| CXCL3 \| 7.8716E-234 \| 2.477499102 \| 1.7181E-229 \| 10 \| \| IER3 \| 2.9574E-230 \| 1.586962296 \| 6.4552E-226 \| 10 \| \| LAP3 \| 6.1597E-229 \| 1.090734602 \| 1.3445E-224 \| 10 \| \| ZFP36 \| 1.445E-220 \| 1.044645912 \| 3.154E-216 \| 10 \| \| ATP2B1 \| 2.486E-220 \| 1.192995908 \| 5.4262E-216 \| 10 \| \| NABP1 \| 9.6149E-219 \| 1.13135784 \| 2.0986E-214 \| 10 \| \| APLP2 \| 6.6314E-212 \| 1.035970268 \| 1.4474E-207 \| 10 \| \| CD44 \| 8.6022E-202 \| 1.255577408 \| 1.8776E-197 \| 10 \| \| YBX3 \| 1.1625E-201 \| 1.137184585 \| 2.5373E-197 \| 10 \| \| JUN \| 1.5344E-200 \| -1.351428373 \| 3.3492E-196 \| 10 \| \| CCL4L2 \| 5.119E-197 \| 1.423197044 \| 1.1173E-192 \| 10 \| \| GBP1 \| 9.3381E-193 \| 1.416305898 \| 2.0382E-188 \| 10 \| \| NFKBIZ \| 1.1813E-192 \| 1.228100573 \| 2.5785E-188 \| 10 \| \| SLC25A37 \| 1.7223E-192 \| 1.356038109 \| 3.7593E-188 \| 10 \| \| SYNE2 \| 4.6734E-183 \| -1.439087966 \| 1.0201E-178 \| 10 \| \| PLIN2 \| 1.82E-180 \| 1.266272464 \| 3.9724E-176 \| 10 \| \| CDKN1A \| 1.6496E-175 \| 1.012341992 \| 3.6006E-171 \| 10 \| \| ALOX5AP \| 1.402E-165 \| 1.26304886 \| 3.0602E-161 \| 10 \| \| ITM2C \| 1.2124E-159 \| -1.631254207 \| 2.6464E-155 \| 10 \| \| PPDPF \| 1.0695E-147 \| -1.409872021 \| 2.3344E-143 \| 10 \| \| IFI27 \| 1.4345E-139 \| -2.762394263 \| 3.131E-135 \| 10 \| \| SPTBN1 \| 1.8586E-134 \| -1.419774792 \| 4.0568E-130 \| 10 \| \| KRT18 \| 2.0138E-133 \| -2.569736088 \| 4.3955E-129 \| 10 \| \| ELF3 \| 4.1359E-133 \| -2.239620317 \| 9.0273E-129 \| 10 \| \| IVNS1ABP \| 1.9165E-132 \| 1.256354128 \| 4.1832E-128 \| 10 \| \| EHD1 \| 3.7596E-129 \| 1.017359754 \| 8.206E-125 \| 10 \| \| KRT8 \| 1.0795E-128 \| -2.727146369 \| 2.3561E-124 \| 10 \| \| TMEM238 \| 1.8154E-127 \| -1.245607441 \| 3.9625E-123 \| 10 \| \| LGALS4 \| 3.7862E-127 \| -2.841335467 \| 8.264E-123 \| 10 \| \| EPCAM \| 2.7893E-126 \| -2.052300256 \| 6.0883E-122 \| 10 \| \| C12orf57 \| 4.5416E-126 \| -1.103504988 \| 9.913E-122 \| 10 \| \| ITGA6 \| 2.6364E-125 \| -1.157257317 \| 5.7546E-121 \| 10 \| \| CLDN4 \| 1.7158E-124 \| -2.101710416 \| 3.7452E-120 \| 10 \| \| VEGFA \| 2.1586E-123 \| 1.010936955 \| 4.7115E-119 \| 10 \| \| CLDN3 \| 4.6684E-122 \| -2.05620426 \| 1.019E-117 \| 10 \| \| PHGR1 \| 6.8281E-122 \| -4.325108732 \| 1.4904E-117 \| 10 \| \| FKBP11 \| 2.9276E-120 \| -1.2306093 \| 6.39E-116 \| 10 \| \| CAMK2N1 \| 1.2139E-119 \| -1.3965853 \| 2.6496E-115 \| 10 \| \| CDC42EP5 \| 9.3305E-118 \| -1.479459453 \| 2.0366E-113 \| 10 \| \| KRT19 \| 8.87E-114 \| -2.695483208 \| 1.9361E-109 \| 10 \| \| LGALS3BP \| 1.2743E-113 \| -1.389993237 \| 2.7814E-109 \| 10 \| \| SMIM22 \| 3.0864E-112 \| -1.6488866 \| 6.7368E-108 \| 10 \| \| TSPAN8 \| 2.1546E-107 \| -2.221373853 \| 4.7029E-103 \| 10 \| \| DSP \| 4.8678E-106 \| -1.389117217 \| 1.0625E-101 \| 10 \| \| PERP \| 7.4462E-106 \| -1.313834167 \| 1.6253E-101 \| 10 \| \| TENT5C \| 5.1255E-104 \| -1.114107485 \| 1.1187E-99 \| 10 \| \| SLC44A4 \| 1.3029E-101 \| -1.322197029 \| 2.84388E-97 \| 10 \| \| FXYD3 \| 9.6172E-101 \| -2.526223421 \| 2.09914E-96 \| 10 \| \| TSPAN1 \| 6.8513E-99 \| -2.198274024 \| 1.49542E-94 \| 10 \| \| TPM1 \| 6.3251E-98 \| -1.335118421 \| 1.38058E-93 \| 10 \| \| TMEM54 \| 3.13742E-96 \| -1.57686064 \| 6.84805E-92 \| 10 \| \| FAM3D \| 1.9328E-93 \| -1.681719231 \| 4.21873E-89 \| 10 \| \| S100A14 \| 8.53261E-91 \| -1.258352726 \| 1.86241E-86 \| 10 \| \| PDLIM1 \| 2.74392E-90 \| -1.042205314 \| 5.98916E-86 \| 10 \| \| AGR2 \| 3.76215E-89 \| -2.360011647 \| 8.21164E-85 \| 10 \| \| CD24 \| 8.63294E-89 \| -1.563548216 \| 1.88431E-84 \| 10 \| \| MUC13 \| 1.17359E-88 \| -1.494169601 \| 2.5616E-84 \| 10 \| \| CD3D \| 5.75429E-83 \| -1.801228101 \| 1.25599E-78 \| 10 \| \| SELENOP \| 2.88748E-82 \| -2.186266597 \| 6.3025E-78 \| 10 \| \| PIGR \| 2.18797E-80 \| -3.480683114 \| 4.77569E-76 \| 10 \| \| KLF5 \| 1.01654E-79 \| -1.161410238 \| 2.21881E-75 \| 10 \| \| TFF3 \| 5.96406E-79 \| -3.405637072 \| 1.30178E-74 \| 10 \| \| GPX2 \| 3.03995E-78 \| -1.385865199 \| 6.63531E-74 \| 10 \| \| RRBP1 \| 4.15096E-75 \| -1.000503605 \| 9.06029E-71 \| 10 \| \| TRAC \| 4.9732E-75 \| -1.603775207 \| 1.0855E-70 \| 10 \| \| LIMA1 \| 8.36613E-72 \| -1.001547655 \| 1.82608E-67 \| 10 \| \| SSR4 \| 1.6191E-69 \| -1.394546413 \| 3.53401E-65 \| 10 \| \| LCN2 \| 4.07238E-67 \| -1.999573323 \| 8.88878E-63 \| 10 \| \| ID1 \| 8.3373E-65 \| -1.17558091 \| 1.81978E-60 \| 10 \| \| ADM \| 1.23183E-62 \| 1.169214729 \| 2.68872E-58 \| 10 \| \| CKB \| 1.68818E-60 \| -1.935997087 \| 3.6848E-56 \| 10 \| \| ARID5B \| 1.01468E-56 \| -1.000407657 \| 2.21475E-52 \| 10 \| \| CD2 \| 6.22318E-56 \| -1.579918436 \| 1.35833E-51 \| 10 \| \| SLC12A2 \| 8.39114E-55 \| -1.097832143 \| 1.83153E-50 \| 10 \| \| CD9 \| 7.12307E-49 \| -1.040367158 \| 1.55475E-44 \| 10 \| \| IGKC \| 4.48834E-46 \| -4.754031693 \| 9.7967E-42 \| 10 \| \| FABP1 \| 3.50295E-41 \| -3.347790663 \| 7.64589E-37 \| 10 \| \| CCL20 \| 8.47793E-41 \| 1.638268753 \| 1.85048E-36 \| 10 \| \| JCHAIN \| 1.03422E-31 \| -4.277960639 \| 2.2574E-27 \| 10 \| \| IGHA1 \| 1.24116E-26 \| -3.926891442 \| 2.70907E-22 \| 10 \| \| LGALS3 \| 2.56933E-13 \| -1.075505554 \| 5.60808E-09 \| 10 \| \| CD69 \| 6.11872E-12 \| -1.016064537 \| 1.33553E-07 \| 10 \| \| IGLC2 \| 3.53743E-10 \| -5.657037978 \| 7.72114E-06 \| 10 \| \| SPP1 \| 0 \| 5.604748285 \| 0 \| 11 \| \| APOE \| 0 \| 5.419198855 \| 0 \| 11 \| \| C1QB \| 0 \| 5.374334967 \| 0 \| 11 \| \| C1QA \| 0 \| 5.286332753 \| 0 \| 11 \| \| C1QC \| 0 \| 4.517783718 \| 0 \| 11 \| \| MMP12 \| 0 \| 4.077550197 \| 0 \| 11 \| \| HLA-DRA \| 0 \| 3.882769245 \| 0 \| 11 \| \| HLA-DRB1 \| 0 \| 3.730068884 \| 0 \| 11 \| \| GPNMB \| 0 \| 3.703539627 \| 0 \| 11 \| \| HLA-DPA1 \| 0 \| 3.696645528 \| 0 \| 11 \| \| APOC1 \| 0 \| 3.693961453 \| 0 \| 11 \| \| FTL \| 0 \| 3.653695589 \| 0 \| 11 \| \| CD14 \| 0 \| 3.55286842 \| 0 \| 11 \| \| PSAP \| 0 \| 3.478516801 \| 0 \| 11 \| \| CD74 \| 0 \| 3.442663421 \| 0 \| 11 \| \| CCL18 \| 0 \| 3.434235404 \| 0 \| 11 \| \| TYROBP \| 0 \| 3.374978004 \| 0 \| 11 \| \| CTSL \| 0 \| 3.36566936 \| 0 \| 11 \| \| CD68 \| 0 \| 3.358600729 \| 0 \| 11 \| \| CCL3 \| 0 \| 3.357130215 \| 0 \| 11 \| \| HLA-DPB1 \| 0 \| 3.348644155 \| 0 \| 11 \| \| LYZ \| 0 \| 3.288452632 \| 0 \| 11 \| \| AIF1 \| 0 \| 3.259394412 \| 0 \| 11 \| \| HLA-DRB5 \| 0 \| 3.170366922 \| 0 \| 11 \| \| LGMN \| 0 \| 3.084898194 \| 0 \| 11 \| \| FCGR3A \| 0 \| 3.044966313 \| 0 \| 11 \| \| MMP9 \| 0 \| 3.043623198 \| 0 \| 11 \| \| RNASE1 \| 0 \| 3.037056101 \| 0 \| 11 \| \| MRC1 \| 0 \| 2.982242197 \| 0 \| 11 \| \| CTSZ \| 0 \| 2.980791899 \| 0 \| 11 \| \| CXCL8 \| 0 \| 2.919452793 \| 0 \| 11 \| \| MS4A6A \| 0 \| 2.917429394 \| 0 \| 11 \| \| ACP5 \| 0 \| 2.851206835 \| 0 \| 11 \| \| FGL2 \| 0 \| 2.814332404 \| 0 \| 11 \| \| CYBB \| 0 \| 2.765316333 \| 0 \| 11 \| \| GLUL \| 0 \| 2.693867803 \| 0 \| 11 \| \| SLC40A1 \| 0 \| 2.691723499 \| 0 \| 11 \| \| CXCL2 \| 0 \| 2.655514599 \| 0 \| 11 \| \| STAB1 \| 0 \| 2.631050606 \| 0 \| 11 \| \| MS4A4A \| 0 \| 2.624938531 \| 0 \| 11 \| \| HLA-DQB1 \| 0 \| 2.605219093 \| 0 \| 11 \| \| HLA-DMB \| 0 \| 2.601856482 \| 0 \| 11 \| \| ASAH1 \| 0 \| 2.523060989 \| 0 \| 11 \| \| GRN \| 0 \| 2.501922874 \| 0 \| 11 \| \| PLA2G7 \| 0 \| 2.479869052 \| 0 \| 11 \| \| CPVL \| 0 \| 2.473005168 \| 0 \| 11 \| \| LIPA \| 0 \| 2.469944478 \| 0 \| 11 \| \| C5AR1 \| 0 \| 2.461929278 \| 0 \| 11 \| \| MAFB \| 0 \| 2.441788335 \| 0 \| 11 \| \| MPEG1 \| 0 \| 2.438285436 \| 0 \| 11 \| \| CXCL3 \| 0 \| 2.410848416 \| 0 \| 11 \| \| SLCO2B1 \| 0 \| 2.410360852 \| 0 \| 11 \| \| CSF1R \| 0 \| 2.397192092 \| 0 \| 11 \| \| CCL3L1 \| 0 \| 2.390651869 \| 0 \| 11 \| \| CTSC \| 0 \| 2.36980801 \| 0 \| 11 \| \| HLA-DQA1 \| 0 \| 2.351808592 \| 0 \| 11 \| \| CAPG \| 0 \| 2.343954006 \| 0 \| 11 \| \| CD163 \| 0 \| 2.338275157 \| 0 \| 11 \| \| IL1B \| 0 \| 2.295568698 \| 0 \| 11 \| \| KCTD12 \| 0 \| 2.28101301 \| 0 \| 11 \| \| FPR3 \| 0 \| 2.280376793 \| 0 \| 11 \| \| LAPTM5 \| 0 \| 2.250071229 \| 0 \| 11 \| \| ITGB2 \| 0 \| 2.246401079 \| 0 \| 11 \| \| CCL4L2 \| 0 \| 2.239438185 \| 0 \| 11 \| \| TYMP \| 0 \| 2.23775699 \| 0 \| 11 \| \| SAT1 \| 0 \| 2.200091534 \| 0 \| 11 \| \| CTSH \| 0 \| 2.195096974 \| 0 \| 11 \| \| SGK1 \| 0 \| 2.191737783 \| 0 \| 11 \| \| SLC16A10 \| 0 \| 2.170713424 \| 0 \| 11 \| \| HMOX1 \| 0 \| 2.161457558 \| 0 \| 11 \| \| IGSF6 \| 0 \| 2.136832984 \| 0 \| 11 \| \| FCGRT \| 0 \| 2.094813332 \| 0 \| 11 \| \| RNASE6 \| 0 \| 2.06338703 \| 0 \| 11 \| \| VSIG4 \| 0 \| 2.058103587 \| 0 \| 11 \| \| CXCL16 \| 0 \| 2.040647528 \| 0 \| 11 \| \| CTSS \| 0 \| 2.002151434 \| 0 \| 11 \| \| LITAF \| 0 \| 1.999016062 \| 0 \| 11 \| \| FTH1 \| 0 \| 1.997348318 \| 0 \| 11 \| \| HLA-DMA \| 0 \| 1.988208516 \| 0 \| 11 \| \| TMEM176B \| 0 \| 1.985000882 \| 0 \| 11 \| \| PPT1 \| 0 \| 1.98021771 \| 0 \| 11 \| \| TGFBI \| 0 \| 1.975782481 \| 0 \| 11 \| \| MS4A7 \| 0 \| 1.967985465 \| 0 \| 11 \| \| HLA-DQA2 \| 0 \| 1.948479275 \| 0 \| 11 \| \| LGALS1 \| 0 \| 1.940825236 \| 0 \| 11 \| \| RAB31 \| 0 \| 1.930553448 \| 0 \| 11 \| \| SDS \| 0 \| 1.922979808 \| 0 \| 11 \| \| C3AR1 \| 0 \| 1.919636157 \| 0 \| 11 \| \| MFSD1 \| 0 \| 1.913293249 \| 0 \| 11 \| \| ABCA1 \| 0 \| 1.880944078 \| 0 \| 11 \| \| RNF130 \| 0 \| 1.865798192 \| 0 \| 11 \| \| PLEK \| 0 \| 1.86221576 \| 0 \| 11 \| \| TIMP2 \| 0 \| 1.855927798 \| 0 \| 11 \| \| SPI1 \| 0 \| 1.842586223 \| 0 \| 11 \| \| ANXA5 \| 0 \| 1.824847492 \| 0 \| 11 \| \| PLTP \| 0 \| 1.818514518 \| 0 \| 11 \| \| CD81 \| 0 \| 1.808309176 \| 0 \| 11 \| \| AC020656.1 \| 0 \| 1.807682556 \| 0 \| 11 \| \| TPP1 \| 0 \| 1.785051451 \| 0 \| 11 \| \| G0S2 \| 0 \| 1.775975849 \| 0 \| 11 \| \| GM2A \| 0 \| 1.773193298 \| 0 \| 11 \| \| PMP22 \| 0 \| 1.758710066 \| 0 \| 11 \| \| ADAP2 \| 0 \| 1.741697361 \| 0 \| 11 \| \| CD83 \| 0 \| 1.740456978 \| 0 \| 11 \| \| FBP1 \| 0 \| 1.739049766 \| 0 \| 11 \| \| PLXDC2 \| 0 \| 1.736492918 \| 0 \| 11 \| \| FUCA1 \| 0 \| 1.728698806 \| 0 \| 11 \| \| AKR1B1 \| 0 \| 1.715282269 \| 0 \| 11 \| \| RASSF4 \| 0 \| 1.711326612 \| 0 \| 11 \| \| GPR183 \| 0 \| 1.695372756 \| 0 \| 11 \| \| FOLR2 \| 0 \| 1.688876508 \| 0 \| 11 \| \| ATP6AP2 \| 0 \| 1.676951681 \| 0 \| 11 \| \| ATP6V1F \| 0 \| 1.676826527 \| 0 \| 11 \| \| CEBPB \| 0 \| 1.675296665 \| 0 \| 11 \| \| MNDA \| 0 \| 1.673381469 \| 0 \| 11 \| \| SAMHD1 \| 0 \| 1.671601986 \| 0 \| 11 \| \| CLEC7A \| 0 \| 1.664040834 \| 0 \| 11 \| \| PTGS2 \| 0 \| 1.661615316 \| 0 \| 11 \| \| CD4 \| 0 \| 1.650538031 \| 0 \| 11 \| \| ITM2B \| 0 \| 1.650493199 \| 0 \| 11 \| \| LST1 \| 0 \| 1.647769066 \| 0 \| 11 \| \| NRP1 \| 0 \| 1.64753235 \| 0 \| 11 \| \| ICAM1 \| 0 \| 1.644440145 \| 0 \| 11 \| \| PLAU \| 0 \| 1.633361225 \| 0 \| 11 \| \| F13A1 \| 0 \| 1.626778142 \| 0 \| 11 \| \| CTSB \| 0 \| 1.626103939 \| 0 \| 11 \| \| CD86 \| 0 \| 1.624972578 \| 0 \| 11 \| \| ATP6V0B \| 0 \| 1.607717623 \| 0 \| 11 \| \| ADA2 \| 0 \| 1.60215384 \| 0 \| 11 \| \| S100A11 \| 0 \| 1.592050232 \| 0 \| 11 \| \| MGAT1 \| 0 \| 1.59069318 \| 0 \| 11 \| \| HIF1A \| 0 \| 1.590010369 \| 0 \| 11 \| \| TREM2 \| 0 \| 1.585006812 \| 0 \| 11 \| \| VIM \| 0 \| 1.579471556 \| 0 \| 11 \| \| SLC7A7 \| 0 \| 1.577517 \| 0 \| 11 \| \| TTYH3 \| 0 \| 1.576740946 \| 0 \| 11 \| \| LILRB4 \| 0 \| 1.574666201 \| 0 \| 11 \| \| GNS \| 0 \| 1.56786139 \| 0 \| 11 \| \| SLC15A3 \| 0 \| 1.553673348 \| 0 \| 11 \| \| SDCBP \| 0 \| 1.553186014 \| 0 \| 11 \| \| ABL2 \| 0 \| 1.551390035 \| 0 \| 11 \| \| MSR1 \| 0 \| 1.547271046 \| 0 \| 11 \| \| CCL4 \| 0 \| 1.537146649 \| 0 \| 11 \| \| SCPEP1 \| 0 \| 1.531471518 \| 0 \| 11 \| \| GNB4 \| 0 \| 1.530266062 \| 0 \| 11 \| \| MARCKS \| 0 \| 1.530257028 \| 0 \| 11 \| \| SIRPA \| 0 \| 1.504780933 \| 0 \| 11 \| \| CD84 \| 0 \| 1.502627869 \| 0 \| 11 \| \| TNFAIP2 \| 0 \| 1.501066816 \| 0 \| 11 \| \| LAMP1 \| 0 \| 1.500094377 \| 0 \| 11 \| \| GPR34 \| 0 \| 1.496305105 \| 0 \| 11 \| \| LAIR1 \| 0 \| 1.495282661 \| 0 \| 11 \| \| GRINA \| 0 \| 1.491467524 \| 0 \| 11 \| \| ARL4C \| 0 \| 1.483819424 \| 0 \| 11 \| \| SLC8A1 \| 0 \| 1.482225075 \| 0 \| 11 \| \| A2M \| 0 \| 1.472161416 \| 0 \| 11 \| \| CEBPD \| 0 \| 1.457723315 \| 0 \| 11 \| \| NCF2 \| 0 \| 1.457047479 \| 0 \| 11 \| \| EMILIN2 \| 0 \| 1.451247263 \| 0 \| 11 \| \| PLXNC1 \| 0 \| 1.45086055 \| 0 \| 11 \| \| DAB2 \| 0 \| 1.448106324 \| 0 \| 11 \| \| LYN \| 0 \| 1.44638858 \| 0 \| 11 \| \| BASP1 \| 0 \| 1.434683491 \| 0 \| 11 \| \| TNFSF13B \| 0 \| 1.433334075 \| 0 \| 11 \| \| CD63 \| 0 \| 1.430400855 \| 0 \| 11 \| \| C1orf162 \| 0 \| 1.430389866 \| 0 \| 11 \| \| OLR1 \| 0 \| 1.429303618 \| 0 \| 11 \| \| TMEM176A \| 0 \| 1.415290232 \| 0 \| 11 \| \| YWHAH \| 0 \| 1.40581595 \| 0 \| 11 \| \| COTL1 \| 0 \| 1.404429171 \| 0 \| 11 \| \| ATP6AP1 \| 0 \| 1.399013295 \| 0 \| 11 \| \| PRDX1 \| 0 \| 1.398213401 \| 0 \| 11 \| \| GNAI2 \| 0 \| 1.393289822 \| 0 \| 11 \| \| FCGR2B \| 0 \| 1.390901383 \| 0 \| 11 \| \| AXL \| 0 \| 1.390889815 \| 0 \| 11 \| \| ATOX1 \| 0 \| 1.386630336 \| 0 \| 11 \| \| MMP19 \| 0 \| 1.381943783 \| 0 \| 11 \| \| ARHGAP18 \| 0 \| 1.379579638 \| 0 \| 11 \| \| CD93 \| 0 \| 1.377975423 \| 0 \| 11 \| \| LIMS1 \| 0 \| 1.371880967 \| 0 \| 11 \| \| HLA-DOA \| 0 \| 1.369950994 \| 0 \| 11 \| \| HEXB \| 0 \| 1.369333072 \| 0 \| 11 \| \| AP2S1 \| 0 \| 1.369025036 \| 0 \| 11 \| \| VMO1 \| 0 \| 1.367502402 \| 0 \| 11 \| \| GSTO1 \| 0 \| 1.36280905 \| 0 \| 11 \| \| DMXL2 \| 0 \| 1.360601382 \| 0 \| 11 \| \| OGFRL1 \| 0 \| 1.359346417 \| 0 \| 11 \| \| ENPP2 \| 0 \| 1.353987986 \| 0 \| 11 \| \| ALOX5AP \| 0 \| 1.342422111 \| 0 \| 11 \| \| VAMP8 \| 0 \| 1.33223114 \| 0 \| 11 \| \| ACTB \| 0 \| 1.331642708 \| 0 \| 11 \| \| IL1RN \| 0 \| 1.330389263 \| 0 \| 11 \| \| IFNGR1 \| 0 \| 1.33024866 \| 0 \| 11 \| \| LILRB2 \| 0 \| 1.328992996 \| 0 \| 11 \| \| GAA \| 0 \| 1.324309981 \| 0 \| 11 \| \| CD300A \| 0 \| 1.322496602 \| 0 \| 11 \| \| C1orf54 \| 0 \| 1.322367061 \| 0 \| 11 \| \| RGS10 \| 0 \| 1.321000345 \| 0 \| 11 \| \| CCDC88A \| 0 \| 1.317699173 \| 0 \| 11 \| \| LY96 \| 0 \| 1.305921877 \| 0 \| 11 \| \| LHFPL2 \| 0 \| 1.30296875 \| 0 \| 11 \| \| 1-Mar \| 0 \| 1.295267998 \| 0 \| 11 \| \| APLP2 \| 0 \| 1.293582632 \| 0 \| 11 \| \| NINJ1 \| 0 \| 1.292112838 \| 0 \| 11 \| \| CYFIP1 \| 0 \| 1.283145655 \| 0 \| 11 \| \| RNF13 \| 0 \| 1.282484956 \| 0 \| 11 \| \| FCER1G \| 0 \| 1.282007333 \| 0 \| 11 \| \| CLEC10A \| 0 \| 1.272555 \| 0 \| 11 \| \| NAMPT \| 0 \| 1.270555318 \| 0 \| 11 \| \| PDK4 \| 0 \| 1.270552079 \| 0 \| 11 \| \| RGS2 \| 0 \| 1.269429737 \| 0 \| 11 \| \| BCL2A1 \| 0 \| 1.263612381 \| 0 \| 11 \| \| SIGLEC10 \| 0 \| 1.259618593 \| 0 \| 11 \| \| MPP1 \| 0 \| 1.257967218 \| 0 \| 11 \| \| ARRB2 \| 0 \| 1.257692609 \| 0 \| 11 \| \| TSPAN4 \| 0 \| 1.254544513 \| 0 \| 11 \| \| PLIN2 \| 0 \| 1.251275415 \| 0 \| 11 \| \| LAP3 \| 0 \| 1.24721619 \| 0 \| 11 \| \| FAM49B \| 0 \| 1.234784914 \| 0 \| 11 \| \| HBEGF \| 0 \| 1.234429695 \| 0 \| 11 \| \| ITGAX \| 0 \| 1.231886469 \| 0 \| 11 \| \| ALCAM \| 0 \| 1.228390479 \| 0 \| 11 \| \| QKI \| 0 \| 1.228079086 \| 0 \| 11 \| \| ADAMDEC1 \| 0 \| 1.226005509 \| 0 \| 11 \| \| CTSA \| 0 \| 1.215709446 \| 0 \| 11 \| \| SNX10 \| 0 \| 1.21168328 \| 0 \| 11 \| \| LY86 \| 0 \| 1.211355056 \| 0 \| 11 \| \| RGL1 \| 0 \| 1.207631861 \| 0 \| 11 \| \| TFEC \| 0 \| 1.206218285 \| 0 \| 11 \| \| IL4I1 \| 0 \| 1.202690494 \| 0 \| 11 \| \| TBXAS1 \| 0 \| 1.200731877 \| 0 \| 11 \| \| SRGN \| 0 \| 1.19827205 \| 0 \| 11 \| \| CCR1 \| 0 \| 1.19350202 \| 0 \| 11 \| \| AOAH \| 0 \| 1.185283573 \| 0 \| 11 \| \| HAVCR2 \| 0 \| 1.182934117 \| 0 \| 11 \| \| NCOA4 \| 0 \| 1.178702966 \| 0 \| 11 \| \| NR4A3 \| 0 \| 1.17485394 \| 0 \| 11 \| \| BCAT1 \| 0 \| 1.17354383 \| 0 \| 11 \| \| PRNP \| 0 \| 1.172866824 \| 0 \| 11 \| \| FAM49A \| 0 \| 1.168869668 \| 0 \| 11 \| \| BMP2K \| 0 \| 1.159782501 \| 0 \| 11 \| \| PTAFR \| 0 \| 1.15915917 \| 0 \| 11 \| \| RAB20 \| 0 \| 1.157059261 \| 0 \| 11 \| \| SH2B3 \| 0 \| 1.152928526 \| 0 \| 11 \| \| RHOA \| 0 \| 1.151695955 \| 0 \| 11 \| \| CD209 \| 0 \| 1.149198304 \| 0 \| 11 \| \| AP1B1 \| 0 \| 1.145862024 \| 0 \| 11 \| \| SGPL1 \| 0 \| 1.140783565 \| 0 \| 11 \| \| THBD \| 0 \| 1.119099012 \| 0 \| 11 \| \| MERTK \| 0 \| 1.119075427 \| 0 \| 11 \| \| CYBA \| 0 \| 1.114698694 \| 0 \| 11 \| \| EPB41L3 \| 0 \| 1.11139796 \| 0 \| 11 \| \| DAPK1 \| 0 \| 1.104944366 \| 0 \| 11 \| \| OTUD1 \| 0 \| 1.104135572 \| 0 \| 11 \| \| FNIP2 \| 0 \| 1.103624266 \| 0 \| 11 \| \| THEMIS2 \| 0 \| 1.101950096 \| 0 \| 11 \| \| UCP2 \| 0 \| 1.100945307 \| 0 \| 11 \| \| PILRA \| 0 \| 1.100770182 \| 0 \| 11 \| \| SOAT1 \| 0 \| 1.097270168 \| 0 \| 11 \| \| CD36 \| 0 \| 1.097215909 \| 0 \| 11 \| \| RNF144B \| 0 \| 1.096430791 \| 0 \| 11 \| \| SCARB2 \| 0 \| 1.094128158 \| 0 \| 11 \| \| RAC1 \| 0 \| 1.09209613 \| 0 \| 11 \| \| LCP1 \| 0 \| 1.091757209 \| 0 \| 11 \| \| PEA15 \| 0 \| 1.089769547 \| 0 \| 11 \| \| DOCK4 \| 0 \| 1.088232783 \| 0 \| 11 \| \| SPRED1 \| 0 \| 1.086457312 \| 0 \| 11 \| \| LILRB5 \| 0 \| 1.08182955 \| 0 \| 11 \| \| CD53 \| 0 \| 1.081541997 \| 0 \| 11 \| \| LRP1 \| 0 \| 1.077175668 \| 0 \| 11 \| \| H2AFY \| 0 \| 1.076341335 \| 0 \| 11 \| \| GLA \| 0 \| 1.074524859 \| 0 \| 11 \| \| ITGAM \| 0 \| 1.072727709 \| 0 \| 11 \| \| ITPR2 \| 0 \| 1.069101683 \| 0 \| 11 \| \| SLC1A3 \| 0 \| 1.064902346 \| 0 \| 11 \| \| NPL \| 0 \| 1.062438926 \| 0 \| 11 \| \| HCK \| 0 \| 1.061551831 \| 0 \| 11 \| \| S100B \| 0 \| 1.061056044 \| 0 \| 11 \| \| CFD \| 0 \| 1.059905925 \| 0 \| 11 \| \| CYP27A1 \| 0 \| 1.059790321 \| 0 \| 11 \| \| IRF8 \| 0 \| 1.057857338 \| 0 \| 11 \| \| S100A9 \| 0 \| 1.056070516 \| 0 \| 11 \| \| HCLS1 \| 0 \| 1.052799649 \| 0 \| 11 \| \| ADGRE2 \| 0 \| 1.047206081 \| 0 \| 11 \| \| CD302 \| 0 \| 1.044804176 \| 0 \| 11 \| \| NCKAP1L \| 0 \| 1.042327234 \| 0 \| 11 \| \| GNA13 \| 0 \| 1.041132946 \| 0 \| 11 \| \| PTPRE \| 0 \| 1.032396957 \| 0 \| 11 \| \| CMKLR1 \| 0 \| 1.032324192 \| 0 \| 11 \| \| SNX2 \| 0 \| 1.029064505 \| 0 \| 11 \| \| FMNL2 \| 0 \| 1.025258811 \| 0 \| 11 \| \| SOD2 \| 0 \| 1.02499678 \| 0 \| 11 \| \| RNASET2 \| 0 \| 1.022907536 \| 0 \| 11 \| \| FERMT3 \| 0 \| 1.017705053 \| 0 \| 11 \| \| SDC3 \| 0 \| 1.01658996 \| 0 \| 11 \| \| RTN4 \| 0 \| 1.014988795 \| 0 \| 11 \| \| FCGR1A \| 0 \| 1.014901032 \| 0 \| 11 \| \| RAB32 \| 0 \| 1.012940536 \| 0 \| 11 \| \| COLGALT1 \| 0 \| 1.002156931 \| 0 \| 11 \| \| ADAM9 \| 0 \| 1.001603468 \| 0 \| 11 \| \| PHACTR1 \| 0 \| 1.000764291 \| 0 \| 11 \| \| TUBB \| 3.0984E-307 \| 1.15434185 \| 6.7628E-303 \| 11 \| \| TNF \| 1.9995E-306 \| 1.187676 \| 4.3644E-302 \| 11 \| \| ATP6V0E1 \| 3.8334E-301 \| 1.005143178 \| 8.3671E-297 \| 11 \| \| CAP1 \| 4.6859E-295 \| 1.009202297 \| 1.0228E-290 \| 11 \| \| ATF3 \| 1.1146E-293 \| 1.171222155 \| 2.4328E-289 \| 11 \| \| CSTB \| 1.7058E-292 \| 1.615215117 \| 3.7232E-288 \| 11 \| \| ACTR2 \| 3.417E-281 \| 1.056515082 \| 7.4582E-277 \| 11 \| \| ANXA2 \| 1.0585E-276 \| 1.108825611 \| 2.3105E-272 \| 11 \| \| EGR2 \| 7.8526E-276 \| 1.119910269 \| 1.714E-271 \| 11 \| \| NR4A2 \| 1.2424E-274 \| 1.245530199 \| 2.7118E-270 \| 11 \| \| TUBA1B \| 1.9021E-271 \| 1.191657899 \| 4.1517E-267 \| 11 \| \| ZFAND5 \| 9.717E-269 \| 1.133459169 \| 2.1209E-264 \| 11 \| \| DUSP1 \| 1.1211E-255 \| 1.148422746 \| 2.4471E-251 \| 11 \| \| IER3 \| 3.1252E-252 \| 1.440740025 \| 6.8213E-248 \| 11 \| \| SELENOP \| 2.6529E-250 \| 1.988796262 \| 5.7905E-246 \| 11 \| \| GBP1 \| 2.8069E-231 \| 1.009098266 \| 6.1267E-227 \| 11 \| \| MALAT1 \| 2.5178E-221 \| -1.317561031 \| 5.4955E-217 \| 11 \| \| RPS18 \| 5.6388E-214 \| -1.288790707 \| 1.2308E-209 \| 11 \| \| RPS27 \| 4.799E-200 \| -1.470952392 \| 1.0475E-195 \| 11 \| \| HSPA1A \| 3.3276E-196 \| 1.032240583 \| 7.2631E-192 \| 11 \| \| NFKBIA \| 2.007E-192 \| 1.255425265 \| 4.3808E-188 \| 11 \| \| RPL36 \| 5.2609E-189 \| -1.166813541 \| 1.1483E-184 \| 11 \| \| SYNE2 \| 6.6572E-183 \| -1.820770899 \| 1.4531E-178 \| 11 \| \| CXCL1 \| 5.3128E-177 \| 1.465377184 \| 1.1596E-172 \| 11 \| \| RPL35A \| 3.6874E-170 \| -1.028879087 \| 8.0485E-166 \| 11 \| \| EREG \| 3.9898E-164 \| 1.211566945 \| 8.7086E-160 \| 11 \| \| RPL34 \| 9.307E-160 \| -1.091760095 \| 2.0314E-155 \| 11 \| \| RPL13 \| 1.1268E-151 \| -1.028776491 \| 2.4594E-147 \| 11 \| \| NUPR1 \| 2.1042E-134 \| 1.084061968 \| 4.5929E-130 \| 11 \| \| SPTBN1 \| 5.4477E-126 \| -1.555638891 \| 1.1891E-121 \| 11 \| \| RPS21 \| 5.3723E-120 \| -1.164020273 \| 1.1726E-115 \| 11 \| \| SLC38A1 \| 1.3181E-115 \| -1.194038386 \| 2.8771E-111 \| 11 \| \| ISG20 \| 1.8289E-113 \| -1.424866464 \| 3.992E-109 \| 11 \| \| RPS29 \| 3.0771E-112 \| -1.092696456 \| 6.7164E-108 \| 11 \| \| KRT18 \| 8.6126E-110 \| -2.933801013 \| 1.8799E-105 \| 11 \| \| CLDN3 \| 4.9041E-109 \| -2.172671416 \| 1.0704E-104 \| 11 \| \| KRT8 \| 5.241E-108 \| -2.881291322 \| 1.1439E-103 \| 11 \| \| LGALS4 \| 7.0458E-106 \| -2.644487982 \| 1.5379E-101 \| 11 \| \| EPCAM \| 1.4314E-105 \| -2.090740053 \| 3.1242E-101 \| 11 \| \| ELF3 \| 1.0522E-103 \| -2.299191224 \| 2.2967E-99 \| 11 \| \| FKBP11 \| 2.6606E-103 \| -1.270625633 \| 5.8074E-99 \| 11 \| \| RPS26 \| 1.793E-102 \| -1.154231735 \| 3.91358E-98 \| 11 \| \| TMEM238 \| 2.2354E-100 \| -1.320721093 \| 4.87916E-96 \| 11 \| \| PHGR1 \| 1.4072E-99 \| -3.651700228 \| 3.07159E-95 \| 11 \| \| IGKC \| 5.4897E-99 \| -4.106599231 \| 1.19824E-94 \| 11 \| \| CAMK2N1 \| 1.01885E-97 \| -1.405527511 \| 2.22385E-93 \| 11 \| \| KRT19 \| 1.05691E-97 \| -2.747643699 \| 2.30692E-93 \| 11 \| \| CDC42EP5 \| 5.84548E-96 \| -1.495848247 \| 1.27589E-91 \| 11 \| \| SMIM22 \| 2.52742E-95 \| -1.635391989 \| 5.51659E-91 \| 11 \| \| CLDN4 \| 1.29244E-94 \| -2.254200076 \| 2.821E-90 \| 11 \| \| DSP \| 1.40775E-94 \| -1.499094231 \| 3.07269E-90 \| 11 \| \| PERP \| 3.01451E-93 \| -1.41374358 \| 6.57977E-89 \| 11 \| \| OCIAD2 \| 6.56312E-93 \| -1.034507099 \| 1.43253E-88 \| 11 \| \| TSPAN8 \| 9.86759E-93 \| -2.283837065 \| 2.1538E-88 \| 11 \| \| IFI27 \| 5.07177E-91 \| -2.436226817 \| 1.10701E-86 \| 11 \| \| PDLIM1 \| 6.24236E-90 \| -1.136735169 \| 1.36252E-85 \| 11 \| \| C12orf57 \| 3.85191E-89 \| -1.207700255 \| 8.40757E-85 \| 11 \| \| AGR2 \| 4.1813E-89 \| -2.512906684 \| 9.12653E-85 \| 11 \| \| ITM2C \| 7.69099E-88 \| -1.635194367 \| 1.67871E-83 \| 11 \| \| THBS1 \| 7.90301E-88 \| 1.120424968 \| 1.72499E-83 \| 11 \| \| RPL31 \| 3.63638E-85 \| -1.036697823 \| 7.93713E-81 \| 11 \| \| SLC44A4 \| 5.86282E-83 \| -1.304963509 \| 1.27968E-78 \| 11 \| \| TMEM54 \| 6.89665E-81 \| -1.55371981 \| 1.50533E-76 \| 11 \| \| CD24 \| 3.10418E-80 \| -1.766895887 \| 6.7755E-76 \| 11 \| \| TUBA4A \| 9.74441E-80 \| -1.115684354 \| 2.12691E-75 \| 11 \| \| FAM3D \| 1.78642E-79 \| -1.655710853 \| 3.89922E-75 \| 11 \| \| S100A14 \| 1.09848E-78 \| -1.345464289 \| 2.39764E-74 \| 11 \| \| CD3D \| 1.26059E-77 \| -2.039296477 \| 2.75149E-73 \| 11 \| \| MUC13 \| 4.37439E-77 \| -1.523258123 \| 9.54798E-73 \| 11 \| \| TENT5C \| 5.82449E-77 \| -1.148188545 \| 1.27131E-72 \| 11 \| \| PIGR \| 3.9155E-75 \| -3.53110317 \| 8.54635E-71 \| 11 \| \| FXYD3 \| 8.11012E-74 \| -2.353353303 \| 1.77019E-69 \| 11 \| \| TSPAN1 \| 1.07467E-73 \| -2.053164658 \| 2.34568E-69 \| 11 \| \| CDH17 \| 3.43787E-73 \| -1.177721826 \| 7.50383E-69 \| 11 \| \| TFF3 \| 2.57556E-71 \| -3.093667572 \| 5.62168E-67 \| 11 \| \| KLF5 \| 2.95954E-70 \| -1.312321096 \| 6.45978E-66 \| 11 \| \| TRAC \| 9.16219E-69 \| -1.789627956 \| 1.99983E-64 \| 11 \| \| GPX2 \| 1.34558E-68 \| -1.588009597 \| 2.93701E-64 \| 11 \| \| ITGA6 \| 1.56722E-66 \| -1.129030169 \| 3.42077E-62 \| 11 \| \| ID1 \| 1.05968E-64 \| -1.339529005 \| 2.31297E-60 \| 11 \| \| CD2 \| 1.18983E-64 \| -1.910362945 \| 2.59705E-60 \| 11 \| \| CKB \| 1.88019E-61 \| -2.08414711 \| 4.10388E-57 \| 11 \| \| ARGLU1 \| 9.73722E-61 \| -1.184132128 \| 2.12534E-56 \| 11 \| \| PPDPF \| 1.27356E-59 \| -1.18854969 \| 2.77979E-55 \| 11 \| \| LCN2 \| 6.75185E-55 \| -2.278795125 \| 1.47373E-50 \| 11 \| \| CD69 \| 2.36601E-42 \| -2.059428426 \| 5.16429E-38 \| 11 \| \| FABP1 \| 5.11891E-41 \| -3.177561193 \| 1.1173E-36 \| 11 \| \| PDCD4 \| 9.29772E-41 \| -1.089990289 \| 2.02941E-36 \| 11 \| \| TPM1 \| 1.20406E-40 \| -1.217577995 \| 2.6281E-36 \| 11 \| \| SLC12A2 \| 5.99689E-33 \| -1.225689141 \| 1.30894E-28 \| 11 \| \| JCHAIN \| 1.42742E-32 \| -3.89241356 \| 3.11564E-28 \| 11 \| \| AC058791.1 \| 4.47678E-29 \| -1.168006107 \| 9.77147E-25 \| 11 \| \| IGHA1 \| 5.04084E-24 \| -3.567879512 \| 1.10026E-19 \| 11 \| \| CD52 \| 9.60595E-23 \| -1.622371665 \| 2.09669E-18 \| 11 \| \| S100A6 \| 1.12233E-22 \| -1.431229035 \| 2.44971E-18 \| 11 \| \| SSR4 \| 3.20541E-20 \| -1.171780161 \| 6.99645E-16 \| 11 \| \| IGLC3 \| 2.96675E-10 \| -4.62543273 \| 6.47552E-06 \| 11 \| \| PI3 \| 0 \| 3.490697199 \| 0 \| 12 \| \| MMP7 \| 0 \| 3.106657295 \| 0 \| 12 \| \| LCN2 \| 0 \| 2.793992325 \| 0 \| 12 \| \| DUOXA2 \| 0 \| 2.691961554 \| 0 \| 12 \| \| TFF1 \| 0 \| 2.55554341 \| 0 \| 12 \| \| PERP \| 0 \| 2.408610892 \| 0 \| 12 \| \| KLK10 \| 0 \| 2.320824224 \| 0 \| 12 \| \| SLPI \| 0 \| 2.309187987 \| 0 \| 12 \| \| LMO7 \| 0 \| 2.244052837 \| 0 \| 12 \| \| S100P \| 0 \| 2.208180261 \| 0 \| 12 \| \| CEACAM6 \| 0 \| 2.171809382 \| 0 \| 12 \| \| TACSTD2 \| 0 \| 2.14575617 \| 0 \| 12 \| \| LAMB3 \| 0 \| 2.120768926 \| 0 \| 12 \| \| GPRC5A \| 0 \| 2.080265025 \| 0 \| 12 \| \| S100A11 \| 0 \| 2.062483746 \| 0 \| 12 \| \| LAMC2 \| 0 \| 2.057092838 \| 0 \| 12 \| \| S100A6 \| 0 \| 2.037364013 \| 0 \| 12 \| \| KRT18 \| 0 \| 2.011684242 \| 0 \| 12 \| \| ATP1B1 \| 0 \| 2.007474646 \| 0 \| 12 \| \| ANXA2 \| 0 \| 1.989559573 \| 0 \| 12 \| \| DUOX2 \| 0 \| 1.956241047 \| 0 \| 12 \| \| S100A16 \| 0 \| 1.949492803 \| 0 \| 12 \| \| PMEPA1 \| 0 \| 1.913467662 \| 0 \| 12 \| \| KRT17 \| 0 \| 1.913461226 \| 0 \| 12 \| \| EFNA1 \| 0 \| 1.895461827 \| 0 \| 12 \| \| TM4SF1 \| 0 \| 1.88038427 \| 0 \| 12 \| \| KRT8 \| 0 \| 1.86730951 \| 0 \| 12 \| \| SLC7A11 \| 0 \| 1.850505185 \| 0 \| 12 \| \| SLC2A1 \| 0 \| 1.826420181 \| 0 \| 12 \| \| TSPAN8 \| 0 \| 1.815427903 \| 0 \| 12 \| \| TFF2 \| 0 \| 1.778073472 \| 0 \| 12 \| \| FOXQ1 \| 0 \| 1.766176026 \| 0 \| 12 \| \| ABHD2 \| 0 \| 1.74095099 \| 0 \| 12 \| \| CLDN4 \| 0 \| 1.737714569 \| 0 \| 12 \| \| S100A14 \| 0 \| 1.716038066 \| 0 \| 12 \| \| KRT19 \| 0 \| 1.713427168 \| 0 \| 12 \| \| PDZK1IP1 \| 0 \| 1.695226424 \| 0 \| 12 \| \| S100A10 \| 0 \| 1.666046567 \| 0 \| 12 \| \| KLF5 \| 0 \| 1.620187423 \| 0 \| 12 \| \| ELF3 \| 0 \| 1.604800031 \| 0 \| 12 \| \| ITGA2 \| 0 \| 1.590526154 \| 0 \| 12 \| \| MSLN \| 0 \| 1.579389921 \| 0 \| 12 \| \| LAMA3 \| 0 \| 1.541685764 \| 0 \| 12 \| \| DSP \| 0 \| 1.531288777 \| 0 \| 12 \| \| AQP5 \| 0 \| 1.528576648 \| 0 \| 12 \| \| PHLDA2 \| 0 \| 1.527655107 \| 0 \| 12 \| \| CAMK2N1 \| 0 \| 1.502338104 \| 0 \| 12 \| \| DSG2 \| 0 \| 1.475113086 \| 0 \| 12 \| \| CCND1 \| 0 \| 1.47504519 \| 0 \| 12 \| \| GPX2 \| 0 \| 1.474863325 \| 0 \| 12 \| \| CD24 \| 0 \| 1.46713025 \| 0 \| 12 \| \| ANXA11 \| 0 \| 1.435850117 \| 0 \| 12 \| \| COL17A1 \| 0 \| 1.423381971 \| 0 \| 12 \| \| CD9 \| 0 \| 1.423295851 \| 0 \| 12 \| \| MAL2 \| 0 \| 1.419347271 \| 0 \| 12 \| \| TRIM29 \| 0 \| 1.41817608 \| 0 \| 12 \| \| SERPINB5 \| 0 \| 1.414239414 \| 0 \| 12 \| \| GDF15 \| 0 \| 1.399032763 \| 0 \| 12 \| \| EPCAM \| 0 \| 1.396545986 \| 0 \| 12 \| \| TINAGL1 \| 0 \| 1.392369457 \| 0 \| 12 \| \| CTSE \| 0 \| 1.390344193 \| 0 \| 12 \| \| CD46 \| 0 \| 1.38889972 \| 0 \| 12 \| \| HMGA1 \| 0 \| 1.373162568 \| 0 \| 12 \| \| GSTP1 \| 0 \| 1.372269491 \| 0 \| 12 \| \| SDC4 \| 0 \| 1.366745952 \| 0 \| 12 \| \| PON2 \| 0 \| 1.365333057 \| 0 \| 12 \| \| SCD \| 0 \| 1.361983606 \| 0 \| 12 \| \| RAB25 \| 0 \| 1.353193327 \| 0 \| 12 \| \| ERO1A \| 0 \| 1.349682718 \| 0 \| 12 \| \| DDR1 \| 0 \| 1.345531873 \| 0 \| 12 \| \| MET \| 0 \| 1.343529266 \| 0 \| 12 \| \| TESC \| 0 \| 1.340476267 \| 0 \| 12 \| \| ANXA3 \| 0 \| 1.337459047 \| 0 \| 12 \| \| FERMT1 \| 0 \| 1.331632242 \| 0 \| 12 \| \| PKM \| 0 \| 1.311002679 \| 0 \| 12 \| \| MYO6 \| 0 \| 1.310776477 \| 0 \| 12 \| \| S100A13 \| 0 \| 1.308947222 \| 0 \| 12 \| \| ANXA4 \| 0 \| 1.299428433 \| 0 \| 12 \| \| ASS1 \| 0 \| 1.296533279 \| 0 \| 12 \| \| ITGA6 \| 0 \| 1.296081071 \| 0 \| 12 \| \| MUC13 \| 0 \| 1.296039615 \| 0 \| 12 \| \| JUP \| 0 \| 1.295947095 \| 0 \| 12 \| \| VNN1 \| 0 \| 1.293966331 \| 0 \| 12 \| \| EFNB2 \| 0 \| 1.290926727 \| 0 \| 12 \| \| LSR \| 0 \| 1.288670239 \| 0 \| 12 \| \| SERPINA1 \| 0 \| 1.280674289 \| 0 \| 12 \| \| H1F0 \| 0 \| 1.255640035 \| 0 \| 12 \| \| F11R \| 0 \| 1.251099242 \| 0 \| 12 \| \| SPRY2 \| 0 \| 1.250651032 \| 0 \| 12 \| \| FHL2 \| 0 \| 1.248931427 \| 0 \| 12 \| \| SOX9 \| 0 \| 1.226613682 \| 0 \| 12 \| \| PLPP2 \| 0 \| 1.222200131 \| 0 \| 12 \| \| BACE2 \| 0 \| 1.197673535 \| 0 \| 12 \| \| HEBP2 \| 0 \| 1.195475193 \| 0 \| 12 \| \| KCNK1 \| 0 \| 1.193164951 \| 0 \| 12 \| \| AGR2 \| 0 \| 1.188950025 \| 0 \| 12 \| \| EMP1 \| 0 \| 1.166330971 \| 0 \| 12 \| \| PRSS22 \| 0 \| 1.157321366 \| 0 \| 12 \| \| SPINT1 \| 0 \| 1.155395532 \| 0 \| 12 \| \| MACC1 \| 0 \| 1.150225418 \| 0 \| 12 \| \| TMC5 \| 0 \| 1.149491983 \| 0 \| 12 \| \| CDH17 \| 0 \| 1.141742205 \| 0 \| 12 \| \| SPTBN1 \| 0 \| 1.134133065 \| 0 \| 12 \| \| CEMIP \| 0 \| 1.132457251 \| 0 \| 12 \| \| FIBCD1 \| 0 \| 1.130711941 \| 0 \| 12 \| \| PLEC \| 0 \| 1.111920697 \| 0 \| 12 \| \| TMPRSS4 \| 0 \| 1.110653494 \| 0 \| 12 \| \| CLDN3 \| 0 \| 1.104198332 \| 0 \| 12 \| \| NDFIP2 \| 0 \| 1.084224467 \| 0 \| 12 \| \| F3 \| 0 \| 1.081037009 \| 0 \| 12 \| \| TRIM31 \| 0 \| 1.062548729 \| 0 \| 12 \| \| MUC17 \| 0 \| 1.058364863 \| 0 \| 12 \| \| TMEM45B \| 0 \| 1.054794534 \| 0 \| 12 \| \| SFN \| 0 \| 1.050759698 \| 0 \| 12 \| \| MDK \| 0 \| 1.045781298 \| 0 \| 12 \| \| FAT1 \| 0 \| 1.04204466 \| 0 \| 12 \| \| TNFRSF12A \| 0 \| 1.040613947 \| 0 \| 12 \| \| DSC2 \| 0 \| 1.014123963 \| 0 \| 12 \| \| BMP4 \| 0 \| 1.011904926 \| 0 \| 12 \| \| EPHA2 \| 0 \| 1.008141979 \| 0 \| 12 \| \| NECTIN4 \| 0 \| 1.008052302 \| 0 \| 12 \| \| QSOX1 \| 0 \| 1.007587404 \| 0 \| 12 \| \| REG4 \| 1.4896E-306 \| 1.760921887 \| 3.2514E-302 \| 12 \| \| CXCL1 \| 1.1883E-304 \| 1.286118673 \| 2.5937E-300 \| 12 \| \| TXN \| 2.5711E-292 \| 1.256315295 \| 5.6119E-288 \| 12 \| \| TMSB10 \| 3.2311E-287 \| 1.072020304 \| 7.0524E-283 \| 12 \| \| SERPINB1 \| 2.4515E-277 \| 1.14377877 \| 5.3509E-273 \| 12 \| \| SOX4 \| 5.453E-271 \| 1.198320291 \| 1.1902E-266 \| 12 \| \| CD55 \| 5.5057E-260 \| 1.067207366 \| 1.2017E-255 \| 12 \| \| CSTB \| 6.7664E-259 \| 1.140373291 \| 1.4769E-254 \| 12 \| \| NDRG1 \| 1.6545E-258 \| 1.18584919 \| 3.6112E-254 \| 12 \| \| LGALS3 \| 2.3186E-256 \| 1.10749436 \| 5.0609E-252 \| 12 \| \| SRGN \| 4.7813E-256 \| -3.723501684 \| 1.0436E-251 \| 12 \| \| VIM \| 1.1802E-253 \| -3.567150994 \| 2.576E-249 \| 12 \| \| RAC1 \| 2.1226E-252 \| 1.066611494 \| 4.633E-248 \| 12 \| \| TGFBI \| 7.2155E-245 \| 1.133758444 \| 1.5749E-240 \| 12 \| \| CLTB \| 1.1565E-244 \| 1.181618297 \| 2.5243E-240 \| 12 \| \| LYZ \| 2.485E-233 \| 1.153488055 \| 5.424E-229 \| 12 \| \| SQSTM1 \| 5.2045E-232 \| 1.02513479 \| 1.136E-227 \| 12 \| \| KRAS \| 2.6207E-224 \| 1.020707797 \| 5.7202E-220 \| 12 \| \| DNAJC15 \| 7.907E-224 \| 1.026689729 \| 1.7259E-219 \| 12 \| \| TSC22D3 \| 3.7362E-222 \| -2.77028987 \| 8.1549E-218 \| 12 \| \| PTPRC \| 1.7694E-185 \| -2.700034884 \| 3.862E-181 \| 12 \| \| IGKC \| 2.6566E-182 \| -6.27949305 \| 5.7986E-178 \| 12 \| \| RGS1 \| 9.4875E-160 \| -2.747540345 \| 2.0708E-155 \| 12 \| \| HSPB1 \| 2.4647E-159 \| 1.098097488 \| 5.3796E-155 \| 12 \| \| PCCA \| 3.9678E-156 \| 1.006358449 \| 8.6606E-152 \| 12 \| \| CYTIP \| 1.3952E-143 \| -1.782989013 \| 3.0453E-139 \| 12 \| \| CD48 \| 1.6741E-139 \| -1.832921453 \| 3.6541E-135 \| 12 \| \| CD52 \| 1.9065E-133 \| -2.586412494 \| 4.1612E-129 \| 12 \| \| GMFG \| 7.9981E-132 \| -1.5581412 \| 1.7458E-127 \| 12 \| \| PHGR1 \| 1.5091E-113 \| -1.229280518 \| 3.294E-109 \| 12 \| \| LAPTM5 \| 5.0752E-111 \| -2.029735219 \| 1.1078E-106 \| 12 \| \| CXCR4 \| 5.9349E-109 \| -3.032210943 \| 1.2954E-104 \| 12 \| \| LCP1 \| 3.3806E-108 \| -1.88083756 \| 7.3789E-104 \| 12 \| \| ARHGDIB \| 2.6326E-105 \| -1.918840643 \| 5.7462E-101 \| 12 \| \| GYPC \| 5.8232E-105 \| -1.106433995 \| 1.271E-100 \| 12 \| \| CD53 \| 1.7983E-104 \| -1.511741101 \| 3.9251E-100 \| 12 \| \| LSP1 \| 3.5037E-103 \| -1.31034233 \| 7.6475E-99 \| 12 \| \| DUSP1 \| 4.7947E-101 \| -1.329905933 \| 1.04654E-96 \| 12 \| \| CD69 \| 7.4818E-99 \| -2.54944296 \| 1.63304E-94 \| 12 \| \| CD37 \| 1.43321E-98 \| -1.585529306 \| 3.12826E-94 \| 12 \| \| HCST \| 7.15717E-94 \| -1.705056926 \| 1.5622E-89 \| 12 \| \| TMSB4X \| 3.25586E-92 \| -1.126186 \| 7.10656E-88 \| 12 \| \| ZNF331 \| 1.66496E-91 \| -1.928877708 \| 3.63412E-87 \| 12 \| \| JUND \| 5.96402E-88 \| -1.045308578 \| 1.30177E-83 \| 12 \| \| CD3D \| 8.24771E-87 \| -2.171085437 \| 1.80023E-82 \| 12 \| \| GPR183 \| 3.45527E-86 \| -1.911517866 \| 7.54183E-82 \| 12 \| \| SAMSN1 \| 2.87233E-82 \| -1.563897009 \| 6.26944E-78 \| 12 \| \| EVI2B \| 3.24928E-82 \| -1.057443209 \| 7.09221E-78 \| 12 \| \| CD2 \| 3.37817E-82 \| -2.079812033 \| 7.37354E-78 \| 12 \| \| IL7R \| 5.86051E-82 \| -3.070722642 \| 1.27917E-77 \| 12 \| \| FYB1 \| 7.92118E-82 \| -1.856904656 \| 1.72896E-77 \| 12 \| \| RPS26 \| 9.72429E-82 \| -1.035881571 \| 2.12252E-77 \| 12 \| \| HCLS1 \| 1.12445E-81 \| -1.049232162 \| 2.45433E-77 \| 12 \| \| TRAC \| 2.27152E-81 \| -1.936452858 \| 4.95804E-77 \| 12 \| \| GLIPR1 \| 5.24845E-79 \| -1.236184849 \| 1.14558E-74 \| 12 \| \| LGALS1 \| 3.73629E-77 \| -2.025109432 \| 8.15521E-73 \| 12 \| \| SMAP2 \| 4.53874E-77 \| -1.545177571 \| 9.90671E-73 \| 12 \| \| CLEC2B \| 9.44399E-77 \| -1.414393031 \| 2.06134E-72 \| 12 \| \| TAGAP \| 8.48384E-75 \| -1.197837184 \| 1.85177E-70 \| 12 \| \| FNBP1 \| 1.29155E-71 \| -1.140705244 \| 2.81906E-67 \| 12 \| \| EMP3 \| 2.22937E-71 \| -1.254906301 \| 4.86605E-67 \| 12 \| \| PDE4B \| 4.63848E-69 \| -1.072764573 \| 1.01244E-64 \| 12 \| \| JUNB \| 5.64705E-69 \| -1.195763268 \| 1.23258E-64 \| 12 \| \| FKBP5 \| 1.09905E-64 \| -1.200842494 \| 2.39889E-60 \| 12 \| \| TENT5C \| 2.4209E-62 \| -1.154800369 \| 5.28411E-58 \| 12 \| \| SLC2A3 \| 2.3244E-61 \| -1.323123102 \| 5.07346E-57 \| 12 \| \| HLA-DPB1 \| 1.49702E-60 \| -2.475021383 \| 3.26754E-56 \| 12 \| \| BTG1 \| 2.79468E-59 \| -1.503278406 \| 6.09995E-55 \| 12 \| \| MSN \| 8.82038E-57 \| -1.117705978 \| 1.92522E-52 \| 12 \| \| JCHAIN \| 2.0193E-56 \| -6.126154438 \| 4.40752E-52 \| 12 \| \| HLA-DPA1 \| 3.01732E-55 \| -2.413104767 \| 6.5859E-51 \| 12 \| \| RAC2 \| 1.04211E-54 \| -1.267539797 \| 2.27461E-50 \| 12 \| \| HERPUD1 \| 4.61296E-54 \| -1.36886193 \| 1.00687E-49 \| 12 \| \| DUSP2 \| 1.60367E-50 \| -1.631669528 \| 3.50034E-46 \| 12 \| \| TNFAIP3 \| 2.08424E-50 \| -2.225928308 \| 4.54927E-46 \| 12 \| \| CREM \| 2.20185E-50 \| -1.832893358 \| 4.80598E-46 \| 12 \| \| AC058791.1 \| 5.38215E-49 \| -1.389841757 \| 1.17476E-44 \| 12 \| \| CD74 \| 2.4825E-46 \| -1.99779002 \| 5.41854E-42 \| 12 \| \| PRDM1 \| 3.42713E-45 \| -1.220240277 \| 7.4804E-41 \| 12 \| \| PDCD4 \| 1.21971E-42 \| -1.190312734 \| 2.66226E-38 \| 12 \| \| RGS2 \| 6.6398E-42 \| -1.412242477 \| 1.44927E-37 \| 12 \| \| NFKBIA \| 3.99424E-41 \| -1.167680625 \| 8.71822E-37 \| 12 \| \| STK17B \| 1.26226E-40 \| -1.336553965 \| 2.75514E-36 \| 12 \| \| IGHA1 \| 3.30808E-39 \| -5.141880296 \| 7.22054E-35 \| 12 \| \| FCGBP \| 1.09008E-35 \| -1.663697389 \| 2.37931E-31 \| 12 \| \| SOCS3 \| 4.9182E-33 \| -1.044316298 \| 1.0735E-28 \| 12 \| \| DNAJA1 \| 1.15657E-29 \| -1.111640827 \| 2.52444E-25 \| 12 \| \| SARAF \| 1.40835E-28 \| -1.041527389 \| 3.074E-24 \| 12 \| \| FAM107B \| 9.86003E-28 \| -1.025504635 \| 2.15215E-23 \| 12 \| \| FKBP11 \| 1.18551E-27 \| -1.041739323 \| 2.58761E-23 \| 12 \| \| NR4A2 \| 9.16871E-26 \| -1.419693431 \| 2.00125E-21 \| 12 \| \| ARL4C \| 8.36703E-23 \| -1.300022897 \| 1.82627E-18 \| 12 \| \| HLA-DRA \| 9.45785E-22 \| -3.272145077 \| 2.06437E-17 \| 12 \| \| TUBA1A \| 9.68878E-22 \| -1.030956539 \| 2.11477E-17 \| 12 \| \| IGLC3 \| 4.91833E-21 \| -4.698862178 \| 1.07352E-16 \| 12 \| \| RGCC \| 3.07539E-18 \| -1.292451961 \| 6.71264E-14 \| 12 \| \| COTL1 \| 2.53267E-17 \| -1.25922686 \| 5.52806E-13 \| 12 \| \| IGLC2 \| 2.89E-15 \| -6.262947346 \| 6.30801E-11 \| 12 \| \| KLF2 \| 7.84501E-14 \| -1.08287859 \| 1.71233E-09 \| 12 \| \| CNOT6L \| 1.97899E-12 \| -1.027877774 \| 4.31953E-08 \| 12 \| \| SSR4 \| 4.84757E-09 \| -1.066995826 \| 0.000105808 \| 12 \| \| COL1A1 \| 0 \| 6.252881148 \| 0 \| 13 \| \| COL1A2 \| 0 \| 6.111992548 \| 0 \| 13 \| \| COL3A1 \| 0 \| 6.098206203 \| 0 \| 13 \| \| LUM \| 0 \| 5.978826686 \| 0 \| 13 \| \| MGP \| 0 \| 5.766422155 \| 0 \| 13 \| \| CXCL14 \| 0 \| 5.509176876 \| 0 \| 13 \| \| DCN \| 0 \| 4.729704912 \| 0 \| 13 \| \| SFRP2 \| 0 \| 4.621180094 \| 0 \| 13 \| \| COL6A3 \| 0 \| 4.487025891 \| 0 \| 13 \| \| SPARC \| 0 \| 4.249479138 \| 0 \| 13 \| \| IGFBP5 \| 0 \| 4.217439365 \| 0 \| 13 \| \| CCL11 \| 0 \| 4.108139029 \| 0 \| 13 \| \| IGFBP7 \| 0 \| 4.026940783 \| 0 \| 13 \| \| POSTN \| 0 \| 4.008811351 \| 0 \| 13 \| \| COL6A2 \| 0 \| 3.984067794 \| 0 \| 13 \| \| CFD \| 0 \| 3.979318357 \| 0 \| 13 \| \| C1R \| 0 \| 3.969569759 \| 0 \| 13 \| \| C1S \| 0 \| 3.904729166 \| 0 \| 13 \| \| RARRES2 \| 0 \| 3.859653944 \| 0 \| 13 \| \| ADAMDEC1 \| 0 \| 3.858736622 \| 0 \| 13 \| \| COL6A1 \| 0 \| 3.745142322 \| 0 \| 13 \| \| SERPINF1 \| 0 \| 3.68766181 \| 0 \| 13 \| \| FBLN1 \| 0 \| 3.644307705 \| 0 \| 13 \| \| TIMP1 \| 0 \| 3.535546708 \| 0 \| 13 \| \| MMP2 \| 0 \| 3.488681313 \| 0 \| 13 \| \| PTGDS \| 0 \| 3.455015788 \| 0 \| 13 \| \| CALD1 \| 0 \| 3.427909871 \| 0 \| 13 \| \| APOD \| 0 \| 3.421330567 \| 0 \| 13 \| \| CTSK \| 0 \| 3.419612693 \| 0 \| 13 \| \| THY1 \| 0 \| 3.385146816 \| 0 \| 13 \| \| CCDC80 \| 0 \| 3.340012934 \| 0 \| 13 \| \| COL5A2 \| 0 \| 3.28251634 \| 0 \| 13 \| \| BGN \| 0 \| 3.278962114 \| 0 \| 13 \| \| CTGF \| 0 \| 3.239919715 \| 0 \| 13 \| \| AEBP1 \| 0 \| 3.215482549 \| 0 \| 13 \| \| SULF1 \| 0 \| 3.20016071 \| 0 \| 13 \| \| LGALS1 \| 0 \| 3.17014692 \| 0 \| 13 \| \| C11orf96 \| 0 \| 3.154774151 \| 0 \| 13 \| \| TIMP3 \| 0 \| 3.137610594 \| 0 \| 13 \| \| NNMT \| 0 \| 3.109724381 \| 0 \| 13 \| \| CTHRC1 \| 0 \| 3.10509236 \| 0 \| 13 \| \| MMP11 \| 0 \| 3.065556592 \| 0 \| 13 \| \| C3 \| 0 \| 3.042647279 \| 0 \| 13 \| \| COL12A1 \| 0 \| 3.031423559 \| 0 \| 13 \| \| SPON2 \| 0 \| 3.019992225 \| 0 \| 13 \| \| PCOLCE \| 0 \| 3.013679399 \| 0 \| 13 \| \| SERPINE2 \| 0 \| 2.938723644 \| 0 \| 13 \| \| C7 \| 0 \| 2.903200744 \| 0 \| 13 \| \| FN1 \| 0 \| 2.901800344 \| 0 \| 13 \| \| SFRP4 \| 0 \| 2.894825506 \| 0 \| 13 \| \| TPM2 \| 0 \| 2.893842566 \| 0 \| 13 \| \| IGFBP4 \| 0 \| 2.89143909 \| 0 \| 13 \| \| VCAN \| 0 \| 2.884954694 \| 0 \| 13 \| \| EMILIN1 \| 0 \| 2.88442197 \| 0 \| 13 \| \| FSTL1 \| 0 \| 2.879034767 \| 0 \| 13 \| \| CYR61 \| 0 \| 2.86897344 \| 0 \| 13 \| \| COL14A1 \| 0 \| 2.837637485 \| 0 \| 13 \| \| COL5A1 \| 0 \| 2.830987867 \| 0 \| 13 \| \| ASPN \| 0 \| 2.796898306 \| 0 \| 13 \| \| IFITM3 \| 0 \| 2.747676425 \| 0 \| 13 \| \| IGFBP6 \| 0 \| 2.744489513 \| 0 \| 13 \| \| GREM1 \| 0 \| 2.726493892 \| 0 \| 13 \| \| A2M \| 0 \| 2.724895107 \| 0 \| 13 \| \| SERPING1 \| 0 \| 2.723752394 \| 0 \| 13 \| \| TIMP2 \| 0 \| 2.717911433 \| 0 \| 13 \| \| PDGFRA \| 0 \| 2.695649278 \| 0 \| 13 \| \| TAGLN \| 0 \| 2.664825005 \| 0 \| 13 \| \| FBN1 \| 0 \| 2.644486153 \| 0 \| 13 \| \| DPT \| 0 \| 2.635777869 \| 0 \| 13 \| \| ADH1B \| 0 \| 2.534629439 \| 0 \| 13 \| \| HTRA1 \| 0 \| 2.51520918 \| 0 \| 13 \| \| PLAT \| 0 \| 2.502347913 \| 0 \| 13 \| \| MXRA5 \| 0 \| 2.494625184 \| 0 \| 13 \| \| CCL2 \| 0 \| 2.481213828 \| 0 \| 13 \| \| THBS2 \| 0 \| 2.446532661 \| 0 \| 13 \| \| SPARCL1 \| 0 \| 2.382359202 \| 0 \| 13 \| \| F3 \| 0 \| 2.379142376 \| 0 \| 13 \| \| MXRA8 \| 0 \| 2.341510212 \| 0 \| 13 \| \| CDH11 \| 0 \| 2.324345029 \| 0 \| 13 \| \| SELENOM \| 0 \| 2.304488188 \| 0 \| 13 \| \| MYL9 \| 0 \| 2.299962183 \| 0 \| 13 \| \| ANTXR1 \| 0 \| 2.266263212 \| 0 \| 13 \| \| COL18A1 \| 0 \| 2.247235669 \| 0 \| 13 \| \| CFH \| 0 \| 2.214670264 \| 0 \| 13 \| \| ACTA2 \| 0 \| 2.192733044 \| 0 \| 13 \| \| COL8A1 \| 0 \| 2.179294608 \| 0 \| 13 \| \| NBL1 \| 0 \| 2.179093468 \| 0 \| 13 \| \| PMP22 \| 0 \| 2.176877226 \| 0 \| 13 \| \| TCF4 \| 0 \| 2.141052047 \| 0 \| 13 \| \| LRP1 \| 0 \| 2.111361675 \| 0 \| 13 \| \| PALLD \| 0 \| 2.106870924 \| 0 \| 13 \| \| GSN \| 0 \| 2.08805836 \| 0 \| 13 \| \| IGF1 \| 0 \| 2.087393208 \| 0 \| 13 \| \| LAMA4 \| 0 \| 2.054856457 \| 0 \| 13 \| \| MRC2 \| 0 \| 2.030823987 \| 0 \| 13 \| \| COL4A2 \| 0 \| 2.018799539 \| 0 \| 13 \| \| AKAP12 \| 0 \| 2.017338068 \| 0 \| 13 \| \| ISLR \| 0 \| 2.009817953 \| 0 \| 13 \| \| S100A13 \| 0 \| 1.987768589 \| 0 \| 13 \| \| MFGE8 \| 0 \| 1.987123824 \| 0 \| 13 \| \| PPIC \| 0 \| 1.986755071 \| 0 \| 13 \| \| PLAC9 \| 0 \| 1.986439659 \| 0 \| 13 \| \| SDC2 \| 0 \| 1.960122989 \| 0 \| 13 \| \| CLEC11A \| 0 \| 1.95180185 \| 0 \| 13 \| \| LAMB1 \| 0 \| 1.947262391 \| 0 \| 13 \| \| LAPTM4A \| 0 \| 1.934610389 \| 0 \| 13 \| \| CYGB \| 0 \| 1.906458835 \| 0 \| 13 \| \| TMEM176B \| 0 \| 1.904555773 \| 0 \| 13 \| \| EFEMP1 \| 0 \| 1.903990006 \| 0 \| 13 \| \| BICC1 \| 0 \| 1.896073768 \| 0 \| 13 \| \| NR2F1 \| 0 \| 1.894987702 \| 0 \| 13 \| \| LHFPL6 \| 0 \| 1.893093783 \| 0 \| 13 \| \| LTBP4 \| 0 \| 1.889491562 \| 0 \| 13 \| \| CD63 \| 0 \| 1.874332919 \| 0 \| 13 \| \| PDPN \| 0 \| 1.866704198 \| 0 \| 13 \| \| CNN3 \| 0 \| 1.839275429 \| 0 \| 13 \| \| CD81 \| 0 \| 1.816267571 \| 0 \| 13 \| \| GUCY1A1 \| 0 \| 1.805043894 \| 0 \| 13 \| \| NFIA \| 0 \| 1.802631618 \| 0 \| 13 \| \| NFIC \| 0 \| 1.795690369 \| 0 \| 13 \| \| CEBPD \| 0 \| 1.76962209 \| 0 \| 13 \| \| CLMP \| 0 \| 1.762155775 \| 0 \| 13 \| \| VIM \| 0 \| 1.755504304 \| 0 \| 13 \| \| NEXN \| 0 \| 1.755013173 \| 0 \| 13 \| \| DDR2 \| 0 \| 1.742898102 \| 0 \| 13 \| \| RARRES1 \| 0 \| 1.742089248 \| 0 \| 13 \| \| TSHZ2 \| 0 \| 1.739667948 \| 0 \| 13 \| \| TNC \| 0 \| 1.739381103 \| 0 \| 13 \| \| DKK3 \| 0 \| 1.725901917 \| 0 \| 13 \| \| OGN \| 0 \| 1.725748335 \| 0 \| 13 \| \| COL4A1 \| 0 \| 1.720264131 \| 0 \| 13 \| \| BEX3 \| 0 \| 1.714156452 \| 0 \| 13 \| \| TCEAL9 \| 0 \| 1.703095895 \| 0 \| 13 \| \| PLXDC2 \| 0 \| 1.6991629 \| 0 \| 13 \| \| CAVIN1 \| 0 \| 1.687641153 \| 0 \| 13 \| \| ALDH1A3 \| 0 \| 1.687551109 \| 0 \| 13 \| \| TFPI \| 0 \| 1.686514023 \| 0 \| 13 \| \| EDIL3 \| 0 \| 1.684177419 \| 0 \| 13 \| \| CAVIN3 \| 0 \| 1.682419848 \| 0 \| 13 \| \| ANXA5 \| 0 \| 1.678163407 \| 0 \| 13 \| \| TMEM158 \| 0 \| 1.677050978 \| 0 \| 13 \| \| CYBRD1 \| 0 \| 1.669964098 \| 0 \| 13 \| \| GGT5 \| 0 \| 1.668794407 \| 0 \| 13 \| \| FGF7 \| 0 \| 1.661057711 \| 0 \| 13 \| \| HTRA3 \| 0 \| 1.651628052 \| 0 \| 13 \| \| F2R \| 0 \| 1.648201192 \| 0 \| 13 \| \| NID1 \| 0 \| 1.64228882 \| 0 \| 13 \| \| PCDH18 \| 0 \| 1.634193667 \| 0 \| 13 \| \| EID1 \| 0 \| 1.62973261 \| 0 \| 13 \| \| FHL1 \| 0 \| 1.622794376 \| 0 \| 13 \| \| BMP4 \| 0 \| 1.62202278 \| 0 \| 13 \| \| LAMC1 \| 0 \| 1.620406308 \| 0 \| 13 \| \| FNDC1 \| 0 \| 1.618118026 \| 0 \| 13 \| \| GEM \| 0 \| 1.613893193 \| 0 \| 13 \| \| RBP1 \| 0 \| 1.611824061 \| 0 \| 13 \| \| TMEM176A \| 0 \| 1.611140458 \| 0 \| 13 \| \| TSPAN4 \| 0 \| 1.610486703 \| 0 \| 13 \| \| ANGPTL2 \| 0 \| 1.588993851 \| 0 \| 13 \| \| IL6ST \| 0 \| 1.587026918 \| 0 \| 13 \| \| PLPP1 \| 0 \| 1.578638841 \| 0 \| 13 \| \| ELN \| 0 \| 1.568045206 \| 0 \| 13 \| \| ITIH5 \| 0 \| 1.559724638 \| 0 \| 13 \| \| IGFBP3 \| 0 \| 1.55937108 \| 0 \| 13 \| \| PHLDA3 \| 0 \| 1.551415573 \| 0 \| 13 \| \| GNG11 \| 0 \| 1.547291079 \| 0 \| 13 \| \| PLTP \| 0 \| 1.539896089 \| 0 \| 13 \| \| GPC6 \| 0 \| 1.530366705 \| 0 \| 13 \| \| CAV1 \| 0 \| 1.530188692 \| 0 \| 13 \| \| PLAU \| 0 \| 1.528253661 \| 0 \| 13 \| \| LOXL2 \| 0 \| 1.528195762 \| 0 \| 13 \| \| ECM1 \| 0 \| 1.522174905 \| 0 \| 13 \| \| FKBP10 \| 0 \| 1.513318401 \| 0 \| 13 \| \| ITGB1 \| 0 \| 1.510095343 \| 0 \| 13 \| \| COL11A1 \| 0 \| 1.504747979 \| 0 \| 13 \| \| APOE \| 0 \| 1.500415218 \| 0 \| 13 \| \| NUPR1 \| 0 \| 1.493281157 \| 0 \| 13 \| \| FRMD6 \| 0 \| 1.481920707 \| 0 \| 13 \| \| PAPPA \| 0 \| 1.477613085 \| 0 \| 13 \| \| SOD3 \| 0 \| 1.468443985 \| 0 \| 13 \| \| RUNX1T1 \| 0 \| 1.466372924 \| 0 \| 13 \| \| PTCH1 \| 0 \| 1.459423889 \| 0 \| 13 \| \| KANK2 \| 0 \| 1.456995356 \| 0 \| 13 \| \| FRZB \| 0 \| 1.45386025 \| 0 \| 13 \| \| DIO2 \| 0 \| 1.452677424 \| 0 \| 13 \| \| FAP \| 0 \| 1.446234833 \| 0 \| 13 \| \| FOXF1 \| 0 \| 1.442034003 \| 0 \| 13 \| \| FERMT2 \| 0 \| 1.439451302 \| 0 \| 13 \| \| ADAMTS1 \| 0 \| 1.427058866 \| 0 \| 13 \| \| SERPINH1 \| 0 \| 1.410870507 \| 0 \| 13 \| \| NEGR1 \| 0 \| 1.410714062 \| 0 \| 13 \| \| AGT \| 0 \| 1.409936506 \| 0 \| 13 \| \| EDNRB \| 0 \| 1.406562417 \| 0 \| 13 \| \| COX7A1 \| 0 \| 1.403278001 \| 0 \| 13 \| \| THBS1 \| 0 \| 1.397178848 \| 0 \| 13 \| \| PTMS \| 0 \| 1.392632557 \| 0 \| 13 \| \| LSAMP \| 0 \| 1.387176204 \| 0 \| 13 \| \| NDN \| 0 \| 1.384439951 \| 0 \| 13 \| \| TGFB1I1 \| 0 \| 1.369163884 \| 0 \| 13 \| \| PPIB \| 0 \| 1.361356109 \| 0 \| 13 \| \| PITX1 \| 0 \| 1.357703368 \| 0 \| 13 \| \| SPON1 \| 0 \| 1.355021879 \| 0 \| 13 \| \| ZBTB20 \| 0 \| 1.350610283 \| 0 \| 13 \| \| COL15A1 \| 0 \| 1.350036068 \| 0 \| 13 \| \| VCAM1 \| 0 \| 1.34523568 \| 0 \| 13 \| \| WNT5A \| 0 \| 1.345098164 \| 0 \| 13 \| \| IL1R1 \| 0 \| 1.342554987 \| 0 \| 13 \| \| EVA1B \| 0 \| 1.341407985 \| 0 \| 13 \| \| PLOD2 \| 0 \| 1.341316032 \| 0 \| 13 \| \| MYLK \| 0 \| 1.337291428 \| 0 \| 13 \| \| WWTR1 \| 0 \| 1.336894361 \| 0 \| 13 \| \| NR2F2 \| 0 \| 1.331914553 \| 0 \| 13 \| \| RAB34 \| 0 \| 1.326156632 \| 0 \| 13 \| \| YBX3 \| 0 \| 1.322432934 \| 0 \| 13 \| \| 11-Sep \| 0 \| 1.314617215 \| 0 \| 13 \| \| RAB13 \| 0 \| 1.310139687 \| 0 \| 13 \| \| BNC2 \| 0 \| 1.30328265 \| 0 \| 13 \| \| RAB31 \| 0 \| 1.296232257 \| 0 \| 13 \| \| LTBP2 \| 0 \| 1.295792847 \| 0 \| 13 \| \| GLT8D2 \| 0 \| 1.285037397 \| 0 \| 13 \| \| ACTN1 \| 0 \| 1.283462029 \| 0 \| 13 \| \| FAM114A1 \| 0 \| 1.276357953 \| 0 \| 13 \| \| ITGB5 \| 0 \| 1.273561082 \| 0 \| 13 \| \| PODN \| 0 \| 1.267878748 \| 0 \| 13 \| \| ITGAV \| 0 \| 1.257127896 \| 0 \| 13 \| \| LTBP1 \| 0 \| 1.249576801 \| 0 \| 13 \| \| C9orf3 \| 0 \| 1.248388302 \| 0 \| 13 \| \| ABI3BP \| 0 \| 1.248205081 \| 0 \| 13 \| \| PXDN \| 0 \| 1.239352672 \| 0 \| 13 \| \| PDE5A \| 0 \| 1.238010332 \| 0 \| 13 \| \| MFAP4 \| 0 \| 1.234944639 \| 0 \| 13 \| \| EMID1 \| 0 \| 1.230454777 \| 0 \| 13 \| \| TCEAL4 \| 0 \| 1.229064463 \| 0 \| 13 \| \| CD248 \| 0 \| 1.223985588 \| 0 \| 13 \| \| PAM \| 0 \| 1.223732311 \| 0 \| 13 \| \| FBLN5 \| 0 \| 1.221102515 \| 0 \| 13 \| \| RBMS3 \| 0 \| 1.214462762 \| 0 \| 13 \| \| GPNMB \| 0 \| 1.212094256 \| 0 \| 13 \| \| SGCB \| 0 \| 1.211925971 \| 0 \| 13 \| \| SGCD \| 0 \| 1.207258034 \| 0 \| 13 \| \| EFEMP2 \| 0 \| 1.191826229 \| 0 \| 13 \| \| DPYSL3 \| 0 \| 1.184215239 \| 0 \| 13 \| \| TMEM119 \| 0 \| 1.182603823 \| 0 \| 13 \| \| SPRY1 \| 0 \| 1.18209795 \| 0 \| 13 \| \| COL16A1 \| 0 \| 1.174922089 \| 0 \| 13 \| \| VEGFB \| 0 \| 1.172534454 \| 0 \| 13 \| \| PRRX1 \| 0 \| 1.171050128 \| 0 \| 13 \| \| FBLN2 \| 0 \| 1.164834132 \| 0 \| 13 \| \| TUBA1A \| 0 \| 1.162143968 \| 0 \| 13 \| \| GJA1 \| 0 \| 1.156696461 \| 0 \| 13 \| \| CTSO \| 0 \| 1.156468644 \| 0 \| 13 \| \| FAM198B \| 0 \| 1.155976382 \| 0 \| 13 \| \| PCDH7 \| 0 \| 1.155575762 \| 0 \| 13 \| \| PTN \| 0 \| 1.155344318 \| 0 \| 13 \| \| ADAMTS2 \| 0 \| 1.155087316 \| 0 \| 13 \| \| FIBIN \| 0 \| 1.149039656 \| 0 \| 13 \| \| APP \| 0 \| 1.143639379 \| 0 \| 13 \| \| ZCCHC24 \| 0 \| 1.140104409 \| 0 \| 13 \| \| OAF \| 0 \| 1.138546873 \| 0 \| 13 \| \| ABCA8 \| 0 \| 1.135923146 \| 0 \| 13 \| \| HCFC1R1 \| 0 \| 1.134982651 \| 0 \| 13 \| \| CTSF \| 0 \| 1.130949537 \| 0 \| 13 \| \| LTBP3 \| 0 \| 1.130709682 \| 0 \| 13 \| \| FAM20C \| 0 \| 1.128675475 \| 0 \| 13 \| \| ZEB1 \| 0 \| 1.125890291 \| 0 \| 13 \| \| CRTAP \| 0 \| 1.124164728 \| 0 \| 13 \| \| LINC01082 \| 0 \| 1.119049286 \| 0 \| 13 \| \| NOVA1 \| 0 \| 1.11063895 \| 0 \| 13 \| \| CH25H \| 0 \| 1.106463432 \| 0 \| 13 \| \| RUNX1 \| 0 \| 1.098621332 \| 0 \| 13 \| \| LAMA2 \| 0 \| 1.093510997 \| 0 \| 13 \| \| SCPEP1 \| 0 \| 1.091434394 \| 0 \| 13 \| \| SNAI2 \| 0 \| 1.088127301 \| 0 \| 13 \| \| RTL8C \| 0 \| 1.078803943 \| 0 \| 13 \| \| COLEC12 \| 0 \| 1.076532603 \| 0 \| 13 \| \| AXL \| 0 \| 1.07480893 \| 0 \| 13 \| \| LOX \| 0 \| 1.069683817 \| 0 \| 13 \| \| FXYD1 \| 0 \| 1.067655082 \| 0 \| 13 \| \| LAMB2 \| 0 \| 1.067610734 \| 0 \| 13 \| \| COL7A1 \| 0 \| 1.065148193 \| 0 \| 13 \| \| PDLIM4 \| 0 \| 1.063620908 \| 0 \| 13 \| \| WNT2B \| 0 \| 1.061904584 \| 0 \| 13 \| \| ITGA11 \| 0 \| 1.05705805 \| 0 \| 13 \| \| SRPX \| 0 \| 1.055333853 \| 0 \| 13 \| \| BOC \| 0 \| 1.05419401 \| 0 \| 13 \| \| CRYAB \| 0 \| 1.043395539 \| 0 \| 13 \| \| TNFAIP6 \| 0 \| 1.040649152 \| 0 \| 13 \| \| GAS1 \| 0 \| 1.039806884 \| 0 \| 13 \| \| TWSG1 \| 0 \| 1.031551938 \| 0 \| 13 \| \| VCL \| 0 \| 1.030218575 \| 0 \| 13 \| \| FMOD \| 0 \| 1.029436594 \| 0 \| 13 \| \| FARP1 \| 0 \| 1.02858546 \| 0 \| 13 \| \| RRAS \| 0 \| 1.025169999 \| 0 \| 13 \| \| COPZ2 \| 0 \| 1.025104197 \| 0 \| 13 \| \| PDLIM7 \| 0 \| 1.019379343 \| 0 \| 13 \| \| LOXL1 \| 0 \| 1.018391542 \| 0 \| 13 \| \| PRKAR2B \| 0 \| 1.015372078 \| 0 \| 13 \| \| VASN \| 0 \| 1.013681245 \| 0 \| 13 \| \| SVIL \| 0 \| 1.010814579 \| 0 \| 13 \| \| RBMS1 \| 0 \| 1.010400067 \| 0 \| 13 \| \| SLIT3 \| 0 \| 1.008799981 \| 0 \| 13 \| \| PDGFRB \| 0 \| 1.0079782 \| 0 \| 13 \| \| SMARCA1 \| 0 \| 1.003414248 \| 0 \| 13 \| \| STEAP1 \| 0 \| 1.003319189 \| 0 \| 13 \| \| MAFB \| 0 \| 1.002360647 \| 0 \| 13 \| \| RND3 \| 2.6287E-307 \| 1.018475796 \| 5.7376E-303 \| 13 \| \| CYB5R3 \| 1.1853E-303 \| 1.015489408 \| 2.5872E-299 \| 13 \| \| FHL2 \| 1.288E-297 \| 1.158005724 \| 2.8113E-293 \| 13 \| \| DSTN \| 2.6265E-278 \| 1.197797368 \| 5.7329E-274 \| 13 \| \| TPM1 \| 1.8882E-272 \| 1.476259343 \| 4.1215E-268 \| 13 \| \| CD99 \| 1.9921E-268 \| 1.33201241 \| 4.3483E-264 \| 13 \| \| ID3 \| 5.2237E-260 \| 1.744188895 \| 1.1402E-255 \| 13 \| \| SULF2 \| 3.6475E-251 \| 1.007871072 \| 7.9613E-247 \| 13 \| \| ZFP36L1 \| 7.4182E-237 \| 1.169832973 \| 1.6192E-232 \| 13 \| \| NUCKS1 \| 4.2668E-231 \| 1.088462261 \| 9.3131E-227 \| 13 \| \| TSC22D1 \| 1.0508E-224 \| 1.107614698 \| 2.2936E-220 \| 13 \| \| CYBA \| 1.1003E-223 \| -1.559817829 \| 2.4016E-219 \| 13 \| \| TGFBI \| 2.1946E-217 \| 1.087780797 \| 4.7901E-213 \| 13 \| \| EZR \| 2.3218E-217 \| -2.123452198 \| 5.0677E-213 \| 13 \| \| SRGN \| 3.4039E-200 \| -3.340501003 \| 7.4296E-196 \| 13 \| \| MARCKS \| 1.6263E-185 \| 1.208927306 \| 3.5497E-181 \| 13 \| \| NFIB \| 4.0434E-180 \| 1.261955559 \| 8.8255E-176 \| 13 \| \| TPM4 \| 2.463E-176 \| 1.115770358 \| 5.3759E-172 \| 13 \| \| CEBPB \| 1.1543E-171 \| 1.285674618 \| 2.5195E-167 \| 13 \| \| BST2 \| 1.4553E-169 \| 1.157155974 \| 3.1766E-165 \| 13 \| \| VAMP8 \| 4.9851E-153 \| -1.68111009 \| 1.0881E-148 \| 13 \| \| EGR1 \| 1.0517E-151 \| 1.075976916 \| 2.2955E-147 \| 13 \| \| CXCL1 \| 8.1738E-147 \| 1.636044016 \| 1.7841E-142 \| 13 \| \| PTPRC \| 1.9448E-144 \| -2.551135876 \| 4.2449E-140 \| 13 \| \| RAB11FIP1 \| 1.986E-126 \| -1.770428349 \| 4.3348E-122 \| 13 \| \| IGKC \| 3.3694E-123 \| -4.85353397 \| 7.3544E-119 \| 13 \| \| RGS1 \| 5.1379E-120 \| -2.572763089 \| 1.1214E-115 \| 13 \| \| CYTIP \| 9.1887E-118 \| -1.784930547 \| 2.0056E-113 \| 13 \| \| ARHGDIB \| 7.4061E-112 \| -1.944732506 \| 1.6165E-107 \| 13 \| \| CD48 \| 2.1536E-110 \| -1.773911005 \| 4.7008E-106 \| 13 \| \| SLC38A1 \| 1.8322E-105 \| -1.310020299 \| 3.9991E-101 \| 13 \| \| CD52 \| 1.6317E-102 \| -2.384245025 \| 3.56151E-98 \| 13 \| \| LAPTM5 \| 6.85037E-96 \| -2.05763559 \| 1.49523E-91 \| 13 \| \| TMEM238 \| 5.28316E-95 \| -1.373894238 \| 1.15316E-90 \| 13 \| \| GADD45G \| 2.88856E-94 \| 1.010085674 \| 6.30486E-90 \| 13 \| \| TUBA4A \| 1.73116E-93 \| -1.283675558 \| 3.77861E-89 \| 13 \| \| ITGA6 \| 3.31537E-93 \| -1.339265414 \| 7.23645E-89 \| 13 \| \| CXCR4 \| 2.45692E-91 \| -2.768054471 \| 5.36273E-87 \| 13 \| \| DUSP2 \| 2.97658E-89 \| -2.053260142 \| 6.49697E-85 \| 13 \| \| RAC2 \| 6.68977E-89 \| -1.38906534 \| 1.46018E-84 \| 13 \| \| EPCAM \| 1.56065E-87 \| -2.223160986 \| 3.40643E-83 \| 13 \| \| CD53 \| 1.77872E-87 \| -1.467176619 \| 3.88241E-83 \| 13 \| \| ELF3 \| 1.24505E-86 \| -2.459587848 \| 2.71757E-82 \| 13 \| \| CLDN3 \| 5.91873E-86 \| -2.242194283 \| 1.29188E-81 \| 13 \| \| LCP1 \| 1.39713E-85 \| -1.797370971 \| 3.04953E-81 \| 13 \| \| CLDN4 \| 1.18411E-84 \| -2.373549914 \| 2.58456E-80 \| 13 \| \| AREG \| 1.33572E-84 \| -2.171898928 \| 2.91548E-80 \| 13 \| \| RGS16 \| 4.65693E-84 \| 1.069685363 \| 1.01647E-79 \| 13 \| \| STK17B \| 1.83573E-82 \| -1.649490016 \| 4.00686E-78 \| 13 \| \| TSPAN8 \| 1.5983E-80 \| -2.427854328 \| 3.4886E-76 \| 13 \| \| KRT19 \| 9.01222E-80 \| -2.91433762 \| 1.9671E-75 \| 13 \| \| ST14 \| 1.21528E-79 \| -1.284649895 \| 2.65259E-75 \| 13 \| \| SMIM22 \| 4.42118E-79 \| -1.713219286 \| 9.65012E-75 \| 13 \| \| FYB1 \| 4.72693E-78 \| -1.826880279 \| 1.03175E-73 \| 13 \| \| CD37 \| 1.04185E-76 \| -1.561140506 \| 2.27406E-72 \| 13 \| \| LGALS4 \| 6.50241E-76 \| -2.78238326 \| 1.41928E-71 \| 13 \| \| CD69 \| 5.9451E-75 \| -2.348961748 \| 1.29764E-70 \| 13 \| \| RBM47 \| 1.75331E-73 \| -1.042249673 \| 3.82695E-69 \| 13 \| \| GPR183 \| 2.80951E-73 \| -1.942708123 \| 6.13232E-69 \| 13 \| \| EVI2B \| 4.4961E-73 \| -1.089910324 \| 9.81364E-69 \| 13 \| \| PHGR1 \| 9.43062E-71 \| -3.732545549 \| 2.05842E-66 \| 13 \| \| KRT18 \| 1.61531E-70 \| -2.909341449 \| 3.52573E-66 \| 13 \| \| AGR2 \| 1.76001E-70 \| -2.645211462 \| 3.84157E-66 \| 13 \| \| FAM3D \| 1.72791E-69 \| -1.801744817 \| 3.77151E-65 \| 13 \| \| HCST \| 1.22306E-68 \| -1.564974961 \| 2.66957E-64 \| 13 \| \| CD74 \| 3.73999E-67 \| -2.565906643 \| 8.16327E-63 \| 13 \| \| OCIAD2 \| 1.19873E-66 \| -1.033976087 \| 2.61646E-62 \| 13 \| \| MUC13 \| 1.27333E-66 \| -1.654275716 \| 2.7793E-62 \| 13 \| \| SEC11C \| 1.53696E-66 \| -1.307567008 \| 3.35472E-62 \| 13 \| \| HMGA1 \| 5.35722E-65 \| -1.071837065 \| 1.16932E-60 \| 13 \| \| CD3D \| 1.98549E-64 \| -2.042784319 \| 4.33373E-60 \| 13 \| \| SAMSN1 \| 2.01164E-64 \| -1.484722345 \| 4.3908E-60 \| 13 \| \| BTG2 \| 6.11935E-64 \| -1.093160317 \| 1.33567E-59 \| 13 \| \| SLC44A4 \| 2.04644E-63 \| -1.336048193 \| 4.46677E-59 \| 13 \| \| SMAP2 \| 2.44205E-62 \| -1.457224956 \| 5.33027E-58 \| 13 \| \| KRT8 \| 6.36536E-62 \| -2.841152136 \| 1.38937E-57 \| 13 \| \| GPX2 \| 2.25618E-61 \| -1.668650263 \| 4.92457E-57 \| 13 \| \| PIGR \| 1.18231E-60 \| -3.960441415 \| 2.58062E-56 \| 13 \| \| TSPAN1 \| 1.30691E-60 \| -2.182609023 \| 2.85258E-56 \| 13 \| \| CD2 \| 4.62343E-60 \| -1.941927609 \| 1.00916E-55 \| 13 \| \| FXYD3 \| 1.00081E-59 \| -2.586585773 \| 2.18446E-55 \| 13 \| \| CYCS \| 2.05927E-59 \| -1.104450247 \| 4.49478E-55 \| 13 \| \| DSP \| 2.01024E-58 \| -1.422138616 \| 4.38776E-54 \| 13 \| \| MT2A \| 6.13879E-58 \| 1.235710688 \| 1.33991E-53 \| 13 \| \| TENT5C \| 6.54488E-58 \| -1.226475471 \| 1.42855E-53 \| 13 \| \| CRYBG1 \| 1.06783E-57 \| -1.063313774 \| 2.33076E-53 \| 13 \| \| BTG1 \| 1.21512E-56 \| -1.539505865 \| 2.65225E-52 \| 13 \| \| SYNE2 \| 1.53703E-56 \| -1.33362859 \| 3.35488E-52 \| 13 \| \| GMFG \| 3.60957E-56 \| -1.112070743 \| 7.87861E-52 \| 13 \| \| SH3BGRL3 \| 9.91529E-55 \| -1.127037057 \| 2.16421E-50 \| 13 \| \| CD24 \| 3.28755E-54 \| -1.700459148 \| 7.17573E-50 \| 13 \| \| TFF3 \| 3.77273E-51 \| -3.563631403 \| 8.23474E-47 \| 13 \| \| PDCD4 \| 5.52141E-51 \| -1.230641668 \| 1.20516E-46 \| 13 \| \| LCN2 \| 1.7547E-50 \| -2.433918554 \| 3.82999E-46 \| 13 \| \| S100A14 \| 2.73743E-50 \| -1.286747936 \| 5.97499E-46 \| 13 \| \| PMAIP1 \| 6.59642E-49 \| -1.143915855 \| 1.4398E-44 \| 13 \| \| LYZ \| 8.41066E-49 \| -3.131417132 \| 1.83579E-44 \| 13 \| \| ISG20 \| 6.77459E-47 \| -1.150001737 \| 1.47869E-42 \| 13 \| \| EFHD2 \| 1.13986E-39 \| -1.116440168 \| 2.48798E-35 \| 13 \| \| JPT1 \| 9.67673E-38 \| -1.036618904 \| 2.11214E-33 \| 13 \| \| CREM \| 1.92242E-37 \| -1.743924731 \| 4.19606E-33 \| 13 \| \| AC058791.1 \| 8.02042E-37 \| -1.225820284 \| 1.75062E-32 \| 13 \| \| REL \| 1.00198E-36 \| -1.573660448 \| 2.18702E-32 \| 13 \| \| RGCC \| 2.50845E-36 \| -1.419569695 \| 5.47519E-32 \| 13 \| \| HLA-DRA \| 2.74029E-36 \| -3.391465139 \| 5.98123E-32 \| 13 \| \| IGHA1 \| 8.75398E-36 \| -4.023728039 \| 1.91073E-31 \| 13 \| \| NR4A2 \| 9.26839E-36 \| -1.481891522 \| 2.02301E-31 \| 13 \| \| KLF5 \| 3.42701E-35 \| -1.240136691 \| 7.48012E-31 \| 13 \| \| JCHAIN \| 1.29443E-33 \| -3.433758461 \| 2.82535E-29 \| 13 \| \| TNFAIP3 \| 1.26906E-32 \| -1.814621493 \| 2.76998E-28 \| 13 \| \| IL7R \| 9.65393E-32 \| -2.352055479 \| 2.10716E-27 \| 13 \| \| FRYL \| 7.8244E-30 \| -1.016913065 \| 1.70783E-25 \| 13 \| \| IVNS1ABP \| 1.9074E-26 \| -1.010309412 \| 4.16327E-22 \| 13 \| \| ATP1B1 \| 4.99544E-26 \| -1.207669581 \| 1.09035E-21 \| 13 \| \| IGLC2 \| 3.72658E-23 \| -3.355823765 \| 8.13401E-19 \| 13 \| \| HSPD1 \| 2.28497E-22 \| -1.104174502 \| 4.9874E-18 \| 13 \| \| HERPUD1 \| 2.59164E-22 \| -1.098321701 \| 5.65677E-18 \| 13 \| \| ZNF331 \| 7.11187E-22 \| -1.538380735 \| 1.55231E-17 \| 13 \| \| FABP1 \| 2.82264E-21 \| -3.309257472 \| 6.16098E-17 \| 13 \| \| COTL1 \| 2.17409E-18 \| -1.263372504 \| 4.74539E-14 \| 13 \| \| YPEL5 \| 1.28997E-14 \| -1.009631156 \| 2.81563E-10 \| 13 \| \| HLA-DPA1 \| 1.93033E-13 \| -2.082629659 \| 4.21333E-09 \| 13 \| \| IGLC3 \| 2.92612E-10 \| -2.681278366 \| 6.38685E-06 \| 13 \| \| CKB \| 1.80139E-07 \| -1.837275448 \| 0.003931903 \| 13 \| \| TAGLN \| 0 \| 5.602225757 \| 0 \| 14 \| \| ACTA2 \| 0 \| 5.163283508 \| 0 \| 14 \| \| IGFBP7 \| 0 \| 4.636967747 \| 0 \| 14 \| \| MYL9 \| 0 \| 4.569193342 \| 0 \| 14 \| \| C11orf96 \| 0 \| 4.380244092 \| 0 \| 14 \| \| MYH11 \| 0 \| 4.291439471 \| 0 \| 14 \| \| TPM2 \| 0 \| 4.254629316 \| 0 \| 14 \| \| SPARCL1 \| 0 \| 4.180617815 \| 0 \| 14 \| \| ADIRF \| 0 \| 3.899660882 \| 0 \| 14 \| \| COL4A1 \| 0 \| 3.816382335 \| 0 \| 14 \| \| CALD1 \| 0 \| 3.769352015 \| 0 \| 14 \| \| CRYAB \| 0 \| 3.684014112 \| 0 \| 14 \| \| COL18A1 \| 0 \| 3.56588721 \| 0 \| 14 \| \| COL4A2 \| 0 \| 3.550145559 \| 0 \| 14 \| \| SPARC \| 0 \| 3.415380002 \| 0 \| 14 \| \| SOD3 \| 0 \| 3.338298544 \| 0 \| 14 \| \| NOTCH3 \| 0 \| 3.204721973 \| 0 \| 14 \| \| MCAM \| 0 \| 3.132802624 \| 0 \| 14 \| \| IGFBP5 \| 0 \| 3.110243964 \| 0 \| 14 \| \| MYLK \| 0 \| 3.070016148 \| 0 \| 14 \| \| CSRP2 \| 0 \| 3.069156451 \| 0 \| 14 \| \| ADAMTS1 \| 0 \| 3.059402955 \| 0 \| 14 \| \| A2M \| 0 \| 3.055503428 \| 0 \| 14 \| \| COL6A2 \| 0 \| 3.046060031 \| 0 \| 14 \| \| MGP \| 0 \| 3.021932604 \| 0 \| 14 \| \| BGN \| 0 \| 2.982288768 \| 0 \| 14 \| \| MAP1B \| 0 \| 2.974258547 \| 0 \| 14 \| \| IFITM3 \| 0 \| 2.845595892 \| 0 \| 14 \| \| SELENOM \| 0 \| 2.845318407 \| 0 \| 14 \| \| TIMP3 \| 0 \| 2.844834498 \| 0 \| 14 \| \| DES \| 0 \| 2.826444068 \| 0 \| 14 \| \| CAV1 \| 0 \| 2.819517336 \| 0 \| 14 \| \| COL3A1 \| 0 \| 2.790002057 \| 0 \| 14 \| \| NDUFA4L2 \| 0 \| 2.781826851 \| 0 \| 14 \| \| MFGE8 \| 0 \| 2.756261604 \| 0 \| 14 \| \| COL1A2 \| 0 \| 2.725937808 \| 0 \| 14 \| \| COL6A1 \| 0 \| 2.666640984 \| 0 \| 14 \| \| FN1 \| 0 \| 2.654656469 \| 0 \| 14 \| \| LHFPL6 \| 0 \| 2.653866241 \| 0 \| 14 \| \| COL1A1 \| 0 \| 2.627289905 \| 0 \| 14 \| \| THY1 \| 0 \| 2.588873361 \| 0 \| 14 \| \| C1R \| 0 \| 2.572456799 \| 0 \| 14 \| \| PDGFRB \| 0 \| 2.544696701 \| 0 \| 14 \| \| SERPING1 \| 0 \| 2.520887768 \| 0 \| 14 \| \| ACTG2 \| 0 \| 2.510082241 \| 0 \| 14 \| \| CAVIN3 \| 0 \| 2.506244777 \| 0 \| 14 \| \| IGFBP4 \| 0 \| 2.499778313 \| 0 \| 14 \| \| NNMT \| 0 \| 2.480868361 \| 0 \| 14 \| \| LGALS1 \| 0 \| 2.474376929 \| 0 \| 14 \| \| NR2F2 \| 0 \| 2.473714746 \| 0 \| 14 \| \| PLN \| 0 \| 2.460681419 \| 0 \| 14 \| \| COL14A1 \| 0 \| 2.40102497 \| 0 \| 14 \| \| C1S \| 0 \| 2.384648062 \| 0 \| 14 \| \| ADAMTS4 \| 0 \| 2.355764277 \| 0 \| 14 \| \| CPE \| 0 \| 2.338970451 \| 0 \| 14 \| \| COL5A2 \| 0 \| 2.335378784 \| 0 \| 14 \| \| CYR61 \| 0 \| 2.306286322 \| 0 \| 14 \| \| CAVIN1 \| 0 \| 2.240351052 \| 0 \| 14 \| \| COL6A3 \| 0 \| 2.210358469 \| 0 \| 14 \| \| LMOD1 \| 0 \| 2.162939764 \| 0 \| 14 \| \| FXYD1 \| 0 \| 2.148074993 \| 0 \| 14 \| \| PPP1R14A \| 0 \| 2.146698418 \| 0 \| 14 \| \| NEXN \| 0 \| 2.105095579 \| 0 \| 14 \| \| FRZB \| 0 \| 2.100797319 \| 0 \| 14 \| \| ITGA1 \| 0 \| 2.093571076 \| 0 \| 14 \| \| AEBP1 \| 0 \| 2.08702202 \| 0 \| 14 \| \| CNN3 \| 0 \| 2.086487862 \| 0 \| 14 \| \| TNS1 \| 0 \| 2.078793437 \| 0 \| 14 \| \| CCDC102B \| 0 \| 2.078518198 \| 0 \| 14 \| \| ID4 \| 0 \| 2.043411557 \| 0 \| 14 \| \| CRIP2 \| 0 \| 2.029731276 \| 0 \| 14 \| \| KANK2 \| 0 \| 2.015996403 \| 0 \| 14 \| \| EDNRA \| 0 \| 2.01135566 \| 0 \| 14 \| \| TFPI \| 0 \| 1.967920003 \| 0 \| 14 \| \| ASPN \| 0 \| 1.96616887 \| 0 \| 14 \| \| TGFB1I1 \| 0 \| 1.953003909 \| 0 \| 14 \| \| EPAS1 \| 0 \| 1.951744675 \| 0 \| 14 \| \| GJA4 \| 0 \| 1.944037782 \| 0 \| 14 \| \| PALLD \| 0 \| 1.940779647 \| 0 \| 14 \| \| PMP22 \| 0 \| 1.931666077 \| 0 \| 14 \| \| PLAC9 \| 0 \| 1.910117819 \| 0 \| 14 \| \| FSTL1 \| 0 \| 1.900005814 \| 0 \| 14 \| \| EBF1 \| 0 \| 1.880090248 \| 0 \| 14 \| \| PTP4A3 \| 0 \| 1.865690962 \| 0 \| 14 \| \| AKAP12 \| 0 \| 1.843808955 \| 0 \| 14 \| \| FHL1 \| 0 \| 1.842550228 \| 0 \| 14 \| \| LTBP1 \| 0 \| 1.800509419 \| 0 \| 14 \| \| CAV2 \| 0 \| 1.786020426 \| 0 \| 14 \| \| HES4 \| 0 \| 1.785959095 \| 0 \| 14 \| \| ANGPT2 \| 0 \| 1.777151196 \| 0 \| 14 \| \| SLIT3 \| 0 \| 1.77188765 \| 0 \| 14 \| \| FERMT2 \| 0 \| 1.753158321 \| 0 \| 14 \| \| LAMA4 \| 0 \| 1.752157408 \| 0 \| 14 \| \| LAMB2 \| 0 \| 1.752051347 \| 0 \| 14 \| \| NID1 \| 0 \| 1.735681556 \| 0 \| 14 \| \| DKK3 \| 0 \| 1.7262993 \| 0 \| 14 \| \| CNN1 \| 0 \| 1.716627887 \| 0 \| 14 \| \| CARMN \| 0 \| 1.705155633 \| 0 \| 14 \| \| SDC2 \| 0 \| 1.704782375 \| 0 \| 14 \| \| EHD2 \| 0 \| 1.701936105 \| 0 \| 14 \| \| RCAN2 \| 0 \| 1.701212264 \| 0 \| 14 \| \| PLXDC1 \| 0 \| 1.681859504 \| 0 \| 14 \| \| LAMC1 \| 0 \| 1.65797131 \| 0 \| 14 \| \| COX7A1 \| 0 \| 1.652363395 \| 0 \| 14 \| \| GUCY1A1 \| 0 \| 1.648595555 \| 0 \| 14 \| \| GUCY1B1 \| 0 \| 1.640059285 \| 0 \| 14 \| \| C1QTNF1 \| 0 \| 1.631384533 \| 0 \| 14 \| \| ABCC9 \| 0 \| 1.62020588 \| 0 \| 14 \| \| PDLIM3 \| 0 \| 1.591581209 \| 0 \| 14 \| \| TNC \| 0 \| 1.590872686 \| 0 \| 14 \| \| MXRA8 \| 0 \| 1.580847439 \| 0 \| 14 \| \| BCAM \| 0 \| 1.578712596 \| 0 \| 14 \| \| RBPMS \| 0 \| 1.577039151 \| 0 \| 14 \| \| PCOLCE \| 0 \| 1.575008735 \| 0 \| 14 \| \| DLC1 \| 0 \| 1.540178447 \| 0 \| 14 \| \| PLS3 \| 0 \| 1.524755467 \| 0 \| 14 \| \| RARRES2 \| 0 \| 1.523807888 \| 0 \| 14 \| \| KCNJ8 \| 0 \| 1.518210973 \| 0 \| 14 \| \| EDIL3 \| 0 \| 1.514670559 \| 0 \| 14 \| \| ARHGEF17 \| 0 \| 1.505121215 \| 0 \| 14 \| \| DDR2 \| 0 \| 1.497492707 \| 0 \| 14 \| \| COL5A1 \| 0 \| 1.493487661 \| 0 \| 14 \| \| CCDC3 \| 0 \| 1.483767425 \| 0 \| 14 \| \| CSPG4 \| 0 \| 1.476077055 \| 0 \| 14 \| \| SERPINI1 \| 0 \| 1.474068362 \| 0 \| 14 \| \| AXL \| 0 \| 1.467438733 \| 0 \| 14 \| \| ITGA7 \| 0 \| 1.461150799 \| 0 \| 14 \| \| SLC7A2 \| 0 \| 1.456996938 \| 0 \| 14 \| \| TPPP3 \| 0 \| 1.442813091 \| 0 \| 14 \| \| COL5A3 \| 0 \| 1.430514556 \| 0 \| 14 \| \| SNCG \| 0 \| 1.425648865 \| 0 \| 14 \| \| OLFML2A \| 0 \| 1.422878011 \| 0 \| 14 \| \| GGT5 \| 0 \| 1.421049671 \| 0 \| 14 \| \| ADGRF5 \| 0 \| 1.406008087 \| 0 \| 14 \| \| EMILIN1 \| 0 \| 1.403539333 \| 0 \| 14 \| \| TMEM47 \| 0 \| 1.392405779 \| 0 \| 14 \| \| COX4I2 \| 0 \| 1.390658079 \| 0 \| 14 \| \| CD248 \| 0 \| 1.388073899 \| 0 \| 14 \| \| PHLDA3 \| 0 \| 1.387212319 \| 0 \| 14 \| \| MMP11 \| 0 \| 1.382010184 \| 0 \| 14 \| \| STOM \| 0 \| 1.38037816 \| 0 \| 14 \| \| S1PR3 \| 0 \| 1.372525969 \| 0 \| 14 \| \| FILIP1 \| 0 \| 1.371615265 \| 0 \| 14 \| \| ITIH5 \| 0 \| 1.357824813 \| 0 \| 14 \| \| NRP1 \| 0 \| 1.356491248 \| 0 \| 14 \| \| SYNPO2 \| 0 \| 1.352624147 \| 0 \| 14 \| \| LOXL2 \| 0 \| 1.351368944 \| 0 \| 14 \| \| EFHD1 \| 0 \| 1.323026605 \| 0 \| 14 \| \| CDH6 \| 0 \| 1.31860133 \| 0 \| 14 \| \| PDE5A \| 0 \| 1.29286073 \| 0 \| 14 \| \| SORBS1 \| 0 \| 1.288877656 \| 0 \| 14 \| \| FAM20C \| 0 \| 1.280833869 \| 0 \| 14 \| \| COL12A1 \| 0 \| 1.278976326 \| 0 \| 14 \| \| COL15A1 \| 0 \| 1.276967521 \| 0 \| 14 \| \| ANTXR1 \| 0 \| 1.273590708 \| 0 \| 14 \| \| ADGRA2 \| 0 \| 1.271522896 \| 0 \| 14 \| \| MAP3K7CL \| 0 \| 1.260880059 \| 0 \| 14 \| \| GJC1 \| 0 \| 1.259775578 \| 0 \| 14 \| \| PGF \| 0 \| 1.259503128 \| 0 \| 14 \| \| PHLDB2 \| 0 \| 1.229878803 \| 0 \| 14 \| \| AOC3 \| 0 \| 1.229469564 \| 0 \| 14 \| \| ANGPTL1 \| 0 \| 1.229316675 \| 0 \| 14 \| \| NES \| 0 \| 1.228585322 \| 0 \| 14 \| \| HSPB6 \| 0 \| 1.22005825 \| 0 \| 14 \| \| SYNM \| 0 \| 1.210731597 \| 0 \| 14 \| \| ARHGAP29 \| 0 \| 1.19222584 \| 0 \| 14 \| \| PXDN \| 0 \| 1.176855191 \| 0 \| 14 \| \| RNF152 \| 0 \| 1.171054687 \| 0 \| 14 \| \| GNG11 \| 0 \| 1.155559398 \| 0 \| 14 \| \| GUCY1A2 \| 0 \| 1.145240382 \| 0 \| 14 \| \| VASN \| 0 \| 1.144542265 \| 0 \| 14 \| \| HEYL \| 0 \| 1.138549334 \| 0 \| 14 \| \| 4-Sep \| 0 \| 1.128822733 \| 0 \| 14 \| \| RASL12 \| 0 \| 1.123951598 \| 0 \| 14 \| \| ANGPT1 \| 0 \| 1.123101019 \| 0 \| 14 \| \| SULF1 \| 0 \| 1.119357895 \| 0 \| 14 \| \| ISYNA1 \| 0 \| 1.099900811 \| 0 \| 14 \| \| RBMS3 \| 0 \| 1.093650723 \| 0 \| 14 \| \| FAM13C \| 0 \| 1.091927734 \| 0 \| 14 \| \| KCNMB1 \| 0 \| 1.081427519 \| 0 \| 14 \| \| PRKG1 \| 0 \| 1.078195297 \| 0 \| 14 \| \| ARHGAP10 \| 0 \| 1.067665543 \| 0 \| 14 \| \| NDN \| 0 \| 1.052259082 \| 0 \| 14 \| \| PRELP \| 0 \| 1.044350005 \| 0 \| 14 \| \| PDE3A \| 0 \| 1.039071946 \| 0 \| 14 \| \| LRRC32 \| 0 \| 1.030243119 \| 0 \| 14 \| \| RAB34 \| 0 \| 1.020830768 \| 0 \| 14 \| \| WTIP \| 0 \| 1.014636666 \| 0 \| 14 \| \| PDLIM7 \| 2.1797E-304 \| 1.363129137 \| 4.7577E-300 \| 14 \| \| TIMP2 \| 2.2421E-302 \| 1.647411507 \| 4.8938E-298 \| 14 \| \| FLNA \| 4.5505E-300 \| 2.42080197 \| 9.9324E-296 \| 14 \| \| C9orf3 \| 2.4373E-298 \| 1.329270994 \| 5.32E-294 \| 14 \| \| NEURL1B \| 1.0607E-297 \| 1.084208381 \| 2.3152E-293 \| 14 \| \| CSRP1 \| 7.582E-296 \| 2.384226901 \| 1.6549E-291 \| 14 \| \| TCF4 \| 1.6564E-294 \| 1.503897803 \| 3.6155E-290 \| 14 \| \| CYGB \| 5.7423E-292 \| 1.210959065 \| 1.2534E-287 \| 14 \| \| GEM \| 5.0638E-290 \| 1.652290425 \| 1.1053E-285 \| 14 \| \| VCL \| 3.5127E-285 \| 1.698172444 \| 7.6672E-281 \| 14 \| \| PPP1R12B \| 1.9854E-279 \| 1.637395909 \| 4.3335E-275 \| 14 \| \| KLF9 \| 1.7715E-275 \| 1.733868832 \| 3.8666E-271 \| 14 \| \| VIM \| 7.6294E-265 \| 2.198674777 \| 1.6653E-260 \| 14 \| \| PDGFA \| 6.8059E-264 \| 1.686833573 \| 1.4855E-259 \| 14 \| \| TPM1 \| 3.6107E-262 \| 2.482356472 \| 7.8811E-258 \| 14 \| \| CYBRD1 \| 8.1553E-262 \| 1.015636484 \| 1.7801E-257 \| 14 \| \| NFIC \| 4.0849E-261 \| 1.618515257 \| 8.9161E-257 \| 14 \| \| INAFM1 \| 1.2515E-258 \| 1.178173245 \| 2.7316E-254 \| 14 \| \| CD81 \| 1.6939E-258 \| 1.890795809 \| 3.6973E-254 \| 14 \| \| BEX3 \| 1.0825E-255 \| 1.284573296 \| 2.3627E-251 \| 14 \| \| ENAH \| 4.2075E-252 \| 1.380857053 \| 9.1837E-248 \| 14 \| \| ADCY3 \| 7.0065E-247 \| 1.205770588 \| 1.5293E-242 \| 14 \| \| ZFHX3 \| 6.8941E-246 \| 1.697989265 \| 1.5048E-241 \| 14 \| \| CCL2 \| 3.4015E-245 \| 1.460341011 \| 7.4244E-241 \| 14 \| \| THBS1 \| 4.7215E-244 \| 1.870590171 \| 1.0306E-239 \| 14 \| \| ID3 \| 9.6577E-234 \| 2.437559714 \| 2.108E-229 \| 14 \| \| CTGF \| 3.3292E-231 \| 1.686807725 \| 7.2667E-227 \| 14 \| \| PTMS \| 4.4103E-231 \| 1.557171945 \| 9.6264E-227 \| 14 \| \| LPP \| 9.631E-231 \| 2.150468211 \| 2.1022E-226 \| 14 \| \| EVA1B \| 2.2759E-230 \| 1.266342797 \| 4.9675E-226 \| 14 \| \| GSN \| 2.7045E-224 \| 1.148724348 \| 5.9032E-220 \| 14 \| \| NUPR1 \| 2.9186E-224 \| 1.058689453 \| 6.3705E-220 \| 14 \| \| TINAGL1 \| 2.7731E-222 \| 1.734968881 \| 6.0529E-218 \| 14 \| \| CFH \| 1.3349E-220 \| 1.178205069 \| 2.9138E-216 \| 14 \| \| CLU \| 4.4394E-218 \| 2.967898231 \| 9.6899E-214 \| 14 \| \| TIMP1 \| 2.2798E-212 \| 2.183802617 \| 4.9761E-208 \| 14 \| \| ACTN1 \| 4.9739E-212 \| 1.787819367 \| 1.0856E-207 \| 14 \| \| DSTN \| 8.7804E-211 \| 2.819893622 \| 1.9165E-206 \| 14 \| \| RRAS \| 3.6099E-209 \| 1.273323462 \| 7.8794E-205 \| 14 \| \| PTK2 \| 2.1171E-207 \| 1.300796882 \| 4.621E-203 \| 14 \| \| CBX6 \| 2.6946E-205 \| 1.052202159 \| 5.8815E-201 \| 14 \| \| PDLIM4 \| 4.0405E-203 \| 1.248611545 \| 8.8192E-199 \| 14 \| \| FSTL3 \| 4.1707E-203 \| 1.1348414 \| 9.1034E-199 \| 14 \| \| SERPINF1 \| 1.8471E-202 \| 1.186879002 \| 4.0318E-198 \| 14 \| \| EID1 \| 1.6071E-201 \| 1.542344542 \| 3.5077E-197 \| 14 \| \| TUBA1A \| 6.7787E-196 \| 1.54248905 \| 1.4796E-191 \| 14 \| \| SMTN \| 1.0015E-195 \| 1.161736024 \| 2.186E-191 \| 14 \| \| HSPB1 \| 1.4232E-193 \| 1.702130107 \| 3.1065E-189 \| 14 \| \| LAPTM4A \| 1.8404E-193 \| 1.388755234 \| 4.017E-189 \| 14 \| \| EPS8 \| 1.8735E-188 \| 1.466265013 \| 4.0892E-184 \| 14 \| \| ITGB1 \| 3.7391E-188 \| 1.837031978 \| 8.1613E-184 \| 14 \| \| ITGAV \| 2.0151E-187 \| 1.24257552 \| 4.3983E-183 \| 14 \| \| TSC22D1 \| 2.9565E-186 \| 1.927769408 \| 6.4531E-182 \| 14 \| \| ATP2B4 \| 5.1411E-185 \| 1.080087611 \| 1.1222E-180 \| 14 \| \| MT2A \| 6.272E-182 \| 2.30442615 \| 1.369E-177 \| 14 \| \| TCEAL4 \| 1.5215E-181 \| 1.222040531 \| 3.3209E-177 \| 14 \| \| CEBPD \| 2.5219E-180 \| 2.042758532 \| 5.5045E-176 \| 14 \| \| SORBS2 \| 2.9209E-177 \| 1.424075826 \| 6.3755E-173 \| 14 \| \| RAB13 \| 3.047E-177 \| 1.353454611 \| 6.6507E-173 \| 14 \| \| MEF2C \| 9.3415E-175 \| 1.39765033 \| 2.039E-170 \| 14 \| \| MT1M \| 3.7973E-171 \| 1.610321485 \| 8.2883E-167 \| 14 \| \| HCFC1R1 \| 8.3443E-168 \| 1.28023567 \| 1.8213E-163 \| 14 \| \| CD63 \| 8.0714E-165 \| 1.248063703 \| 1.7618E-160 \| 14 \| \| RGS16 \| 5.0621E-158 \| 1.668020125 \| 1.1049E-153 \| 14 \| \| CYB5R3 \| 3.3349E-157 \| 1.258897659 \| 7.2792E-153 \| 14 \| \| OAZ2 \| 4.1836E-153 \| 1.074777036 \| 9.1316E-149 \| 14 \| \| SELENOW \| 2.1598E-152 \| 1.325694908 \| 4.7143E-148 \| 14 \| \| YBX3 \| 2.0056E-151 \| 1.508544968 \| 4.3777E-147 \| 14 \| \| NFIA \| 4.6772E-149 \| 1.055070072 \| 1.0209E-144 \| 14 \| \| 7-Sep \| 7.3485E-149 \| 1.39458845 \| 1.604E-144 \| 14 \| \| CYBA \| 7.9226E-147 \| -1.626325292 \| 1.7293E-142 \| 14 \| \| TCEAL9 \| 6.7904E-143 \| 1.115062435 \| 1.4821E-138 \| 14 \| \| MYL6 \| 1.63E-140 \| 1.407560265 \| 3.5577E-136 \| 14 \| \| PMEPA1 \| 1.4189E-139 \| 1.870178956 \| 3.097E-135 \| 14 \| \| CRTAP \| 3.8296E-139 \| 1.095735984 \| 8.3589E-135 \| 14 \| \| MYO1B \| 5.1312E-137 \| 1.452019367 \| 1.12E-132 \| 14 \| \| SH3BGRL \| 5.484E-136 \| 1.206320085 \| 1.197E-131 \| 14 \| \| EMP2 \| 1.4086E-129 \| 1.029799007 \| 3.0747E-125 \| 14 \| \| NFIB \| 1.9013E-129 \| 1.254440428 \| 4.1499E-125 \| 14 \| \| PPP1R12A \| 7.6557E-129 \| 1.269477217 \| 1.671E-124 \| 14 \| \| CRIM1 \| 2.5659E-123 \| 1.46883032 \| 5.6007E-119 \| 14 \| \| MGST3 \| 2.9719E-123 \| 1.201264509 \| 6.4868E-119 \| 14 \| \| FBXO32 \| 1.7292E-121 \| 1.635458663 \| 3.7744E-117 \| 14 \| \| UACA \| 4.2767E-121 \| 1.705731829 \| 9.3347E-117 \| 14 \| \| PTEN \| 1.7448E-119 \| 1.037534968 \| 3.8083E-115 \| 14 \| \| ZBTB20 \| 3.0925E-116 \| 1.225088023 \| 6.75E-112 \| 14 \| \| MAGED2 \| 9.6808E-113 \| 1.013658164 \| 2.113E-108 \| 14 \| \| MAPRE2 \| 5.9662E-112 \| 1.033083331 \| 1.3022E-107 \| 14 \| \| ANXA5 \| 1.2711E-110 \| 1.125573253 \| 2.7745E-106 \| 14 \| \| TPM4 \| 1.0455E-109 \| 1.267452857 \| 2.2819E-105 \| 14 \| \| SRGN \| 9.2034E-108 \| -3.051055819 \| 2.0088E-103 \| 14 \| \| SPON2 \| 1.6881E-105 \| 1.170035854 \| 3.6846E-101 \| 14 \| \| TLN1 \| 1.7483E-105 \| 1.110921197 \| 3.8161E-101 \| 14 \| \| EZR \| 2.1872E-103 \| -1.744631136 \| 4.7739E-99 \| 14 \| \| VAMP8 \| 2.0922E-100 \| -1.73229766 \| 4.56674E-96 \| 14 \| \| NUCKS1 \| 4.4975E-100 \| 1.030276591 \| 9.81674E-96 \| 14 \| \| MT1E \| 1.39397E-97 \| 1.118980942 \| 3.04263E-93 \| 14 \| \| LTBP4 \| 1.39262E-93 \| 1.140975057 \| 3.03968E-89 \| 14 \| \| PDK4 \| 3.9138E-93 \| 1.236328933 \| 8.54264E-89 \| 14 \| \| UGCG \| 8.92185E-89 \| 1.090599503 \| 1.94737E-84 \| 14 \| \| ZFP36L1 \| 1.26147E-88 \| 1.103054054 \| 2.75341E-84 \| 14 \| \| LAMB1 \| 1.78863E-85 \| 1.159130756 \| 3.90404E-81 \| 14 \| \| SH3BGRL3 \| 2.474E-85 \| -1.686160114 \| 5.40001E-81 \| 14 \| \| TNS3 \| 9.19679E-85 \| 1.004199826 \| 2.00738E-80 \| 14 \| \| PTPRC \| 7.46482E-84 \| -2.404124732 \| 1.62935E-79 \| 14 \| \| PHLDA1 \| 1.24411E-81 \| 1.287767829 \| 2.71553E-77 \| 14 \| \| RAB11FIP1 \| 7.61301E-77 \| -1.697147981 \| 1.66169E-72 \| 14 \| \| HES1 \| 3.502E-74 \| 1.044585401 \| 7.64381E-70 \| 14 \| \| GADD45B \| 1.25396E-72 \| 1.553285152 \| 2.73703E-68 \| 14 \| \| CYTIP \| 4.66895E-71 \| -1.710621027 \| 1.01909E-66 \| 14 \| \| CXCR4 \| 4.18381E-69 \| -2.879683739 \| 9.13201E-65 \| 14 \| \| RGS1 \| 1.23337E-68 \| -2.379026623 \| 2.69209E-64 \| 14 \| \| CD52 \| 4.4776E-63 \| -2.377796234 \| 9.77325E-59 \| 14 \| \| CD48 \| 6.09492E-63 \| -1.679640861 \| 1.33034E-58 \| 14 \| \| STK17B \| 9.94203E-63 \| -1.609268666 \| 2.17005E-58 \| 14 \| \| MT1X \| 1.46052E-61 \| 1.731569939 \| 3.18788E-57 \| 14 \| \| RHOB \| 2.56139E-60 \| 1.176886196 \| 5.59075E-56 \| 14 \| \| TMEM238 \| 1.94206E-58 \| -1.339878254 \| 4.23894E-54 \| 14 \| \| LAPTM5 \| 4.09794E-57 \| -1.958974166 \| 8.94458E-53 \| 14 \| \| ELF3 \| 2.40995E-56 \| -2.383557115 \| 5.2602E-52 \| 14 \| \| ZFP36L2 \| 3.9066E-56 \| -1.1606686 \| 8.52695E-52 \| 14 \| \| RAC2 \| 5.9396E-56 \| -1.417307919 \| 1.29644E-51 \| 14 \| \| CLDN3 \| 1.71792E-55 \| -2.225355915 \| 3.7497E-51 \| 14 \| \| EPCAM \| 1.06641E-54 \| -2.217671033 \| 2.32764E-50 \| 14 \| \| AREG \| 1.55763E-54 \| -2.426147633 \| 3.39985E-50 \| 14 \| \| CD74 \| 1.75954E-53 \| -1.710147838 \| 3.84055E-49 \| 14 \| \| CLDN4 \| 2.94788E-53 \| -2.325177169 \| 6.43434E-49 \| 14 \| \| FAM241A \| 1.02361E-52 \| 1.225899686 \| 2.23424E-48 \| 14 \| \| CD53 \| 1.87301E-52 \| -1.394098835 \| 4.08821E-48 \| 14 \| \| LCP1 \| 2.3398E-51 \| -1.706148006 \| 5.10708E-47 \| 14 \| \| TSPAN8 \| 5.05836E-51 \| -2.429080541 \| 1.10409E-46 \| 14 \| \| RBM47 \| 1.87429E-50 \| -1.0718195 \| 4.09102E-46 \| 14 \| \| KRT19 \| 4.81161E-50 \| -2.859569341 \| 1.05023E-45 \| 14 \| \| IGKC \| 1.37034E-49 \| -2.868160191 \| 2.99105E-45 \| 14 \| \| ST14 \| 3.95271E-49 \| -1.291086893 \| 8.62758E-45 \| 14 \| \| LGALS4 \| 5.55152E-49 \| -2.879813323 \| 1.21173E-44 \| 14 \| \| RPS29 \| 6.87976E-49 \| -1.016396776 \| 1.50164E-44 \| 14 \| \| DUSP2 \| 1.6402E-48 \| -1.732153001 \| 3.58007E-44 \| 14 \| \| LSP1 \| 8.47364E-48 \| -1.179291651 \| 1.84954E-43 \| 14 \| \| SMIM22 \| 1.44286E-47 \| -1.724416916 \| 3.14933E-43 \| 14 \| \| ISG20 \| 1.7539E-47 \| -1.213074249 \| 3.82823E-43 \| 14 \| \| PHGR1 \| 6.40895E-47 \| -4.38885618 \| 1.39888E-42 \| 14 \| \| CTSS \| 1.52774E-46 \| -1.223415444 \| 3.33459E-42 \| 14 \| \| FYB1 \| 6.45138E-46 \| -1.744367931 \| 1.40814E-41 \| 14 \| \| CD69 \| 7.50273E-46 \| -2.39771498 \| 1.63762E-41 \| 14 \| \| OCIAD2 \| 1.02877E-45 \| -1.021013638 \| 2.24549E-41 \| 14 \| \| CD37 \| 3.4002E-45 \| -1.469183158 \| 7.42162E-41 \| 14 \| \| AGR2 \| 7.84822E-45 \| -2.735994612 \| 1.71303E-40 \| 14 \| \| TPM3 \| 1.68008E-44 \| -1.060352631 \| 3.6671E-40 \| 14 \| \| HCST \| 3.48002E-44 \| -1.584963648 \| 7.59583E-40 \| 14 \| \| DSP \| 1.37679E-43 \| -1.453070767 \| 3.00511E-39 \| 14 \| \| TENT5C \| 1.82713E-43 \| -1.150578691 \| 3.98808E-39 \| 14 \| \| MUC13 \| 5.23626E-43 \| -1.666562057 \| 1.14292E-38 \| 14 \| \| CD24 \| 8.70805E-43 \| -1.80643689 \| 1.90071E-38 \| 14 \| \| EVI2B \| 1.16206E-42 \| -1.033507447 \| 2.53642E-38 \| 14 \| \| SLC44A4 \| 5.54469E-42 \| -1.34249122 \| 1.21024E-37 \| 14 \| \| SAMSN1 \| 1.87144E-41 \| -1.4703538 \| 4.08478E-37 \| 14 \| \| FAM3D \| 4.74217E-41 \| -1.758646109 \| 1.03507E-36 \| 14 \| \| IVNS1ABP \| 1.29626E-40 \| -1.080456056 \| 2.82935E-36 \| 14 \| \| PIGR \| 1.53332E-40 \| -4.076513762 \| 3.34678E-36 \| 14 \| \| CRYBG1 \| 2.58328E-40 \| -1.020540634 \| 5.63852E-36 \| 14 \| \| GPX2 \| 7.07832E-40 \| -1.649682565 \| 1.54499E-35 \| 14 \| \| TSPAN1 \| 8.9009E-39 \| -2.176335311 \| 1.9428E-34 \| 14 \| \| KLF5 \| 6.48322E-38 \| -1.359979703 \| 1.41509E-33 \| 14 \| \| JPT1 \| 1.49336E-37 \| -1.0539719 \| 3.25955E-33 \| 14 \| \| CD2 \| 2.29684E-37 \| -1.881070099 \| 5.01332E-33 \| 14 \| \| GPR183 \| 2.85543E-37 \| -1.834441822 \| 6.23254E-33 \| 14 \| \| REL \| 3.98058E-37 \| -1.570728773 \| 8.6884E-33 \| 14 \| \| CD3D \| 6.73641E-37 \| -1.88700097 \| 1.47036E-32 \| 14 \| \| EFHD2 \| 7.16155E-37 \| -1.124721467 \| 1.56315E-32 \| 14 \| \| S100A4 \| 7.19822E-37 \| 1.027952656 \| 1.57116E-32 \| 14 \| \| LCN2 \| 1.65717E-36 \| -2.312462817 \| 3.6171E-32 \| 14 \| \| TNFAIP3 \| 2.71158E-36 \| -1.874164047 \| 5.91857E-32 \| 14 \| \| TRAC \| 2.01703E-35 \| -1.738128263 \| 4.40256E-31 \| 14 \| \| TFF3 \| 2.47186E-35 \| -3.538519549 \| 5.39532E-31 \| 14 \| \| S100A14 \| 2.937E-35 \| -1.289822856 \| 6.41059E-31 \| 14 \| \| SAT1 \| 5.86536E-35 \| -1.086593108 \| 1.28023E-30 \| 14 \| \| PDCD4 \| 1.0813E-34 \| -1.086418347 \| 2.36015E-30 \| 14 \| \| IL7R \| 1.18287E-34 \| -2.752951762 \| 2.58186E-30 \| 14 \| \| SSR4 \| 1.34914E-34 \| -1.33256729 \| 2.94477E-30 \| 14 \| \| ELF1 \| 2.42414E-34 \| -1.041450585 \| 5.29118E-30 \| 14 \| \| TNFSF10 \| 6.19379E-33 \| -1.007345311 \| 1.35192E-28 \| 14 \| \| ARL4C \| 2.77578E-32 \| -1.382186572 \| 6.05871E-28 \| 14 \| \| HERPUD1 \| 1.59459E-31 \| -1.237564659 \| 3.48052E-27 \| 14 \| \| PMAIP1 \| 2.81356E-31 \| -1.092607928 \| 6.14115E-27 \| 14 \| \| KRT8 \| 3.38559E-31 \| -2.610076196 \| 7.38972E-27 \| 14 \| \| CAPG \| 1.77564E-30 \| -1.003530645 \| 3.87569E-26 \| 14 \| \| AC058791.1 \| 6.60256E-30 \| -1.23074276 \| 1.44114E-25 \| 14 \| \| FXYD3 \| 9.19262E-30 \| -2.269183892 \| 2.00647E-25 \| 14 \| \| KRT18 \| 9.30952E-30 \| -2.301228896 \| 2.03199E-25 \| 14 \| \| LYZ \| 2.07306E-29 \| -3.072776292 \| 4.52487E-25 \| 14 \| \| COTL1 \| 2.65653E-29 \| -1.308444027 \| 5.7984E-25 \| 14 \| \| FKBP11 \| 7.44894E-29 \| -1.020105388 \| 1.62588E-24 \| 14 \| \| RGCC \| 3.37498E-28 \| -1.38276837 \| 7.36657E-24 \| 14 \| \| ATP1B1 \| 6.52739E-28 \| -1.234590926 \| 1.42473E-23 \| 14 \| \| EGR1 \| 6.78862E-28 \| 1.052160716 \| 1.48175E-23 \| 14 \| \| PRNP \| 2.27964E-24 \| 1.011604016 \| 4.97576E-20 \| 14 \| \| JCHAIN \| 4.91247E-24 \| -2.566611032 \| 1.07225E-19 \| 14 \| \| SMAP2 \| 3.03442E-21 \| -1.082494485 \| 6.62322E-17 \| 14 \| \| CREM \| 3.18405E-21 \| -1.558979384 \| 6.94982E-17 \| 14 \| \| FABP1 \| 2.05633E-20 \| -3.487378613 \| 4.48834E-16 \| 14 \| \| HLA-DPA1 \| 4.34726E-19 \| -1.710172137 \| 9.48877E-15 \| 14 \| \| IGHA1 \| 5.17109E-19 \| -2.929095528 \| 1.12869E-14 \| 14 \| \| IGLC2 \| 6.72368E-18 \| -3.345180408 \| 1.46758E-13 \| 14 \| \| HLA-DRA \| 7.99427E-17 \| -2.165613753 \| 1.74491E-12 \| 14 \| \| NR4A2 \| 5.25768E-16 \| -1.030411078 \| 1.14759E-11 \| 14 \| \| IGLC3 \| 6.65343E-15 \| -3.436361654 \| 1.45224E-10 \| 14 \| \| TMEM54 \| 7.86517E-14 \| -1.261120161 \| 1.71673E-09 \| 14 \| \| ZNF331 \| 1.71627E-11 \| -1.142081178 \| 3.7461E-07 \| 14 \| \| ITM2C \| 4.65219E-07 \| -1.037004021 \| 0.010154343 \| 14 \| \| VWF \| 0 \| 4.895786651 \| 0 \| 15 \| \| SPARCL1 \| 0 \| 4.527663627 \| 0 \| 15 \| \| COL4A1 \| 0 \| 4.272560276 \| 0 \| 15 \| \| HSPG2 \| 0 \| 4.260279671 \| 0 \| 15 \| \| PECAM1 \| 0 \| 4.179551657 \| 0 \| 15 \| \| IGFBP7 \| 0 \| 4.119465515 \| 0 \| 15 \| \| ACKR1 \| 0 \| 4.071010174 \| 0 \| 15 \| \| AQP1 \| 0 \| 3.909176259 \| 0 \| 15 \| \| CLDN5 \| 0 \| 3.887471332 \| 0 \| 15 \| \| A2M \| 0 \| 3.743676 \| 0 \| 15 \| \| EGFL7 \| 0 \| 3.721848434 \| 0 \| 15 \| \| GNG11 \| 0 \| 3.698512678 \| 0 \| 15 \| \| CAV1 \| 0 \| 3.682386142 \| 0 \| 15 \| \| RAMP2 \| 0 \| 3.641991843 \| 0 \| 15 \| \| IGFBP3 \| 0 \| 3.626019981 \| 0 \| 15 \| \| COL4A2 \| 0 \| 3.612336621 \| 0 \| 15 \| \| SPARC \| 0 \| 3.601349076 \| 0 \| 15 \| \| ENG \| 0 \| 3.552300839 \| 0 \| 15 \| \| COL15A1 \| 0 \| 3.530065119 \| 0 \| 15 \| \| CD93 \| 0 \| 3.498479132 \| 0 \| 15 \| \| TM4SF1 \| 0 \| 3.221562481 \| 0 \| 15 \| \| TCF4 \| 0 \| 3.219168042 \| 0 \| 15 \| \| CALCRL \| 0 \| 3.136167693 \| 0 \| 15 \| \| CLEC14A \| 0 \| 3.054182026 \| 0 \| 15 \| \| IGFBP4 \| 0 \| 3.048865352 \| 0 \| 15 \| \| PCDH17 \| 0 \| 3.019768051 \| 0 \| 15 \| \| CRIP2 \| 0 \| 2.987405071 \| 0 \| 15 \| \| CDH5 \| 0 \| 2.980627876 \| 0 \| 15 \| \| PODXL \| 0 \| 2.968094579 \| 0 \| 15 \| \| EPAS1 \| 0 \| 2.958259087 \| 0 \| 15 \| \| IFITM3 \| 0 \| 2.926881564 \| 0 \| 15 \| \| HYAL2 \| 0 \| 2.880601105 \| 0 \| 15 \| \| FABP4 \| 0 \| 2.855687628 \| 0 \| 15 \| \| CAVIN2 \| 0 \| 2.834422026 \| 0 \| 15 \| \| CAVIN1 \| 0 \| 2.829951812 \| 0 \| 15 \| \| SOX18 \| 0 \| 2.826276254 \| 0 \| 15 \| \| PLVAP \| 0 \| 2.818794356 \| 0 \| 15 \| \| VWA1 \| 0 \| 2.814508612 \| 0 \| 15 \| \| EMCN \| 0 \| 2.671019825 \| 0 \| 15 \| \| TGFBR2 \| 0 \| 2.66761072 \| 0 \| 15 \| \| SLC9A3R2 \| 0 \| 2.658589398 \| 0 \| 15 \| \| NFIB \| 0 \| 2.649768671 \| 0 \| 15 \| \| CTGF \| 0 \| 2.641675476 \| 0 \| 15 \| \| SPRY1 \| 0 \| 2.625134541 \| 0 \| 15 \| \| FLT1 \| 0 \| 2.620189202 \| 0 \| 15 \| \| STC1 \| 0 \| 2.604993834 \| 0 \| 15 \| \| ADGRF5 \| 0 \| 2.604930585 \| 0 \| 15 \| \| MGP \| 0 \| 2.584716432 \| 0 \| 15 \| \| PTPRB \| 0 \| 2.557679533 \| 0 \| 15 \| \| SLCO2A1 \| 0 \| 2.534198027 \| 0 \| 15 \| \| WWTR1 \| 0 \| 2.523101905 \| 0 \| 15 \| \| HEG1 \| 0 \| 2.521106468 \| 0 \| 15 \| \| MMRN2 \| 0 \| 2.473876257 \| 0 \| 15 \| \| TIMP3 \| 0 \| 2.459607696 \| 0 \| 15 \| \| RNASE1 \| 0 \| 2.447121505 \| 0 \| 15 \| \| SERPINE1 \| 0 \| 2.442318271 \| 0 \| 15 \| \| MCAM \| 0 \| 2.432038191 \| 0 \| 15 \| \| KDR \| 0 \| 2.428699353 \| 0 \| 15 \| \| ENPP2 \| 0 \| 2.422555346 \| 0 \| 15 \| \| STOM \| 0 \| 2.412861037 \| 0 \| 15 \| \| ADAMTS1 \| 0 \| 2.389546069 \| 0 \| 15 \| \| S1PR1 \| 0 \| 2.357383972 \| 0 \| 15 \| \| PALMD \| 0 \| 2.333979571 \| 0 \| 15 \| \| NRP1 \| 0 \| 2.299661385 \| 0 \| 15 \| \| HTRA1 \| 0 \| 2.297883104 \| 0 \| 15 \| \| ARHGAP29 \| 0 \| 2.284860422 \| 0 \| 15 \| \| PRCP \| 0 \| 2.266052505 \| 0 \| 15 \| \| CRIM1 \| 0 \| 2.189818757 \| 0 \| 15 \| \| THSD7A \| 0 \| 2.182985938 \| 0 \| 15 \| \| CXCL12 \| 0 \| 2.169160906 \| 0 \| 15 \| \| CYYR1 \| 0 \| 2.166861309 \| 0 \| 15 \| \| NUAK1 \| 0 \| 2.164893238 \| 0 \| 15 \| \| PXDN \| 0 \| 2.147855712 \| 0 \| 15 \| \| BMPR2 \| 0 \| 2.136973592 \| 0 \| 15 \| \| COL18A1 \| 0 \| 2.121886771 \| 0 \| 15 \| \| JAM2 \| 0 \| 2.113306931 \| 0 \| 15 \| \| PLPP1 \| 0 \| 2.088612515 \| 0 \| 15 \| \| LIFR \| 0 \| 2.0802667 \| 0 \| 15 \| \| POSTN \| 0 \| 2.069752041 \| 0 \| 15 \| \| TM4SF18 \| 0 \| 2.068807159 \| 0 \| 15 \| \| NNMT \| 0 \| 2.055125165 \| 0 \| 15 \| \| BCAM \| 0 \| 2.030882254 \| 0 \| 15 \| \| EMP1 \| 0 \| 2.01137226 \| 0 \| 15 \| \| ROBO4 \| 0 \| 2.00383085 \| 0 \| 15 \| \| FAM167B \| 0 \| 1.999594983 \| 0 \| 15 \| \| LDB2 \| 0 \| 1.983461497 \| 0 \| 15 \| \| CYR61 \| 0 \| 1.973627034 \| 0 \| 15 \| \| ELK3 \| 0 \| 1.972033633 \| 0 \| 15 \| \| FN1 \| 0 \| 1.963720167 \| 0 \| 15 \| \| TIE1 \| 0 \| 1.960451003 \| 0 \| 15 \| \| PREX2 \| 0 \| 1.96038584 \| 0 \| 15 \| \| LMO2 \| 0 \| 1.946178266 \| 0 \| 15 \| \| RAI14 \| 0 \| 1.937199829 \| 0 \| 15 \| \| ADAMTS9 \| 0 \| 1.933093199 \| 0 \| 15 \| \| DOCK9 \| 0 \| 1.92430042 \| 0 \| 15 \| \| GJA1 \| 0 \| 1.917583354 \| 0 \| 15 \| \| ADAM15 \| 0 \| 1.90912102 \| 0 \| 15 \| \| APLNR \| 0 \| 1.905175642 \| 0 \| 15 \| \| LAMA4 \| 0 \| 1.891126545 \| 0 \| 15 \| \| LAMB1 \| 0 \| 1.88918577 \| 0 \| 15 \| \| FAM198B \| 0 \| 1.883174164 \| 0 \| 15 \| \| FSTL1 \| 0 \| 1.875483426 \| 0 \| 15 \| \| ECE1 \| 0 \| 1.873520067 \| 0 \| 15 \| \| MMRN1 \| 0 \| 1.869759505 \| 0 \| 15 \| \| ITGA5 \| 0 \| 1.86589261 \| 0 \| 15 \| \| FILIP1 \| 0 \| 1.847996002 \| 0 \| 15 \| \| RAB13 \| 0 \| 1.847295931 \| 0 \| 15 \| \| VAMP5 \| 0 \| 1.845888877 \| 0 \| 15 \| \| IL3RA \| 0 \| 1.838477873 \| 0 \| 15 \| \| FZD4 \| 0 \| 1.838190821 \| 0 \| 15 \| \| TSPAN7 \| 0 \| 1.833054476 \| 0 \| 15 \| \| CAV2 \| 0 \| 1.830991584 \| 0 \| 15 \| \| THBD \| 0 \| 1.828339025 \| 0 \| 15 \| \| PTPRM \| 0 \| 1.827699388 \| 0 \| 15 \| \| TGM2 \| 0 \| 1.816887554 \| 0 \| 15 \| \| ICAM2 \| 0 \| 1.81033504 \| 0 \| 15 \| \| LAMC1 \| 0 \| 1.80156762 \| 0 \| 15 \| \| MPZL2 \| 0 \| 1.793665801 \| 0 \| 15 \| \| MAP1B \| 0 \| 1.787438904 \| 0 \| 15 \| \| PCAT19 \| 0 \| 1.78683688 \| 0 \| 15 \| \| KCTD12 \| 0 \| 1.776036518 \| 0 \| 15 \| \| SELP \| 0 \| 1.770971216 \| 0 \| 15 \| \| C1orf54 \| 0 \| 1.759766025 \| 0 \| 15 \| \| RBP7 \| 0 \| 1.758377201 \| 0 \| 15 \| \| MTUS1 \| 0 \| 1.757767094 \| 0 \| 15 \| \| PLXND1 \| 0 \| 1.732780404 \| 0 \| 15 \| \| TFPI \| 0 \| 1.723625979 \| 0 \| 15 \| \| SHANK3 \| 0 \| 1.714602196 \| 0 \| 15 \| \| INHBB \| 0 \| 1.705022152 \| 0 \| 15 \| \| EHD4 \| 0 \| 1.704204763 \| 0 \| 15 \| \| NES \| 0 \| 1.698323535 \| 0 \| 15 \| \| ANGPT2 \| 0 \| 1.694495738 \| 0 \| 15 \| \| CFH \| 0 \| 1.692328411 \| 0 \| 15 \| \| LMCD1 \| 0 \| 1.685521832 \| 0 \| 15 \| \| SYNPO \| 0 \| 1.680607166 \| 0 \| 15 \| \| CAVIN3 \| 0 \| 1.665265042 \| 0 \| 15 \| \| CNN3 \| 0 \| 1.656089704 \| 0 \| 15 \| \| F2RL3 \| 0 \| 1.64374631 \| 0 \| 15 \| \| DLC1 \| 0 \| 1.639372536 \| 0 \| 15 \| \| CD34 \| 0 \| 1.629935438 \| 0 \| 15 \| \| ADGRL4 \| 0 \| 1.629649194 \| 0 \| 15 \| \| GIMAP7 \| 0 \| 1.628589017 \| 0 \| 15 \| \| LIMS2 \| 0 \| 1.620146278 \| 0 \| 15 \| \| FBN1 \| 0 \| 1.615377278 \| 0 \| 15 \| \| IL33 \| 0 \| 1.615189652 \| 0 \| 15 \| \| SASH1 \| 0 \| 1.602584599 \| 0 \| 15 \| \| RASIP1 \| 0 \| 1.591091376 \| 0 \| 15 \| \| MCF2L \| 0 \| 1.5792159 \| 0 \| 15 \| \| PDGFB \| 0 \| 1.569595861 \| 0 \| 15 \| \| TGFBR3 \| 0 \| 1.55580063 \| 0 \| 15 \| \| TEK \| 0 \| 1.550024697 \| 0 \| 15 \| \| SWAP70 \| 0 \| 1.546536559 \| 0 \| 15 \| \| RDX \| 0 \| 1.533262966 \| 0 \| 15 \| \| ZNF385D \| 0 \| 1.532005749 \| 0 \| 15 \| \| ERG \| 0 \| 1.516199144 \| 0 \| 15 \| \| MKL2 \| 0 \| 1.513935695 \| 0 \| 15 \| \| SEMA3F \| 0 \| 1.510898459 \| 0 \| 15 \| \| KANK3 \| 0 \| 1.507916543 \| 0 \| 15 \| \| NID1 \| 0 \| 1.502067562 \| 0 \| 15 \| \| TMEM88 \| 0 \| 1.500186729 \| 0 \| 15 \| \| RAPGEF5 \| 0 \| 1.499561305 \| 0 \| 15 \| \| GIMAP4 \| 0 \| 1.496191968 \| 0 \| 15 \| \| CXorf36 \| 0 \| 1.49534699 \| 0 \| 15 \| \| ZEB1 \| 0 \| 1.479964338 \| 0 \| 15 \| \| TMEM204 \| 0 \| 1.47837213 \| 0 \| 15 \| \| TJP1 \| 0 \| 1.47064547 \| 0 \| 15 \| \| PLS3 \| 0 \| 1.463580514 \| 0 \| 15 \| \| TMTC1 \| 0 \| 1.460218412 \| 0 \| 15 \| \| CALD1 \| 0 \| 1.457157869 \| 0 \| 15 \| \| TSHZ2 \| 0 \| 1.449481683 \| 0 \| 15 \| \| TNS2 \| 0 \| 1.446136604 \| 0 \| 15 \| \| TSPAN4 \| 0 \| 1.415629188 \| 0 \| 15 \| \| FGD5 \| 0 \| 1.404848315 \| 0 \| 15 \| \| ADGRL2 \| 0 \| 1.402332836 \| 0 \| 15 \| \| PLAT \| 0 \| 1.400965565 \| 0 \| 15 \| \| ACKR3 \| 0 \| 1.399944489 \| 0 \| 15 \| \| NRN1 \| 0 \| 1.397361781 \| 0 \| 15 \| \| PIK3R3 \| 0 \| 1.390385897 \| 0 \| 15 \| \| JAG2 \| 0 \| 1.388771441 \| 0 \| 15 \| \| LRRC32 \| 0 \| 1.386633915 \| 0 \| 15 \| \| FSCN1 \| 0 \| 1.386121837 \| 0 \| 15 \| \| DPYSL2 \| 0 \| 1.367772702 \| 0 \| 15 \| \| LAMB2 \| 0 \| 1.366546061 \| 0 \| 15 \| \| SNCG \| 0 \| 1.355821607 \| 0 \| 15 \| \| ADCY4 \| 0 \| 1.351968555 \| 0 \| 15 \| \| F2R \| 0 \| 1.347832597 \| 0 \| 15 \| \| LAMA5 \| 0 \| 1.345441276 \| 0 \| 15 \| \| KIAA0355 \| 0 \| 1.341574601 \| 0 \| 15 \| \| PKP4 \| 0 \| 1.337925788 \| 0 \| 15 \| \| SOX17 \| 0 \| 1.330225491 \| 0 \| 15 \| \| GRB10 \| 0 \| 1.311478432 \| 0 \| 15 \| \| SEMA6B \| 0 \| 1.29853353 \| 0 \| 15 \| \| AFAP1L1 \| 0 \| 1.294594429 \| 0 \| 15 \| \| VEGFC \| 0 \| 1.292403167 \| 0 \| 15 \| \| CD109 \| 0 \| 1.29238113 \| 0 \| 15 \| \| JCAD \| 0 \| 1.286292685 \| 0 \| 15 \| \| ADAMTS4 \| 0 \| 1.284895697 \| 0 \| 15 \| \| RPS6KA2 \| 0 \| 1.274642499 \| 0 \| 15 \| \| TMEM255B \| 0 \| 1.274333871 \| 0 \| 15 \| \| PPM1F \| 0 \| 1.271196357 \| 0 \| 15 \| \| CSGALNACT1 \| 0 \| 1.266357691 \| 0 \| 15 \| \| PGF \| 0 \| 1.265822388 \| 0 \| 15 \| \| FKBP9 \| 0 \| 1.263839468 \| 0 \| 15 \| \| DPYSL3 \| 0 \| 1.257658402 \| 0 \| 15 \| \| MAST4 \| 0 \| 1.249196183 \| 0 \| 15 \| \| EHD2 \| 0 \| 1.237418882 \| 0 \| 15 \| \| DOCK4 \| 0 \| 1.235520392 \| 0 \| 15 \| \| FAM107A \| 0 \| 1.234219991 \| 0 \| 15 \| \| STAB1 \| 0 \| 1.229973585 \| 0 \| 15 \| \| HIP1 \| 0 \| 1.225282383 \| 0 \| 15 \| \| LHFPL6 \| 0 \| 1.219794227 \| 0 \| 15 \| \| MMP2 \| 0 \| 1.212055976 \| 0 \| 15 \| \| MAGI1 \| 0 \| 1.209754755 \| 0 \| 15 \| \| PROS1 \| 0 \| 1.202858102 \| 0 \| 15 \| \| SMAD1 \| 0 \| 1.20278106 \| 0 \| 15 \| \| TNFAIP1 \| 0 \| 1.192029402 \| 0 \| 15 \| \| FERMT2 \| 0 \| 1.19090827 \| 0 \| 15 \| \| RAPGEF3 \| 0 \| 1.187440303 \| 0 \| 15 \| \| ACVRL1 \| 0 \| 1.18552952 \| 0 \| 15 \| \| LIMCH1 \| 0 \| 1.185052646 \| 0 \| 15 \| \| HECW2 \| 0 \| 1.18301343 \| 0 \| 15 \| \| SPNS2 \| 0 \| 1.180501044 \| 0 \| 15 \| \| MYCT1 \| 0 \| 1.175449574 \| 0 \| 15 \| \| GALNT18 \| 0 \| 1.167187216 \| 0 \| 15 \| \| FZD6 \| 0 \| 1.167132045 \| 0 \| 15 \| \| SERPINH1 \| 0 \| 1.166897445 \| 0 \| 15 \| \| FAM241A \| 0 \| 1.164669314 \| 0 \| 15 \| \| PTPN14 \| 0 \| 1.161851146 \| 0 \| 15 \| \| PCDH12 \| 0 \| 1.158671454 \| 0 \| 15 \| \| HHEX \| 0 \| 1.151212589 \| 0 \| 15 \| \| FAM110D \| 0 \| 1.151164408 \| 0 \| 15 \| \| KLF7 \| 0 \| 1.151148157 \| 0 \| 15 \| \| EPHB4 \| 0 \| 1.148768253 \| 0 \| 15 \| \| GIMAP6 \| 0 \| 1.145826064 \| 0 \| 15 \| \| CX3CL1 \| 0 \| 1.14284754 \| 0 \| 15 \| \| CLIC2 \| 0 \| 1.136834339 \| 0 \| 15 \| \| PTPRG \| 0 \| 1.131958519 \| 0 \| 15 \| \| SCARF1 \| 0 \| 1.131231386 \| 0 \| 15 \| \| COX7A1 \| 0 \| 1.127613349 \| 0 \| 15 \| \| EVA1B \| 0 \| 1.1199206 \| 0 \| 15 \| \| TSPAN18 \| 0 \| 1.110800622 \| 0 \| 15 \| \| GFOD1 \| 0 \| 1.109649656 \| 0 \| 15 \| \| GRASP \| 0 \| 1.106720099 \| 0 \| 15 \| \| CPLX1 \| 0 \| 1.103089859 \| 0 \| 15 \| \| GIMAP8 \| 0 \| 1.102934216 \| 0 \| 15 \| \| ARHGEF15 \| 0 \| 1.097650258 \| 0 \| 15 \| \| MCTP1 \| 0 \| 1.094459584 \| 0 \| 15 \| \| SERPING1 \| 0 \| 1.088150992 \| 0 \| 15 \| \| CDH13 \| 0 \| 1.086890525 \| 0 \| 15 \| \| F8 \| 0 \| 1.069840751 \| 0 \| 15 \| \| DOCK6 \| 0 \| 1.068052983 \| 0 \| 15 \| \| ARHGAP31 \| 0 \| 1.05508741 \| 0 \| 15 \| \| ZNF521 \| 0 \| 1.053821954 \| 0 \| 15 \| \| EBF1 \| 0 \| 1.052836671 \| 0 \| 15 \| \| MATN2 \| 0 \| 1.052678437 \| 0 \| 15 \| \| EXOC3L2 \| 0 \| 1.052618459 \| 0 \| 15 \| \| PDE2A \| 0 \| 1.051268304 \| 0 \| 15 \| \| ARAP3 \| 0 \| 1.039924093 \| 0 \| 15 \| \| NDST1 \| 0 \| 1.038540987 \| 0 \| 15 \| \| SOX7 \| 0 \| 1.016430162 \| 0 \| 15 \| \| HSPA12B \| 0 \| 1.013366324 \| 0 \| 15 \| \| SHROOM4 \| 0 \| 1.013184697 \| 0 \| 15 \| \| ARHGAP23 \| 0 \| 1.011895654 \| 0 \| 15 \| \| COL6A2 \| 0 \| 1.009674985 \| 0 \| 15 \| \| TTC28 \| 0 \| 1.007773595 \| 0 \| 15 \| \| SH2D3C \| 0 \| 1.006521078 \| 0 \| 15 \| \| LINC01235 \| 0 \| 1.006021636 \| 0 \| 15 \| \| ADAMTS6 \| 4.06E-303 \| 1.180806282 \| 8.8617E-299 \| 15 \| \| PPFIBP1 \| 2.9871E-300 \| 1.136675232 \| 6.5199E-296 \| 15 \| \| GNB4 \| 9.1633E-300 \| 1.01834116 \| 2.0001E-295 \| 15 \| \| UACA \| 9.2424E-298 \| 1.543299919 \| 2.0173E-293 \| 15 \| \| APP \| 6.5339E-297 \| 2.035361568 \| 1.4262E-292 \| 15 \| \| ARL15 \| 2.2195E-296 \| 1.172184521 \| 4.8445E-292 \| 15 \| \| RASAL2 \| 7.7173E-296 \| 1.169529194 \| 1.6844E-291 \| 15 \| \| SULF2 \| 8.4423E-294 \| 1.400976139 \| 1.8427E-289 \| 15 \| \| NEURL1B \| 2.2567E-290 \| 1.111424215 \| 4.9256E-286 \| 15 \| \| RGS3 \| 5.0403E-285 \| 1.205758078 \| 1.1002E-280 \| 15 \| \| SH3BP5 \| 1.4016E-284 \| 1.541433643 \| 3.0592E-280 \| 15 \| \| CCDC85B \| 2.7069E-280 \| 1.927518882 \| 5.9084E-276 \| 15 \| \| CNKSR3 \| 4.6051E-278 \| 1.331313699 \| 1.0051E-273 \| 15 \| \| HDAC7 \| 6.2325E-278 \| 1.155112433 \| 1.3604E-273 \| 15 \| \| NFIA \| 1.0224E-277 \| 1.26818617 \| 2.2316E-273 \| 15 \| \| CLIC4 \| 2.3976E-268 \| 1.048916438 \| 5.2332E-264 \| 15 \| \| NOSTRIN \| 2.5008E-268 \| 1.032531739 \| 5.4586E-264 \| 15 \| \| GBP4 \| 2.7808E-268 \| 1.179274885 \| 6.0697E-264 \| 15 \| \| MEF2C \| 4.7336E-265 \| 1.171416237 \| 1.0332E-260 \| 15 \| \| PDK4 \| 4.2829E-264 \| 1.462094009 \| 9.3483E-260 \| 15 \| \| VIM \| 4.758E-263 \| 2.073125662 \| 1.0385E-258 \| 15 \| \| MGST2 \| 2.567E-261 \| 1.481250578 \| 5.6029E-257 \| 15 \| \| IL6ST \| 2.0637E-259 \| 1.618297007 \| 4.5044E-255 \| 15 \| \| TCN2 \| 2.7072E-259 \| 1.007484265 \| 5.9089E-255 \| 15 \| \| RFLNB \| 2.796E-255 \| 1.132716653 \| 6.1028E-251 \| 15 \| \| 10-Sep \| 1.9525E-253 \| 1.051635516 \| 4.2617E-249 \| 15 \| \| NCKAP1 \| 1.1251E-251 \| 1.265422227 \| 2.4558E-247 \| 15 \| \| SPTBN1 \| 2.3151E-251 \| 1.795800528 \| 5.0532E-247 \| 15 \| \| PPIC \| 4.3975E-248 \| 1.117383624 \| 9.5984E-244 \| 15 \| \| ABL2 \| 7.8011E-246 \| 1.166133311 \| 1.7028E-241 \| 15 \| \| PDLIM1 \| 4.9534E-245 \| 1.882639902 \| 1.0812E-240 \| 15 \| \| INSR \| 5.5424E-245 \| 2.611674924 \| 1.2097E-240 \| 15 \| \| PEA15 \| 1.9279E-241 \| 1.144223824 \| 4.2081E-237 \| 15 \| \| PLXNA2 \| 2.9031E-237 \| 1.058277204 \| 6.3367E-233 \| 15 \| \| GNAI2 \| 3.8008E-233 \| 1.675394475 \| 8.2959E-229 \| 15 \| \| BEX3 \| 1.1033E-229 \| 1.066397247 \| 2.4081E-225 \| 15 \| \| LAPTM4B \| 7.9679E-229 \| 1.022035225 \| 1.7391E-224 \| 15 \| \| TRIOBP \| 2.3552E-228 \| 1.11549106 \| 5.1407E-224 \| 15 \| \| ITGA6 \| 4.3179E-227 \| 1.842170867 \| 9.4246E-223 \| 15 \| \| PTMS \| 1.5528E-226 \| 1.488099494 \| 3.3892E-222 \| 15 \| \| ASAP1 \| 1.7227E-224 \| 1.167601092 \| 3.7601E-220 \| 15 \| \| SEC14L1 \| 4.7532E-223 \| 1.243833756 \| 1.0375E-218 \| 15 \| \| S100A13 \| 5.3028E-223 \| 1.366977473 \| 1.1574E-218 \| 15 \| \| DUSP6 \| 8.6205E-223 \| 1.513453731 \| 1.8816E-218 \| 15 \| \| NR2F2 \| 1.1923E-222 \| 1.526469303 \| 2.6024E-218 \| 15 \| \| ATP2B4 \| 3.1166E-221 \| 1.021180234 \| 6.8027E-217 \| 15 \| \| S100A16 \| 5.153E-221 \| 1.225471553 \| 1.1247E-216 \| 15 \| \| QKI \| 6.8674E-220 \| 1.1174897 \| 1.4989E-215 \| 15 \| \| TINAGL1 \| 4.1055E-218 \| 1.237162243 \| 8.9611E-214 \| 15 \| \| TMEM173 \| 8.4332E-218 \| 1.056416754 \| 1.8407E-213 \| 15 \| \| MECOM \| 9.4681E-210 \| 1.062085383 \| 2.0666E-205 \| 15 \| \| FAM213A \| 6.4868E-209 \| 1.387843578 \| 1.4159E-204 \| 15 \| \| GSN \| 1.2372E-208 \| 1.65007747 \| 2.7004E-204 \| 15 \| \| NFIC \| 1.4519E-208 \| 1.071336578 \| 3.169E-204 \| 15 \| \| ITGB1 \| 3.5574E-208 \| 1.436339839 \| 7.7647E-204 \| 15 \| \| YES1 \| 4.2289E-208 \| 1.061610213 \| 9.2304E-204 \| 15 \| \| ITGA1 \| 6.8848E-208 \| 1.171558548 \| 1.5027E-203 \| 15 \| \| TBCD \| 1.4294E-207 \| 1.073345386 \| 3.12E-203 \| 15 \| \| CYB5R3 \| 3.0901E-206 \| 1.337279132 \| 6.7448E-202 \| 15 \| \| CDC42EP3 \| 2.2369E-205 \| 1.158524275 \| 4.8825E-201 \| 15 \| \| PTPN12 \| 3.9298E-205 \| 1.060485969 \| 8.5776E-201 \| 15 \| \| MAP4K4 \| 1.9308E-202 \| 1.120891213 \| 4.2144E-198 \| 15 \| \| CD81 \| 4.3971E-201 \| 1.663823647 \| 9.5975E-197 \| 15 \| \| NOTCH1 \| 3.6709E-199 \| 1.022106404 \| 8.0124E-195 \| 15 \| \| ID3 \| 4.9906E-199 \| 1.723861505 \| 1.0893E-194 \| 15 \| \| RBMS1 \| 5.7906E-198 \| 1.013182225 \| 1.2639E-193 \| 15 \| \| MSN \| 3.7445E-193 \| 1.489005119 \| 8.1731E-189 \| 15 \| \| TNFRSF1A \| 9.0356E-193 \| 1.084069776 \| 1.9722E-188 \| 15 \| \| EMP2 \| 1.8526E-192 \| 1.178112477 \| 4.0436E-188 \| 15 \| \| EDN1 \| 4.6881E-191 \| 2.06311494 \| 1.0233E-186 \| 15 \| \| CRYBG3 \| 8.4103E-187 \| 1.208147002 \| 1.8357E-182 \| 15 \| \| IFI27 \| 2.213E-184 \| 1.157212322 \| 4.8302E-180 \| 15 \| \| CLU \| 7.8031E-182 \| 1.959253347 \| 1.7032E-177 \| 15 \| \| IGFBP5 \| 7.9658E-179 \| 2.793511948 \| 1.7387E-174 \| 15 \| \| ACTN1 \| 6.4073E-177 \| 1.334491071 \| 1.3985E-172 \| 15 \| \| DUSP23 \| 9.3683E-175 \| 1.546105513 \| 2.0448E-170 \| 15 \| \| ARHGEF12 \| 3.757E-174 \| 1.030402025 \| 8.2005E-170 \| 15 \| \| KLF9 \| 6.2004E-174 \| 1.127065803 \| 1.3534E-169 \| 15 \| \| ITM2A \| 7.2158E-174 \| 1.034715686 \| 1.575E-169 \| 15 \| \| WARS \| 1.7409E-173 \| 1.273121075 \| 3.7998E-169 \| 15 \| \| CD320 \| 3.5193E-173 \| 2.040078284 \| 7.6816E-169 \| 15 \| \| SELENOW \| 2.3007E-172 \| 1.352989783 \| 5.0218E-168 \| 15 \| \| TCIM \| 5.7479E-172 \| 1.043268988 \| 1.2546E-167 \| 15 \| \| VGLL4 \| 4.1907E-171 \| 1.076985059 \| 9.147E-167 \| 15 \| \| MYO1B \| 6.4402E-171 \| 1.123979739 \| 1.4057E-166 \| 15 \| \| TNFSF10 \| 7.3089E-171 \| 1.30172471 \| 1.5953E-166 \| 15 \| \| CMIP \| 5.1003E-170 \| 1.197689895 \| 1.1132E-165 \| 15 \| \| TACC1 \| 1.8371E-169 \| 1.291797481 \| 4.0099E-165 \| 15 \| \| CYBA \| 7.5208E-167 \| -2.076822136 \| 1.6416E-162 \| 15 \| \| ZBTB20 \| 8.8018E-167 \| 1.32458969 \| 1.9212E-162 \| 15 \| \| ETS1 \| 1.2352E-166 \| 1.031743842 \| 2.6961E-162 \| 15 \| \| HIPK3 \| 1.5338E-165 \| 1.01331106 \| 3.3479E-161 \| 15 \| \| XAF1 \| 1.5352E-165 \| 1.078025544 \| 3.3509E-161 \| 15 \| \| ETS2 \| 4.274E-165 \| 1.2155686 \| 9.3289E-161 \| 15 \| \| UTRN \| 1.9954E-163 \| 1.340752467 \| 4.3554E-159 \| 15 \| \| NEDD9 \| 9.072E-161 \| 1.103460584 \| 1.9801E-156 \| 15 \| \| ITM2B \| 1.8005E-160 \| 1.484009183 \| 3.9299E-156 \| 15 \| \| NUCB1 \| 9.6246E-160 \| 1.111340414 \| 2.1008E-155 \| 15 \| \| RHOC \| 1.8075E-159 \| 1.159365257 \| 3.9452E-155 \| 15 \| \| MYH9 \| 1.0794E-158 \| 1.347292674 \| 2.3559E-154 \| 15 \| \| EID1 \| 6.4468E-155 \| 1.294774364 \| 1.4071E-150 \| 15 \| \| YBX3 \| 4.2887E-153 \| 1.350215659 \| 9.3609E-149 \| 15 \| \| FABP5 \| 4.3813E-149 \| 2.056805435 \| 9.563E-145 \| 15 \| \| CD99 \| 8.8363E-149 \| 1.335677092 \| 1.9287E-144 \| 15 \| \| IFI16 \| 1.3997E-148 \| 1.182360906 \| 3.0551E-144 \| 15 \| \| PTTG1IP \| 2.7623E-147 \| 1.097718528 \| 6.0292E-143 \| 15 \| \| MACF1 \| 1.5994E-145 \| 1.19443686 \| 3.491E-141 \| 15 \| \| ID1 \| 1.4424E-143 \| 1.719518521 \| 3.1484E-139 \| 15 \| \| BACE2 \| 2.0513E-143 \| 1.031489707 \| 4.4773E-139 \| 15 \| \| LAPTM4A \| 1.5846E-140 \| 1.14175939 \| 3.4587E-136 \| 15 \| \| KLF2 \| 1.1772E-138 \| 1.302109046 \| 2.5694E-134 \| 15 \| \| NCOA7 \| 6.5961E-137 \| 1.293269684 \| 1.4397E-132 \| 15 \| \| SRP14 \| 1.3799E-135 \| 1.36837005 \| 3.012E-131 \| 15 \| \| ADIRF \| 1.5083E-131 \| 1.539200441 \| 3.2922E-127 \| 15 \| \| ICAM1 \| 2.572E-130 \| 1.379854886 \| 5.6138E-126 \| 15 \| \| RAB11A \| 8.7987E-127 \| 1.033240392 \| 1.9205E-122 \| 15 \| \| C2CD4B \| 4.7329E-120 \| 1.033955846 \| 1.0331E-115 \| 15 \| \| TPM4 \| 7.07E-120 \| 1.16828763 \| 1.5432E-115 \| 15 \| \| HLA-E \| 2.435E-119 \| 1.038654916 \| 5.315E-115 \| 15 \| \| APLP2 \| 3.319E-119 \| 1.037453727 \| 7.2443E-115 \| 15 \| \| NDRG1 \| 1.8701E-118 \| 1.008847866 \| 4.0819E-114 \| 15 \| \| EFNB2 \| 2.1402E-117 \| 1.379624342 \| 4.6714E-113 \| 15 \| \| GAS6 \| 2.8446E-115 \| 1.004195179 \| 6.209E-111 \| 15 \| \| HIF1A \| 4.5104E-115 \| 1.050871181 \| 9.8449E-111 \| 15 \| \| RAC1 \| 1.0379E-113 \| 1.016600841 \| 2.2655E-109 \| 15 \| \| ANXA2 \| 8.4516E-112 \| 1.00777377 \| 1.8447E-107 \| 15 \| \| TAGLN2 \| 5.6423E-111 \| 1.037174622 \| 1.2316E-106 \| 15 \| \| BST2 \| 9.69569E-93 \| 1.138288709 \| 2.11628E-88 \| 15 \| \| RHOB \| 8.78323E-89 \| 1.218800021 \| 1.91712E-84 \| 15 \| \| UGCG \| 1.0734E-88 \| 1.018794843 \| 2.34292E-84 \| 15 \| \| LGALS1 \| 7.5931E-87 \| 1.113854716 \| 1.65735E-82 \| 15 \| \| ITM2C \| 1.22463E-78 \| -2.154495393 \| 2.673E-74 \| 15 \| \| PTPRC \| 5.04605E-76 \| -2.455373079 \| 1.1014E-71 \| 15 \| \| EZR \| 1.90567E-74 \| -1.677217515 \| 4.15951E-70 \| 15 \| \| ARRDC3 \| 1.94036E-72 \| 1.088115464 \| 4.23522E-68 \| 15 \| \| SOCS3 \| 4.44383E-72 \| 1.021822926 \| 9.69954E-68 \| 15 \| \| CD44 \| 2.34558E-69 \| -1.561829317 \| 5.1197E-65 \| 15 \| \| VAMP8 \| 5.36214E-67 \| -1.499322994 \| 1.17039E-62 \| 15 \| \| RGS1 \| 5.10469E-65 \| -2.498341267 \| 1.1142E-60 \| 15 \| \| JUND \| 3.77299E-63 \| -1.244559811 \| 8.23531E-59 \| 15 \| \| STK17B \| 3.16665E-62 \| -1.725732637 \| 6.91185E-58 \| 15 \| \| CYTIP \| 3.52511E-61 \| -1.71023646 \| 7.69426E-57 \| 15 \| \| RAB11FIP1 \| 6.84012E-61 \| -1.597576045 \| 1.49299E-56 \| 15 \| \| CD48 \| 8.84569E-56 \| -1.716501615 \| 1.93075E-51 \| 15 \| \| TMEM238 \| 1.03441E-53 \| -1.353028894 \| 2.2578E-49 \| 15 \| \| TENT5C \| 9.38094E-49 \| -1.389193622 \| 2.04758E-44 \| 15 \| \| CHCHD10 \| 9.90736E-48 \| -1.376268642 \| 2.16248E-43 \| 15 \| \| ELF3 \| 1.49783E-47 \| -2.376050624 \| 3.26931E-43 \| 15 \| \| CXCR4 \| 1.54112E-47 \| -2.826842572 \| 3.36381E-43 \| 15 \| \| CD53 \| 8.29218E-46 \| -1.425147261 \| 1.80994E-41 \| 15 \| \| CD52 \| 8.85988E-46 \| -2.296239575 \| 1.93385E-41 \| 15 \| \| RAC2 \| 1.18619E-45 \| -1.365028662 \| 2.58909E-41 \| 15 \| \| DUSP2 \| 2.35118E-45 \| -2.012671184 \| 5.13191E-41 \| 15 \| \| AREG \| 2.97288E-45 \| -2.455670931 \| 6.48891E-41 \| 15 \| \| CLDN3 \| 3.59906E-45 \| -2.087478103 \| 7.85567E-41 \| 15 \| \| CLDN4 \| 8.59147E-45 \| -2.276783701 \| 1.87526E-40 \| 15 \| \| LCP1 \| 2.99059E-44 \| -1.77366219 \| 6.52756E-40 \| 15 \| \| ATP1B1 \| 1.40307E-43 \| -1.69855473 \| 3.06249E-39 \| 15 \| \| LGALS4 \| 9.71364E-43 \| -2.769720556 \| 2.1202E-38 \| 15 \| \| ST14 \| 9.04108E-42 \| -1.247206815 \| 1.9734E-37 \| 15 \| \| EPCAM \| 7.10454E-41 \| -2.031903159 \| 1.55071E-36 \| 15 \| \| CD37 \| 7.9336E-41 \| -1.517059249 \| 1.73167E-36 \| 15 \| \| RBM47 \| 1.0493E-40 \| -1.013177745 \| 2.29031E-36 \| 15 \| \| FYB1 \| 1.88956E-40 \| -1.811852847 \| 4.12435E-36 \| 15 \| \| KRT8 \| 4.9439E-40 \| -2.852548704 \| 1.07911E-35 \| 15 \| \| GPR183 \| 1.05579E-39 \| -1.909426874 \| 2.30448E-35 \| 15 \| \| IRS2 \| 3.4006E-39 \| -1.202164751 \| 7.42249E-35 \| 15 \| \| TSPAN8 \| 6.00406E-39 \| -2.202108611 \| 1.31051E-34 \| 15 \| \| SMIM22 \| 9.14607E-39 \| -1.641566273 \| 1.99631E-34 \| 15 \| \| LSP1 \| 1.63526E-38 \| -1.218197618 \| 3.56928E-34 \| 15 \| \| CD69 \| 2.30935E-38 \| -2.374259566 \| 5.04062E-34 \| 15 \| \| ISG20 \| 4.47995E-38 \| -1.305233707 \| 9.77838E-34 \| 15 \| \| AGR2 \| 6.25097E-38 \| -2.5787492 \| 1.3644E-33 \| 15 \| \| HCST \| 2.70334E-37 \| -1.597107324 \| 5.90057E-33 \| 15 \| \| CD24 \| 3.59271E-37 \| -1.832158166 \| 7.84181E-33 \| 15 \| \| IGKC \| 5.14626E-37 \| -3.536181899 \| 1.12327E-32 \| 15 \| \| LGALS3BP \| 5.83705E-37 \| -1.354443174 \| 1.27405E-32 \| 15 \| \| PHGR1 \| 6.33205E-37 \| -4.311075239 \| 1.3821E-32 \| 15 \| \| ADGRE5 \| 9.62131E-37 \| -1.019431768 \| 2.10004E-32 \| 15 \| \| KLF5 \| 2.87783E-36 \| -1.352660192 \| 6.28145E-32 \| 15 \| \| FXYD3 \| 3.19165E-36 \| -2.578679137 \| 6.96641E-32 \| 15 \| \| KRT19 \| 4.60754E-36 \| -2.627868847 \| 1.00569E-31 \| 15 \| \| EVI2B \| 1.06158E-35 \| -1.027594156 \| 2.31711E-31 \| 15 \| \| BTG2 \| 1.21584E-35 \| -1.27234066 \| 2.65381E-31 \| 15 \| \| FTL \| 2.10721E-35 \| -1.369065977 \| 4.59942E-31 \| 15 \| \| CD3D \| 2.88047E-35 \| -2.020398329 \| 6.2872E-31 \| 15 \| \| S100A4 \| 5.1172E-35 \| -1.74633311 \| 1.11693E-30 \| 15 \| \| GLIPR1 \| 5.13818E-35 \| -1.226835708 \| 1.12151E-30 \| 15 \| \| CELF2 \| 5.45699E-35 \| -1.185470088 \| 1.1911E-30 \| 15 \| \| MUC13 \| 8.86852E-34 \| -1.579989326 \| 1.93573E-29 \| 15 \| \| SMAP2 \| 5.21056E-33 \| -1.439206959 \| 1.13731E-28 \| 15 \| \| SAMSN1 \| 1.30916E-32 \| -1.441582353 \| 2.85751E-28 \| 15 \| \| PIGR \| 9.17743E-32 \| -3.760649939 \| 2.00316E-27 \| 15 \| \| TRAC \| 4.09136E-31 \| -1.823654033 \| 8.93021E-27 \| 15 \| \| GPX2 \| 1.64522E-30 \| -1.606786982 \| 3.59103E-26 \| 15 \| \| IL7R \| 3.34134E-30 \| -2.75192827 \| 7.29315E-26 \| 15 \| \| VEGFA \| 1.01057E-29 \| -1.218558166 \| 2.20577E-25 \| 15 \| \| RGS2 \| 2.33341E-28 \| -1.499878924 \| 5.09312E-24 \| 15 \| \| FAM107B \| 5.78161E-28 \| -1.146590005 \| 1.26195E-23 \| 15 \| \| S100A14 \| 1.83776E-27 \| -1.272775801 \| 4.01128E-23 \| 15 \| \| CAMK2N1 \| 7.13058E-27 \| -1.19548329 \| 1.55639E-22 \| 15 \| \| RGS10 \| 3.17679E-26 \| -1.036030837 \| 6.93397E-22 \| 15 \| \| CEBPB \| 2.46482E-25 \| -1.202610792 \| 5.37996E-21 \| 15 \| \| CAPG \| 9.89249E-25 \| -1.022457842 \| 2.15923E-20 \| 15 \| \| CKB \| 1.55873E-24 \| -2.171763232 \| 3.40223E-20 \| 15 \| \| FAM3D \| 1.66868E-24 \| -1.62998782 \| 3.64222E-20 \| 15 \| \| HSP90AA1 \| 2.09676E-24 \| -1.067728397 \| 4.5766E-20 \| 15 \| \| TSPAN1 \| 3.63049E-24 \| -2.072962738 \| 7.92428E-20 \| 15 \| \| DNAJB1 \| 1.30308E-23 \| -1.36136541 \| 2.84423E-19 \| 15 \| \| TNFAIP3 \| 2.52345E-23 \| -1.913655552 \| 5.50793E-19 \| 15 \| \| KRT18 \| 4.18202E-23 \| -2.56913796 \| 9.12809E-19 \| 15 \| \| LYZ \| 5.52359E-23 \| -3.196639262 \| 1.20563E-18 \| 15 \| \| HERPUD1 \| 1.76469E-20 \| -1.218510517 \| 3.85178E-16 \| 15 \| \| LCN2 \| 2.14276E-20 \| -2.015185386 \| 4.67701E-16 \| 15 \| \| ZNF331 \| 3.99935E-20 \| -1.608123876 \| 8.72939E-16 \| 15 \| \| JCHAIN \| 8.96071E-20 \| -3.920058794 \| 1.95585E-15 \| 15 \| \| SLC44A4 \| 2.68753E-19 \| -1.154039329 \| 5.86607E-15 \| 15 \| \| AC058791.1 \| 1.47909E-17 \| -1.149898093 \| 3.2284E-13 \| 15 \| \| XBP1 \| 1.5141E-16 \| -1.163917128 \| 3.30482E-12 \| 15 \| \| IGHA1 \| 1.78616E-16 \| -3.757839316 \| 3.89864E-12 \| 15 \| \| FABP1 \| 2.37078E-16 \| -3.495116498 \| 5.1747E-12 \| 15 \| \| LAPTM5 \| 3.07622E-16 \| -1.50532009 \| 6.71446E-12 \| 15 \| \| PMAIP1 \| 8.60799E-16 \| -1.018415524 \| 1.87887E-11 \| 15 \| \| TFF3 \| 3.82171E-13 \| -3.327037 \| 8.34164E-09 \| 15 \| \| NR4A2 \| 4.34088E-13 \| -1.441464123 \| 9.47484E-09 \| 15 \| \| DSP \| 4.54826E-13 \| -1.114868332 \| 9.92748E-09 \| 15 \| \| SSR4 \| 4.85562E-12 \| -1.189038058 \| 1.05984E-07 \| 15 \| \| FTH1 \| 1.08207E-09 \| -1.142812462 \| 2.36184E-05 \| 15 \| \| IGLC2 \| 5.18913E-08 \| -5.917113939 \| 0.001132631 \| 15 \| \| IGLC3 \| 1.26058E-07 \| -3.102674127 \| 0.002751465 \| 15 \| \| BTG1 \| 2.93643E-07 \| -1.069896197 \| 0.006409344 \| 15 \| \| TMEM54 \| 2.19246E-06 \| -1.219840468 \| 0.047854925 \| 15 \| \| TPSAB1 \| 0 \| 7.024333912 \| 0 \| 16 \| \| CPA3 \| 0 \| 6.288975444 \| 0 \| 16 \| \| MS4A2 \| 0 \| 4.581877576 \| 0 \| 16 \| \| CTSG \| 0 \| 4.519191578 \| 0 \| 16 \| \| HPGDS \| 0 \| 4.285719343 \| 0 \| 16 \| \| LTC4S \| 0 \| 4.116930328 \| 0 \| 16 \| \| HPGD \| 0 \| 3.972610281 \| 0 \| 16 \| \| KIT \| 0 \| 3.913296613 \| 0 \| 16 \| \| CLU \| 0 \| 3.844784385 \| 0 \| 16 \| \| GATA2 \| 0 \| 3.762287853 \| 0 \| 16 \| \| SLC18A2 \| 0 \| 3.483369496 \| 0 \| 16 \| \| ADCYAP1 \| 0 \| 2.940751887 \| 0 \| 16 \| \| ALOX5AP \| 0 \| 2.892013914 \| 0 \| 16 \| \| HDC \| 0 \| 2.784718662 \| 0 \| 16 \| \| PTGS2 \| 0 \| 2.682828031 \| 0 \| 16 \| \| RHEX \| 0 \| 2.543704285 \| 0 \| 16 \| \| VWA5A \| 0 \| 2.42935167 \| 0 \| 16 \| \| RGS13 \| 0 \| 2.263964507 \| 0 \| 16 \| \| CSF1 \| 0 \| 2.152599206 \| 0 \| 16 \| \| MAOB \| 0 \| 2.106776317 \| 0 \| 16 \| \| PTGS1 \| 0 \| 1.964274456 \| 0 \| 16 \| \| TYROBP \| 0 \| 1.87594881 \| 0 \| 16 \| \| AKAP12 \| 0 \| 1.829439702 \| 0 \| 16 \| \| FCER1A \| 0 \| 1.823293757 \| 0 \| 16 \| \| SLC45A3 \| 0 \| 1.694441369 \| 0 \| 16 \| \| IL1RL1 \| 0 \| 1.679224144 \| 0 \| 16 \| \| KRT1 \| 0 \| 1.640576055 \| 0 \| 16 \| \| GCSAML \| 0 \| 1.639712246 \| 0 \| 16 \| \| CLNK \| 0 \| 1.547454084 \| 0 \| 16 \| \| MITF \| 0 \| 1.431870817 \| 0 \| 16 \| \| CAVIN2 \| 0 \| 1.281103957 \| 0 \| 16 \| \| ARMH1 \| 0 \| 1.13350382 \| 0 \| 16 \| \| ACSL4 \| 3.3673E-305 \| 1.841523906 \| 7.3498E-301 \| 16 \| \| ALOX5 \| 9.4738E-293 \| 1.833668607 \| 2.0678E-288 \| 16 \| \| ANXA1 \| 2.9213E-269 \| 2.54769362 \| 6.3763E-265 \| 16 \| \| GRAP2 \| 8.5633E-267 \| 1.192814727 \| 1.8691E-262 \| 16 \| \| VIM \| 2.1142E-254 \| 2.615594902 \| 4.6147E-250 \| 16 \| \| GLUL \| 4.5915E-249 \| 2.362118751 \| 1.0022E-244 \| 16 \| \| CAPG \| 2.1429E-248 \| 2.384737327 \| 4.6772E-244 \| 16 \| \| SAMSN1 \| 2.8775E-243 \| 2.189291623 \| 6.2807E-239 \| 16 \| \| BTK \| 1.3043E-238 \| 1.468508471 \| 2.8469E-234 \| 16 \| \| CD9 \| 1.9133E-237 \| 2.188163944 \| 4.1762E-233 \| 16 \| \| LYL1 \| 3.312E-235 \| 1.343967461 \| 7.229E-231 \| 16 \| \| TESPA1 \| 3.2006E-225 \| 1.093348868 \| 6.9859E-221 \| 16 \| \| SRGN \| 1.7365E-221 \| 2.049418329 \| 3.7902E-217 \| 16 \| \| GMPR \| 4.8695E-221 \| 1.103350372 \| 1.0629E-216 \| 16 \| \| DLC1 \| 5.4421E-220 \| 1.23517588 \| 1.1878E-215 \| 16 \| \| P2RX1 \| 4.0675E-219 \| 1.227777386 \| 8.8782E-215 \| 16 \| \| LAPTM4A \| 1.5656E-218 \| 2.231241919 \| 3.4173E-214 \| 16 \| \| CTTNBP2 \| 2.7464E-211 \| 1.277759936 \| 5.9945E-207 \| 16 \| \| STXBP6 \| 1.0536E-210 \| 1.573049383 \| 2.2997E-206 \| 16 \| \| CD69 \| 4.6589E-207 \| 2.203795644 \| 1.0169E-202 \| 16 \| \| C1orf162 \| 6.0521E-204 \| 1.455010679 \| 1.321E-199 \| 16 \| \| ARHGEF6 \| 1.5403E-185 \| 1.674804688 \| 3.362E-181 \| 16 \| \| SMYD3 \| 9.9647E-183 \| 1.409403855 \| 2.175E-178 \| 16 \| \| LIF \| 6.161E-178 \| 1.456126247 \| 1.3448E-173 \| 16 \| \| LAT2 \| 2.9436E-173 \| 1.335058636 \| 6.4249E-169 \| 16 \| \| CD63 \| 1.5694E-170 \| 1.732101291 \| 3.4256E-166 \| 16 \| \| LEO1 \| 3.5701E-169 \| 2.064220543 \| 7.7924E-165 \| 16 \| \| FTH1 \| 2.0713E-167 \| 1.856390359 \| 4.521E-163 \| 16 \| \| AC020571.1 \| 1.3179E-164 \| 1.326307672 \| 2.8766E-160 \| 16 \| \| BEX4 \| 6.5152E-160 \| 1.563408332 \| 1.4221E-155 \| 16 \| \| ALDH1A1 \| 1.3763E-158 \| 1.450553099 \| 3.0041E-154 \| 16 \| \| AREG \| 1.3935E-157 \| 3.241454976 \| 3.0417E-153 \| 16 \| \| BMP2K \| 2.1678E-147 \| 1.663507859 \| 4.7316E-143 \| 16 \| \| RAC2 \| 1.6061E-146 \| 1.76315299 \| 3.5056E-142 \| 16 \| \| S100A4 \| 4.0272E-146 \| 1.552158222 \| 8.7902E-142 \| 16 \| \| RAB32 \| 3.7477E-141 \| 1.662287446 \| 8.1801E-137 \| 16 \| \| H3F3B \| 6.2404E-140 \| 1.21407403 \| 1.3621E-135 \| 16 \| \| IL18R1 \| 5.645E-139 \| 1.315232493 \| 1.2321E-134 \| 16 \| \| RGS10 \| 1.1427E-138 \| 1.702683899 \| 2.4942E-134 \| 16 \| \| ARHGAP18 \| 6.6725E-135 \| 1.733324101 \| 1.4564E-130 \| 16 \| \| TMEM154 \| 9.5838E-135 \| 1.289532307 \| 2.0919E-130 \| 16 \| \| CSF2RB \| 1.0321E-132 \| 1.413418953 \| 2.2528E-128 \| 16 \| \| CD84 \| 1.8044E-132 \| 1.255885434 \| 3.9384E-128 \| 16 \| \| GPR65 \| 3.7877E-132 \| 1.722811803 \| 8.2675E-128 \| 16 \| \| NFKBIA \| 1.2565E-128 \| 1.665684015 \| 2.7427E-124 \| 16 \| \| PLIN2 \| 4.932E-128 \| 1.511307346 \| 1.0765E-123 \| 16 \| \| FCER1G \| 5.3651E-128 \| 1.105118535 \| 1.171E-123 \| 16 \| \| LAT \| 7.6685E-124 \| 1.120144487 \| 1.6738E-119 \| 16 \| \| FOSB \| 5.6924E-123 \| 1.640139281 \| 1.2425E-118 \| 16 \| \| CLIC1 \| 8.8456E-121 \| 1.342423715 \| 1.9307E-116 \| 16 \| \| ADRB2 \| 1.1569E-120 \| 1.28956817 \| 2.5252E-116 \| 16 \| \| NFKBIZ \| 8.1229E-120 \| 1.823936129 \| 1.773E-115 \| 16 \| \| BACE2 \| 5.8667E-115 \| 1.507394033 \| 1.2805E-110 \| 16 \| \| CD37 \| 4.7796E-114 \| 1.284676357 \| 1.0432E-109 \| 16 \| \| FXYD5 \| 7.0033E-113 \| 1.467431076 \| 1.5286E-108 \| 16 \| \| CD44 \| 6.8445E-112 \| 1.512116293 \| 1.4939E-107 \| 16 \| \| ARHGDIB \| 1.6931E-110 \| 1.257802677 \| 3.6956E-106 \| 16 \| \| CTNNBL1 \| 2.9646E-110 \| 1.407812446 \| 6.4708E-106 \| 16 \| \| NSMCE1 \| 1.1211E-109 \| 1.947901648 \| 2.4471E-105 \| 16 \| \| RNF130 \| 1.4413E-106 \| 1.378060032 \| 3.146E-102 \| 16 \| \| ACOT7 \| 4.2037E-100 \| 1.238623413 \| 9.17541E-96 \| 16 \| \| RGS1 \| 1.7147E-99 \| 1.402141031 \| 3.74273E-95 \| 16 \| \| LMO4 \| 7.5309E-99 \| 1.765025134 \| 1.64377E-94 \| 16 \| \| ATP6V0A2 \| 1.78978E-98 \| 1.209276624 \| 3.90655E-94 \| 16 \| \| UBB \| 4.42904E-97 \| 1.243619773 \| 9.66727E-93 \| 16 \| \| RPL36AL \| 3.49989E-94 \| 1.043310789 \| 7.63921E-90 \| 16 \| \| SELENOK \| 1.27404E-93 \| 1.529200798 \| 2.78084E-89 \| 16 \| \| RGS2 \| 4.26773E-90 \| 1.487469306 \| 9.31518E-86 \| 16 \| \| SDCBP \| 4.99827E-89 \| 1.486232805 \| 1.09097E-84 \| 16 \| \| CKLF \| 1.52456E-88 \| 1.333067395 \| 3.32766E-84 \| 16 \| \| LAPTM5 \| 2.62062E-87 \| 1.023214651 \| 5.72002E-83 \| 16 \| \| TNFSF10 \| 3.98036E-87 \| 1.385319058 \| 8.68794E-83 \| 16 \| \| CD82 \| 7.53143E-86 \| 1.35964374 \| 1.64389E-81 \| 16 \| \| ITM2A \| 3.37204E-82 \| 1.113324611 \| 7.36014E-78 \| 16 \| \| ATP6V1F \| 5.21749E-80 \| 1.346311635 \| 1.13882E-75 \| 16 \| \| PPP1R15A \| 2.9078E-79 \| 1.25611451 \| 6.34686E-75 \| 16 \| \| CD81 \| 7.96799E-79 \| 1.176221889 \| 1.73917E-74 \| 16 \| \| FOXP1 \| 1.00949E-78 \| 1.325126949 \| 2.20341E-74 \| 16 \| \| IL18 \| 1.35512E-78 \| 1.220171681 \| 2.95783E-74 \| 16 \| \| RHOBTB3 \| 2.46221E-76 \| 1.010272699 \| 5.37427E-72 \| 16 \| \| PRDX1 \| 2.56087E-76 \| 1.292102684 \| 5.58961E-72 \| 16 \| \| BST2 \| 4.64442E-76 \| 1.22113085 \| 1.01374E-71 \| 16 \| \| RASSF5 \| 2.3927E-75 \| 1.086711733 \| 5.22254E-71 \| 16 \| \| LPCAT2 \| 5.0023E-75 \| 1.15175178 \| 1.09185E-70 \| 16 \| \| RHOH \| 8.60219E-74 \| 1.110387914 \| 1.8776E-69 \| 16 \| \| SOCS1 \| 1.04448E-72 \| 1.427439663 \| 2.27978E-68 \| 16 \| \| SLC2A3 \| 7.60579E-72 \| 1.127418702 \| 1.66012E-67 \| 16 \| \| EMP3 \| 3.23659E-71 \| 1.186274232 \| 7.0645E-67 \| 16 \| \| ABCA1 \| 1.89803E-70 \| 1.213164566 \| 4.14284E-66 \| 16 \| \| LDHB \| 1.09946E-69 \| 1.15698181 \| 2.3998E-65 \| 16 \| \| AC020916.1 \| 9.07981E-69 \| 1.423587726 \| 1.98185E-64 \| 16 \| \| EGR1 \| 2.92755E-66 \| 1.62976727 \| 6.38996E-62 \| 16 \| \| TMEM176B \| 7.0814E-65 \| 1.339566222 \| 1.54566E-60 \| 16 \| \| SYNE2 \| 3.16963E-64 \| -1.700656151 \| 6.91835E-60 \| 16 \| \| ITM2B \| 7.99556E-64 \| 1.049240045 \| 1.74519E-59 \| 16 \| \| NR4A3 \| 9.15515E-64 \| 1.213223942 \| 1.9983E-59 \| 16 \| \| HINT1 \| 1.01142E-63 \| -1.075373988 \| 2.20763E-59 \| 16 \| \| SEC11A \| 3.7895E-63 \| 1.224355987 \| 8.27135E-59 \| 16 \| \| MYADM \| 8.09442E-63 \| 1.366934841 \| 1.76677E-58 \| 16 \| \| BHLHE40 \| 1.76921E-62 \| 1.093280344 \| 3.86165E-58 \| 16 \| \| MLPH \| 9.27754E-62 \| 1.001185955 \| 2.02501E-57 \| 16 \| \| MARCKS \| 2.38161E-61 \| -1.671645175 \| 5.19833E-57 \| 16 \| \| RPS4Y1 \| 1.51842E-60 \| 1.248522602 \| 3.31425E-56 \| 16 \| \| B4GALT5 \| 6.31425E-60 \| 1.137851213 \| 1.37821E-55 \| 16 \| \| 2-Sep \| 8.65795E-60 \| 1.273787532 \| 1.88977E-55 \| 16 \| \| ELL2 \| 1.44838E-59 \| 1.414513312 \| 3.16139E-55 \| 16 \| \| ISG20 \| 1.3561E-58 \| -1.446183631 \| 2.95995E-54 \| 16 \| \| DNAJA1 \| 5.23214E-58 \| 1.110601181 \| 1.14202E-53 \| 16 \| \| ASAH1 \| 3.99093E-56 \| 1.183254534 \| 8.711E-52 \| 16 \| \| NFE2L3 \| 9.65484E-56 \| 1.108735673 \| 2.10736E-51 \| 16 \| \| EZR \| 4.27532E-55 \| -1.322186835 \| 9.33173E-51 \| 16 \| \| OSBPL8 \| 2.00523E-53 \| 1.217611196 \| 4.37681E-49 \| 16 \| \| GADD45B \| 6.7899E-53 \| 1.377231373 \| 1.48203E-48 \| 16 \| \| ANXA2 \| 6.79688E-52 \| -1.565275765 \| 1.48356E-47 \| 16 \| \| NR4A1 \| 1.74544E-50 \| 1.124185851 \| 3.80977E-46 \| 16 \| \| GALNT6 \| 2.8445E-49 \| 1.261302614 \| 6.2087E-45 \| 16 \| \| TNFAIP8 \| 1.59899E-48 \| 1.016549393 \| 3.49013E-44 \| 16 \| \| HSPA1A \| 6.82323E-48 \| 1.461283594 \| 1.48931E-43 \| 16 \| \| IDS \| 2.48572E-47 \| 1.094370554 \| 5.42558E-43 \| 16 \| \| DUSP10 \| 5.78389E-47 \| 1.028893523 \| 1.26245E-42 \| 16 \| \| KRT18 \| 6.21766E-46 \| -2.627538825 \| 1.35713E-41 \| 16 \| \| PRNP \| 4.58503E-45 \| 1.112739462 \| 1.00077E-40 \| 16 \| \| RAB11FIP1 \| 1.10908E-44 \| -1.35775267 \| 2.42078E-40 \| 16 \| \| CYTIP \| 2.48909E-44 \| -1.539217616 \| 5.43293E-40 \| 16 \| \| ITGA6 \| 8.07175E-44 \| -1.267728992 \| 1.76182E-39 \| 16 \| \| PHGR1 \| 1.59213E-43 \| -2.907535804 \| 3.47514E-39 \| 16 \| \| TUBA1A \| 4.5425E-43 \| 1.690016791 \| 9.91491E-39 \| 16 \| \| SYAP1 \| 4.74396E-43 \| 1.436352492 \| 1.03546E-38 \| 16 \| \| EGR3 \| 2.30654E-42 \| 1.144347253 \| 5.03449E-38 \| 16 \| \| PRDM1 \| 3.42316E-42 \| -1.421390212 \| 7.47173E-38 \| 16 \| \| KRT8 \| 3.88581E-42 \| -2.572927499 \| 8.48157E-38 \| 16 \| \| CLDN4 \| 5.62412E-42 \| -2.145366898 \| 1.22758E-37 \| 16 \| \| KDM6B \| 6.58812E-42 \| 1.212682629 \| 1.43799E-37 \| 16 \| \| LGALS3BP \| 2.05605E-41 \| -1.402650748 \| 4.48773E-37 \| 16 \| \| ELF3 \| 2.41142E-41 \| -2.203545856 \| 5.26341E-37 \| 16 \| \| TUBA4A \| 2.86261E-41 \| -1.143254689 \| 6.24821E-37 \| 16 \| \| IFI27 \| 3.38285E-41 \| -2.630147445 \| 7.38374E-37 \| 16 \| \| APP \| 5.82942E-41 \| -1.290480956 \| 1.27239E-36 \| 16 \| \| ARID5B \| 5.84708E-41 \| -1.308369286 \| 1.27624E-36 \| 16 \| \| GOLGB1 \| 1.76286E-40 \| -1.074173671 \| 3.8478E-36 \| 16 \| \| FOSL2 \| 4.19286E-40 \| 1.030554426 \| 9.15176E-36 \| 16 \| \| TMEM238 \| 9.84259E-40 \| -1.209158161 \| 2.14834E-35 \| 16 \| \| DUSP6 \| 6.13843E-39 \| 1.283530964 \| 1.33983E-34 \| 16 \| \| EPCAM \| 4.64674E-38 \| -1.936900315 \| 1.01424E-33 \| 16 \| \| CLDN3 \| 4.79263E-38 \| -2.001971171 \| 1.04609E-33 \| 16 \| \| SKIL \| 9.41818E-38 \| 1.129700993 \| 2.05571E-33 \| 16 \| \| ALAS1 \| 1.90747E-37 \| 1.130714767 \| 4.16343E-33 \| 16 \| \| RPS6KA5 \| 2.7129E-37 \| 1.007004956 \| 5.92146E-33 \| 16 \| \| PDLIM1 \| 1.00856E-36 \| -1.110603255 \| 2.20138E-32 \| 16 \| \| TWISTNB \| 1.51786E-36 \| 1.14702161 \| 3.31303E-32 \| 16 \| \| CEMIP2 \| 2.24584E-36 \| -1.22200794 \| 4.90199E-32 \| 16 \| \| LMNA \| 1.67179E-35 \| 1.502627473 \| 3.64902E-31 \| 16 \| \| LGALS4 \| 2.63626E-35 \| -2.440243741 \| 5.75418E-31 \| 16 \| \| CEBPD \| 5.24481E-35 \| -1.46365122 \| 1.14479E-30 \| 16 \| \| RNF213 \| 2.39051E-34 \| -1.056961989 \| 5.21776E-30 \| 16 \| \| CPEB4 \| 1.09462E-33 \| 1.132325119 \| 2.38923E-29 \| 16 \| \| AC058791.1 \| 1.17091E-33 \| -1.320079585 \| 2.55574E-29 \| 16 \| \| LIMA1 \| 1.31436E-33 \| -1.119036562 \| 2.86884E-29 \| 16 \| \| CDC42EP5 \| 1.67472E-33 \| -1.375626495 \| 3.65542E-29 \| 16 \| \| CXCR4 \| 2.71957E-33 \| -2.174364722 \| 5.93601E-29 \| 16 \| \| TSPAN8 \| 3.0596E-33 \| -2.093745813 \| 6.6782E-29 \| 16 \| \| TSC22D2 \| 6.40176E-33 \| 1.125482805 \| 1.39731E-28 \| 16 \| \| TENT5C \| 8.56268E-33 \| -1.058723497 \| 1.86898E-28 \| 16 \| \| FKBP11 \| 1.04726E-32 \| -1.137643336 \| 2.28585E-28 \| 16 \| \| LSP1 \| 1.06229E-32 \| -1.139047995 \| 2.31866E-28 \| 16 \| \| MUC13 \| 6.41437E-32 \| -1.588221673 \| 1.40006E-27 \| 16 \| \| DSP \| 7.19749E-32 \| -1.421378237 \| 1.571E-27 \| 16 \| \| FXYD3 \| 8.11745E-32 \| -2.421022078 \| 1.7718E-27 \| 16 \| \| SMIM22 \| 9.93262E-32 \| -1.543574582 \| 2.16799E-27 \| 16 \| \| CD74 \| 2.45773E-31 \| -2.094296669 \| 5.3645E-27 \| 16 \| \| CD38 \| 7.54248E-31 \| 1.040916451 \| 1.6463E-26 \| 16 \| \| JCHAIN \| 1.70829E-30 \| -4.234648219 \| 3.72869E-26 \| 16 \| \| CD24 \| 5.66489E-30 \| -1.593642007 \| 1.23647E-25 \| 16 \| \| KLF5 \| 3.17289E-29 \| -1.320583341 \| 6.92546E-25 \| 16 \| \| SLC44A4 \| 3.95119E-29 \| -1.23841474 \| 8.62426E-25 \| 16 \| \| AGR2 \| 4.00505E-29 \| -2.0392416 \| 8.74182E-25 \| 16 \| \| PERP \| 4.85682E-29 \| -1.10371742 \| 1.0601E-24 \| 16 \| \| PIGR \| 5.47281E-29 \| -2.888176517 \| 1.19455E-24 \| 16 \| \| TMEM54 \| 5.72765E-29 \| -1.402490253 \| 1.25017E-24 \| 16 \| \| FAM3D \| 6.70118E-29 \| -1.556654662 \| 1.46267E-24 \| 16 \| \| CAMK2N1 \| 6.97229E-29 \| -1.193749652 \| 1.52184E-24 \| 16 \| \| ARL4C \| 7.73397E-29 \| -1.42131466 \| 1.68809E-24 \| 16 \| \| DDIT4 \| 2.30942E-28 \| 1.070263106 \| 5.04078E-24 \| 16 \| \| TRAC \| 3.39758E-28 \| -1.61679682 \| 7.4159E-24 \| 16 \| \| TFF3 \| 1.48022E-27 \| -2.738250674 \| 3.23088E-23 \| 16 \| \| GLIPR1 \| 3.29453E-27 \| -1.0967152 \| 7.19096E-23 \| 16 \| \| CD3D \| 4.96816E-27 \| -1.850376184 \| 1.0844E-22 \| 16 \| \| IGHA1 \| 1.1264E-26 \| -4.076942457 \| 2.4586E-22 \| 16 \| \| TSPAN1 \| 1.81646E-26 \| -1.936438947 \| 3.96479E-22 \| 16 \| \| S100A14 \| 3.6179E-26 \| -1.177691354 \| 7.89679E-22 \| 16 \| \| ID3 \| 1.15196E-25 \| -1.050051173 \| 2.51438E-21 \| 16 \| \| SLC12A2 \| 3.97389E-25 \| -1.257663368 \| 8.67381E-21 \| 16 \| \| FOS \| 7.25215E-25 \| 1.114946877 \| 1.58293E-20 \| 16 \| \| IGKC \| 2.21852E-24 \| -3.679442898 \| 4.84236E-20 \| 16 \| \| CKB \| 2.86523E-24 \| -2.052754406 \| 6.25395E-20 \| 16 \| \| GPX2 \| 8.124E-24 \| -1.332646542 \| 1.77323E-19 \| 16 \| \| IL7R \| 1.08021E-23 \| -2.488553582 \| 2.35778E-19 \| 16 \| \| HSPA1B \| 3.05029E-23 \| 1.15535 \| 6.65787E-19 \| 16 \| \| RGCC \| 2.48534E-22 \| -1.023467468 \| 5.42476E-18 \| 16 \| \| DNAJB1 \| 7.79546E-22 \| 1.006575754 \| 1.70151E-17 \| 16 \| \| ID1 \| 1.79226E-21 \| -1.162490643 \| 3.91197E-17 \| 16 \| \| LYZ \| 3.73453E-20 \| -2.701697005 \| 8.15135E-16 \| 16 \| \| LCN2 \| 1.12653E-19 \| -2.113041556 \| 2.45887E-15 \| 16 \| \| S100A10 \| 3.63195E-17 \| -1.141232938 \| 7.92746E-13 \| 16 \| \| FYB1 \| 1.30593E-15 \| -1.304982843 \| 2.85046E-11 \| 16 \| \| FABP1 \| 8.9311E-14 \| -2.597326539 \| 1.94939E-09 \| 16 \| \| HLA-DRA \| 8.54114E-13 \| -2.534060104 \| 1.86427E-08 \| 16 \| \| IGLC2 \| 2.84775E-12 \| -4.549662307 \| 6.21578E-08 \| 16 \| \| KRT19 \| 5.40298E-11 \| -1.861833156 \| 1.17931E-06 \| 16 \| \| IGLC3 \| 7.01464E-08 \| -4.938707251 \| 0.001531086 \| 16 \| \| TOP2A \| 0 \| 2.573542638 \| 0 \| 17 \| \| TYMS \| 0 \| 2.446493132 \| 0 \| 17 \| \| MKI67 \| 0 \| 2.384476674 \| 0 \| 17 \| \| NUSAP1 \| 0 \| 2.324317904 \| 0 \| 17 \| \| CENPF \| 0 \| 2.205777921 \| 0 \| 17 \| \| ASPM \| 0 \| 2.173922502 \| 0 \| 17 \| \| PTTG1 \| 0 \| 1.913430167 \| 0 \| 17 \| \| UBE2C \| 0 \| 1.84334495 \| 0 \| 17 \| \| TPX2 \| 0 \| 1.822040694 \| 0 \| 17 \| \| SMC2 \| 0 \| 1.787408179 \| 0 \| 17 \| \| CENPE \| 0 \| 1.751444576 \| 0 \| 17 \| \| BIRC5 \| 0 \| 1.69528565 \| 0 \| 17 \| \| HIST1H1B \| 0 \| 1.634037637 \| 0 \| 17 \| \| CLSPN \| 0 \| 1.524769955 \| 0 \| 17 \| \| CDK1 \| 0 \| 1.514175656 \| 0 \| 17 \| \| DLGAP5 \| 0 \| 1.49780299 \| 0 \| 17 \| \| TK1 \| 0 \| 1.479861043 \| 0 \| 17 \| \| MYBL2 \| 0 \| 1.460914526 \| 0 \| 17 \| \| MAD2L1 \| 0 \| 1.443870311 \| 0 \| 17 \| \| GTSE1 \| 0 \| 1.440659144 \| 0 \| 17 \| \| FAM111B \| 0 \| 1.424111883 \| 0 \| 17 \| \| KIF11 \| 0 \| 1.39084003 \| 0 \| 17 \| \| CENPM \| 0 \| 1.376987129 \| 0 \| 17 \| \| ZWINT \| 0 \| 1.335970523 \| 0 \| 17 \| \| STMN1 \| 0 \| 1.335210688 \| 0 \| 17 \| \| HMMR \| 0 \| 1.299353501 \| 0 \| 17 \| \| NUF2 \| 0 \| 1.268245802 \| 0 \| 17 \| \| NCAPG \| 0 \| 1.263074156 \| 0 \| 17 \| \| CCNB2 \| 0 \| 1.251986294 \| 0 \| 17 \| \| CCNA2 \| 0 \| 1.247598681 \| 0 \| 17 \| \| SGO2 \| 0 \| 1.24059578 \| 0 \| 17 \| \| CEP55 \| 0 \| 1.218237363 \| 0 \| 17 \| \| CENPU \| 0 \| 1.207266072 \| 0 \| 17 \| \| SHCBP1 \| 0 \| 1.152835372 \| 0 \| 17 \| \| UBE2T \| 0 \| 1.111696518 \| 0 \| 17 \| \| AURKB \| 0 \| 1.096601847 \| 0 \| 17 \| \| KIF23 \| 0 \| 1.095401945 \| 0 \| 17 \| \| NDC80 \| 0 \| 1.057847366 \| 0 \| 17 \| \| CDKN3 \| 0 \| 1.04141908 \| 0 \| 17 \| \| FBXO5 \| 1.4907E-301 \| 1.102400467 \| 3.2539E-297 \| 17 \| \| CENPK \| 1.6173E-296 \| 1.029573071 \| 3.5301E-292 \| 17 \| \| TACC3 \| 1.7655E-295 \| 1.095371045 \| 3.8535E-291 \| 17 \| \| ATAD2 \| 2.2332E-294 \| 1.464760699 \| 4.8745E-290 \| 17 \| \| KIF20B \| 1.1588E-276 \| 1.580983096 \| 2.5293E-272 \| 17 \| \| SMC4 \| 1.6363E-276 \| 2.032771676 \| 3.5715E-272 \| 17 \| \| HMGB2 \| 2.9819E-259 \| 3.178366603 \| 6.5086E-255 \| 17 \| \| LMNB1 \| 1.7433E-249 \| 1.172790243 \| 3.8052E-245 \| 17 \| \| CKS1B \| 9.6938E-249 \| 1.089867818 \| 2.1159E-244 \| 17 \| \| HELLS \| 7.7918E-243 \| 1.247406387 \| 1.7007E-238 \| 17 \| \| ATAD5 \| 2.5775E-226 \| 1.057769895 \| 5.6259E-222 \| 17 \| \| DTYMK \| 1.7168E-223 \| 1.139922076 \| 3.7472E-219 \| 17 \| \| SKA2 \| 2.3439E-217 \| 1.158382772 \| 5.116E-213 \| 17 \| \| ANP32E \| 1.4042E-213 \| 1.557506304 \| 3.065E-209 \| 17 \| \| HMGN2 \| 1.1954E-205 \| 1.060565208 \| 2.6092E-201 \| 17 \| \| EZH2 \| 3.4745E-202 \| 1.376505196 \| 7.5838E-198 \| 17 \| \| TMPO \| 8.0161E-200 \| 1.421531148 \| 1.7497E-195 \| 17 \| \| NUDT1 \| 8.3361E-197 \| 1.318488631 \| 1.8195E-192 \| 17 \| \| CORO1A \| 9.504E-183 \| 1.565364057 \| 2.0744E-178 \| 17 \| \| SNRNP25 \| 3.2127E-179 \| 1.132373487 \| 7.0124E-175 \| 17 \| \| TUBB \| 2.4612E-177 \| 2.48370172 \| 5.372E-173 \| 17 \| \| MCM5 \| 4.619E-172 \| 1.00988864 \| 1.0082E-167 \| 17 \| \| RRM1 \| 4.9117E-170 \| 1.041943505 \| 1.0721E-165 \| 17 \| \| PCNA \| 2.94E-167 \| 1.876790859 \| 6.4171E-163 \| 17 \| \| DNMT1 \| 6.3148E-164 \| 1.404296678 \| 1.3783E-159 \| 17 \| \| HIST1H1D \| 4.3482E-159 \| 1.525711774 \| 9.4908E-155 \| 17 \| \| DUT \| 1.955E-158 \| 2.038929741 \| 4.2672E-154 \| 17 \| \| H2AFZ \| 6.945E-154 \| 1.920826842 \| 1.5159E-149 \| 17 \| \| APOBEC3G \| 1.4844E-152 \| 1.070496058 \| 3.2399E-148 \| 17 \| \| RPA3 \| 8.3455E-152 \| 1.266685032 \| 1.8216E-147 \| 17 \| \| CD38 \| 2.3557E-150 \| 1.092671821 \| 5.1419E-146 \| 17 \| \| MCM4 \| 3.3299E-150 \| 1.022283997 \| 7.2683E-146 \| 17 \| \| DDX39A \| 5.7066E-149 \| 1.18875622 \| 1.2456E-144 \| 17 \| \| MCM3 \| 1.2047E-145 \| 1.154510145 \| 2.6296E-141 \| 17 \| \| MCM7 \| 2.0807E-145 \| 1.238539856 \| 4.5416E-141 \| 17 \| \| USP1 \| 1.0231E-140 \| 1.210500239 \| 2.2332E-136 \| 17 \| \| TUBA1B \| 3.5441E-139 \| 2.290812463 \| 7.7356E-135 \| 17 \| \| HIST2H2AC \| 4.2597E-138 \| 1.224080314 \| 9.2977E-134 \| 17 \| \| HMGB1 \| 1.4854E-134 \| 1.640946373 \| 3.2422E-130 \| 17 \| \| CCDC167 \| 8.9223E-134 \| 1.127743528 \| 1.9475E-129 \| 17 \| \| ANP32B \| 2.4038E-127 \| 1.591969552 \| 5.2468E-123 \| 17 \| \| ITGAE \| 2.5941E-124 \| 1.020215185 \| 5.6621E-120 \| 17 \| \| CLEC2D \| 1.3456E-121 \| 1.011998918 \| 2.937E-117 \| 17 \| \| RAN \| 4.6572E-117 \| 1.415409443 \| 1.0165E-112 \| 17 \| \| RANBP1 \| 9.5957E-116 \| 1.403297306 \| 2.0944E-111 \| 17 \| \| RAC2 \| 5.0435E-115 \| 1.281559151 \| 1.1008E-110 \| 17 \| \| LRMP \| 4.0428E-112 \| 1.058738487 \| 8.8243E-108 \| 17 \| \| CBX5 \| 4.4323E-112 \| 1.073583697 \| 9.6743E-108 \| 17 \| \| SMC1A \| 1.9256E-111 \| 1.097929076 \| 4.203E-107 \| 17 \| \| SUZ12 \| 5.3882E-111 \| 1.090480119 \| 1.1761E-106 \| 17 \| \| HIST1H4C \| 6.9698E-111 \| 2.969234669 \| 1.5213E-106 \| 17 \| \| SMC3 \| 1.4908E-110 \| 1.251659794 \| 3.2539E-106 \| 17 \| \| LCP1 \| 1.4862E-104 \| 1.285923636 \| 3.2438E-100 \| 17 \| \| LSP1 \| 1.2571E-103 \| 1.030769176 \| 2.7439E-99 \| 17 \| \| RAD21 \| 5.7447E-101 \| 1.278359304 \| 1.2539E-96 \| 17 \| \| NASP \| 4.8159E-97 \| 1.054681992 \| 1.05117E-92 \| 17 \| \| PSIP1 \| 3.83257E-96 \| 1.105640638 \| 8.36536E-92 \| 17 \| \| IDH2 \| 1.96909E-91 \| 1.060132474 \| 4.29792E-87 \| 17 \| \| PARP1 \| 8.63542E-90 \| 1.243237964 \| 1.88485E-85 \| 17 \| \| GAPDH \| 1.58787E-87 \| 1.143186463 \| 3.46584E-83 \| 17 \| \| IFI16 \| 3.37046E-87 \| 1.019878131 \| 7.3567E-83 \| 17 \| \| ARHGDIB \| 8.35539E-86 \| 1.156677935 \| 1.82373E-81 \| 17 \| \| CD79B \| 2.35164E-85 \| 1.280604887 \| 5.13292E-81 \| 17 \| \| PSMB9 \| 2.49847E-82 \| 1.080293881 \| 5.45342E-78 \| 17 \| \| CALM3 \| 4.75006E-80 \| 1.106831624 \| 1.0368E-75 \| 17 \| \| ARPC2 \| 3.13646E-79 \| 1.183144232 \| 6.84595E-75 \| 17 \| \| NUCKS1 \| 1.1949E-76 \| 1.111933467 \| 2.60812E-72 \| 17 \| \| PFN1 \| 1.74707E-74 \| 1.286611234 \| 3.81334E-70 \| 17 \| \| MRPL51 \| 1.60091E-73 \| 1.026363183 \| 3.4943E-69 \| 17 \| \| COTL1 \| 1.51897E-71 \| 1.280375517 \| 3.31546E-67 \| 17 \| \| GZMB \| 6.80422E-67 \| 1.310358753 \| 1.48516E-62 \| 17 \| \| HMGN1 \| 8.23961E-66 \| 1.187865181 \| 1.79846E-61 \| 17 \| \| GZMA \| 3.00937E-65 \| 1.930175382 \| 6.56856E-61 \| 17 \| \| DEK \| 1.21909E-60 \| 1.344911275 \| 2.66091E-56 \| 17 \| \| SUB1 \| 2.27648E-57 \| 1.183099412 \| 4.96888E-53 \| 17 \| \| ACTB \| 2.55986E-57 \| 1.194309805 \| 5.58741E-53 \| 17 \| \| DUSP4 \| 1.80911E-55 \| 1.280587233 \| 3.94875E-51 \| 17 \| \| NKG7 \| 1.47865E-53 \| 1.152975424 \| 3.22745E-49 \| 17 \| \| S100A6 \| 9.06999E-49 \| -2.586691183 \| 1.97971E-44 \| 17 \| \| TXNIP \| 1.47302E-46 \| -1.858708195 \| 3.21517E-42 \| 17 \| \| MALAT1 \| 7.35041E-45 \| -1.067629686 \| 1.60437E-40 \| 17 \| \| FTL \| 9.43522E-38 \| -1.435730871 \| 2.05943E-33 \| 17 \| \| MZB1 \| 1.83662E-34 \| 1.465493218 \| 4.00878E-30 \| 17 \| \| IFI27 \| 2.7287E-34 \| -3.098445767 \| 5.95594E-30 \| 17 \| \| PHGR1 \| 2.33915E-33 \| -4.905296578 \| 5.10567E-29 \| 17 \| \| ELF3 \| 3.41396E-32 \| -2.43456794 \| 7.45164E-28 \| 17 \| \| FCGRT \| 3.68562E-32 \| -1.351251583 \| 8.0446E-28 \| 17 \| \| KRT8 \| 2.37191E-31 \| -3.031759624 \| 5.17718E-27 \| 17 \| \| CLDN3 \| 2.17181E-30 \| -2.200855375 \| 4.74041E-26 \| 17 \| \| EPCAM \| 2.82011E-30 \| -2.160446921 \| 6.15545E-26 \| 17 \| \| TMEM54 \| 4.6623E-30 \| -1.705895882 \| 1.01764E-25 \| 17 \| \| LGALS4 \| 8.46559E-30 \| -2.977482307 \| 1.84778E-25 \| 17 \| \| MARCKS \| 1.37216E-29 \| -1.5381768 \| 2.99501E-25 \| 17 \| \| APP \| 1.73491E-29 \| -1.324699027 \| 3.78679E-25 \| 17 \| \| LGALS3BP \| 4.90306E-29 \| -1.46253992 \| 1.07019E-24 \| 17 \| \| CDC42EP5 \| 6.05713E-29 \| -1.525781135 \| 1.32209E-24 \| 17 \| \| SMIM22 \| 6.30942E-29 \| -1.703439412 \| 1.37716E-24 \| 17 \| \| IER3 \| 8.99747E-29 \| -1.732635813 \| 1.96388E-24 \| 17 \| \| CD9 \| 1.44792E-28 \| -1.683010713 \| 3.16038E-24 \| 17 \| \| GSN \| 1.86807E-28 \| -1.806628141 \| 4.07744E-24 \| 17 \| \| CAMK2N1 \| 2.15504E-28 \| -1.432894633 \| 4.7038E-24 \| 17 \| \| KRT18 \| 2.71712E-28 \| -2.821841222 \| 5.93067E-24 \| 17 \| \| KRT19 \| 2.31613E-27 \| -2.954826052 \| 5.05542E-23 \| 17 \| \| CD63 \| 2.64665E-27 \| -1.280722523 \| 5.77683E-23 \| 17 \| \| CLDN4 \| 2.81438E-27 \| -2.356088676 \| 6.14295E-23 \| 17 \| \| IFITM3 \| 3.03865E-27 \| -1.697256086 \| 6.63247E-23 \| 17 \| \| TMEM176B \| 5.66024E-27 \| -1.26919398 \| 1.23546E-22 \| 17 \| \| TPM1 \| 9.53174E-27 \| -1.502518287 \| 2.08049E-22 \| 17 \| \| FAM3D \| 1.16238E-25 \| -1.778961923 \| 2.53712E-21 \| 17 \| \| DST \| 1.35309E-25 \| -1.401427199 \| 2.9534E-21 \| 17 \| \| DSP \| 2.16044E-25 \| -1.442768078 \| 4.71559E-21 \| 17 \| \| TIMP1 \| 2.40454E-25 \| -1.991136624 \| 5.2484E-21 \| 17 \| \| AGR2 \| 1.07009E-24 \| -2.753878767 \| 2.33568E-20 \| 17 \| \| KLF4 \| 1.07047E-24 \| -1.44643087 \| 2.33652E-20 \| 17 \| \| MUC13 \| 2.37125E-24 \| -1.614973611 \| 5.17572E-20 \| 17 \| \| FXYD3 \| 1.62189E-23 \| -2.66388899 \| 3.54009E-19 \| 17 \| \| ST14 \| 3.70037E-23 \| -1.139276832 \| 8.0768E-19 \| 17 \| \| SLC44A4 \| 3.86677E-23 \| -1.35781953 \| 8.43999E-19 \| 17 \| \| S100A14 \| 7.29058E-23 \| -1.365953443 \| 1.59132E-18 \| 17 \| \| LGALS3 \| 7.40111E-23 \| -2.010820698 \| 1.61544E-18 \| 17 \| \| TSPAN1 \| 1.84808E-21 \| -2.199836896 \| 4.0338E-17 \| 17 \| \| TSPAN8 \| 2.10785E-21 \| -2.324174559 \| 4.60081E-17 \| 17 \| \| ETS2 \| 2.55662E-21 \| -1.139028782 \| 5.58034E-17 \| 17 \| \| VEGFA \| 1.80679E-20 \| -1.194990284 \| 3.94369E-16 \| 17 \| \| PIGR \| 1.85274E-20 \| -4.218570617 \| 4.04398E-16 \| 17 \| \| KLF5 \| 4.94224E-20 \| -1.345879653 \| 1.07874E-15 \| 17 \| \| PHLDA2 \| 1.26291E-19 \| -1.079164331 \| 2.75656E-15 \| 17 \| \| CD24 \| 1.4645E-19 \| -1.754152477 \| 3.19656E-15 \| 17 \| \| ITGA6 \| 1.84554E-19 \| -1.065447847 \| 4.02826E-15 \| 17 \| \| EGR1 \| 3.41867E-19 \| -1.425643223 \| 7.46194E-15 \| 17 \| \| HES1 \| 3.51599E-19 \| -1.516558185 \| 7.67435E-15 \| 17 \| \| JUNB \| 1.38249E-18 \| -1.013532027 \| 3.01757E-14 \| 17 \| \| TSC22D1 \| 1.03082E-17 \| -1.097707949 \| 2.24996E-13 \| 17 \| \| GRN \| 1.54428E-17 \| -1.257496649 \| 3.37071E-13 \| 17 \| \| TFF3 \| 3.86454E-17 \| -3.831526979 \| 8.43514E-13 \| 17 \| \| CKB \| 1.00058E-16 \| -2.181155746 \| 2.18396E-12 \| 17 \| \| GPX2 \| 1.64314E-16 \| -1.529598206 \| 3.58649E-12 \| 17 \| \| ATP1B1 \| 5.40952E-16 \| -1.440871813 \| 1.18074E-11 \| 17 \| \| ID1 \| 4.50066E-15 \| -1.180864692 \| 9.82358E-11 \| 17 \| \| LCN2 \| 1.8535E-14 \| -2.351878933 \| 4.04563E-10 \| 17 \| \| YBX3 \| 1.82512E-13 \| -1.016603226 \| 3.98368E-09 \| 17 \| \| CEBPB \| 1.22857E-12 \| -1.188005053 \| 2.6816E-08 \| 17 \| \| SOX4 \| 1.51076E-12 \| -1.275115777 \| 3.29753E-08 \| 17 \| \| TSPAN3 \| 4.21417E-12 \| -1.006936541 \| 9.19828E-08 \| 17 \| \| CEBPD \| 4.46305E-12 \| -1.167005432 \| 9.7415E-08 \| 17 \| \| S100A11 \| 1.95555E-11 \| -1.095120643 \| 4.26838E-07 \| 17 \| \| PSAP \| 2.98192E-11 \| -1.328516734 \| 6.50864E-07 \| 17 \| \| FABP1 \| 7.43904E-11 \| -3.694101677 \| 1.62372E-06 \| 17 \| \| GLUL \| 1.2703E-10 \| -1.188674024 \| 2.77269E-06 \| 17 \| \| FTH1 \| 6.13065E-10 \| -1.245373467 \| 1.33814E-05 \| 17 \| \| S100A10 \| 3.28707E-09 \| -1.144079389 \| 7.17468E-05 \| 17 \| \| AREG \| 4.06559E-09 \| -1.058438922 \| 8.87397E-05 \| 17 \| \| SAT1 \| 1.96897E-08 \| -1.012013249 \| 0.000429767 \| 17 \| \| HEXIM1 \| 3.25295E-08 \| -1.016837482 \| 0.000710022 \| 17 \| \| PERP \| 1.17225E-07 \| -1.021760273 \| 0.002558664 \| 17 \| |
