# Supplementary material for: Combining single-cell analysis and molecular docking techniques to construct a prognostic model for colon adenocarcinoma and uncovering inhibin subunit βb as a novel therapeutic target
Source: Front Immunol. 2025 Jan 9;15:1524560. doi: 10.3389/fimmu.2024.1524560 (PMC11754261; doi:10.3389/fimmu.2024.1524560)
Supplement: Supplementary file 6 [file Table3.docx]

22 drugs with potential therapeutic prospects for INHBB

| Term | P-value | Adjusted P-value | Odds Ratio | Combined Score | Genes |
| --- | --- | --- | --- | --- | --- |
| syrosingopine PC3 DOWN | 0.001099976 | 0.029211966 | 19978 | 136099.4703 | INHBB |
| niclosamide PC3 DOWN | 0.00144997 | 0.029211966 | 19971 | 130534.6917 | INHBB |
| risperidone CTD 00007248 | 0.001549969 | 0.029211966 | 19969 | 129189.851 | INHBB |
| uric acid CTD 00006967 | 0.002049963 | 0.029211966 | 19959 | 123544.8878 | INHBB |
| 0297417-0002B PC3 DOWN | 0.003599946 | 0.040849422 | 19928 | 112131.5984 | INHBB |
| rifabutin PC3 DOWN | 0.004299939 | 0.040849422 | 19914 | 108514.461 | INHBB |
| valproic acid MCF7 DOWN | 0.006049925 | 0.048607929 | 19879 | 101536.1561 | INHBB |
| HC toxin MCF7 DOWN | 0.007549914 | 0.048607929 | 19849 | 96986.56292 | INHBB |
| trichostatin A ssMCF7 DOWN | 0.008049911 | 0.048607929 | 19839 | 95665.52833 | INHBB |
| 5,6-BENZOFLAVONE CTD 00007276 | 0.009099904 | 0.048607929 | 19818 | 93134.5205 | INHBB |
| NICKEL CTD 00006389 | 0.009449902 | 0.048607929 | 19811 | 92353.94708 | INHBB |
| MG-132 MCF7 DOWN | 0.010349897 | 0.048607929 | 19793 | 90469.42323 | INHBB |
| 1-NITROPYRENE CTD 00001569 | 0.01169989 | 0.048607929 | 19766 | 87922.64429 | INHBB |
| cicloheximide PC3 DOWN | 0.012349886 | 0.048607929 | 19753 | 86796.82367 | INHBB |
| piperlongumine MCF7 DOWN | 0.013099883 | 0.048607929 | 19738 | 85567.23038 | INHBB |
| MS-275 PC3 DOWN | 0.014699875 | 0.048607929 | 19706 | 83157.67015 | INHBB |
| lycorine PC3 DOWN | 0.015099873 | 0.048607929 | 19698 | 82595.07156 | INHBB |
| anisomycin MCF7 DOWN | 0.015349872 | 0.048607929 | 19693 | 82250.73149 | INHBB |
| thapsigargin PC3 DOWN | 0.016449868 | 0.048856767 | 19671 | 80797.40988 | INHBB |
| vorinostat PC3 DOWN | 0.017749862 | 0.048856767 | 19645 | 79196.41123 | INHBB |
| MG-262 PC3 DOWN | 0.017999861 | 0.048856767 | 19640 | 78901.5635 | INHBB |
| daunorubicin PC3 DOWN | 0.019049858 | 0.049356449 | 19619 | 77704.88811 | INHBB |
